# Phenylalanine‐Based DNA‐Encoded Chemical Libraries for the Discovery of Potent and Selective Small Organic Ligands Against Markers of Cancer and Immune Cells

**DOI:** 10.1002/advs.202505351

**Published:** 2025-06-29

**Authors:** Francesca Migliorini, Andrea Ciamarone, Sheila Dakhel Plaza, Tony Georgiev, Marta Mascellani, Emanuela Sabato, Giulio Vistoli, Ilaria Biancofiore, Nicholas Favalli, Emanuele Puca, Sebastian Oehler, Dario Neri, Samuele Cazzamalli

**Affiliations:** ^1^ Philochem AG Otelfingen CH‐8112 Switzerland; ^2^ Department of Pharmacy and Biotechnology University of Bologna Bologna I‐40126 Italy; ^3^ Computational and Chemical Biology Istituto Italiano di Tecnologia Genoa I‐16163 Italy; ^4^ Department of Pharmaceutical Sciences University of Milano Milano I‐20133 Italy; ^5^ Department of Chemistry and Applied Biosciences Swiss Federal Institute of Technology (ETH Zurich) Zurich CH‐8093 Switzerland; ^6^ Philogen SPA Siena I‐53100 Italy

**Keywords:** DNA‐encoded chemical library, immunological target, small molecule radiopharmaceuticals, target therapy, tumor‐associated antigen

## Abstract

DNA‐encoded chemical libraries (DELs) are powerful tools for drug discovery, enabling the high‐throughput screening of vast libraries of small molecules against target proteins of pharmaceutical interest. Here, the synthesis of two new DELs, named FM‐DEL1 and FM‐DEL2, including 7′710 and 5′697’690 compounds, respectively is described. These libraries are constructed by installing one or two sets of building blocks on a phenylalanine central scaffold. FM‐DELs are screened against markers of prostate cancer, and renal cell carcinoma, and against an immunological target expressed on the surface of natural killer cells. Highly potent and selective binders with affinity constants in the nanomolar range are obtained from DEL screenings against those targets. Small‐molecule ligands against tumor‐associated antigens are used to develop small‐molecule radiopharmaceuticals that selectively accumulate at cancer sites after systemic administration.

## Graphical abstract

FM‐DEL1 and FM‐DEL2 are constructed by installing one or two sets of building blocks on a phenylalanine central scaffold. FM‐DELs are screened against markers of prostate, and renal cell carcinoma, and against an immunological target expressed on the surface of natural killer cells. Highly potent and selective binders with affinity constants in the nanomolar range are isolated for those targets.

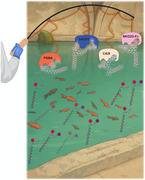

## 1 Introduction

DNA‐encoded chemical libraries (DELs) have emerged as powerful tools for the discovery of small organic ligands against protein targets of pharmaceutical interest.^[^ ^1^, ^2^ ^]^ By combining the principles of combinatorial chemistry with DNA encoding, DELs enable the rapid construction of vast libraries of small molecules, allowing for the parallel screening of millions of compounds at a fraction of the cost of traditional methods.^[^ ^3^ ^]^ The technology has shown remarkable potential in identifying ligands with high affinity and exceptional selectivity, even against targets traditionally deemed “undruggable”.^[^ ^4^, ^5^, ^6^, ^7^ ^]^ The impact of DEL technology on drug discovery has grown exponentially, with a number of DEL‐derived compounds entering into Phase I clinical trials.^[^ ^8^, ^9^, ^10^ ^]^

While monoclonal antibodies (mAbs) have been instrumental in cancer treatment–especially in the form of antibody‐drug conjugates^[^ ^11^ ^]^ and bispecific antibodies^[^ ^12^ ^]^–their effectiveness in solid tumors is often limited. This is primarily due to their slow extravasation and poor penetration into tumor masses.^[^ ^13^, ^14^ ^]^ Small organic ligands, in contrast, exhibit faster pharmacokinetics and more efficient tumor penetration, offering a promising alternative to antibody‐based therapies.^[^ ^13^, ^15^ ^]^ DEL screenings are increasingly used to identify small molecules that can bind with high affinity to cancer biomarkers, providing a new approach for developing targeted therapies that overcome the limitations of mAb‐based treatments.^[^ ^7^, ^16^, ^17^ ^]^

Cancer lesions often overexpress tumor‐associated antigens (TAAs) that are absent in normal tissues,^[^ ^18^ ^]^ presenting a unique opportunity for selective delivery of diagnostic and therapeutic agents.^[^ ^19^, ^20^, ^21^ ^]^ However, the high sequence similarity between these tumor proteins and homologous proteins in normal tissues complicates the development of specific ligands. Our group has demonstrated that parallel DNA‐encoded chemical library (DEL) screenings against tumor targets and their closely related anti‐targets can identify small molecules that effectively distinguish between these similar proteins, offering a strategy for generating selective tumor‐targeting ligands.^[^ ^7^ ^]^ Prostate‐specific membrane antigen (PSMA), a membrane protein overexpressed in prostate cancer (PCa) cells, is a key target in prostate cancer therapy.^[^ ^21^, ^22^ ^]^ However, PSMA‐targeted therapies like ^177^Lu‐PSMA‐617 (Pluvicto) face challenges, including dose‐limiting toxicities due to off‐target accumulation in healthy PSMA‐positive tissues (e.g., salivary glands and kidneys),^[^ ^23^, ^24^ ^]^ as well as cross‐reactivity with other glutamate carboxypeptidases (GCPs), such as GCP3.^[^ ^22^, ^25^ ^]^ In contrast, prostatic acid phosphatase (ACP3) may serve as a superior PCa target,^[^ ^26^, ^27^, ^28^, ^29^ ^]^ as it is expressed in most prostate cancer lesions but undetectable in healthy tissues.^[^ ^30^ ^]^ The identification of new PSMA ligands that distinguish PSMA from other GCPs^23^, along with the development of ACP3 binders, could significantly improve PCa diagnosis and treatment. Another promising cancer target is carbonic anhydrase IX (CAIX), overexpressed in clear cell renal cell carcinoma (ccRCC) and other hypoxic tumors.^[^ ^31^, ^32^ ^]^ However, selective targeting of CAIX is challenging due to its high homology with other carbonic anhydrases, which are expressed in normal tissues like the kidneys and stomach, limiting therapeutic applications.^[^ ^31^, ^32^, ^33^ ^]^

The identification of ligands targeting cancer‐associated antigens such as PSMA, ACP3, and CAIX enables the construction of tumor‐targeted therapies that deliver radionuclides (e.g., Lutetium‐177)^[^ ^19^, ^21^ ^]^ or cytotoxic drugs (e.g., Monomethyl auristatin E‐MMAE).^[^ ^13^, ^34^, ^35^, ^36^ ^]^ Additionally, the identification of small organic binders for immune cell markers could facilitate the development of small‐molecule‐based bispecifics, including those targeting natural killer (NK) cells.^[^ ^37^, ^38^ ^]^ NK‐cell‐targeting bispecifics may offer an alternative to T‐cell bispecifics,^[^ ^39^, ^40^ ^]^ which are associated with cytokine release syndrome (CRS) and neurological toxicity. NKG2D, an activating receptor on NK cells, plays a critical role in identifying and eliminating tumor cells.^[^ ^41^ ^]^ Despite the challenges they pose for DEL screening, these membrane‐associated targets have been prioritized due to their therapeutic relevance in the field of oncology^[^ ^21^, ^30^, ^32^, ^41^ ^]^ and the presence of druggable pockets suitable for small organic molecules.^[^ ^22^, ^32^, ^42^, ^43^ ^]^

Here, we report the design, synthesis, and screening of two novel DNA‐encoded chemical libraries, FM‐DEL1 and FM‐DEL2, to identify new tumor‐targeting ligands and small‐molecule immunomodulators. FM‐DEL1, a single building block library, contains 7′710 compounds, while FM‐DEL2, a double building block library, includes 5′620’590 compounds. These libraries were screened in parallel against CAIX, PSMA, ACP3, NKG2D, and closely related protein targets to discover highly specific and potent ligands for cancer treatment and immunotherapy.

## 2 Results

### 2.1 Design and Construction of Phenylalanine‐Based DNA‐Encoded Chemical Libraries

**Figure 1:**
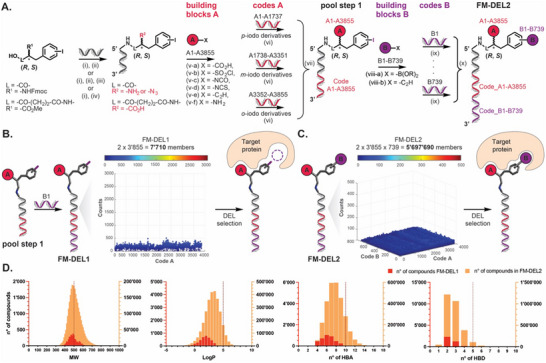
Design, synthetic scheme, and physicochemical properties of FM‐DEL1 and FM‐DEL2. A) Racemic mixtures of three regioisomers of Fmoc‐*iodo*‐phenylalanine were conjugated to a 14‐mer amino‐modified oligonucleotide via amide bond formation i). After Fmoc removal ii), an aliquot underwent on‐DNA diazo‐transfer iii). Regioisomers of 4‐((3‐(iodophenyl)‐1‐methoxy‐1‐oxopropan‐2‐yl)amino)‐4‐oxobutanoic acid were conjugated via amide bond (i), followed by COOMe deprotection iv). Carboxylic acids, sulfonyl chlorides, isocyanates, isothiocyanates, alkynes, and amines (highlighted in red) were individually reacted with the appropriate phenylalanine scaffolds, and the resulting 3′855 products were purified by RP‐HPLC v). Conjugates were encoded with oligonucleotides A1–A3855 (red) via enzymatic splint ligation vi), pooled, and purified by RP‐HPLC at 60 °C vii) to yield pool step 1. This pool was further derivatized with 611 boronic acids viii‐a) and 128 alkynes viii‐b), and encoded with oligonucleotides B1–B739 (purple) via splint ligation ix). The final derivatives were pooled and purified by RP‐HPLC at 60 °C x), forming FM‐DEL2. B) Pool step 1 (7′710 members), encoded with a code B, yielded FM‐DEL1. High‐throughput DNA sequencing (HTS) of FM‐DEL1 before selections (Naïve FM‐DEL1) is shown as a 2D fingerprint: the x‐axis indicates building blocks A, and the y‐axis shows the normalized sequence counts (color‐coded). C) FM‐DEL2 contains 5′697’690 members. HTS of FM‐DEL2 before selections (Naïve FM‐DEL2) is shown as a 3D fingerprint: x‐ and y‐axes represent the building blocks A and B, and the z‐axis and dot color indicate the normalized sequence counts. FM‐DEL1 and FM‐DEL2 were screened in parallel with different targets. D) Physicochemical properties (MW, LogP, HBA, HBD) of FM‐DEL1 (red) and FM‐DEL2 (orange) were calculated using OSIRIS DataWarrior.

**Figure** **1** illustrates the design of FM‐DEL1 and FM‐DEL2, together with their calculated physicochemical properties. Figure 1A details the scheme that was followed to synthesize FM‐DEL1 and FM‐DEL2. The central scaffold in the two DELs is based on the three regioisomers of *iodo*‐phenylalanine (*ortho*, *meta*, *para*), which were encoded using different codes A. Both configurations (*S* and *R*) of the stereocenter in alpha on the scaffold were included in the libraries (racemic mixture). The carboxylic acid moiety of the scaffolds was conjugated by amide‐bond coupling to a universal 5′ amino‐modified 14‐mer oligonucleotide (5′ NH_2_‐C_6_‐GGAGCTTCTGAATT 3′). Building blocks A (including carboxylic acids, alkynes, sulfonamides, isocyanates, and isothiocyanates) were installed after optimization of reaction conditions to expand the library's chemical diversity [Supporting Information].^[^ ^44^ ^]^ Succinic acid was used as a spacer to derivatize the ‐COOH moiety of the phenylalanine with aliphatic amines. To ensure the identity and structural integrity of each library member, the conjugates were purified one by one by HPLC. Each product was EtOH precipitated, and separately redissolved in mQ, obtaining 3′855 highly pure derivatives that were separately encoded via enzymatic splint ligation with 31‐mer oligonucleotides (codes A, A1‐A3’855; single strand 5′ PO_4_‐CTGTGTGCTGXXXXXXCGAGTCCCATGGCGC 3′). The encoded conjugates were pooled and purified by HPLC to obtain pool step 1, which contains 3′855 conjugates in their *S* and *R* configuration (a total of 7′710 conjugates) [Figure 1A]. Pool step 1 was elongated with code B1 (5′ PO_4_‐CGGATCGACGGTCTCACGCGTCAGGCAGC 3′) to obtain a one‐building block library named FM‐DEL1 based on a 45‐mer oligonucleotide [Figure 1B].

Part of pool step 1 was further derivatized with building block B, including 611 boronic acids and 128 alkynes on the iodo moiety of the scaffolds via Suzuki and Sonogashira cross‐couplings. These coupling reactions were chosen to expand the covered chemical space, as they ensure high conversion rates under DNA‐compatible conditions, enabling the efficient incorporation of a second set of building blocks by generating new C‐C bonds. As Suzuki and Sonogashira couplings are performed in the pool on FM‐DEL1, only building blocks that reacted with a conversion rate of over 80% were employed (611 boronic acids and 128 alkynes). The 739 reactions were encoded *vi* *a* enzymatic splint ligation with a 29‐mer oligonucleotide (codes B, B1‐B739; single strand 5′ PO_4_‐CGGATCGACGXXXXXXXGCGTCAGGCAGC), thus yielding a final length of 45 nucleotides. FM‐DEL2 was obtained by pooling and RP‐HPLC purification [Figure 1A; Supporting Information]. The library contains a total of 5′697’690 compounds, obtained by combining 3′855 conjugates in their *S* and *R* configuration (a total of 7′710 conjugates) together with 739 building blocks B [Figure 1C]. Building blocks A and B used for the synthesis of FM‐DEL1 and FM‐DEL2 include structures that were used for the synthesis of a smaller phenylalanine‐based DEL (including a total of 670′752 compounds) that was previously described by our group.^[^ ^45^ ^]^ These building blocks are designated as A1‐204, A1738‐1941, A3352‐3555, and B1‐384, B612‐723.

FM‐DEL1 against FM‐DEL2 screenings can be directly compared as a sequencing strategy, and the length of the oligonucleotide codes is matched (45‐mer), thus minimizing differences between selections [Figure 1B]. Prior to selection experiments, unselected libraries (Naïve DELs) were amplified by polymerase chain reaction (PCR) and sequenced by high‐throughput DNA sequencing (HTS), revealing high DELs quality with a homogeneous count distribution of all library members [Figure 1B,C; Supporting Information]. Both FM‐DEL1 and FM‐DEL2 were screened in parallel with different target proteins, as generally illustrated in Figure 1B, and as detailed in the rest of this paper. Hits were nominated based on their enrichment factor (EF), which is calculated as the number of sequencing counts in the target selection divided by the average count (AC) obtained in the same selection [Experimental Section].

After the enumeration of the DELs, physicochemical properties of the entire molecule repertoire of FM‐DEL1 (in red, Figure 1D) and FM‐DEL2 (in orange, Figure 1D) were calculated using OSIRIS DataWarrior. The average molecular weight (MW) of FM‐DEL1 and FM‐DEL2 was ≈500 Da, with the MW spanning from ≈400 to ≈600 Da and from ≈300 to ≈700 Da for FM‐DEL1 and FM‐DEL2, respectively. The addition of building block B to obtain FM‐DEL2 from FM‐DEL1 led to the generation of a library with a larger size and with a slightly higher average LogP. A higher number of hydrogen bond acceptors and donors (HBA and HBD) is available in FM‐DEL2 as compared to FM‐DEL1. Overall, the physicochemical properties of compounds included in FM‐DELs are compatible with the Lipinski rules of five [Figure 1D].^[^ ^46^ ^]^

FM‐DEL1 and FM‐DEL2 were screened against a series of tumor‐associated antigens (i.e., PSMA, ACP3, and CAIX), an immunological NK‐cell target (i.e., NKG2D), and closely related proteins (“anti‐targets”).^[^ ^7^ ^]^ All targets and anti‐targets were produced as purified recombinant proteins by transient gene expression in CHO cells. Prior to DEL selections, the proteins were biotinylated, as indicated in Supporting Information. Parallel DEL screenings against pharmaceutical targets and closely related anti‐targets were conducted as a useful approach to assess the selectivity profile of DEL hits at the fingerprint level.^[^ ^7^ ^]^ Binding selectivity was subsequently confirmed through orthogonal in vitro hit validation activities.

### 2.2 FM‐DEL Screening and Hit‐Validation Against Prostate Cancer Markers

The development of highly selective PSMA ligands is a challenging task due to the close sequence similarity between PSMA and other glutamate carboxypeptidases.^[^ ^22^ ^]^ For instance, GCP3 (a PSMA anti‐target) is a transmembrane protein expressed in healthy tissues (e.g., kidneys and salivary glands) that shares 83% sequence similarity with PSMA.^[^ ^22^ ^]^ This overlap can cause cross‐reactivity of glutamate‐based PSMA ligands such as Pluvicto and may lead to their accumulation in kidney and salivary glands and contribute to side effects such as xerostomia and renal insufficiency.^[^ ^21^, ^47^ ^]^

**Figure 2:**
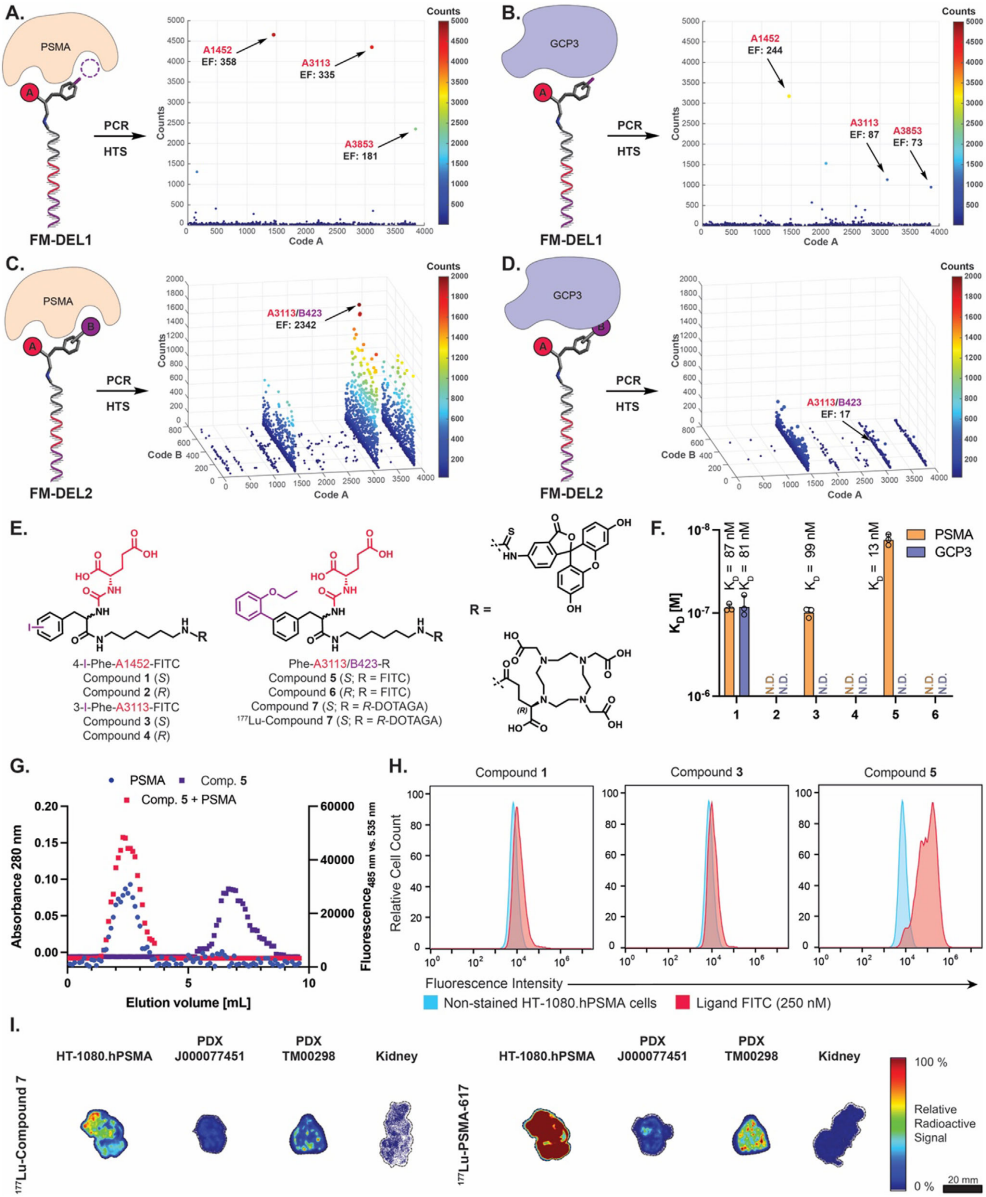
Screening of FM‐DELs and corresponding hit‐validation results against prostate‐specific membrane antigen (PSMA) and glutamate carboxypeptidase III (GCP3). FM‐DEL1 screening results (HTS) are represented as 2D‐plots after selections against PSMA (A, total counts TCs 51′427, average counts AC 13, cut‐off 20) and against GCP3 (B, TCs 49′445, AC 13, cut‐off 20). FM‐DEL2 screening results (HTS) are represented as 3D plots after selections against PSMA (C, TCs 2′389’572, AC 0.84, cut‐off 30) and against GCP3 (D, TCs 2′440’903, AC 0.85, cut‐off 30). DEL selections were performed in duplicate (*n* = 2) (Figures S20 and S21, Supporting Information). The most enriched PSMA hits identified from FM‐DEL1 and FM‐DEL2 are indicated with arrows, along with the corresponding enrichment factors (EFs). E) Chemical structures of hit compounds 1–7. F) Fluorescence polarization (FP) affinity constant values (K_D_) of compounds 1–6 against PSMA (in orange) and GCP3 (in violet). G) Binding of compound 5 to PSMA in a size‐exclusion chromatography co‐elution assay (GCP3 co‐elution shown in Figure S40, Supporting Information). H) Flow cytometry analysis for compounds 1, 3, and 5 on HT‐1080.hPSMA cells (additional data in Figure S43, Supporting Information). I) Autoradiography results obtained for ^177^Lu‐compound 7 and ^177^Lu‐PSMA‐617 (Pluvicto) on tumor tissues (HT‐1080.hPSMA, PDX J000077451, and PDX TM00298) and healthy human kidney specimens.

FM‐DEL1 and FM‐DEL2 were screened against PSMA and GCP3, with the goal of identifying PSMA‐selective ligands [**Figure** **2**A–D]. Three enriched hits in PSMA selections were identified from FM‐DEL1, corresponding to (*S*)‐2‐isocyanatopentanedioic acid coupled with *para*, *meta*, and *ortho* *iodo*‐phenylalanine, respectively (**A1452** EF_PSMA_ = 358; **A3113** EF_PSMA_ = 335; **A3853** EF_PSMA_ = 181). *Para* and *meta* derivatives (top enriched hits in PSMA selections) presented different selectivity against GCP3 (**A1452** EF_PSMA_ /EF_GCP3_ = 1; **A3113** EF_PSMA_ /EF_GCP3_ = 4) [Figure 2A,B]. Hits including the (*S*)‐2‐isocyanatopentanedioic acid building block A were also enriched in FM‐DEL2 screenings on PSMA and GCP3. Specifically, a line of compounds featuring the **A3113** building block [(*S*)‐2‐isocyanatopentanedioic acid coupled with 3‐*iodo*‐phenylalanine scaffold] was preferentially enriched in PSMA selections. **A3113/B423** was identified as the most enriched and selective combination for PSMA (EF_PSMA_ = 2′342; EF_PSMA_ /EF_GCP3_ = 138) [Figure 2C,D].

Hits obtained from FM‐DEL1 (**A1452** and **A3113**) and FM‐DEL2 (**A3113/B423**) were re‐synthesized as stereo‐chemically pure off‐DNA compounds derivatized with a C_6_ linker featuring a fluorescein moiety (coupled to fluorescein isothiocyanate‐FITC) (compounds **1**–**6**) [Figure 2E]. The binding affinity against PSMA and GCP3 (K_D_) of fluoresceinated compounds **1**–**6** was measured by fluorescence polarization (FP). Our results indicate that stereochemistry influenced binding on both PSMA and GCP3, with all compounds in the *S* configuration (**1**, **3**, and **5**) revealing higher affinity as compared to their *R* counterparts (**2**, **4**, and **6**) [Figure 2F]. The superiority of compound **5** (*S*‐configuration) in binding PSMA over compound **6** (*R*‐configuration) was confirmed by molecular docking studies [Figure S48, Supporting Information]. Compound **1** (derived from **A1452**) was found to cross‐react with both PSMA and GCP3, exhibiting K_D_ values of 87 and 81 nM, respectively. By contrast and in line with DEL screening results, compounds **3** and **5** (*meta* derivatives derived from **A3113** and **A3113/B423**) displayed selective binding to PSMA, with K_D_ values of 99 nM and 13 nM, respectively. The presence of the sole building block **A3113** on the 3‐*iodo*‐phenylalanine scaffold in the *S*‐configuration gives adequate selectivity for PSMA over GCP3. The addition of the second building block (**B423**) enhances both affinity and selectivity for the target. Compound **5** was able to form a selective and kinetically stable interaction with PSMA, as demonstrated by co‐elution with recombinant PSMA and GCP3 in size‐exclusion chromatography experiments [Figure 2G; Figure S40, Supporting Information].

To evaluate the ability of the compounds to bind to PSMA expressed on the cell surface, a flow cytometry analysis was conducted on HT‐1080.hPSMA tumor cells incubated with compounds **1**, **3**, and **5**. The results showed that only compound **5** was able to bind to the antigen expressed on the tumor cell surface [Figure 2H; Figure S43, Supporting Information].

A DOTAGA derivative of hit **A3113/B423** was synthesized, affording compound **7** that was labeled with Lutetium‐177 (^177^Lu) [Figure 2E] to study binding on healthy tissues and tumor prostate cancer lesions by autoradiography assays. The binding of ^177^Lu‐compound **7** on HT‐1080.hPSMA, PDX J000077451, PDX TM00298, and healthy human kidney was studied in comparison with ^117^Lu‐PSMA617 (Pluvicto) [Figure 2I]. ^177^Lu‐compound **7** exhibited a more selective but less intense uptake on tumor tissues as compared to Pluvicto, with a reduced uptake on healthy kidney tissues.

**Figure 3:**
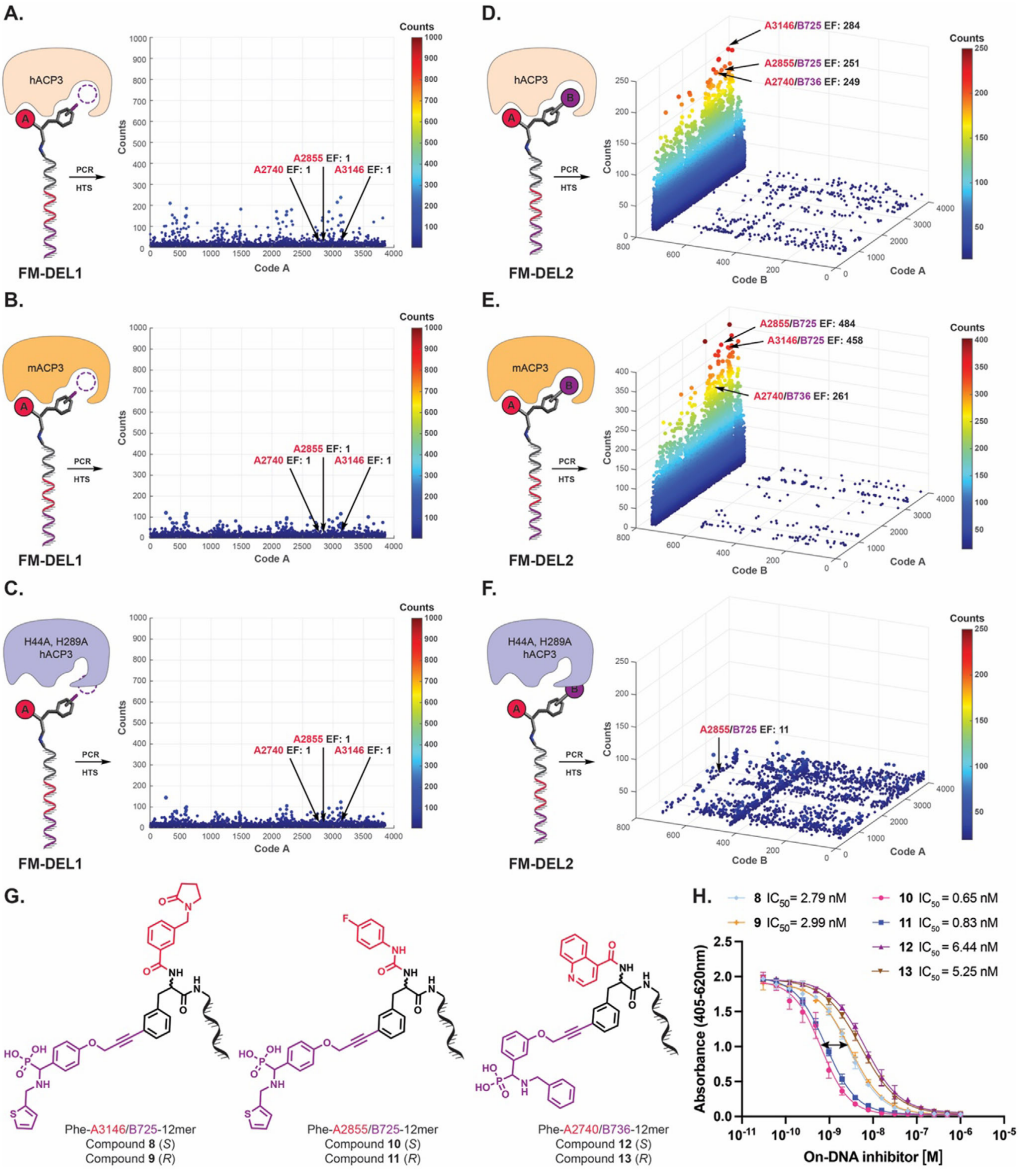
Screening of FM‐DELs and corresponding on‐DNA hit‐validation results against prostatic acid phosphatase variants (human, murine, and mutated ACP3). FM‐DEL1 screening results (HTS) are represented as 2D‐plots after selections against human ACP3 (A, TCs 59′011, AC 15, cut‐off 5), murine ACP3 (B, TCs 52′270, AC 14, cut‐off 5), and double mutant ACP3 (C, TCs 55′642, AC 14, cut‐off 5). FM‐DEL2 screening results (HTS) are represented as 3D plots after selections against human ACP3 (D, TCs 2′254’758, AC 0.85, cut‐off 15), murine ACP3 (E, TCs 2′102’747, AC 0.74, cut‐off 15), and double mutant ACP3 (F, TCs 2′466’870, AC 0.87, cut‐off 20). DEL selections were performed in duplicate (*n* = 2) (Figures S22 and S23). The most enriched human ACP3 hits identified from FM‐DEL2 are indicated with arrows, along with the corresponding EFs. G) Chemical structures of on‐DNA hit compounds 8–13. H) On‐DNA ACP3 enzymatic inhibition assay results for compounds 8–13. Data are presented as mean ± standard error of the mean (SEM) (*n* = 3).

FM‐DEL1 and FM‐DEL2 were screened for activity against recombinant human ACP3 (hACP3 or PAP), a prostate cancer‐associated antigen alternative to PSMA.^[^ ^26^, ^27^, ^28^ ^]^ To identify potential hits with cross‐species reactivity, FM‐DELs were also tested against murine recombinant ACP3 (mACP3), which facilitates future translational activities. Additionally, we designed and expressed a mutant form of human ACP3, where two histidine residues (H44 and H289) in the phosphatase active site were substituted with alanine. FM‐DELs were then screened against the H44A, H289A hACP3 mutant to confirm or exclude binding to the active site. The results of these DEL screenings against hACP3, mACP3, and the H44A, H289A hACP3 mutant are shown in **Figure** **3**A–F. Screening results against tissue non‐specific alkaline phosphatase (TNAP, irrelevant phosphatase control) are presented in Figures S22 and S23 (Supporting Information).

No hits were found to be enriched in FM‐DEL1 selections against ACP3 variants [Figure 3A–C]. By contrast, several chemotypes based on phosphonate derivatives installed in position B on the *iodo*‐phenylalanine scaffold were identified as hits in FM‐DEL2 selections against hACP3 and mACP3 [Figure 3D–F]. Our selection results indicate species cross‐reactivity. Interestingly, all ACP3 hits were not enriched when FM‐DEL2 was screened against an irrelevant phosphatase (TNAP) and against the double mutant hACP3 (H44A, H289A hACP3), suggesting high selectivity and binding in the active site of the protein. **A2740/B736**, **A2855/B725**, and **A3146/B725** were identified as the most enriched building block combinations, with EFs above 240 in both hACP3 and mACP3 selections. *S* and *R* stereoisomers of these hits were re‐synthesized as DNA conjugates coupled with a single‐strand 12‐mer oligonucleotide (5′ NH_2_‐C_6_‐TAGTAGCCATCC 3′), yielding compounds **8**–**13** [Figure 3G]. Hits were tested for their ability to inhibit the in vitro enzymatic activity of hACP3. Compounds **10** (*S*‐**A2855/B725**) and **11** (*R*‐**A2855/B725**) were identified as the most potent inhibitors, with IC_50_ values of 0.65 and 0.83 nM, respectively [Figure 3H].

**Figure 4:**
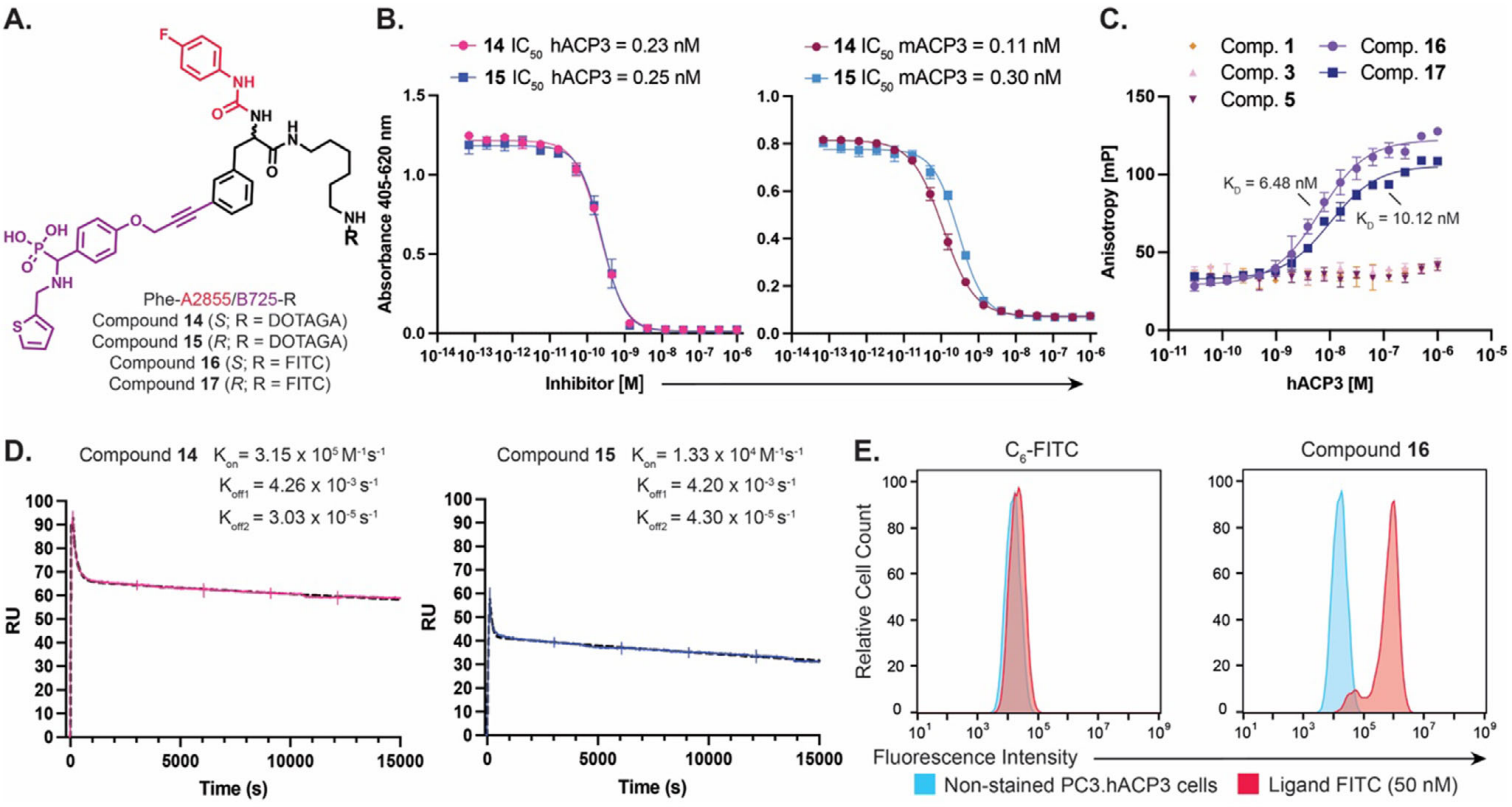
Off‐DNA hit‐validation of FM‐DEL‐derived ACP3 ligands. A) Chemical structures of hit compounds 14–17. B) Off‐DNA ACP3 enzymatic inhibition assay for compounds 14 and 15 with human ACP3 (hACP3, left) with murine ACP3 (mACP3, right). Data are presented as mean ± standard error of the mean (SEM) (*n* = 3). C) Affinity measurement of compounds 16 and 17 by FP against human ACP3. Validated PSMA ligands are included as negative controls. D) Surface plasmon resonance (SPR) analysis of compounds 14 and 15 binding to hACP3 immobilized on a CM5 chip (reaching 4′020 to 4′150 response units, RUs). Compounds 14 and 15 were injected as 1000 nM solutions in PBS. E) Assessment of binding on ACP3‐positive cells (PC3.hACP3) by flow cytometry. Cells were incubated with compound 16 or with C_6_‐FITC (non‐binding negative control). Additional results are reported in Figure S44 (Supporting Information).

Enantiomeric derivatives of hit **A2855/B725** (*R* and *S*) were synthesized replacing the corresponding DNA tag with either DOTAGA (compounds **14** and **15**) or FITC (compounds **16** and **17**) [**Figure** **4**A]. Activity hACP3 assay revealed that DOTAGA derivatives **14** and **15** potently inhibit the activity of the enzyme, with similar IC_50_ in the subnanomolar range (IC_50_ = 0.23 and 0.25 nM, respectively) [Figure 4B]. As observed by our group for other targets, the presence of the DNA can partially disturb the interaction with the target, possibly by steric hindrance.^[^ ^7^ ^]^ Notably, the potency of hit **A2855/B725** was enhanced when DNA was replaced by a small payload such as DOTAGA of a factor ≈3X. The activity of compounds **14** and **15** was retained when tested against the murine isozyme (mACP3) [Figure 4B]. Affinity constants of fluorescein derivatives of **A2855/B725** (*S* and *R*; compounds **16** and **17**) were measured by FP on recombinant human ACP3 (K_D_ = 6.48 and 10.12 nM, respectively). Hit compounds previously identified from PSMA selections using FM‐DEL1 and FM‐DEL2 (compounds **1**, **3**, and **5**) did not bind to the target in the same assay, as expected (negative controls) [Figure 4C].

Binding kinetics on hACP3 of compounds **14** and **15** were measured by surface plasmon resonance (SPR). A fast association was observed for both molecules (k_on_ = 3.15 × 10^5^ M^−1^s^−1^ and 1.33 × 10^4^ M^−1 ^s^−1^, respectively). A biphasic dissociation was measured for the two compounds, with a first phase with a rapid dissociation rate (k_off1_ = 4.26 × 10^−3^ s^−1^ and 4.20 × 10^−3^ s^−1^, respectively) and a second slow dissociation phase (k_off2_ = 3.03×10^−5^ s^−1^ and 4.30×10^−5^ s^−1^, respectively) [Figure 4D]. Slow dissociation from the target is seen as an advantage, especially for tumor therapy purposes, as it may reflect longer target exposure to radioactive emissions.

The binding of (*S)*‐**A2855/B725** (**16**), the most potent ACP3 binder identified from the FM‐DEL platform, on tumor cells was further investigated on ACP3‐positive cancer cells (PC3.hACP3).^[^ ^30^ ^]^ The compound bound to ACP3‐positive cells, as demonstrated by a fluorescence shift in flow cytometry experiments in comparison to cells incubated with a non‐targeted fluorescein control (C_6_‐FITC), non‐stained cells, and ACP3‐negative cancer cells [Figure 4E; Figure S44, Supporting Information].

### 2.3 FM‐DEL Screening and Hit‐Validation Against CAIX, a Marker of ccRCC and Hypoxia

**Figure 5:**
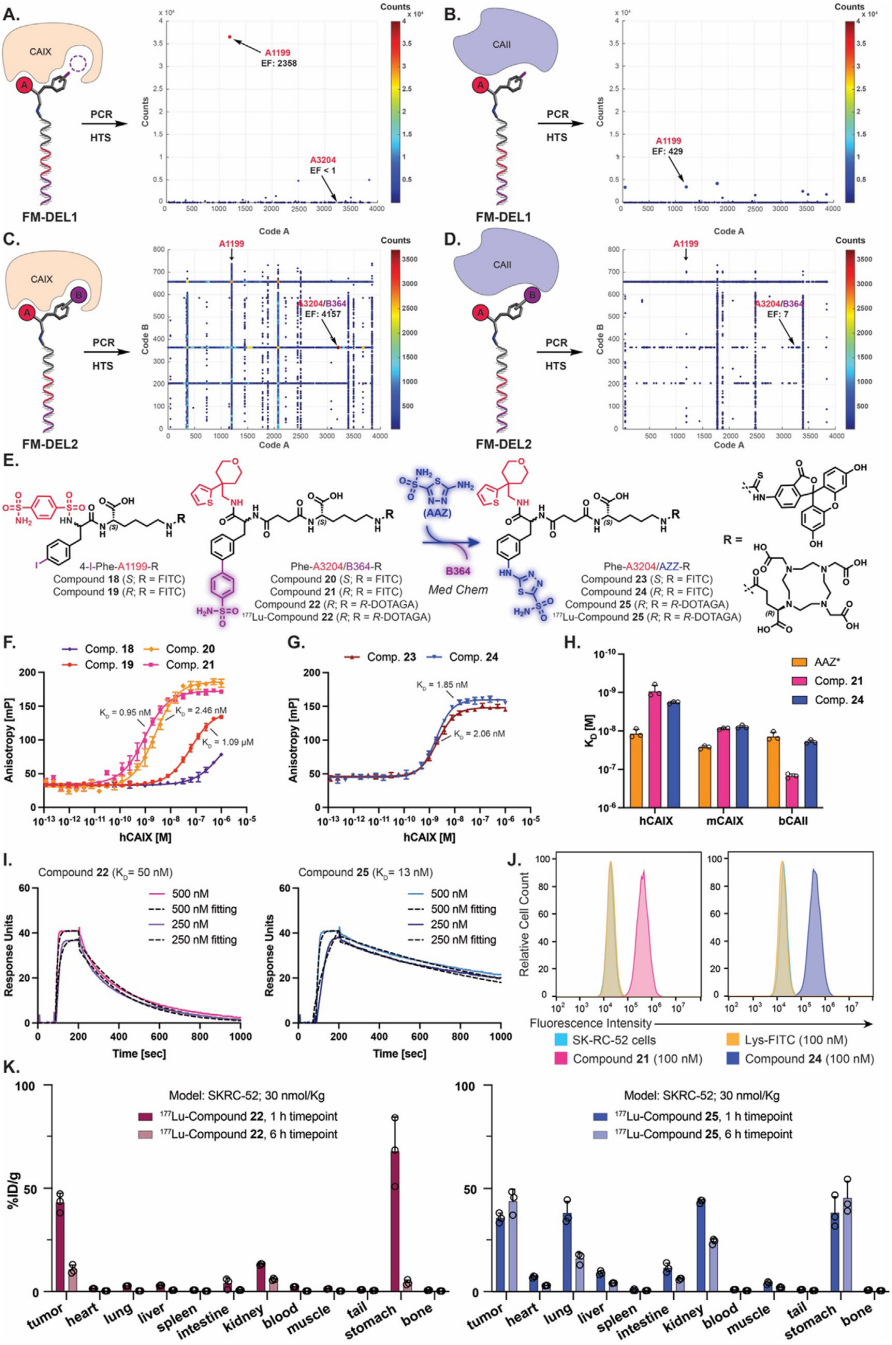
Screening of FM‐DELs, corresponding hit‐validation results against carbonic anhydrases, and in vivo biodistribution studies. FM‐DEL1 screening results (HTS) are represented as 2D‐plots after selections against CAIX (A, TCs 59′564, AC 8, cut‐off 5) and against CAII (B, TCs 30′531, AC 8, cut‐off 5). FM‐DEL2 screening results (HTS) are represented as 3D plots after selections against CAIX (C, TCs 2′546’972, AC 0.89, cut‐off 40) and against CAII (D, TCs 2′454’675, AC 0.86, cut‐off 40). DEL selections were performed in duplicate (*n* = 2) (Figures S24 and S25, Supporting Information). The most enriched CAIX hits identified from FM‐DEL1 and FM‐DEL2 are indicated with arrows, along with the corresponding EFs. E) Chemical structures of hit compounds 18–22 and the acetazolamide (AAZ) derivatives 23–25. Affinity measurement of compounds 18–21 (F) and compounds 23 and 24 (G) by FP against CAIX. H) K_D_ values by FP of the compounds 21, 24, and fluorescent derivative of acetazolamide (AAZ*) against human CAIX (hCAIX), murine CAIX (mCAIX), and bovine CAII (bCAII). I) SPR analysis of compounds 22 and 25 (500 nM to 250 nM) binding to hCAIX immobilized on a CM5 chip (reaching 3′816 RUs). J) Flow cytometry analysis on SK‐RC‐52 cells incubated with Lys‐FITC (negative control), compound 21, or 24. Additional data in Figure S45 (Supporting Information). K) Time‐course biodistribution studies with ^177^Lu‐compound 22 (left) and ^177^Lu‐compound 25 in SK‐RC‐52 BALB/c nu/nu tumor‐bearing mice. Biodistribution studies were performed with female mice at a molar activity of 3.33 MBq/nmol (≈2 MBq/mouse). Data are presented as mean of %ID/g ± standard error of the mean (SEM) (*n* = 3 mice/group).

Aromatic and heteroaromatic sulfonamides are functional groups known to contribute to interactions of small molecules with carbonic anhydrases (CAs).^[^ ^32^, ^48^ ^]^ The development of highly selective ligands for CAIX is challenging because of the substantial homology of its active site with other CAs expressed across multiple healthy human tissues.^[^ ^32^, ^33^, ^49^, ^50^, ^51^, ^52^ ^]^ During the FM‐DELs construction, sulfonamide moieties were incorporated as building blocks A and B with the aim of enhancing the chances of retrieving hits against CAIX. FM ‐DEL1 and FM‐DEL2 were screened against CAIX and carbonic anhydrase II (CAII [**Figure** **5**A–D]. CAII was included in DEL screenings as a closely related anti‐target displaying high structural similarities of the catalytic domain (tertiary structures and presence of Zinc in both enzymatic pockets).^[^ ^53^, ^54^ ^]^ In the FM‐DEL1 screenings, the most enriched combination bearing the building block **A1199** coupled with 4‐*iodo*‐phenylalanine exhibited a selective enrichment for CAIX (EF_CAIX_ = 2′358; EF_CAII_ = 429) [Figure 5A,B]. A line indicating a selective enrichment of library members including building block **A1199** was also visible in FM‐DEL2 selections against CAIX [Figure 5C]. The same line is missing in FM‐DEL2 CAII fingerprints [Figure 5D]. Hit **A3204/B364**, a library member that did not include building block **A1199**, was identified as the most enriched and selective compound toward CAIX (EF_CAIX_ = 4′157; EF_CAII_ = 7) [Figure 5C–E].

Both stereoisomers of hit **A1199** (derived from FM‐DEL1) and of hit **A3204/B364** (derived from FM‐DEL2) were synthesized as FITC derivatives (off‐DNA synthesis of compound **18**–**21**) [Figure 5E]. Moreover, a hybrid compound presenting building block **A3204** conjugated to acetazolamide (**AAZ**), a nanomolar binder of CAIX, on the FM‐DEL scaffold was designed and synthesized. FITC derivatives of **A3204/AAZ** (**23** and **24**) were included in our study to further investigate whether the conjugation of **A3204** could enhance the selectivity of AAZ for CAIX [Figure 5E]. The fluoresceine derivatives **20** and **21** presented a higher affinity for human CAIX (hCAIX; K_D_ = 2.46 nM; K_D_ = 0.95 nM, respectively) compared to the compounds **18** and **19** (K_D_ > 1 µM; Kd = 1.09 µM, respectively) and a similar affinity with the corresponding **AAZ** derivatives **23** and **24** (K_D_ = 2.06 nM; K_D_ = 1.85 nM, respectively) by FP [Figure 5F,G]. Compounds **20**, **21**, **23**, and **24** present a higher affinity toward hCAIX compared to a fluorescein derivative of acetazolamide (AAZ*, K_D_ = 10 nM), suggesting that **A3204** enhances the binding affinity of AAZ against hCAIX [Figure 5G,H]. Molecular docking studies suggest that **A3204** forms hydrophobic contacts with non‐catalytic residues, contributing to its increased binding affinity to CAIX. [Figure S49, Supporting Information]. The stereochemistry appeared to exert minimal influence on binding affinity, although a slight preference for the *R*‐enantiomers was observed (i.e., compounds **21** and **24**). The selective binding properties of the best candidates **21** and **24** have been compared with AAZ* for a panel of CAs including hCAIX, murine (mCAIX), and bovine (bCAII) [Figure 5H]. Compound **21** was identified as the most selective binder for hCAIX and mCAIX (K_D_ = 8.73 nM) over bCAII (K_D_ = 150 nM), confirming the high selectivity observed for hit **A3204/B364** in FM‐DEL2 screenings [Figure 5H].

To generate radiopharmaceutical precursors, DEL‐derived compounds presenting the highest affinities (compounds **21** and **24**) were resynthesized as *R*‐DOTAGA conjugates (**22** and **25**) [Figure 5E]. SPR was employed to further assess the binding kinetics of compounds **22** and **25** [Figure 5I]. Both molecules displayed fast association rates (k_on_). Additionally, compound **25** exhibited a slower dissociation rate (k_off_) compared to compound **22**, suggesting an extended residence time within the CAIX binding pocket [Figure 5I].

To evaluate the ability of the compounds to bind to CAIX expressed on the cell surface, a flow cytometry assay was performed using SK‐RC‐52 renal cell carcinoma cells. Compounds **21** and **24** bound to the antigen expressed on the surface of cancer cells, as evidenced by a shift in fluorescein signal as compared to non‐stained cells and to cells stained with an untargeted FITC compound (Lys‐FITC) [Figure 5J].

Compounds **22** and **25** were labeled with ^177^Lu and their in vivo biodistribution was evaluated in mice subcutaneously xenografted with SK‐RC‐52 tumors. The compounds were injected intravenously at 30 nmol kg^−1^ dose (2 MBq/mouse). Biodistribution values collected at 1 h and at 6 h are shown in Figure 5K. At the 1 h time‐point post‐injection, ^177^Lu compound **22** accumulated to SK‐RC‐52 CAIX positive lesions (≈43%ID/g) with a clean profile in healthy organs, except for kidney (≈13%ID/g) and stomach (≈67%ID/g). The high stomach uptake of ^177^Lu compound **22** may be due to the expression of mCAIX in this organ and to the intrinsic murine cross‐reactivity already highlighted by FP results [Figure 5H]. As anticipated from k_off_ values measured by SPR [Figure 5I], a different biodistribution profile was obtained with ^177^Lu compound **25** that presents a higher tumor retention over 6 h (≈35% ID/g at 1 h and ≈43% ID/g at 6 h) with a higher uptake in healthy organs, likely due to off‐target binding to other CA isoforms.

### 2.4 DEL Screening and Hit‐Validation Against NKG2D, a Marker of NK Cells

**Figure 6:**
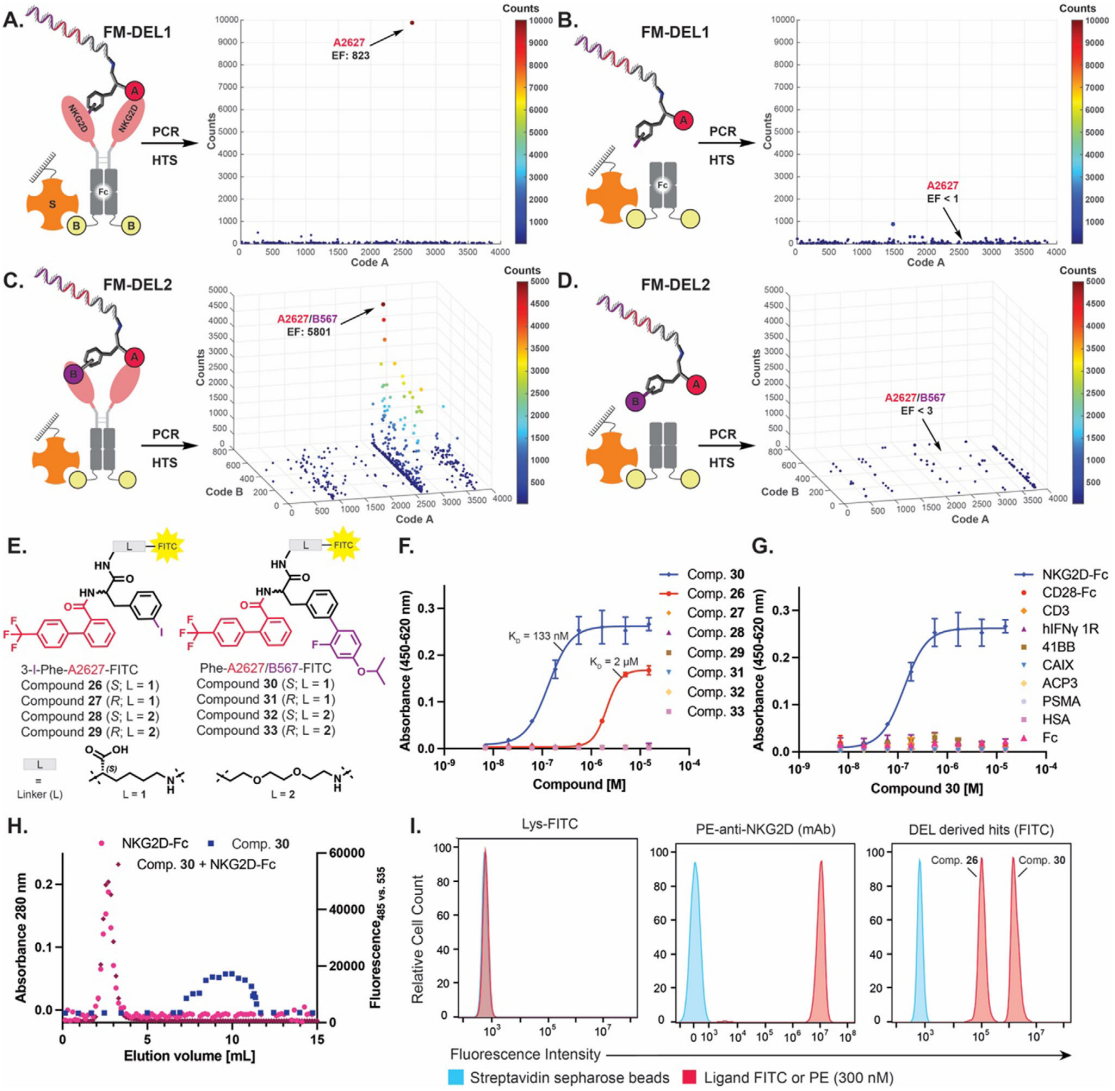
Screening of FM‐DELs and corresponding hit‐validation results against NKG2D‐Fc and hIgG‐Fc domain. FM‐DEL1 screening results (HTS) are represented as 2D‐plots after selections against NKG2D‐Fc (A, TCs 47′388, AC 12, cut‐off 20) and against hIgG‐Fc domain highlighted in the figure as Fc (B, TCs 46′872, AC 12, cut‐off 20). FM‐DEL2 screening results (HTS) are represented as 3D‐plots after selections against NKG2D‐Fc (C, TCs 2′431’707, AC 0.85, cut‐off 40) and against Fc (D, TCs 2′381’058, AC 0.84, cut‐off 25). DEL selections were performed in duplicate (*n* = 2) (Figures S26 and S27, Supporting Information). The most enriched NKG2D hits identified from FM‐DEL1 and FM‐DEL2 are indicated with arrows, along with the corresponding EFs. E) Chemical structures of hit compounds 26–33. F) Affinity measurement of compounds 26–33 by enzyme‐linked immunosorbent assay (ELISA) against NKG2D‐Fc. G) Affinity measurement of compound 30 by ELISA against multiple proteins. H) Binding of compound 30 to NKG2D‐Fc in a size‐exclusion chromatography co‐elution assay (Fc co‐elution shown in Figure S41, Supporting Information). I) Flow cytometry analysis on streptavidin sepharose beads incubated with Lys‐FITC (negative control), PE‐anti‐NKG2D (mAb, positive control), compound 26, or 30). Additional data can be found in Figure S46 (Supporting Information).

In the search for a small molecule that can bind to an immunological target, FM‐DEL1 and FM‐DEL2 were screened for interactions with NKG2D [**Figure** **6**A,C]. The NKG2D‐Fc fusion protein was used in these screenings, where the extracellular domain of NKG2D was fused with an IgG Fc fragment. This design enhances the stability of the NKG2D homodimer, a crucial factor for achieving high‐quality selection results and reliable hit validation. To rule out the possibility that any observed binding was due to non‐specific interactions with the Fc region of the recombinant protein, parallel selections with FM‐DEL1 and FM‐DEL2 were also performed against Fc alone [Figure 6B,D]. The two libraries were screened against human serum albumin (HSA) as an additional irrelevant protein (negative control) to better rule out nonspecific binding of library members [Figures S30 and S31, Supporting Information].

FM‐DEL1 screenings against NKG2D‐Fc showed a selective enrichment of building block **A2627** [4′‐(trifluoromethyl‐1,1′‐biphenyl)‐2‐carboxylic acid] coupled to the 3‐*iodo*‐phenylalanine scaffold, with an enrichment factor of 823 (EF_Fc_ < 1) [Figure 6A,B]. In the screening of FM‐DEL2, we observed that the 3‐I‐Phe‐**A2627** fragment played a crucial role in driving binding toward NKG2D, resulting in a distinct line of enriched hits [Figure 6C]. This selective enrichment was accompanied by a strong retention of specificity against the Fc portion of the recombinant protein (negative control selections) [Figure 6D]. Notably, adding the second building blocks enhanced the EFs obtained after selections, suggesting a synergistic effect on target interaction. Additionally, regioselectivity (i.e., a preference for a *meta* substitution on the aromatic ring of the phenylalanine scaffold), which already appeared in FM‐DEL1 selections, was retained in FM‐DEL2. **A2627/B567** is the most enriched and NKG2D selective hit from FM‐DEL2 (EF_NKG2D_ = 5′801; EF_Fc_ < 3). The selectivity of **A2627** and **A2627/B567** for NKG2D was further assessed by evaluating their EFs and Z‐scores across DEL selections against various proteins (i.e., tumor‐associated antigens and closely related proteins, together with serum proteins), as reported in Table S8 (Supporting Information).

We synthesized a series of FITC derivatives of hits **A2627** and **A2627/B567** and evaluated the impact of scaffold stereochemistry and of different linkers on binding affinity against recombinant NKG2D‐Fc (compounds **26**–**33**). FITC derivatives of both hits in their *R* or *S* configuration were synthesized using a hydrophobic lysine linker (L1) or a polar PEG linker (L2) [Figure 6E]. Compounds **26** and **30**, corresponding to the *S* series of hit **A2627** and **A2627/B567** linked to FITC with L1, bound to NKG2D‐Fc in an enzyme‐linked immunosorbent assay (ELISA) with dissociation constants of 2 and 133 nM, respectively [Figure 6F; Figure S42, Supporting Information]. Interestingly, all derivatives from the *R* series and based on the L2 linker did not bind to the target. Further specificity testing revealed that compound **30** exhibited high selectivity over various unrelated proteins, including TAAs and serological proteins [Figure 6G]. Compound **30** formed a kinetically stable complex with NKG2D‐Fc in a size exclusion chromatography co‐elution experiment [Figure 6H; Figure S41, Supporting Information].

To validate these findings, we performed a flow cytometry experiment using streptavidin sepharose beads. A significant shift in binding was observed for both the commercially available NKG2D monoclonal antibody (mAb, positive control) and compound **30**, while only moderate binding was detected for compound **26** [Figure 6I; Figure S46, Supporting Information]. These results were consistent with the fingerprint analysis, which indicated that adding a second building block significantly enhanced the binding affinity of the 3‐I‐Phe‐**A2627** fragment.

Compounds **26** and **30** were tested for their ability to induce IFN‐γ release by NK cells. An NKG2D functional assay was performed using human NK‐92 cells incubated with the two NKG2D ligands. The two compounds were not found to induce the release of IFN‐γ [Figure S39, Supporting Information], thus confirming that these DEL‐derived compounds are not functionally active but act as pure binders of NKG2D.

## 3 Discussion

In this study, we report the design, synthesis, and characterization of two DELs based on phenylalanine scaffolds for the identification of high‐affinity ligands targeting key proteins implicated in cancer therapy, with a particular focus on prostate cancer, kidney cancer, and immunological targets. Expanding upon our previous work with compact scaffold‐based DELs,^[^ ^7^, ^45^, ^55^ ^]^ we demonstrate how modifications to our earlier NF‐DEL platform can lead to larger and highly productive libraries, enabling the identification of promising binders with enhanced specificity and affinity. The screening of the single building block FM‐DEL1 gave complementary information about the role of certain fragments (building blocks A) in binding to pharmaceutically relevant targets, which was, in certain cases, improved by the addition of a second building block (B).

The synthesis of FM‐DELs involved DNA‐compatible reactions performed on the same scaffold functional groups (i.e., amine and iodide on I‐phenylalanine scaffold), which can be tested to improve yields and purity. This procedure has allowed us to build highly pure libraries, as demonstrated by quality controls on both FM‐DEL1 and FM‐DEL2 [Supporting Information and Figure 1]. This is an advantage when compared to different library designs based on linear sequences of chemical building blocks, where variable fragment reactivities can compromise coupling efficiency and overall fidelity.^[^ ^6^ ^]^ When compared to dual pharmacophore library formats (e.g., encoded self‐assembled chemical formats),^[^ ^56^, ^57^ ^]^ the use of a central scaffold enables straightforward hit validation, avoiding the need for linker optimization and functionalization procedures.^[^ ^30^ ^]^

The FM‐DEL platform represents an evolution of the earlier NF‐DEL design, with a broader structural diversity that facilitates the identification of high‐affinity ligands across a wider spectrum of targets. By optimizing reaction conditions for the incorporation of carboxylic acids, isocyanates, sulfonyl chlorides, amines, and isothiocyanates in position A,^[^ ^44^ ^]^ we generated 3′243 derivatives that were purified one by one by HPLC, with a substantial impact on the quality of FM‐DEL1 and FM‐DEL2. The enhanced productivity of FM‐DEL2 was further attributed to the introduction of 739 building blocks B by derivatization of the FM‐DEL1 iodo moiety. The effect of this expansion was particularly evident across all screening results, as the most enriched hits for each target protein exhibited novel building block combinations (all hit structures not included in our previous NF‐DEL).^[^ ^45^ ^]^ The comparison between FM‐DEL1 and FM‐DEL2 reveals the significant contributions of adding a second building block to the scaffold by combinatorial chemistry. The increased structural complexity in FM‐DEL2 led to a higher diversity of chemical functionalities, which directly translated into improved binding affinities and selectivity. The dual building block approach also allowed for a more refined exploration of the chemical space, resulting in ligands with superior specificity for their target proteins. This highlights the value of library diversification in improving hit discovery and optimizing binding properties. FM‐DEL2 exhibits a competitive hit rate when compared to previously reported DEL platforms based on both single and dual pharmacophore approaches (i.e., DELs based on linear formats and encoded‐self assembled chemical libraries).^[^ ^6^, ^57^, ^58^, ^59^ ^]^

The screening against prostate cancer targets was conducted using both FM‐DEL1 and FM‐DEL2 libraries. The focus was directed toward PSMA and ACP3, with the objective to identify ligands with adequate tumor‐targeting profiles, thereby mitigating off‐target effects often observed with PSMA‐targeting agents, such as renal toxicity and xerostomia.^[^ ^21^, ^47^ ^]^ From the PSMA screenings, **A3113/B423** emerged as the most enriched and selective combination for PSMA compared to GCP3. Compound **5** (*S*‐**A3113/B423**‐FITC) demonstrated selective binding to PSMA with a K_D_ of 13 nM as measured by FP. Further validation using flow cytometry confirmed its binding affinity to HT‐1080.hPSMA cells. The corresponding radiolabeled derivative, ^177^Lu‐compound **7** (*R*‐DOTAGA conjugated), was evaluated in autoradiography. This compound showed good tumor uptake in HT‐1080.hPSMA cells, as well as in patient‐derived xenografts (PDXs) J000077451 and TM00298, while exhibiting reduced uptake in healthy kidney tissue compared to Pluvicto. These findings suggest that the *S*‐**A3113/B423** combination is a promising candidate for further optimization to enhance binding affinity while maintaining its selectivity for prostate lesions. In the hACP3 screenings, phosphonate‐based ligands, such as **A2740/B736**, **A2855/B725**, and **A3146/B725**, emerged as the most enriched combinations, while no enrichment was observed in selections against double mutant hACP3 (H44A, H289A hACP3), suggesting high selectivity and binding in the active site of the protein. Among these combinations, the DOTAGA derivative of *S*‐**A2855/B725** exhibited potent inhibitory activity with a subnanomolar IC_50_ of 0.23 nM, as determined by enzyme inhibition assays with hACP3. The binding affinity of *S*‐**A2855/B725** for hACP3 was further confirmed through in vitro studies. Given the high and selective expression of ACP3 in prostate cancer lesions,^[^ ^30^ ^]^ the use of *S*‐**A2855/B725** as tumor‐targeting moiety may lead to the development of radioligand theranostics for PCa with low uptake in salivary glands and kidney.

Another tumor‐associated antigen explored with FM‐DELs in this study is CAIX. This is an attractive target for both imaging and targeted therapy due to its restricted expression in tumor tissues and its involvement in the adaptation to hypoxia in the tumor microenvironment.^[^ ^31^, ^32^ ^]^ Monoclonal antibodies, peptides, and DEL‐derived small organic ligands have been successfully used to target CAIX‐positive tumors.^[^ ^7^, ^50^, ^51^, ^52^, ^60^ ^]^ Despite the fact that favorable tumor accumulation has been observed in mice and cancer patients with diagnostic agents based on CAIX‐targeting ligands, their localization to normal organs such as kidneys and stomach prevents therapeutic applications.^[^ ^51^, ^52^ ^]^ The hits identified from our FM‐DEL platform against CAIX demonstrated promising selectivity and affinity, with the **A3204/B364** combination emerging as the most enriched for CAIX. The binding of *R*‐**A3204/B364** (compound **21**) to CAIX was highly selective, with minimal off‐target binding to CAII, a closely related protein (K_D _= 0.95 nM with hCAIX, K_D_ = 8.73 nM with mCAIX, and K_D_ = 150 nM with bCAII by FP). Introducing small molecule payloads, such as acetazolamide (**AAZ**), to the FM‐DEL scaffold showed enhanced binding to CAIX (AAZ* K_D_ = 10 nM, compound **23** K_D_ = 2.06 nM, and compound **24** K_D_ = 1.85 nM by FP). The ability of **A3204** to enhance the binding of **AAZ** to CAIX suggests that combining our DEL‐derived scaffolds with existing small molecules could lead to more potent and selective agents for CAIX‐targeted therapies. These findings pave the way for the development of CAIX‐targeted radiopharmaceuticals for patient stratification via PET imaging with radiolabeled analogs (e.g., [^68^Ga]‐compound **22**) and potential combination with hypoxia‐activated prodrugs.

In addition to targeting tumor‐associated antigens, we also explored the potential of FM‐DELs for developing ligands targeting immune system receptors, specifically NKG2D. NKG2D is expressed on the surface of NK cells and plays a crucial role in the immune recognition of tumor cells.^[^ ^41^ ^]^ Identifying small molecules that can bind and modulate NKG2D activity is a promising strategy to generate fully synthetic immunostimulatory agents,^[^ ^37^, ^38^ ^]^ which could present a superior penetration as compared to antibodies in solid tumors^[^ ^13^, ^15^ ^]^ and, therefore, better efficacy. The FM‐DEL screenings against NKG2D yielded several promising hits, particularly from FM‐DEL2, with **A2627/B567** emerging as the most enriched and selective ligand. The synthesis of Lys‐FITC derivatives and subsequent binding assays revealed that the compound **30** (*S*‐**A2627/B567**) could bind specifically to NKG2D with nanomolar affinity (K_D_ = 133 nM measured by ELISA). The compound forms a kinetically stable complex with NKG2D‐Fc, as confirmed by a size exclusion chromatography co‐elution experiment. The interaction with the receptor was further confirmed by a flow cytometry experiment using streptavidin sepharose beads. Our experiments are limited by the absence of cell‐based binding experiments. Compound **30** could serve as a payload for the development of fully synthetic bispecifics, in which tumor‐targeting efficiency would be ensured through conjugation to a small organic targeting moiety.

In summary, the FM‐DEL platform has proven to be an effective tool for discovering high‐affinity ligands targeting key cancer‐related proteins as well as immune targets. The use of *iodo*‐phenylalanine as a scaffold in FM‐DEL1 and FM‐DEL2 enabled orthogonal diversification, with both regio‐ (*ortho*, *meta*, *para*) and stereochemical (*S*/*R*) features significantly influencing affinity and specificity for the target. *Meta*‐substituted isomers frequently exhibited superior binding and selectivity, while the role of stereochemistry (i.e., *R* versus *S* scaffold configuration) varied across targets, being essential in some cases (i.e., PSMA, NKG2D) and negligible in others (i.e., ACP3, CAIX). The structural diversity of FM‐DEL1 and FM‐DEL2 allowed for the identification of selective and potent candidates, which hold great promise for both therapeutic and diagnostic applications. Moving forward, further optimization of these ligands, along with in vivo validation and clinical testing, will be crucial in advancing these compounds into the clinic and improving patient outcomes in cancer treatment.

## 4 Experimental Section

Details on general procedures, synthetic protocols, compound characterization, instrumentation, and additional results are provided in the Supporting Information.

### Library Construction

DNA‐encoded chemical libraries were synthesized using DNA‐recorded split and pool protocols. FM‐DEL1 synthesis was initiated by coupling stereo‐ and regioisomers of three *iodo*‐phenylalanine scaffolds to a 5′‐amino‐modified 14‐mer oligonucleotide (5′ NH_2_‐C_6_‐GGAGCTTCTGAATT). Scaffold‐bearing DNA was split in wells and derivatized, obtaining 2′645 amides, 329 sulfonyl amides, 189 ureas, 52 thioureas, 228 triazoles, and 412 “reverse” amides. DNA conjugates were recorded by the addition of a unique set of 3′855 codes A (5′ CTGTGTGCTGXXXXXXCGAGTCCCATGGCGC, XXXXXX = coding region) and pooled. The pool was HPLC purified and elongated with the obtainment of FM‐DEL1. Part of FM‐DEL1 was subsequently derivatized with 611 boronic acids and 128 alkynes (building blocks B) via Suzuki and Sonogashira cross‐coupling, respectively. After encoding each reaction with code B (5′ CGGATCGACGXXXXXXXGCGTCAGGCAGC, XXXXXXX = coding region), reaction mixtures were pooled and purified by HPLC to yield FM‐DEL2.

### DEL Screening Procedure

Target proteins were biotinylated and subsequently immobilized on magnetic Dynabeads MyOne Streptavidin C1. For each selection experiment, 5 × 10^9^ copies of FM‐DEL1 and 5 × 10^7^ of FM‐DEL2 were used (corresponding to 65 pmol/selection for the FM‐DEL1 and 500 pmol/selection for the FM‐DEL2). Prior to DEL selections, FM‐DEL1 and FM‐DEL2 were pre‐incubated with herring sperm to quench non‐specific DNA‐protein interactions. Automated DEL affinity selections were conducted using a KingFisher magnetic particle processor, following the methodology described in the literature.^[^ ^61^ ^]^ In brief, DELs were incubated with the protein‐coated beads of interest, allowing the interaction between library members and target protein. Non‐binding library members were removed by washing the beads with protein‐specific buffer added with Tween 20 (0.05% v/v). DNA‐compound conjugates that were specifically bound to the target protein were then eluted via heat denaturation (95 °C for 10 min). The eluted DNA conjugates were subjected to two‐step PCR amplification, followed by sequencing using Illumina high‐throughput technology (Novaseq, Functional Genomics Center Zurich). All selection experiments were performed in duplicates.

### Hit Nomination, Synthesis, and Validation

Selection results were decoded with C++^[^ ^61^ ^]^ and visualized using MATLAB R2019b (MathWorks). EFs were calculated by dividing the DNA sequence counts for each hit compound by the average DNA sequence count from the corresponding DEL screening. Additionally, Z‐score values were calculated following methods previously published,^[^ ^60^ ^]^ as present in Supporting Information. Hit compounds with high EFs in the target protein selections and low EFs in the negative controls (anti‐target and streptavidin selections) were selected for hit validation activities. All compounds were synthesized using either solid‐phase peptide synthesis or solution‐phase methodologies. Affinities of fluorescein‐bearing hits were determined by FP or ELISA measurements. Selective and kinetically stable interaction of fluorescent ligands with the target protein was demonstrated by co‐elution experiments based on size‐exclusion chromatography using PD midiTrap G‐25 columns. ACP3 colorimetric inhibition assay was performed with serially diluted compounds at a fixed concentration (5 mM in PBS) of *para*‐nitrophenyl phosphate as substrate. Binding kinetics were assessed on a Biacore X100 instrument with the target protein immobilized on a CM5 chip at 4′000 RUs. Cell binding of fluorescent ligands was evaluated using flow cytometry against antigen‐positive and antigen‐negative (wild‐type) cancer cells. in vitro tissue uptake of ^177^Lu‐labelled DOTAGA PSMA‐radioligands was measured via autoradiography. Prism 9 software (GraphPad Software) was used for data analysis of FP, ELISA, PD‐10, SPR, and enzymatic assay measurements. SPR sensograms were fitted with the Biacore X100 Evaluation Software. Flow cytometry data was processed with the FlowJo 10.4 software.

### In Vivo Biodistribution Studies

Animal experiments were performed in compliance with Swiss animal welfare laws, regulations, and ethical guidelines under the approved license number ZH06/2021 granted by the Veterinary Office of the Canton of Zurich. Tumor cells (SK‐RC‐52) were implanted subcutaneously in the flank of athymic BALB/c AnNRj‐Foxn1 nude mice. Tumor size was measured regularly with an electronic caliper. Tumor volume (in mm^3^) was calculated using the formula: (long side, mm) × (short side, mm) x (short side, mm) × 0.5. Mice were sacrificed when one or more termination criteria indicated by the experimental license was reached (e.g., weight loss >15% or tumor diameter >15 mm). For in vivo biodistribution analysis, test compounds were administered intravenously at 30 nmol kg^−1^ dose (2 MBq/mouse), and animals were sacrificed at different time points. Organs and tumors were excised, and radioactivity was quantified using a Packard Cobra gamma counter. Prism 9 software (GraphPad Software) was used for data analysis.

## Conflict of Interest

D.N. is co‐founder, CEO, CSO, and President of the Scientific Advisory Board of Philogen. F.M., A.C., S.D.P., T.G., I.B., N.F., E.P., S.O., and S.C. are employed by Philochem AG, the research and development unit of the Philogen group. F.M., T.G., S.C., S.O., and D.N. declare to be co‐inventors in patent application PCT/EP2024/080350 (describing ACP3 ligands) filed on October 25^th^, 2024. All other authors do not declare any conflict of interest.

## Author Contributions

All authors have contributed to the preparation of this manuscript. F.M., S.C., and D.N. designed the experiments. F.M., N.F., S.C., and D.N. designed FM‐DEL1 and FM‐DEL2. F.M. synthesized the chemical library. F.M., A.C., and N.F. analyzed the high‐throughput screening data. F.M., A.C., and M.M. performed hits resynthesis and validation. S.D.P. designed, produced, and biotinylated NKG2D‐Fc and Fc. I.B. designed, produced, and biotinylated ACP3 variants. F.M. and S.O. biotinylated CAIX, PSMA, TNAP, and GCP3. F.M., A.C. S.D.P., and S.O. performed in vitro experiments. F.M., T.G., S.O., and S.C. performed in vivo experiments in tumor‐bearing mice. E.S. and G.V. performed molecular docking studies.

## Supporting information



Supporting Information

## Data Availability

The data that support the findings of this study are available from the corresponding author upon reasonable request.
