# Supplementary material for: Phenylalanine‐Based DNA‐Encoded Chemical Libraries for the Discovery of Potent and Selective Small Organic Ligands Against Markers of Cancer and Immune Cells
Source: Adv Sci (Weinh). 2025 Jun 29;12(35):e05351. doi: 10.1002/advs.202505351 (PMC12463120; doi:10.1002/advs.202505351)
Supplement: Supplementary file 1 — Supporting Information [file ADVS-12-e05351-s001.pdf]

ADVANCED SCIENCE
Open Access

Supporting Information

for *Adv. Sci.*, DOI 10.1002/advs.202505351

Phenylalanine-Based DNA-Encoded Chemical Libraries for the Discovery of Potent and Selective Small Organic Ligands Against Markers of Cancer and Immune Cells

Francesca Migliorini, Andrea Ciamarone, Sheila Dakhel Plaza, Tony Georgiev, Marta Mascellani, Emanuela Sabato, Giulio Vistoli, Ilaria Biancofiore, Nicholas Favalli, Emanuele Puca, Sebastian Oehler, Dario Neri and Samuele Cazzamalli**

SUPPLEMENTARY INFORMATION

Phenylalanine-Based DNA-Encoded Chemical Libraries for the Discovery of Potent and Selective Small Organic Ligands Against Markers of Cancer and Immune Cells

Francesca Migliorini[1], Andrea Ciamarone[1,2,3], Sheila Dakhel Plaza[1], Tony Georgiev[1], Marta Mascellani[1,4], Emanuela Sabato[4], Giulio Vistoli[4], Ilaria Biancofiore[1], Nicholas Favalli[1], Emanuele Puca[1,6], Sebastian Oehler[1], Dario Neri[1,5,6†], Samuele Cazzamalli[1†]

[1] Philochem AG, CH-8112 Otelfingen, Switzerland.

[2] University of Bologna, Department of Pharmacy and Biotechnology, I-40126 Bologna, Italy

[3] Computational and Chemical Biology, Istituto Italiano di Tecnologia, I-16163, Genoa, Italy

[4] University of Milano, Department of Pharmaceutical Sciences, I-20133 Milano, Italy.

[5] Department of Chemistry and Applied Biosciences, Swiss Federal Institute of Technology (ETH Zurich), CH-8093 Zurich, Switzerland.

[6] Philogen SPA, I-53100 Siena, Italy.

†Corresponding authors:

Prof. Dr. Dario Neri – Philochem AG, CH-8112 Otelfingen, Switzerland, +41 (0)43 544 88 06 - dario.neri@philogen.com

Dr. Samuele Cazzamalli – Philochem AG, CH-8112 Otelfingen, Switzerland, +41 (0)43 544 88 19 - samuele.cazzamalli@philochem.ch

Table of Contents

1 ABBREVIATIONS 4

2 MATERIAL AND GENERAL METHODS 7

2.1 REAGENTS AND SUPPLIES 7
2.2 SOFTWARE 7
2.3 STATISTICAL ANALYSIS 7
2.4 MOLECULAR DOCKING METHODS 7
2.5 PURIFICATIONS AND ANALYTICAL METHODS FOR OLIGONUCLEOTIDES 8
2.6 PURIFICATIONS AND ANALYTICAL METHODS FOR SMALL MOLECULES 9
2.7 NUCLEAR MAGNETIC RESONANCE (NMR) CHARACTERIZATION 9
2.8 PROTEIN PRODUCTION AND PURIFICATION 9
2.9 SODIUM DODECYL SULFATE-POLYACRYLAMIDE GEL ELECTROPHORESIS (SDS-PAGE) 10
2.10 BIOTINYLATION METHODOLOGIES 10
2.11 FLUORESCENCE POLARIZATION (FP) MEASUREMENTS WITH FLUORESCEINE-LABELLED COMPOUNDS 11
2.12 SIZE-EXCLUSION CHROMATOGRAPHY CO-ELUTION EXPERIMENTS 11
2.13 ACP3 COLORIMETRIC ENZYMATIC ASSAY 12
2.14 SURFACE PLASMON RESONANCE (SPR) 12
2.15 ENZYME-LINKED IMMUNOSORBENT ASSAY (ELISA) WITH SMALL MOLECULES 12
2.16 CELL CULTURES 13
2.17 FLOW CYTOMETRY EXPERIMENTS ON CELLS 13
2.18 FLOW CYTOMETRY EXPERIMENTS ON STREPTAVIDIN BEADS 13
2.19 IN VITRO INTERFERON-GAMMA RELEASE ASSAY 14
2.20 AUTORADIOGRAPHY EXPERIMENTS 14
2.21 QUANTITATIVE IN VIVO BIODISTRIBUTION STUDIES 14

3 DEL SYNTHESIS, PURIFICATION, AND CHARACTERIZATION 16

3.1 ETHANOL PRECIPITATION 16
3.2 SYNTHESIS OF FM-DELS 16
3.3 ENCODING DESIGN FOR FM-DELS 27
3.4 SOLID-PHASE AFFINITY SELECTIONS: PCR AMPLIFICATION AND SEQUENCING 27

4 PROTEINS USED FOR SELECTIONS: DESIGN, PRODUCTION, AND QUALITY CONTROL28

4.1 PROSTATE-SPECIFIC MEMBRANE ANTIGEN (PSMA) 28
4.2 GLUTAMATE CARBOXYPEPTIDASE III (GCP3) 29
4.3 HUMAN PROSTATIC ACID PHOSPHATASE (HACP3) 30
4.4 MURINE PROSTATIC ACID PHOSPHATASE (MACP3) 31
4.5 H44A, H289A HUMAN PROSTATIC ACID PHOSPHATASE MUTANT (H44A, H289A HACP3) 32
4.6 TISSUE NON-SPECIFIC ALKALINE PHOSPHATASE (TNAP) 34
4.7 CARBONIC ANHYDRASE IX (CAIX) 35
4.8 CARBONIC ANHYDRASE II (CAII) 36
4.9 NATURAL KILLER GROUP 2D FC FUSION (NKG2D-FC) 36
4.10 FRAGMENT CRYSTALLIZABLE (FC) OF HUMAN IGG1 38

5 COMPOUND SYNTHESIS 40

5.1 ON-DNA ACP3 HIT RE-SYNTHESIS 40
5.2 GENERAL SYNTHETIC PROCEDURES FOR SMALL MOLECULES 45
5.3 CHEMICAL SYNTHESIS OF HIT COMPOUNDS, AND INTERMEDIATES 47

6 SUPPLEMENTARY RESULTS 79

6.1 DEL-SELECTION FINGERPRINTS 79
6.2 Z-SCORE ANALYSIS OF DEL SELECTIONS 91
6.3 SELECTIVITY OF HITS A2627 AND A2627/B567 FOR NKG2D IN DEL SCREENINGS 100
6.4 INTERFERON-GAMMA RELEASE ASSAY ON HUMAN NK-92 CELLS 102
6.5 ADDITIONAL CO-ELUTION EXPERIMENTS BASED ON SIZE EXCLUSION CHROMATOGRAPHY 103

6.6 ELISA PLATES (PICTURES) ... 104
6.7 FLOW CYTOMETRY ASSAY - ADDITIONAL DATA ... 105
6.8 MOLECULAR DOCKING OF PSMA AND CAIX HIT COMPOUNDS ... 108

7 HRMS AND ^{1}H NMR SPECTRA OF COMPOUNDS ... 110

8 REFERENCES ... 173

1 Abbreviations

41BB: cluster of differentiation 137

AA: antibiotic-antimycotic

AAZ: acetazolamide

AC: average count

ACP3: prostatic acid phosphatase

Ad2: adaptor 2

Boc: di-*tert*-butyl decarbonate

CAII: carbonic anhydrase II

CAIX: carbonic anhydrase IX

CD28-Fc: cluster of differentiation 28 - fragment crystallizable

CD3: cluster of differentiation 3

DCM: dichloromethane

DEL: DNA-encoded library

DIPEA: *N*,*N'*-diisopropylethylamine

DMEM: Dulbecco's modified eagle medium

DMF: *N*,*N'*-dimethylformamide

DMSO: dimethyl sulfoxide

DNA: deoxyribonucleic acid

DOTAGA: 2-(4,7,10-tris(carboxymethyl)-1,4,7,10-tetraazacyclododecan-1-yl)pentanedioic acid

DTT: dithiothreitol

EDC: 1-ethyl-3- (3-dimethylaminopropyl) carbodiimide

EDTA: ethylenediaminetetraacetic acid

EF: enrichment factor

ELISA: enzyme-linked immunosorbent assay

equiv.: equivalent(s)

ESI: electrospray ionization

EtOH: ethanol

FA: formic acid

Fc: fragment crystallizable

FCS: fetal calf serum

FITC: 3',6'-dihydroxy-6-isothiocyanatospiro[2-benzofuran-3,9'-xanthene]-1-one

Fmoc: 9-fluorenylmethyloxycarbonyl

FP: fluorescence polarization

GCP3: GlutamateCarboxyPeptidase III

GP: general procedure

H44A, H289A hACP3: double mutant hACP3 (H44A, H289A)

hACP3: human prostatic acid phosphatase

HATU: *O*-(7-azabenzotriazol-1-yl)-*N,N,N',N'*-tetramethyluronium hexafluorophosphate

HEPES: 2-[4-(2-hydroxyethyl)piperazin-1-yl]ethanesulfonic acid

HFIP: 1, 1, 1, 3, 3, 3-hexafluoro-2-propanol

hIFNγ 1R: gamma interferon receptor 1

HPLC: high-performance liquid chromatography

HRMS: high resolution-mass spectrometry

HSA: human serum albumin

HTS: high-throughput DNA sequencing

IC_{50}: 50% inhibitory concentration

IFN-γ: interferon-gamma

IgG : immunoglobulin G

K_D: dissociation constant

K_{off} : off-rate constant

K_{on} : on-rate constant

L1 : linker 1

L2 : linker 2

LC: liquid chromatography

LC-MS: liquid chromatography-mass spectrometry

mAb: monoclonal antibody

mACP3: murine prostatic acid phosphatase

MBq: mega becquerel

min: minute(s)

mQ water: Milli-Q® water

MS: mass spectrometry

m/z: mass-over-charge ratio

n.c.a.: non carrier added

NHS: *N*-hydroxysuccinimide

NKG2D: Natural Killer group 2D

NKG2D-Fc: Natural Killer group 2D - fragment crystallizable

NMR: nuclear magnetic resonance

OCT: optimal cutting temperature

PBS: phosphate buffered saline

PCR: polymerase chain reaction

PDA: photodiode array

PE: phycoerythrin

PEG: polyethylene glycol

*p*NPP: *para*-nitrophenyl phosphate

PSMA: prostate-specific membrane antigen

RP: reverse phase

rt: room temperature

RUs: response units

SDS-PAGE: sodium dodecyl sulfate-polyacrylamide gel electrophoresis

SEC: size-exclusion chromatography

S-NHS: *N*-hydroxysulfosuccinimide sodium salt

SP: solid-phase procedure

TAAs: tumor-associated antigens

TBE: Tris/Borate/EDTA

$TBTAX_3$: 4,4',4''-(((nitrilotris(methylene))tris(1*H*-1,2,3-triazole-4,1- diyl))tris(methylene))tribenzoic acid

TCs: total counts

TEAA: triethylammonium acetate

TEA: triethylamine

TFA: trifluoroacetic acid

THF: tetrahydrofuran

TIPS: triisopropylsilane

TNAP: tissue-nonspecific alkaline phosphatase

TOF: time-of-flight

TPPTS: 3,3′,3′′-phosphanetriyltris(benzenesulfonic acid) trisodium salt

Tris-HCl: tris(hydroxylmethyl)aminomethane hydrochloride

UPLC: ultra-performance liquid chromatography

UV: ultraviolet

XPhos: dicyclohexyl(2',4',6'-triisopropyl-[1,1'-biphenyl]-2-yl)phosphane

2 Material and general methods

2.1 Reagents and supplies

Solvents and chemicals were used as purchased from ABCR GmbH, Sigma-Aldrich, Merck, Fluorochem, CombiBlocks, VWR, Apollo scientific, Enamine, TCI, Acros, Chematech, and Alfa Aesar. Milli-Q® water (mQ water) was obtained from a Millipore Milli-Q system (Merck). Oligonucleotides were purchased from LGC Biosearch Technologies or Eurofins and quantified with a Nanodrop 2000 Spectrophotometer (Thermo Fisher Scientific). Materials for ligations and polymerase-chain reactions were purchased from New England Biolabs. PCR purification and gel extraction kits were purchased from Qiagen. PD Midi Trap G-25 columns were provided by Cytiva. Lutetium-177 (n.c.a.) was provided by Isotopia or, alternatively, by ITM.

2.2 Software

Databases were organized with DataWarrior (OpenMolecules). Fingerprints have been visualized and evaluated with MATLAB R2019b (The MathWorks, Inc.). Data fitting and statistical analysis were performed with GraphPad Prism (version 9, Graphpad Software, Inc.). Flow cytometry data was analyzed using FlowJo (version 10.4, Tree Star). SPR sensograms were fitted with the Biacore X100 Evaluation Software. HPLC and MS data were analyzed with MassLynx (Waters) and ChemStation (Agilent).

2.3 Statistical analysis

Data reported in this article have been generated as independent replicates. Values deriving from replicate measurements are given as mean ± standard error of the mean (SEM) or standard deviation (SD), as indicated in the figure legends. Error bars have been calculated based on the standard deviation calculated on three or more replicates.

2.4 Molecular docking methods

Ligand structures were generated by using VEGA ZZ suite of programs[1], considering the ionized forms at physiological pH based on the pKa values as predicted by Marvin software v22.19.0. After a preliminary minimization, the structures underwent a Monte Carlo conformational search study by using AMMP software, and the resulting lowest energy conformation was further optimized by a semiempirical PM7-based structural refinement (using MOPAC2016 software)[2].

Molecular docking simulations were carried out by considering the resolved structure of CAIX in complex with the inhibitor acetazolamide (PDB id: 3IAI)[3] and PSMA in complex with the inhibitor PSMA-617 (PDB id: 8BOW)[4]. The protein structures were prepared and optimized by using the VEGA ZZ suite

of programs. The water molecules were removed (except for PSMA structure, where the coordinating water molecule was maintained), missing atoms were added, and residues were ionized by considering the physiological pH of 7.4. The proteins were then refined by two energy minimization procedures, each one of 10,000 steps using NAMD[5]: firstly, only the hydrogen atoms were minimized and, then, the entire protein structure was minimized by keeping fixed the backbone atoms to maintain the experimental folding. Finally, the bound ligand was removed from the optimized protein structure to perform following docking studies on investigated compounds.

Molecular docking studies were carried out by using PLANTS v1.2 software[6]. The protein was simulated rigid, while the ligands were considered as flexible, focusing the search within a 10 Å radius sphere around the co-crystallized ligand and generating 10 poses for each ligand, setting ChemPLP as scoring function and the search equal to 1 (highest accuracy). The docking simulations on PSMA involved pose constrains focused on the common ureido and glutamic moieties of the co-crystallized ligand.

2.5 Purifications and analytical methods for oligonucleotides

2.5.1 Reversed-phase high-performance liquid chromatography (RP-HPLC)

Preparative reversed-phase high-performance liquid chromatography (RP-HPLC) for the purification of oligonucleotides was performed with Agilent 1200 Series as well as a Waters Alliance HT coupled with a PDA UV detector equipped with an XTerra Shield RP18 preparative column (125 Å, 10 x 150 mm). Eluent A: 100 mM triethylammonium acetate (TEAA) pH 7 in mQ water. Eluent B: 100 mM TEAA pH 7 in 80% acetonitrile. Flow rate of 4 mL/min (gradient will be described together with library synthesis procedures). Product-containing fractions were combined, dried under reduced pressure in a SpeedVac vacuum concentrator (RVC 2-25CDplus, Martin Christ), and re-dissolved for ethanol (EtOH) precipitation (see below). Specific HPLC methods for the purification of oligonucleotide conjugates and their corresponding pools are indicated below in the relevant sections.

2.5.2 Analytical procedures for oligonucleotide conjugates

Characterization of oligonucleotide conjugates and DELs was performed by LC-MS using an Agilent 1260 Series LC equipped with an ACQUITY UPLC Oligonucleotide BEH C18 column (130 Å, 1.7 µm, 2.1 x 50 mm) coupled to an Agilent 6100 Series Single Quadrupole MS. Mobile phase was based on two eluents, eluent A (15 mM TEA, 400 mM HFIP in mQ water) and eluent B (methanol). Flow rate was kept at 0.4 mL/min. Column wad heated at 60 °C. Gradient (**Method A**): 0-0.2 min 95% A, 0.2-8.2 min 95% to 5% A, 8.2-8.7 min 5% A, 8.7-9.2 min 5 to 95% A, 9.2-12 min 95% A.

2.6 Purifications and analytical methods for small molecules

2.6.1 Reversed-phase high-performance liquid chromatography (RP-HPLC)

Method B: Small organic molecules that could be produced at higher quantities (>10 mg) were purified on a Thermo Fisher UltiMate 3000 HPLC chromatographer with a PDA UV detector. The system was equipped with an Hypersil Prep HS phenyl column 5 μm, 150 x 30 mm, using a flow rate of 30 mL/min with the following gradient: eluent A (mQ water 0.1% TFA) and eluent B (acetonitrile with 0.1% TFA), 0 – 1 min 80% A, 1 – 20 min 80 to 0% A, 20-22 min 0% A. The flow rate was set to 30 mL/min. Product-containing fractions were combined and lyophilized.

Method C: Compounds (small molecules) that could be produced at lower quantities (<10 mg) were purified on an Agilent 1200 Series HPLC chromatographer with a PDA UV detector. The system was equipped with a Synergi 4 µm, Polar-RP 80 Å 10 × 150 mm C18 column using flow rate of 5 mL/min with the following gradient: eluent A (mQ water 0.1% TFA) and eluent B (acetonitrile with 0.1% TFA): 0-20 min 90% to 0% A, 20-22 min 0% A. Product-containing fractions were combined and lyophilized.

2.6.2 Analytical procedures for small molecules

Small molecules were analyzed by HPLC-MS on an Agilent 1200 Series LC combined with an Agilent 6100 Series Single Quadrupole MS system. The mobile phase was based on two eluents, mQ water with 0.1% formic acid (eluent A) and acetonitrile with 0.1% formic acid (eluent B). Flow rate was set at 0.5 mL/min. A ZORBAX SB-C18 column (2.1 × 50 mm, 1.8 µm particle size, 120 Å pore size), heated at 40 °C, was used.

Method D: 10% to 100% B in 3 min.

Method E: 40% to 100% B in 3 min.

2.7 Nuclear magnetic resonance (NMR) characterization

Proton (^{1}H) and carbon (^{13}C) nuclear magnetic resonance (NMR) spectra were measured at 298 K on a Bruker AV400 (400 MHz) spectrometer. Chemical shifts are reported in parts per million (ppm) with deuterated solvents as reference signal. The coupling constants (*J*) are given in Hz using the following multiplicity abbreviations: s = singlet, d = doublet, t = triplet, q = quartet, dd = doublet of doublets, dt = doublet of triplets, td = triplet of doublets, m = multiplet.

2.8 Protein production and purification

From a glycerol stock (20% glycerol, stored at -80 °C) a small amount of frozen transformed TG-1 E. coli was cultivated in LB medium, supplemented with 100 µg/mL of ampicillin, overnight (37 °C, 180 rpm) using an SSI3 shaking incubator (Shel Lab, Cornelius, USA). After incubation, the bacteria were

harvested, and plasmid DNA (pDNA) was purified using the NucleoBond® XtraMaxi kit (MACHEREY-NAGEL GmbH, Duren, Germany).
CHO cells were seeded at a density of 3 × 10^6 cells/mL in PowerCHO®-2 CD medium (Lonza Group, Basel, Switzerland), supplemented with 1% Anti-Anti (Thermo Fisher Scientific Inc., Waltham, USA) and 2% Ultraglutamine 1 (Lonza Group, Basel, Switzerland). The cells were cultivated overnight (37 °C, 5% CO_2, 120 rpm) using a Climo-Shaker ISF1-XC (Adolf Kuhner AG, Birsfelden, Switzerland). The following day, the cells were counted in a Neubauer chamber and resuspended at a density of 5 × 10^6 cells/ml in 500 mL of ProCHO® medium (Lonza Group, Basel, Switzerland), supplemented with 1% Anti-Anti and 2% Ultraglutamine 1. The pDNA and Poly(ethyleneimine) (PEI) were added at a concentration of 0.75 µg/10^6 cells and 2.5 µg/10^6 cells respectively. The transfected cells were cultured for 6 days (31 °C, 5% CO_2, 120 rpm) using a Climo-Shaker ISF1-XC.
The mammalian cells were centrifuged (4900 rpm, 4 °C, 30 min) and the supernatant was filtered using 0.2 µm NALGENE™ Rapid-Flow™ vacuum filters (Thermo Fisher Scientific Inc., Waltham, USA). The produced protein was purified from the supernatant using Protein A Resin (IBA Lifesciences, Göttingen, Deutschland) or cOmplete His-Tag Purification Resin (Roche, 08778850001) in a 30 x 130 mm SIGMA liquid chromatography column (Merck KGaA, Darmstadt, Germany). The protein was dialysed under gentle stirring at 4 °C for 16 hours with the corresponding buffer (1:100 ratio). After dialysis, the protein was further purified using size-exclusion chromatography (SEC). For SEC, the GE AKTA Explorer (GE Healthcare, Chicago, USA) FPLC system was used together with a Superdex™ 200 Increase 10/300 GL or Superdex™ 75 Increase 10/300 GL according to the molecular weight of the protein.

2.9 Sodium dodecyl sulfate-polyacrylamide gel electrophoresis (SDS-PAGE)

Precast SurePAGE Bis-Tris 10% gels (Invitrogen, NP0302BOX) were used with MOPS running buffer following the supplier's procedure (45 min run time at 180 V and 100 mA). Proteins were mixed with Laemmli buffer and loaded together with Precision Plus Protein™ All Blue Prestained Protein Standards (4 µL, Bio-Rad, #1610373) as a size reference. Proteins were visualized by Coomassie staining. Band shift assays were performed by loading biotinylated protein (3 µg) and a mixture of avidin with the biotinylated protein (3 µg + 3 µg) to visualize complex formation as band shift.

2.10 Biotinylation methodologies

Site-specific biotinylation (Avi-tag)

The protein was site-specific biotinylated using BirA. Protein buffer was exchanged to BirA buffer (100 mM Tris pH 7.5, 200 mM NaCl, 5 mM $MgCl_2$) using a PD-10 column (Cytiva #17085101). The protein (5 mg, in 6 mL BirA buffer, 0.9 mg/mL), was mixed with 350 µg MBPBirA, 625 µL of 500 mM ATP in 1 M Tris HCl pH 9.5, 500 µL of 20 mM biotin in DMSO and half a tablet of protease inhibitor cocktail (cOmplete TM Protease Inhibitor Cocktail, Roche) previously dissolved in 750 µL of mQ water. The enzymatic reaction was gently shaken in a Hula Mixer for 16 hours, in the dark at room temperature.

The protein was then concentrated using Vivaspin® 20 centrifugal concentrators (30'000 Da molecular weight cutoff, Sartorius, #VS2012) and then purified by SEC or PD-10 column. The fractions were measured (absorption at 280 nm) on a Nanodrop 2000 Spectrophotometer to combine protein-containing fractions. Proteins were directly used for selections or stored at - 80 °C until use.

Chemical biotinylation

Protein dissolved in PBS or HEPES buffer was incubated for 30-60 min with 5 equiv. of EZ-LinkTM NHS-LC-Biotin (Thermo Fisher Scientific, #21336). The reaction was quenched by addition of 100 equiv. 500 mM Tris-HCl buffer pH 9.4. The resulting mixture was purified with a pre-equilibrated PD-10 column (Cytiva, #17085101). The fractions were measured (absorption at 280 nm) on a Nanodrop 2000 Spectrophotometer to combine protein-containing fractions. Proteins were directly used for selections or stored at - 80 °C until use.

2.11 Fluorescence polarization (FP) measurements with fluoresceine-labelled compounds

A dilution series of target proteins (i.e., PSMA, GCP3, CAIX, CAII) in the respective formulation buffer were separately prepared in a black 384-well plate (non-binding Greiner small volume plate). Test compounds functionalized with fluorescein were diluted to a concentration between 5 and 10 nM (5 µL) and incubated for 15 min with the protein (final volume corresponding to 10 μL). The fluorophore was excited at 485 nm, and anisotropy was measured at 535 nm on a Spectra Max Paradigm multimode plate reader (Tecan). The experiments were performed in triplicate. K_D values measured using this FP method can differ from dissociation constants obtained by other physical methods (e.g., surface plasmon resonance, see section below). This observation is not uncommon, and it has been reported by our research group and by others[7,8].

2.12 Size-exclusion chromatography co-elution experiments

Test compound (0.3 nmol, stock solution at 5 mM in protein buffer + 5% DMSO) was incubated with 2.7 nmol of target protein in its corresponding buffer (3 μM, ~900 μL final volume) for 5 minutes at room temperature. The ligand-protein solution was then loaded onto a pre-equilibrated PD midiTrap G-25 column (Cytiva, #28918008) and eluted with the respective protein buffer (gravimetric flow). Fractions (100 µL each) were collected, and absorbance was measured to detect protein concentration (λ = 280 nm on a Nanodrop 2000 Spectrophotometer). Fluorescence of each fraction was separately measured on a Tecan Spark® Multimode Microplate Reader (λ Excitation = 485 ± 20 nm, λ Emission = 535 ± 25 nm).

2.13 ACP3 colorimetric enzymatic assay

Grenier 384 transparent, flat-bottom well plates were used. Each test compound was measured in triplicate. 16 μL of a solution of the compound (3μM, 5% DMSO in PBS for small molecules or PBS only for DNA-conjugates, pH 7.4) was loaded into the first well row. A serial 1:2 (on DNA) or 1:3 (off DNA) dilution in DMSO/PBS (5/95, off DNA) or only PBS (on DNA) was performed in a total volume per well of 8 μL. Human recombinant ACP3 (16 μL solution, 2.5 nM in PBS, pH 7.4) was added to each well. After 10 min of incubation, 16 μL of a solution of *para*-nitrophenyl phosphate (pNPP, substrate, 5 mM in PBS, pH 7.4) was added. The plate was left to develop in the dark for 16 hours. Enzymatic reaction on the substrate (phosphate group removal by ACP3) was assessed by measuring absorption at 405 vs 620 nm on a Spectra Max Paradigm multimode plate reader (Tecan).

2.14 Surface plasmon resonance (SPR)

SPR measurements were performed on a Biacore X100. Human recombinant ACP3 or CAIX were immobilized on a CM5 chip (Cytiva, #BR100012, both flow cells), reaching ~4000 RUs, using the EDC/NHS protocol provided by the manufacturer. The target protein immobilized on the reference flow cell was treated with denaturing solution (0.85% H_3PO_4, 10 mM NaOH, and 50 mM HCl). PBS (pH 7.4) was used as running buffer. Test compounds were injected at different concentrations in running buffer, according to a multicycle analysis with the following settings: 120 seconds contact time and 5´000 seconds (CAIX measurements) or 15´000 seconds (ACP3 measurements) dissociation time at a flow rate of 10 µL/min. Sensograms were plotted with GraphPad Prism (version 9, GraphPad Software) and fitted using the Biacore X100 Evaluation Software.

2.15 Enzyme-linked immunosorbent assay (ELISA) with small molecules

The protein (100 µL/well, 170 nM) was incubated for 2 h at 4 °C on a StreptaWell plate (transparent 96-well plate, Roche #11645692001). The protein was blocked by adding 4% Milk in PBS (200 µL/well) for 30 min at rt and washed with PBS (3 x 200 µL/well). The immobilized protein was incubated for 30 min in the dark with serial dilution of FITC-labelled compounds and washed with PBS (3 x 200 µL/well). A solution of rabbit polyclonal FITC antibody-horseradish peroxidase (anti-FITC-HRP, Abcam, #ab196968) 0.25 µg/mL in 2% milk-PBS was added to each well (100 µL/well) and incubated for additional 30 min in the dark at rt. Each well was washed with PBS 0.1% Tween (3 x 200 µL/ well) and with PBS (3 x 200 µL/ well). The substrate 3,3′,5,5′-tetramethylbenzidine (TMB, Sigma Aldrich #T0440) was added (100 µL /well) and developed in the dark for 0.5-1 min. The reaction was stopped by adding 50 µL of an aqueous solution of H_2SO_4 1M. The absorbance was measured at 450 nm (reference level, 620–650 nm) with a Spark multimode microplate reader (Tecan Life Sciences). The experiments were performed in triplicate.

2.16 Cell cultures

HT1080.hPSMA and PC3.hACP3 cell lines were generated by lentiviral transduction on wild type cells (HT1080.wt and PC3.wt), according to published procedures[9]. For long-term storage, cell lines were maintained in liquid nitrogen. Prior to *in vitro* and *in vivo* experiments, cells were cultured in DMEM (PC3.wt, PC3.hACP3, HT1080.wt, and HT1080.hPSMA) or RPMI medium (SK-RC-52) (medium provided by Invitrogen) supplemented with FCS (10%, Invitrogen) and antibiotic-antimycotic (1%, AA, Invitrogen) at 37 °C and 5% CO_2. For passaging, cells were detached using Trypsin-EDTA 0.05% (Invitrogen) when reaching 90% confluency and re-seeded at a dilution of 1:3 (PC3.hACP3 and PC3.wt) or 1:4 (HT1080.hPSMA, HT1080.wt and SK-RC-52).

2.17 Flow cytometry experiments on cells

Cancer cells were detached with an Accutase® cell detachment solution, resuspended in a standard culture medium, and transferred to falcon tubes. Cells were counted and centrifuged at 400 rcf for 3 min. The supernatant was removed, and cell pellets were resuspended in FACS buffer (PBS 1x, 2 mM EDTA) at a density of ~300’000 cells/100 μL. Cell suspensions were kept on ice in 96-well plates (Greiner 96 Well Suspension Culture Plate, 100 μL/well). The plates were incubated on ice for 30 min and subsequently centrifuged at 4 °C for 3 min (400 rcf). The supernatant was removed, and the cells were stained with Zombie NIR (live/dead staining) for 20 min at 4 °C in the dark. Cells were washed and cell pellets were resuspended in FACS buffer containing fluorescein-bearing test compounds (250 nM for PSMA binders, 50 nM for ACP3 binders, and 100 nM for CAIX binders). Plates were further incubated in the dark at rt for 1h and subsequently centrifuged at 4 °C for 3 min (400 rcf). Cells were washed and resuspended in 200 μL FACS buffer. Data were acquired halting the measurement at 10´000 events on a Cytoflex S flow cytometer (Beckman Coulter), and analyzed using the FlowJo software v10 (BD Biosciences).

2.18 Flow cytometry experiments on streptavidin beads

30 μL of Streptavidin Sepharose High Performance beads (Cytiva, # 90100484) were washed twice with 1 mL of water to remove the ethanol used as preservative agent. Beads were resuspended in 900 μL of PBS, and the suspension was split in two aliquots (450 μL/Eppendorf tube). One aliquot was incubated with 50 μL of NKG2D-Fc (6 μM in PBS), while the second aliquot was incubated with a biotin solution 40 μM in PBS (110 μL, negative control). The aliquots were shaken at 8 rpm for 1 hour in a Hula mixer. The suspensions were centrifuged at 2’500 rpm for 3 min, and the supernatants were removed. Free biotin (40 μM in 110 μL of PBS) was added to the NKG2D-Fc-coated beads, and the mixture was shaken at 8 rpm for 5 min in a Hula mixer. The beads were centrifuged at 2’500 rpm for 3 min, and the supernatant was removed. Both biotin- and NKG2D-Fc-coated beads were washed twice with FACS buffer (PBS 1x, 2 mM EDTA), resuspended in 140 μL of FACS buffer, and split in a 96-well

plate (20 μL/well). Test compounds (300 nM, in 175 μL of FACS buffer) were added to the wells containing the beads. Plates were further incubated in the dark at room temperature for 1 hour and subsequently centrifuged at 2'500 rpm at 4 °C for 3 min. Beads were washed and resuspended in FACS buffer (200 μL). Data were acquired halting the measurement at 10'000 events on a Cytoflex S flow cytometer (Beckman Coulter) and analyzed using the FlowJo software v10 (BD Biosciences).

2.19 In vitro interferon-gamma release assay

Compounds **26** and **30** were tested for their ability to induce interferon-gamma (IFN-γ) release upon incubation with NK-cells. NK-92 cells were initially starved for 4 h in plain RPMI medium. Cells were then resuspended at a density of 1×10^6 cells/mL in complete RPMI, and 100 µL of the cell suspension was incubated with a serial dilution of the compounds (initial concentration 7.5 μM in RPMI with 5% DMSO). After 24 h, cell-free supernatants were collected, and the concentration of IFN-γ was measured using the ELISA MAX*TM* Set Human IFN-γ kit (BioLegend).

2.20 Autoradiography experiments

Compounds were dissolved in DMSO (stock solutions at 1 mM) and diluted in sodium acetate buffer (1 M, pH 4.5) to a final concentration of 200 μM. Compound solutions (5 μL) were mixed with $^{177}LuCl_3$ (10 μL, 2 MBq/μL) and with sodium acetate buffer (5 μL, 1 M, pH 4.5). The mixture (20 μL) was heated at 90 °C for 10 min, left to equilibrate to room temperature, and diluted to 5 nM with 1% bovine serum albumin (BSA) in PBS. OCT-embedded frozen tissue slices (10 μm) were thawed at room temperature and fixed in ice-cold acetone for 15 min. The tissue sections were washed two times with PBS (pH 7.4) and dried at room temperature. A hydrophobic circle was drawn around the tissue with a Dako Pen. The sections were blocked with 20% FCS (Invitrogen) and 3% BSA in PBS (pH 7.4) for 30 min and washed three times with PBS (pH 7.4). The tissue slices were separately incubated with ^{177}Lu-labeled compounds (500 μL, 5 nM, ~ 50 kBq) for 1 hour at room temperature. Sections were subsequently washed three times with PBS (pH 7.4), dried at room temperature, and exposed to the phosphor screen overnight. Images were recorded on a CR-35 Bio scanner and processed with the AIDA image analysis software.

2.21 Quantitative in vivo biodistribution studies

SK-RC-52 cells were grown to 80% confluency, detached with trypsin–EDTA, collected by centrifugation (5 min at 1'000 g), and resuspended in sterile Hanks' Balanced Salt Solution at a density of 50 million cells/mL (Gibco, #14170-112). The cell suspension (~100 μL/mouse, ~5 million cells) was subcutaneously injected in the right flank of athymic BALB/c AnNRj-Foxn1 nude mice (female, age 4-8 weeks, Janvier). Upon reaching an average tumor size of ~300 mm^3, mice were intravenously injected with ^{177}Lu-labeled compounds (3.33 MBq/nmol, ~2 MBq/mouse, formulated in sterile PBS). Mice were

sacrificed at different time points post-injection, as indicated in the main article (**figure 5**, and corresponding figure legend). Blood was collected, and tumors and healthy organs were excised to measure radioactivity on a Packard Cobra Gamma Counter. Tissue uptake was calculated as %ID/g ± standard error of the mean (n = 3 mice/group). Measured values were normalized considering the radioactive decay of the corresponding radionuclide (^{177}Lu).

3 DEL synthesis, purification, and characterization

3.1 Ethanol precipitation

DNA was precipitated from aqueous phase by the addition of 10% v/v 5 M NaCl or 3 M acetic acid buffer (pH 5; only if prior to RP-HPLC purifications). After addition of 3 volumes of EtOH, the solution was shaken and left at -20 °C overnight. DNA was recovered as pellet by centrifugation (16´100 rcf, 4 °C, 1 h), the supernatant discarded, and the pellet dried under reduced pressure in a SpeedVac vacuum concentrator.

3.2 Synthesis of FM-DELs

3.2.1 Preparation of pool 1 of FM-DELs

Intermediate pool step 1 was synthesized following procedures described elsewhere[9]. A brief description of the synthetic steps to obtain pool step 1 is reported below.

3.2.1.1 Scaffold preparation and coupling to DNA: d1, d2, and d3

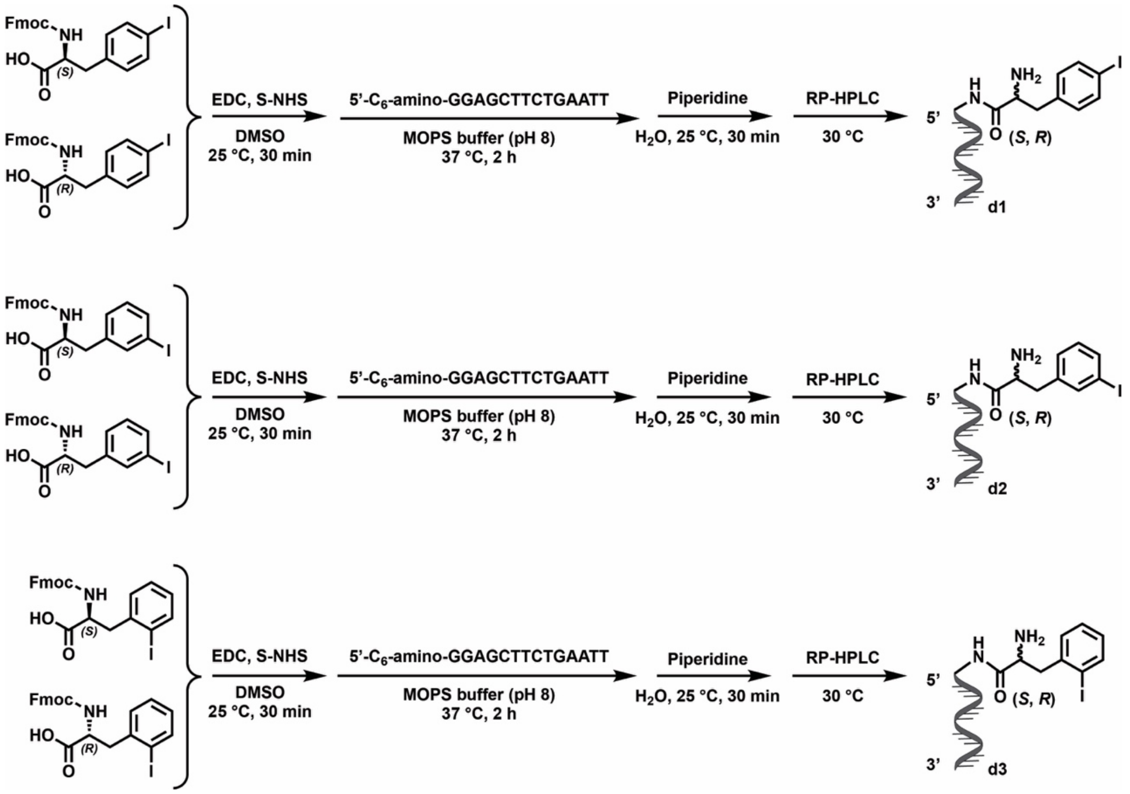

Figure S1. *Synthetic route for the conjugation of iodo-phenylalanine derivatives (library scaffolds) to the amino-modified universal 14-mer oligonucleotide[9].*

The racemic scaffold mixtures (three regioisomers of Fmoc-iodo-phenylalanine) were activated for 30 minutes using EDC/S-NHS. The activated scaffolds were separately added to a solution of amino-modified oligonucleotide (5'-C_6-amino-GGAGCTTCTGAATT-3') in MOPS buffer pH 8, and the resulting mixtures were reacted for 2 h. After EtOH precipitation, the -NH_2 moiety of the scaffolds was deprotected in the presence of a solution 10% v/v of piperidine (Fmoc removal), and the DNA was precipitated. The crude products were purified by HPLC. The flow rate was set to 4 mL/min at 30 °C (column temperature), applying the following gradient: 0-2 min 90% A, 2-10 min 95% to 50% A, 10-13 min 50% to 0% A, 13-17 min 0%, 17-17.1 min 90% A, 17.1-18 min 90% A. Fractions containing scaffold-oligonucleotide conjugates were concentrated under reduced pressure (SpeedVac), precipitated with ethanol, and re-dissolved in mQ water (2 mM final concentration). Compounds **d1**, **d2**, and **d3** were isolated with an average yield of ~ 60% (~ 6 µmol)[9].

3.2.1.2 Scaffold preparation and coupling to DNA: d4, and d5

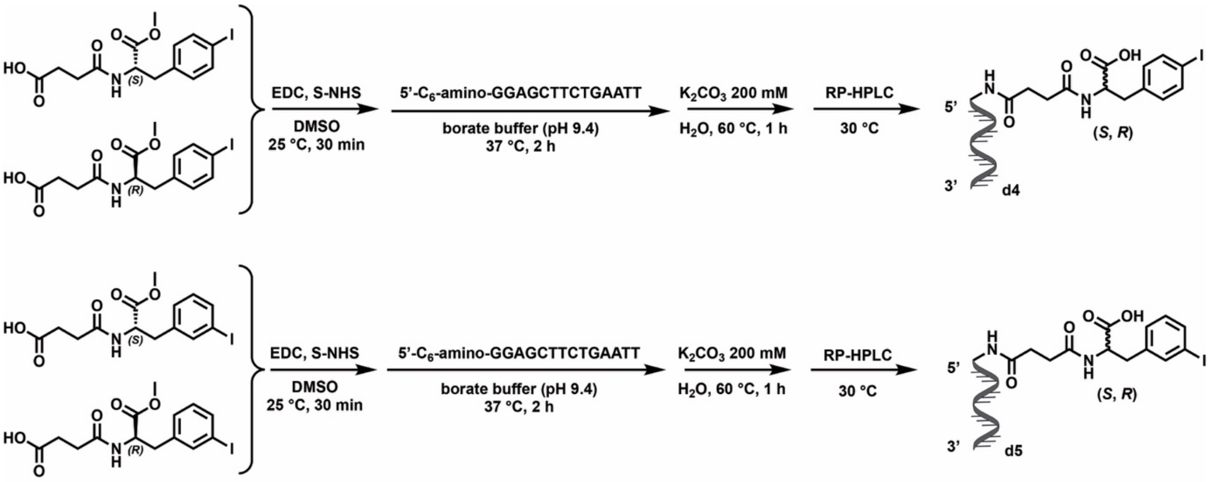

Figure S2. *Synthetic route for the conjugation of 4-((3-(iodophenyl)-1-methoxy-1-oxopropan-2-yl)amino)-4-oxobutanoic acid derivatives to the amino-modified universal 14-mer oligonucleotide[9].*

Racemic mixture of 4-((4-(iodophenyl)-1-methoxy-1-oxopropan-2-yl)amino)-4-oxobutanoic acid and 4-((3-(iodophenyl)-1-methoxy-1-oxopropan-2-yl)amino)-4-oxobutanoic acid were activated for 30 min with EDC/S-NHS. The activated scaffolds were separately added to a solution of amino-modified oligonucleotide (5'-C_6-amino-GGAGCTTCTGAATT-3') in borate buffer pH 9.4, and the resulting mixtures were shaken for 2 h. After EtOH precipitation, the -COOMe moiety of the scaffolds was deprotected (hydrolysis) by dissolving the DNA pellet in 200 mM K_2CO_3, and the DNA was precipitated. The crude products were HPLC-purified. The flow rate was set to 3.5 mL/min at 30 °C column temperature, applying the following gradient: 0-2 min 90% A, 2-12 min 90% to 50% A, 12-14 min 50% to 0% A, 14-15 min 0% A, 15-15.5 min 0% to 90% A, 15.5-16 min 90% A. Fractions containing scaffold-oligonucleotide conjugates were concentrated under reduced pressure (SpeedVac), precipitated with ethanol, and re-dissolved in mQ water (2 mM final concentration). DNA-conjugates **d4** and **d5** were isolated with an average yield of ~ 60% (~ 0.6 µmol)[9].

3.2.1.3 Scaffold preparation and coupling to DNA: d6, d7 and d8

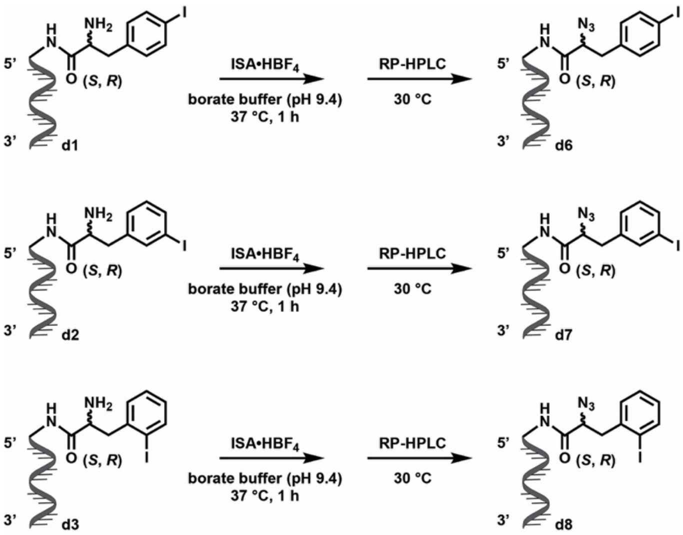

Figure S3. *Synthetic route for the obtainment of conjugates* ***d6***, ***d7***, *and* ***d8*** *after On-DNA diazo-transfer reaction*[9].

DNA-conjugates **d1**, **d2**, or **d3** were separately dissolved borate buffer pH 9.4, and a solution of imidazole-1-sulfonyl azide tetrafluoroborate (ISA•HBF_4) was added to the mixture. The reactions were shaken for 1 h at 37 °C. The DNA was ethanol precipitated and the crude products were HPLC-purified. The flow rate was set to 4 mL/min at 30 °C (column temperature), applying the following gradient: 0-2 min 90% A, 2-10 min 95% to 50% A, 10-13 min 50% to 0% A, 13-17 min 0%, 17-17.1 min 90% A, 17.1-18 min 90% A. Fractions containing scaffold-oligonucleotide conjugates were concentrated under reduced pressure (SpeedVac), precipitated with ethanol, and re-dissolved in mQ water (2 mM final concentration). DNA-conjugates **d6**, **d7**, and **d8** were isolated with an average yield of ~ 60% (~ 0.6 µmol)[9].

3.2.1.4 Synthesis of pool step 1

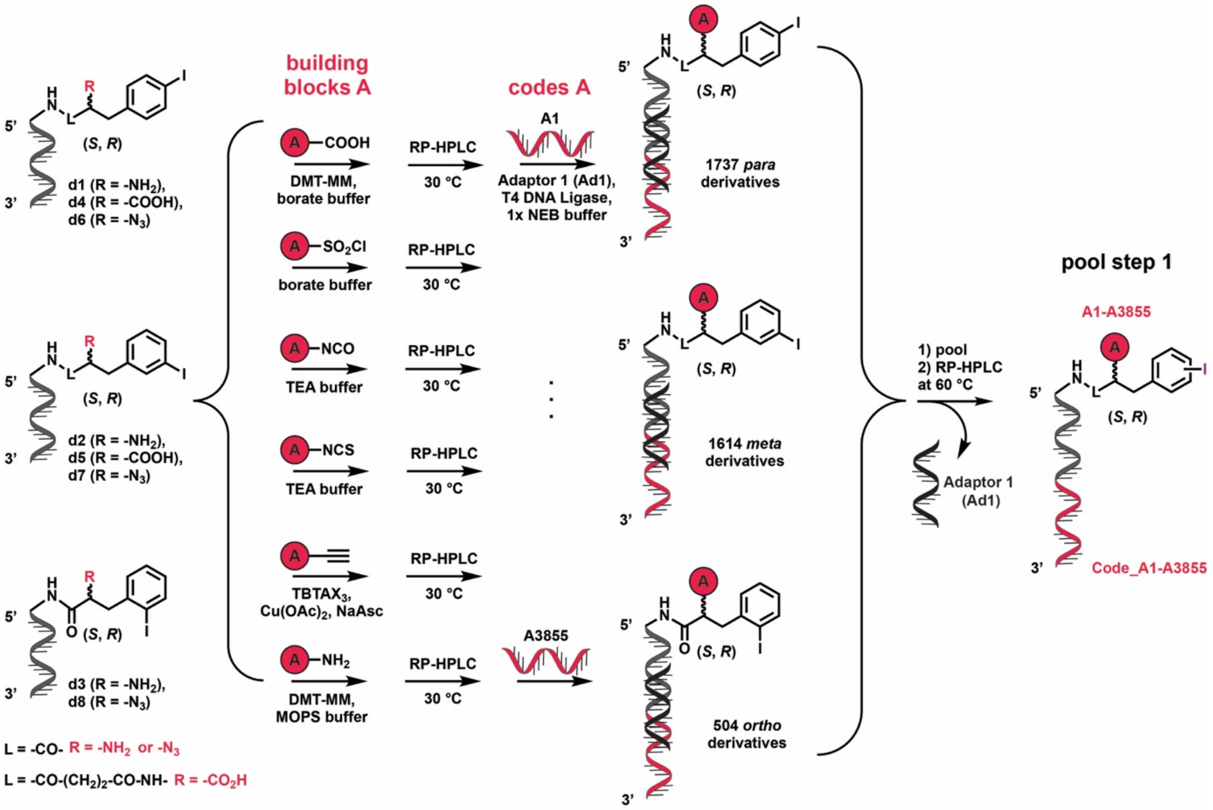

Figure S4. *Synthesis of pool step 1. Building blocks A (i.e., carboxylic acids, sulfonyl chlorides, isocyanates, isothiocyanates, alkynes, and amines) were individually reacted with phenylalanine scaffolds. The resulting products were purified by RP-HPLC at 30 °C, and each conjugate was encoded by enzymatic splint ligation with 29-mer oligonucleotides (Code As). The DNA derivatives were pooled and purified by RP-HPLC at 60 °C to obtain the pool step 1[9].*

Before pool step 1 synthesis, we screened and compared various conditions for coupling sulfonyl chlorides, isocyanates, and isothiocyanates to a single-stranded 5’ amino-modified 14-mer oligonucleotide that is reported in a previous article[10]. We aimed to identify optimal protocols for the on-DNA synthesis of sulfonamides, ureas, and thioureas that were employed during FM-DEL platform construction.

Phenylalanine scaffolds were reacted with building blocks A (i.e., carboxylic acids, sulfonyl chlorides, isocyanates, isothiocyanates, alkynes, and amines) yielding 3’855 DNA-conjugates. Each reaction was ethanol precipitated and the resulting DNA pellet was dissolved in 0.1 M TEAA buffer and purified by HPLC. The flow rate was set to 4 mL/min at 30 °C (column temperature), applying the following gradient: 0-2 min 90% A, 2-10 min 90% to 50% A, 10-13 min 50% to 0% A, 13-16 min 0% A, 16-16.2 min 0% to 90% A 16.2-18 min 90% A. Product containing fractions were dried under reduced pressure (SpeedVac) and ethanol precipitated. The purified 3`855 conjugates were re-dissolved in mQ water, normalizing their concentration to 10 µM. 2 nmol of each conjugate was encoded by enzymatic splint ligation with

29-mer oligonucleotide (code As; 5' PO4-CTGTGTGCTG**XXXXXX**CGAGTCCCATGGCGC 3') in presence of the adaptor 1 (Ad1; 5' CAGCACACAGAATTCAGAAGCTCC 3'). The reactions were pooled and purified by RP-HPLC at 60 °C to remove the adaptor and unreacted codes, as well as buffer ingredients used for enzymatic ligation. The flow rate of the mobile phase for RP-HPLC was set to 4 mL/min at 60 °C (column temperature), applying the following gradient: 0-1 min 95% A, 1-14 min 95% to 83% A, 14-15 min 83% to 20% A, 15-20 min 20% A, 20-20.5 min 20% to 0% A, 20.5-22 min 0% A; 22-22.5 min 0% to 95% A, 22.5-23 min 95% A. Fractions containing the purified pool were dried under reduced pressure (SpeedVac), ethanol precipitated, and re-dissolved in mQ water. The pool step 1 was isolated with a total yield of ~69% (~5.40 µmol)[9].

3.2.2 Synthesis of FM-DEL1

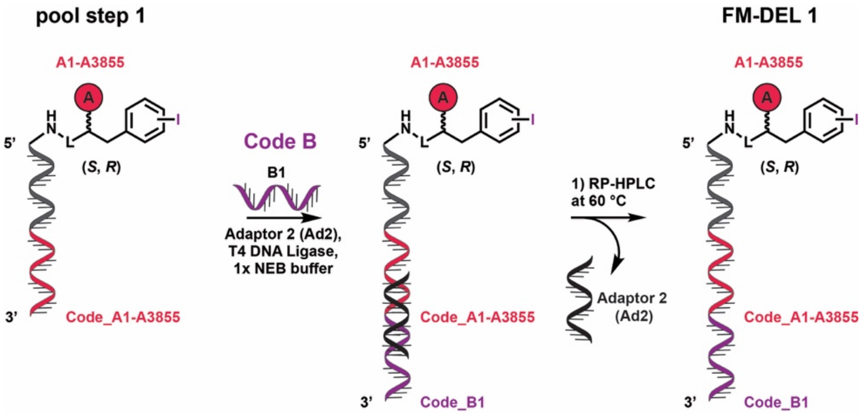

Figure S5. *Synthesis of FM-DEL1. Pool step 1 was encoded by enzymatic splint ligation with a 31-mer oligonucleotide (Code B1). The elongated pool 1 (FM-DEL1) was purified by RP-HPLC at 60 °C to remove the adaptor and unreacted code, as well as buffer ingredients used for enzymatic ligation.*

Pool step 1 (96 *µ*L, 208.5 µM in mQ water, 20 nmol, 1 equiv.), code B1 (130 *µ*L, 229 µM in mQ water, 30 nmol, 1.5 equiv.), adaptor 2 (Ad2, 68*µ*L, 383 µM in mQ water, 26 nmol, 1.3 equiv.), and 10x T4 DNA Ligase Reaction Buffer (300 *µ*L, New England Biolabs, #B0202S). The reaction was heated for 10 min at 70 °C and subsequently left at rt for 30 min to facilitate the annealing of the oligonucleotides. T4 DNA ligase (New England Biolabs, #M0202L, 400`000 units/mL) was diluted with 10x T4 DNA Ligase Reaction Buffer (1:10 dilution) and added to the ligation reaction (100 *µ*L/ligation, 4`000 units/ligation). The ligation proceeded for 2 h at rt and was subsequently quenched by heating for 10 min at 65 °C. The reaction was ethanol precipitated to generate the FM-DEL1.

The FM-DEL1 was re-dissolved in 0.1 M TEAA buffer and filtered through a 0.2 µm filter. The flow rate of the mobile phase for RP-HPLC was set to 4 mL/min at 60 °C (column temperature), applying the following gradient: 0-1 min 95% A, 1-14 min 95% to 83% A, 14-15 min 83% to 20% A, 15-20 min 20% A, 20-20.5 min 20% to 0% A, 20.5-25 min 0% A; 25-25.5 min 0% to 95% A, 25.5-27.5 min 95% A.

Fractions containing the purified FM-DEL1 were dried under reduced pressure (SpeedVac), precipitated with ethanol, and re-dissolved in mQ water. The FM-DEL1 was isolated with a total yield of ~ 60% (~ 12 nmol).

Code B1: (5' PO_4-CGGATCGACG**GTCTCAC**GCGTCAGGCAGC 3')
(**GTCTCAC** = coding region)
Adaptor 2 (Ad2): 5' CGTCGATCCGGCGCCATGG
FM-DEL1 sequence (74-mer):
5' Modif.-GGAGCTTCTGAATTCTGTGTGCTG**XXXXXX**CGAGTCCCATGGCGCCGGATCGACG**GTCTCAC**GCGTCAGGCAGC

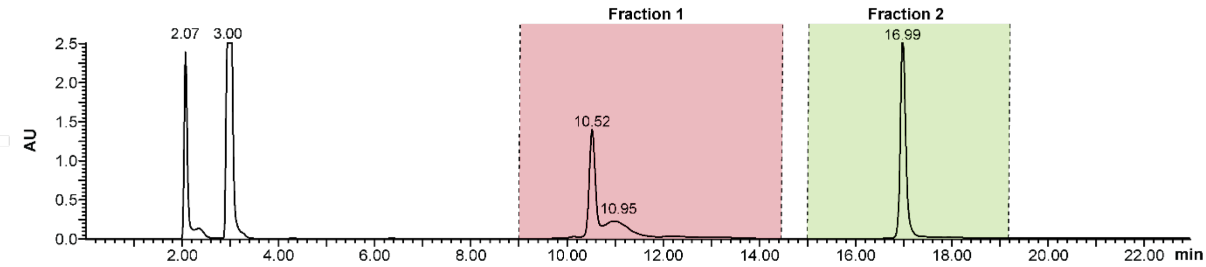

Figure S6. *RP-HPLC chromatogram (AU at λ = 260 nm) of the FM-DEL1 purification. Fraction 1 highlighted in red: Adaptor 2 (Ad2) and unreacted code B1. Fraction 2 highlighted in green: encoded product (FM-DEL1).*

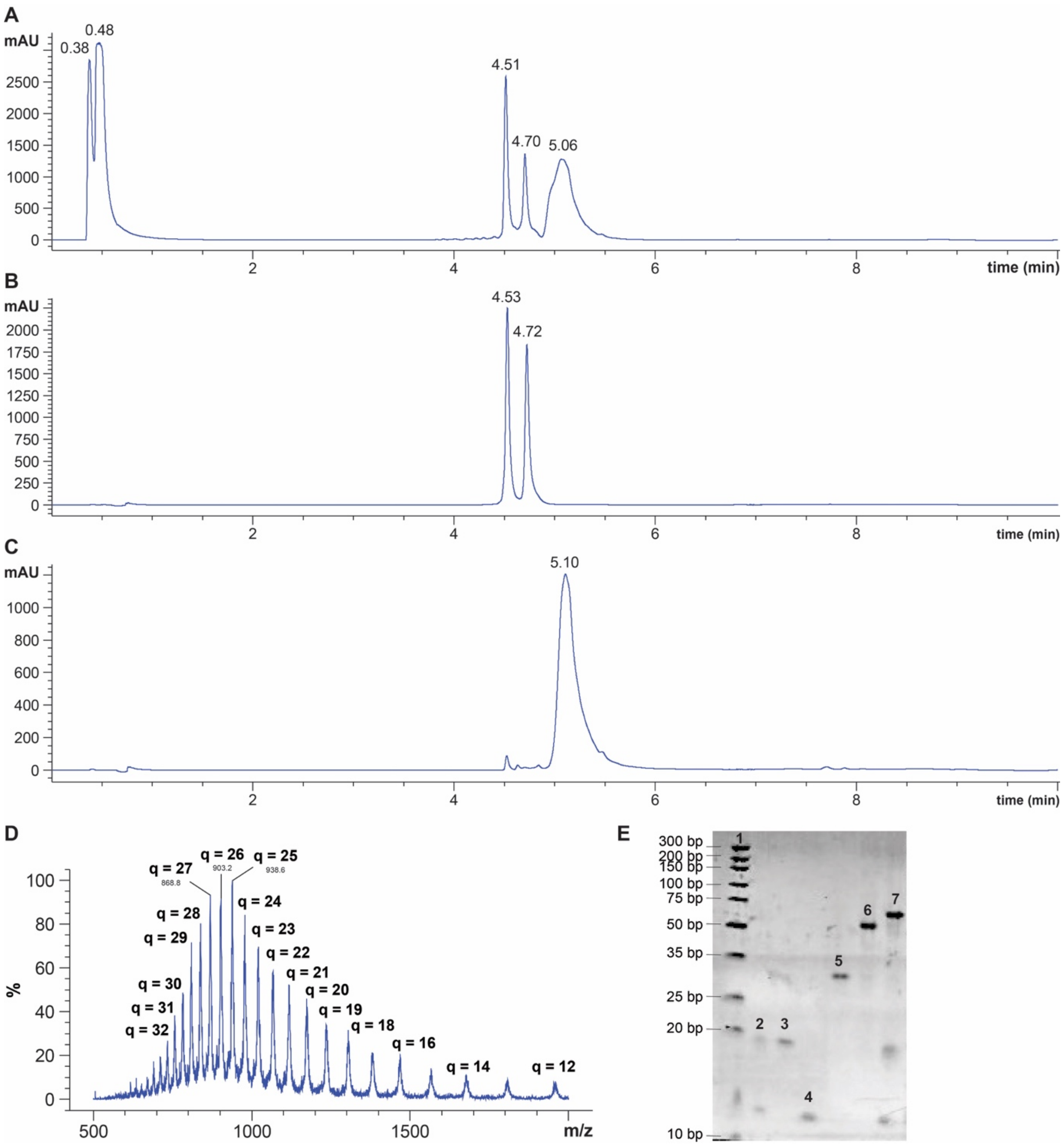

Figure S7. *Quality control of FM-DEL1 purification. LC chromatograms (mAU at λ = 260 nm – Method A) of the FM-DEL1 (**A**) before RP-HPLC purification and (**B**) & (**C**) after RP-HPLC purification (fraction 1 & fraction 2, respectively). (**D**) MS-spectrum (TOF negative mode) of purified FM-DLE1 after HPLC purification. The average mass of the pool is 23.5 kDa, length = 74-mer. q = charge; (**E**) 15% TBE-Urea gel where **1** = ladder, **2** = fraction 1 (Ad2 and unreacted code B1), **3** = ref. code B1, **4** = ref. Ad2, **5** = purified pool step 1, **6** = fraction 2 (FM-DEL1), **7** = crude FM-DEL1.*

3.2.3 Synthesis of FM-DEL2

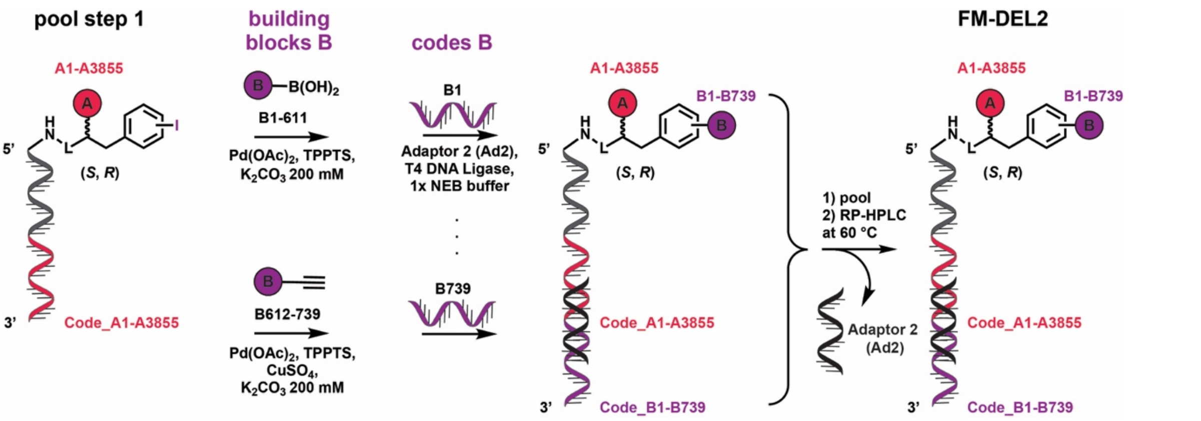

Figure S8. *Synthesis of FM-DEL2. The pool stel 1 was separately coupled to 611 boronic acids and 128 arylalkynes via Suzuki and Sonogashira cross-coupling, respectively. The reactions were encoded by enzymatic splint ligation and pooled to generate FM-DEL2 that was purified by RP-HPLC at 60 °C to remove the adaptor and unreacted codes, as well as buffer ingredients used for enzymatic ligation.*

Suzuki cross-coupling (1.5 nmol scale)

All solvents were degassed in argon atmosphere. The pre-catalyst solution was prepared by mixing $Pd(OAc)_2$ (100 µL, 10 mM in DMA), 3,3′,3′′-phosphanetriyltris(benzenesulfonic acid) trisodium salt (TPPTS, 100 µL, 100 mM in mQ water), and mQ water (800 µL) resulting in a solution of Pd(0)-TPPTS complex (1 mM). To each reaction vessel, an aliquot of pool step 1 (1.5 nmol, 1 equiv.) in 200 mM K_2CO_3 (20 µL) was added. Pre-catalyst solution (4 µL, containing 4 nmol of Pd(0)-TPPTS, 1.3 equiv.) and the boronic acid (20 µL, 100 mM in DMA, 666 equiv.) were subsequently added to the mixture that was heated at 65 °C for 1 h. The reaction was quenched by the addition of dithiothreitol (DTT, 10 µL, 200 mM in mQ water) and 3 M acetate buffer (5 µL, pH 5). The products were isolated by ethanol precipitation.

Sonogashira cross-coupling (1.5 nmol scale)

All solvents were degassed in argon atmosphere. The pre-catalyst solution was prepared by mixing $Pd(OAc)_2$ (100 µL, 10 mM in DMA), 3,3′,3′′-phosphanetriyltris(benzenesulfonic acid) trisodium salt (TPPTS, 100 µL, 100 mM in mQ water), $CuSO_4$ (100 µL, 20 mM in mQ water), and mQ water (700 µL) resulting in a solution of Pd(0)-TPPTS complex (1 mM) and Cu^{2+} (2 mM). To each reaction vessel, an aliquot of pool step 1 (1.5 nmol, 1 equiv.) in 200 mM K_2CO_3 (20 µL) was added. Pre-catalyst solution (4 µL, containing 4 nmol of Pd(0)-TPPTS, 1.3 equiv.) and the alkynes (10 µL, 100 mM in DMSO, 666 equiv.) were subsequently added to the mixture. The copper was reduced by adding NaAsc (10 µL, 10 mM in mQ water, 66 equiv.), and the reaction was heated at 65 °C for 1 h. The reaction was quenched by the addition of dithiothreitol (DTT, 10 µL, 200 mM in mQ water) and 3 M acetate buffer (5 µL, pH 5). The products were isolated by ethanol precipitation.

Splint ligation - Code B

Dry pellets from the step 2 reactions were mixed with the corresponding code B (50 µL, 50 µM in mQ water, 2.55 nmol, 1.7 equiv.), Ad2 (4.8 µL, 620 µM in mQ water, 3.0 nmol, 2 equiv.), and 10x T4 DNA Ligase Reaction Buffer (15 µL, New England Biolabs, #B0202S). The reactions were heated for 10 min at 70 °C and subsequently left at rt for 30 min to facilitate the annealing of oligonucleotides. T4 DNA ligase (New England Biolabs, #M0202L, 400´000 units/mL) was diluted with 10x T4 DNA Ligase Reaction Buffer (1:10 dilution) and added to each ligation reaction (10 µL/ligation, 400 units/ligation). The ligation proceeded for 16 h at rt and was subsequently quenched by heating for 10 min at 65 °C. The 739 ligation products were pooled, concentrated under reduced pressure, and ethanol precipitated to generate the FM-DEL2.

Code B: 5' PO4-CGGATCGACG**XXXXXXX**GCGTCAGGCAGC
(**XXXXXXX** = coding region with 128 different conjugate-specific codes)
Adaptor 2 (Ad2): 5' CGTCGATCCGGCGCCATGG
FMA-DEL (step 2) sequence (74-mer):
5' Modif.-GGAGCTTCTGAATTCTGTGTGCTG**XXXXXX**CGAGTCCCATGGCGCCGGATCGACG**XXXXXXX**GCGTCAGGCAGC

HPLC purification of FM-DEL2

The FM-DEL2 was re-dissolved in 0.1 M TEAA buffer. Prior to RP-HPLC purification, small molecules that were not soluble in TEAA buffer were removed by centrifugation and filtration through a 0.2 µm filter. The flow rate of the mobile phase for RP-HPLC was set to 4 mL/min at 60 °C (column temperature), applying the following gradient: 0-1 min 95% A, 1-14 min 95% to 83% A, 14-15 min 83% to 20% A, 15-20 min 20% A, 20-20.5 min 20% to 0% A, 20.5-22 min 0% A; 22-22.5 min 0% to 95% A, 22.5-23 min 95% A. Fractions containing the purified FM-DEL2 were dried under reduced pressure (SpeedVac), ethanol precipitated, and re-dissolved in mQ water. The FM-DEL2 was isolated with a total yield of ~57% (~ 632 nmol).

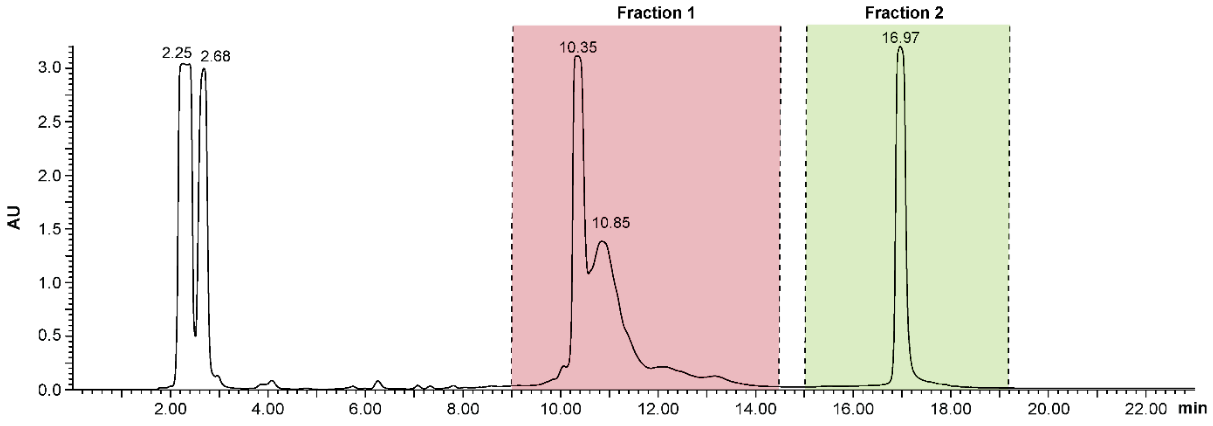

Figure S9. *RP-HPLC chromatogram (AU at λ = 260 nm) of the FM-DEL2 purification. Fraction 1 highlighted in blue: Ad2 and unreacted code Bs. Fraction 2 highlighted in yellow: encoded step 2 (FM-DEL2).*

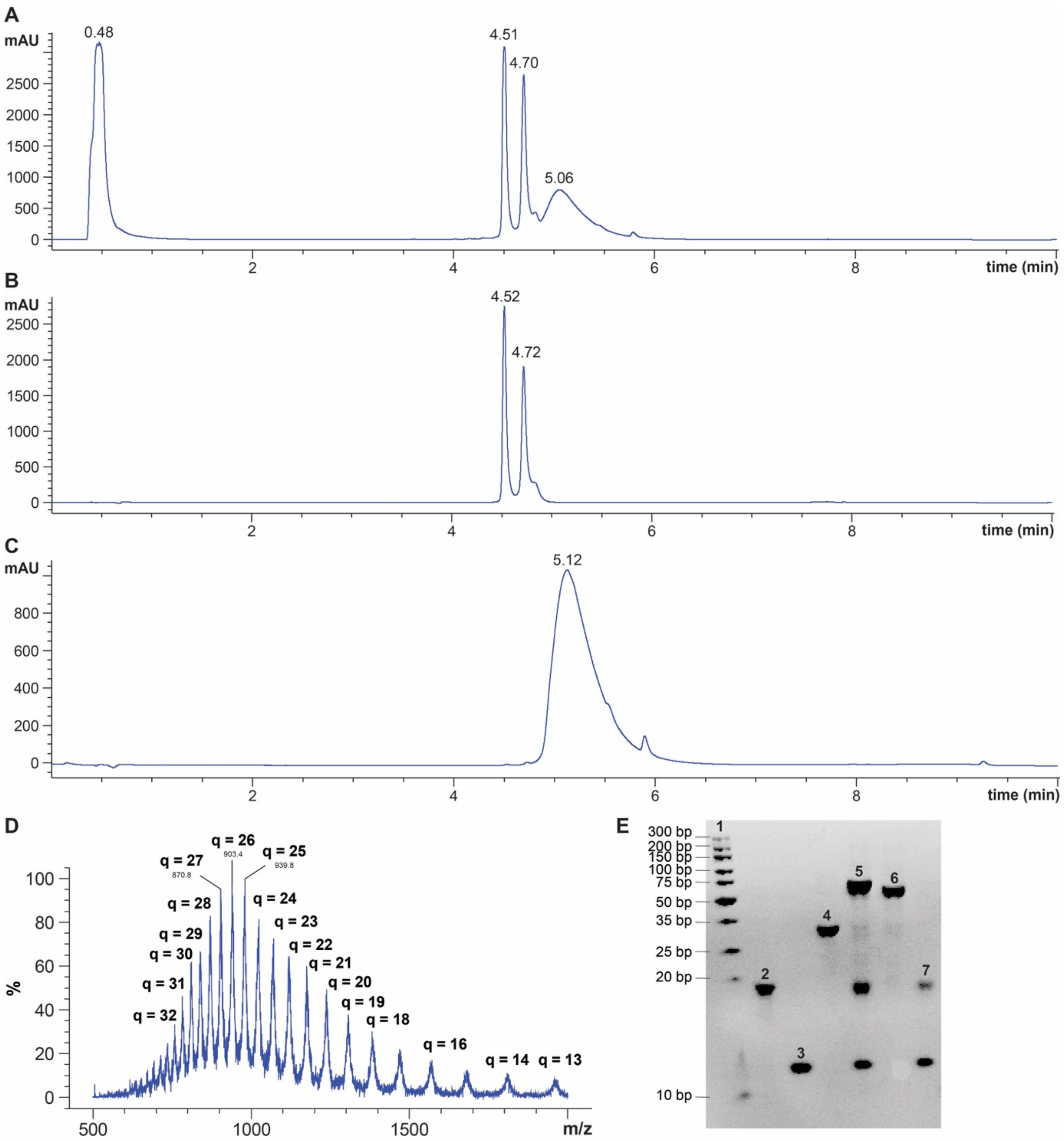

Figure S10. *Quality control of FM-DEL2 purification. LC chromatograms (mAU at λ = 260 nm – Method A) of the FM-DEL2 (**A**) before RP-HPLC purification and (**B**) & (**C**) after RP-HPLC purification (fraction 1 & fraction 2, respectively). (**D**) MS-spectrum (TOF negative mode) of purified FM-DEL2 after HPLC purification. The average mass of the pool is 23.5 kDa, length = 74-mer. q = charge; (**E**) 15% TBE-Urea gel where **1** = ladder, **2** = ref. code B, **3** = ref. Ad2, **4** = purified pool step 1, **5** = crude FM-DEL2, **6** = fraction 2 (FM-DEL2), **7** = fraction 1 (Ad2 and unreacted code Bs).*

3.3 Encoding design for FM-DELs

Universal oligonucleotide (14-mer)

5' Modif.-GGAGCTTCTGAATT

Step1 (45-mer)

5' Modif.-GGAGCTTCTGAATTCTGTGTGCTG**XXXXXX**CGAGTCCCATGGCGC

Step2 (74-mer)

5' Modif.-GGAGCTTCTGAATTCTGTGTGCTG**XXXXXX**CGAGTCCCATGGCGCCGGATCGACG**XXXXXXX**GCGTCAGGCAGC

CodeA: 5' PO4-CTGTGTGCTG**XXXXXX**CGAGTCCCATGGCGC

CodeB: 5' PO4-CGGATCGACG**XXXXXXX**GCGTCAGGCAGC

Adapter 1 (Ad1): 5' CAGCACACAGAATTCAGAAGCTCC

Adapter 2 (Ad2) 5' CGTCGATCCGGCGCCATGG

3.4 Solid-phase affinity selections: PCR amplification and sequencing

The selection eluates were amplified with two PCR steps (PCR1 and PCR2)[11].
The following PCR primers were used:

PCR1-a:

5' TACACGACGCTCTTCCGATCT**XXXXXX**GGAGCTTCTGAATTCTGTGTG

(XXXXXX = variable region to encode the selection)

PCR1-b: 5' CAGACGTGTGCTCTTCCGATC**XXXXXX**GCTGCCTGACGC

(XXXXXX = variable region to encode the selection)

PCR2a:

5' AATGATACGGCGACCACCGAGATCTACACTCTTTCCCTACACGACGCTCTTCCGATCT

PCR2b:

5' CAAGCAGAAGACGGCATACGAGATATTGGCGTGACTGGAGTTCAGACGTGTGCTCTTCCGATC

PCR2 pools were processed and analysed after Illumina high-throughput sequencing (NovaSeq)[12].

4 Proteins used for selections: design, production, and quality control

4.1 Prostate-specific membrane antigen (PSMA)

Human recombinant PSMA was designed, produced in CHO cells as described in the literature[13]. The protein was site-specifically biotinylated as described in **General Methods**.

Quality control of PSMA biotinylation (SDS-PAGE)

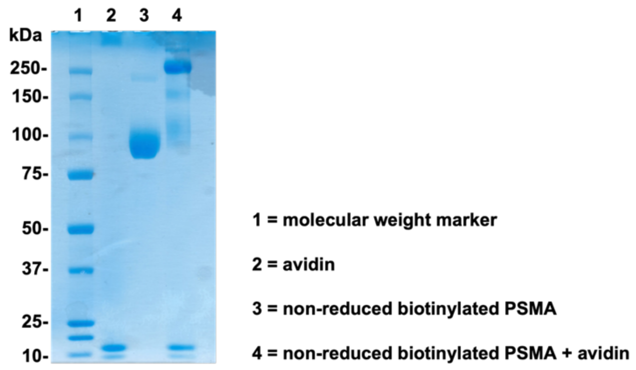

Buffer used for selections: HEPES buffer (50 mM HEPES, 100 mM NaCl, 1 mM $CaCl_2$, 0.5 mM $MgCl_2$, pH 7.4)

Molecular weight: 82.5 kDa (monomer)

ε (280 nm), $M^{-1}cm^{-1}$: 118'735

4.2 Glutamate carboxypeptidase III (GCP3)

Human recombinant GCP3 was designed as described in the literature[13].

The protein was produced in CHO cells and site-specifically biotinylated as described in **General Methods**.

Quality control of GCP3 biotinylation (SDS-PAGE)

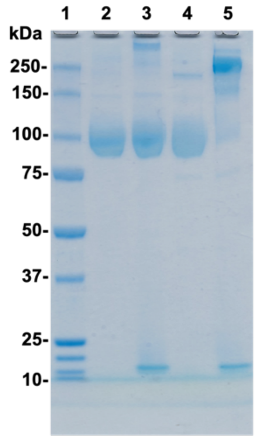

1 = molecular weight marker

2 = non-reduced GCP3

3 = non-reduced GCP3 + avidin

4 = non-reduced biotinylated GCP3

5 = non-reduced biotinylated GCP3 + avidin

Buffer used for selections: HEPES buffer (50 mM HEPES, 100 mM NaCl, pH 7.4)

Molecular weight: 83.0 kDa (monomer)

ε (280 nm), $M^{-1}cm^{-1}$: 120’225

4.3 Human prostatic acid phosphatase (hACP3)

Human recombinant ACP3 was designed as described in the literature[9].

The protein was produced in CHO cells and site-specifically biotinylated as described in **General Methods**.

Quality control of hACP3 biotinylation (SDS-PAGE)

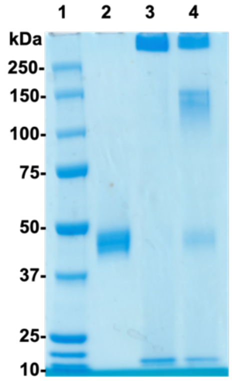

1 = molecular weight marker

2 = non-reduced biotinylated hACP3

3 = avidin

4 = non-reduced biotinylated hACP3 + avidin

Buffer used for selections: PBS (pH 7.4)

Molecular weight: 43.9 kDa (monomer)

ε (280 nm), $M^{-1}cm^{-1}$: 67’185

4.4 Murine prostatic acid phosphatase (mACP3)

Protein design:

The gene for the expression of a secreted variant of mACP3 (AA 32-381, UniProt: Q8CE08) in CHO cells, has been design by replacing the endogenous signal peptide with the signal peptide from a murine IgG and by adding an His-tag and an Avi-tag at the C-terminus of the protein. The corresponding codon optimized sequence has been custom synthesized by Genscript and cloned into the pcDNA3.1 mammalian expression vector using HindIII-NotI restriction sites (see aminoacidic sequence below).

KELKFVTLVFRHGDRGPIETFPTDPITESSWPQGFGQLTQWGMEQHYELGSYIRKR
YGRFLNDTYKHDQIYIRSTDVDRTLMSAMTNLAALFPPEGISIWNPRLLWQPIPVHT
VSLSEDRLLYLPFRDCPRFEELKSETLESEEFLKRLHPYKSFLDTLSSLSGFDDQDL
FGIWSKVYDPLFCESVHNFTLPSWATEDAMIKLKELSELSLLSLYGIHKQKEKSRLQ
GGVLVNEILKNMKLATQPQKYKKLVMYSAHDTTVSGLQMALDVYNGVLPPYASCH
MMELYHDKGGHFVEMYYRNETQNEPYPLTLPGCTHSCPLEKFAELLDPVISQDWA
TECMATSSHQGRNGSGLNDIFEAQKIEWHEGSHHHHHH

Protein production and purification:

The protein was produced in CHO cells as described in **General Methods**.

Quality control of mACP3 production (SEC and SDS-PAGE)

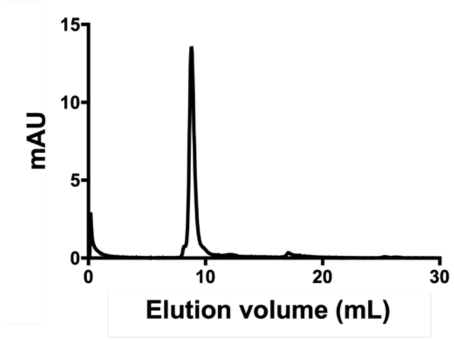

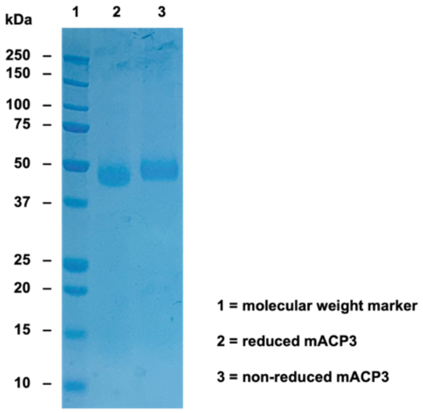

Protein biotinylation:

The protein was site-specific biotinylated as described in **General Methods**.

Quality control of mACP3 biotinylation (SDS-PAGE)

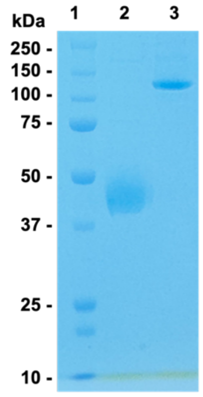

1 = molecular weight marker

2 = non-reduced biotinylated mACP3

3 = non-reduced biotinylated mACP3 + avidin

Buffer used for selections: PBS (pH 7.4)

Molecular weight: 43.4 kDa (monomer)

ε (280 nm), $M^{-1}cm^{-1}$: 69'705

4.5 H44A, H289A human prostatic acid phosphatase mutant (H44A, H289A hACP3)

Protein design:

The gene for the expression of a secreted variant of double mutant human ACP3 (AA 31-380, H44A, H289A; UniProt: P15309) in CHO cells, has been design by replacing the endogenous signal peptide with the signal peptide from a murine IgG and by adding an His-tag and an Avi-tag at the C-terminus of the protein. The corresponding codon optimized sequence has been custom synthesized by Genscript and cloned into the pcDNA3.1 mammalian expression vector using HindIII-NotI restriction sites (see aminoacidic sequence below).

KELKFVTLVFR**A**GDRSPIDTFPTDPIKESSWPQGFGQLTQLGMEQHYELGEYIRKR
YRKFLNESYKHEQVYIRSTDVDRTLMSAMTNLAALFPPEGVSIWNPILLWQPIPVHT
VPLSEDQLLYLPFRNCPRFQELESETLKSEEFQKRLHPYKDFIATLGKLSGLHGQDL
FGIWSKVYDPLYCESVHNFTLPSWATEDTMTKLRELSELSLLSLYGIHKQKEKSRLQ
GGVLVNEILNHMKRATQIPSYKKLIMYSA**A**DTTVSGLQMALDVYNGLLPPYASCHLT
ELYFEKGEYFVEMYYRNETQHEPYPLMLPGCSPSCPLERFAELVGPVIPQDWSTE
CMTTNSHQGTEDSTDGSGLNDIFEAQKIEWHEGSHHHHHH

Protein production and purification:

The protein was produced in CHO cells as described in **General Methods**.

Quality control of H44A, H289A hACP3 production (SEC and SDS-PAGE)

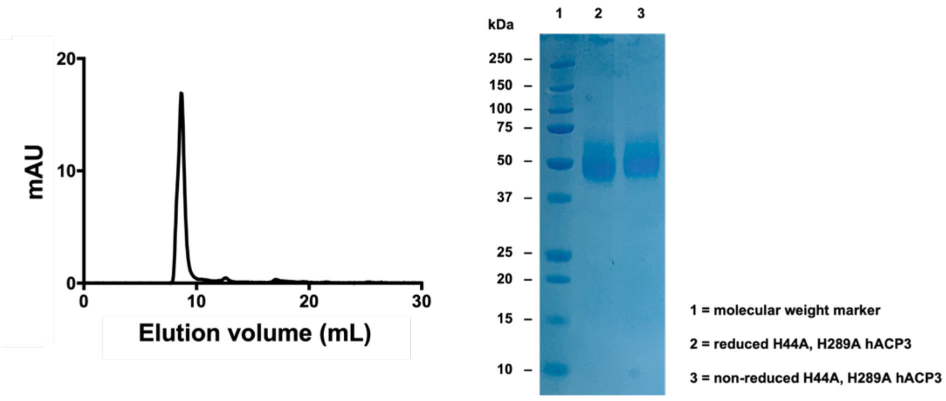

Protein biotinylation:

The protein was site-specific biotinylated as described in **General Methods**.

Quality control of H44A, H289A hACP3 biotinylation (SDS-PAGE)

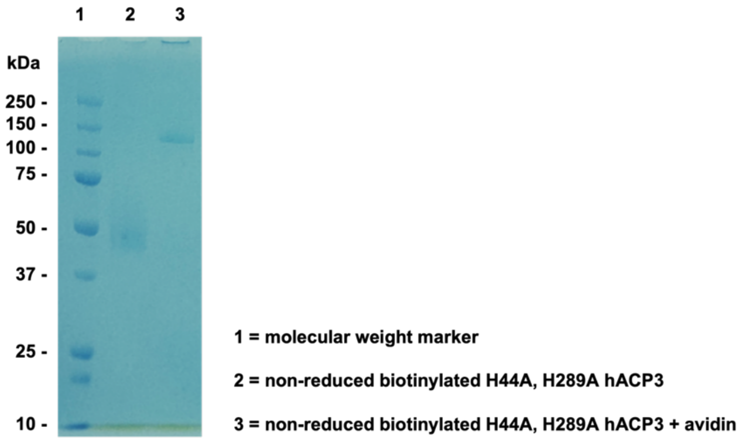

Buffer used for selections: PBS (pH 7.4)

Molecular weight: 43.8 kDa (monomer)

ε (280 nm), $M^{-1}cm^{-1}$: 67’185

4.6 Tissue non-specific alkaline phosphatase (TNAP)

Human recombinant TNAP was designed, produced in CHO cells as described in the literature[9]. The protein was chemically biotinylated as described in **General Methods**.

<u>Quality control of TNAP biotinylation (SDS-PAGE)</u>

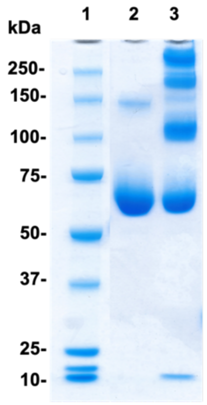

1 = molecular weight marker

2 = non-reduced biotinylated TNAP

3 = non-reduced biotinylated TNAP + avidin

Ladder lane (M) was run in a non-adjacent lane on the same blot and re-arranged when preparing the figure.

<u>Buffer used for selections:</u> HEPES buffer (25 mM HEPES, 150 mM NaCl, 2 mM $MgCl_2$, pH 7.4)

<u>Molecular weight:</u> 54.0 kDa (monomer)

<u>ε (280 nm), $M^{-1}cm^{-1}$:</u> 57´550

4.7 Carbonic anhydrase IX (CAIX)

Human recombinant CAIX was designed as described in the literature[11].

The protein was produced in CHO cells and chemically biotinylated as described in **General Methods**.

Quality control of CAIX biotinylation (SDS-PAGE)

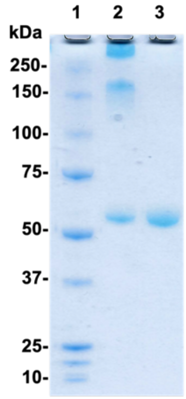

1 = molecular weight marker

2 = non-reduced biotinylated CAIX + avidin

3 = non-reduced biotinylated CAIX

Buffer used for selections: PBS (pH 7.4)

Molecular weight: 32.7 kDa (monomer)

ε (280 nm), $M^{-1}cm^{-1}$: 34´850

4.8 Carbonic anhydrase II (CAII)

Bovine recombinant CAII was bought from Sigma Aldrich (C2624-100MG).
The protein was chemically biotinylated as described in **General Methods**.

Quality control of CAII biotinylation (SDS-PAGE)

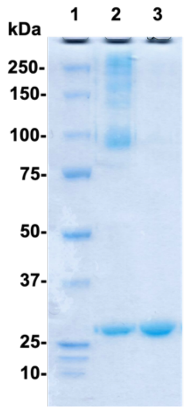

1 = molecular weight marker

2 = non-reduced biotinylated CAII + avidin

3 = non-reduced biotinylated CAII

Buffer used for selections: PBS (pH 7.4)

Molecular weight: 29.0 kDa (monomer)

ε (280 nm), $M^{-1}cm^{-1}$: 50´420

4.9 Natural Killer group 2D Fc fusion (NKG2D-Fc)

Protein design:

The pcDNA 3.1(+) expression vector containing a plasmid with the extracellular domain of human NKG2D (AA90216, Uniprot: P26718) followed by the Fc region of human IgG1 and an avi-tag at the C-terminus for site-specific biotinylation purposes was purchased (Genscript). After transformation of 100ng into TG1, sequence was checked (see below).

PLTESYCGPCPKNWICYKNNCYQFFDESKNWYESQASCMSQNASLLKVYSKEDQDLLKLVKSYHW
MGLVHIPTNGSWQWEDGSILSPNLLTIIEMQKGDCALYASSFKGYIENCSTPNTYICMQRTVGSEPKS
SDKTHTCPPCPAPELLGGPSVFLFPPKPKDTLMISRTPEVTCVVVDVSHEDPEVKFNWYVDGVEVHN
AKTKPREEQYNSTYRVVSVLTVLHQDWLNGKEYKCKVSNKALPAPIEKTISKAKGQPREPQVYTLPP
SRDELTKNQVSLTCLVKGFYPSDIAVEWESNGQPENNYKTTPPVLDSDGSFFLYSKLTVDKSRWQQ
GNVFSCSVMHEALHNHYTQKSLSLSPGKGSSGGLNDIFEAQKIEWHE

Protein production and purification:

The protein was produced in CHO cells as described in **General Methods**.

Quality control of NKG2D-Fc production (SEC)

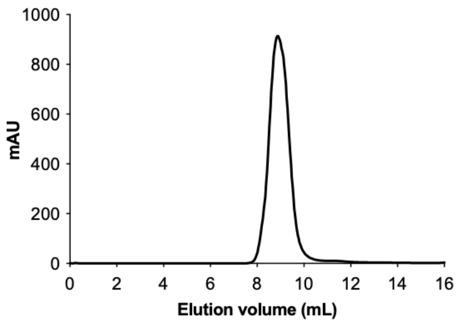

Protein biotinylation:

The protein was site-specific biotinylated as described in **General Methods**.

Quality control of NKG2D-Fc biotinylation (SDS-PAGE)

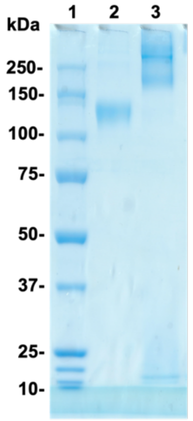

1 = molecular weight marker

2 = non-reduced biotinylated NKG2D-Fc

3 = non-reduced biotinylated NKG2D-Fc + avidin

Buffer used for selections: PBS (pH 7.4)

Molecular weight: 120.0 kDa (dimer)

ε (280 nm), $M^{-1}cm^{-1}$: 228´480

4.10 Fragment crystallizable (Fc) of human IgG1

Protein design:

The sequence containing the Fc region of human IgG1 and an avi-tag at the C-terminus for site-specific biotinylation purposes was obtained after amplifying the corresponding sequence using as a template the plasmid NKG2D-Fc. The following primers were used for the different PCRs:

PCR 1

Forward (5'-3'):

CCTGTTCCTCGTCGCTGTGGCTACAGGTGTGCACTCGGAGCCCAAATCTTCTGACAAAAC

Reverse (5'-3'): TTTTCCTTTTGCGGCCGCTTATCATTCATGCCACTCAATCTTCTGTGCTT

PCR 2

Forward (5'-3'):

CCCAAGCTTGTCGACCATGGGCTGGAGCCTGATCCTCCTGTTCCTCGTCGCTGTGGCTAC

Reverse (5'-3'): TTTTCCTTTTGCGGCCGCTTATCATTCATGCCACTCAATCTTCTGTGCTT

After transformation of 100ng into TG1, sequence was checked (see below).

EPKSSDKTHTCPPCPAPELLGGPSVFLFPPKPKDTLMISRTPEVTCVVVDVSHEDPEVKFNWYVDGV
EVHNAKTKPREEQYNSTYRVVSVLTVLHQDWLNGKEYKCKVSNKALPAPIEKTISKAKGQPREPQVY
TLPPSRDELTKNQVSLTCLVKGFYPSDIAVEWESNGQPENNYKTTPPVLDSDGSFFLYSKLTVDKSR
WQQGNVFSCSVMHEALHNHYTQKSLSLSPGKGSSGGLNDIFEAQKIEWHE

Protein production and purification:

The protein was produced in CHO cells as described in **General Methods**.

Quality control of Fc production (SEC)

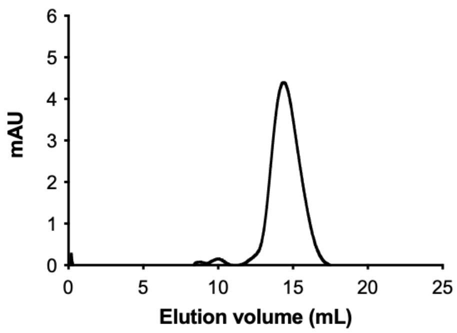

Protein biotinylation:

The protein was site-specific biotinylated as described in **General Methods**.

Quality control of Fc biotinylation (SDS-PAGE)

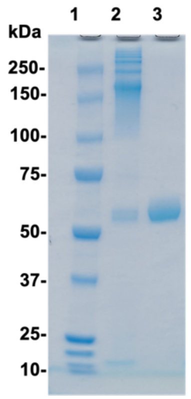

1 = molecular weight marker

2 = non-reduced biotinylated Fc + avidin

3 = non-reduced biotinylated Fc

Buffer used for selections: PBS (pH 7.4)

Molecular weight: 28.2 kDa (monomer)

ε (280 nm), $M^{-1}cm^{-1}$: 40´910

5 Compound synthesis

5.1 On-DNA ACP3 hit re-synthesis

On-DNA hit resynthesis was performed with a short 5'-amino-modified oligonucleotide. Scaffold synthesis and the derivatizations with corresponding building blocks were performed following synthetic and purification protocols reported for library construction. The stereoisomers for each series were reacted, purified, and characterized by LC-MS separately.

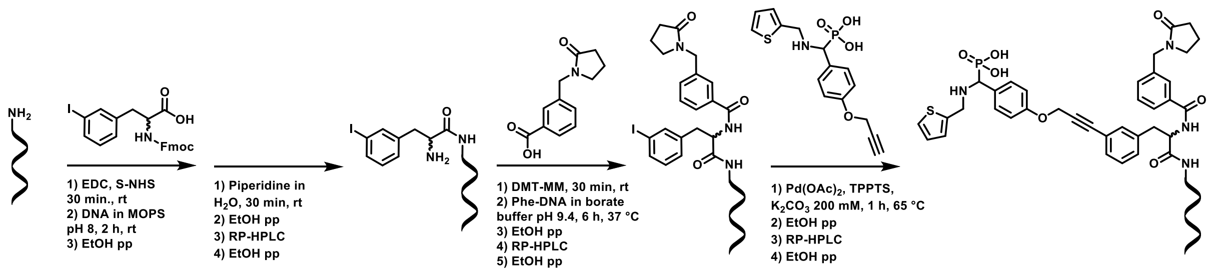

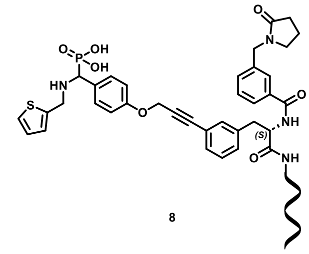

8

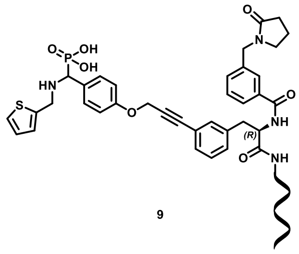

9

Figure S11. *On-DNA synthesis of conjugates* ***8*** *and* ***9****.*

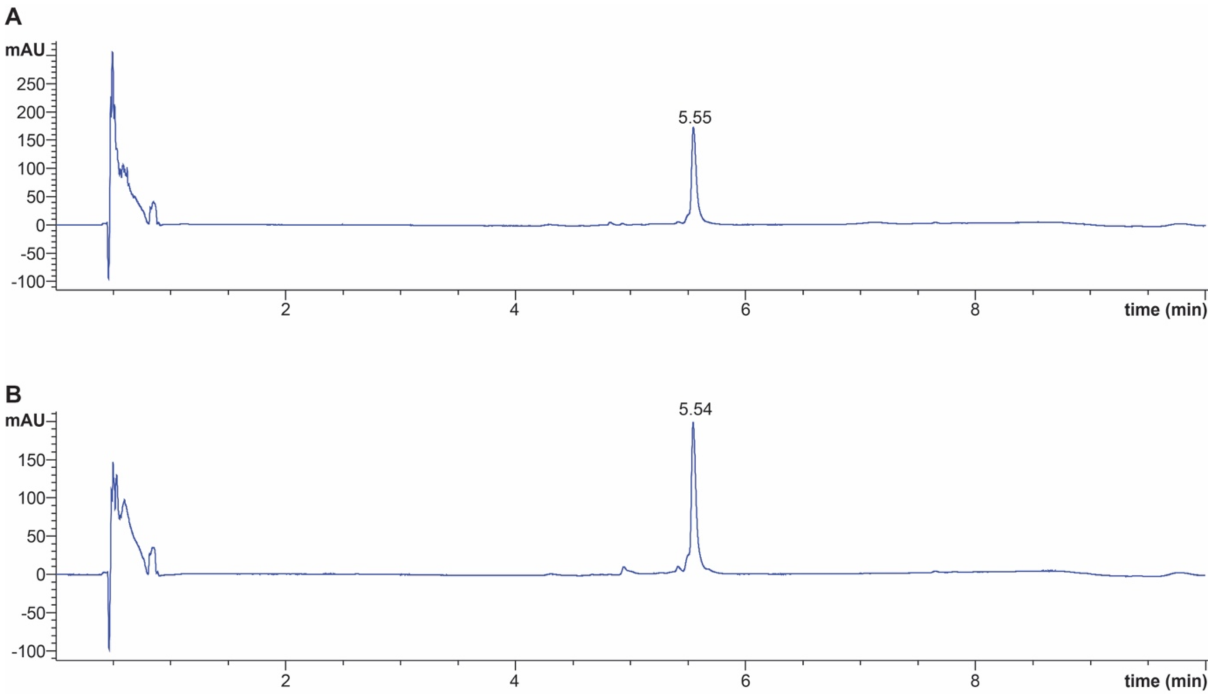

Figure S12. *Analytical LC chromatograms (mAU at λ = 260 nm) of the purified DNA conjugates (**A**) **8**, and (**B**) **9**. Method A.*

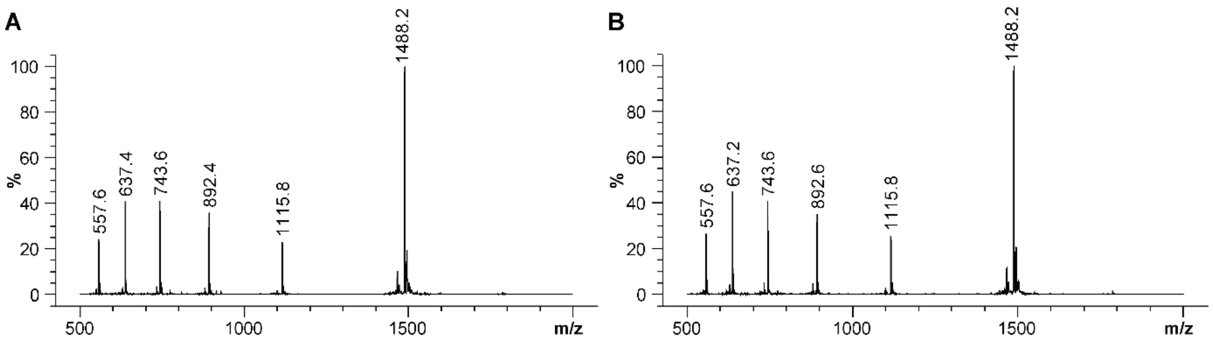

Figure S13. *Mass spectrum of (**A**) **8**, and (**B**) **9** synthesized on amino-modified a 12-mer oligonucleotide (5' C_6-amino-TAGTAGCCATCC). Deconvolution consistently resulted in an observed mass of 4467.7 Da (calculated mass: 4467.2 Da).*

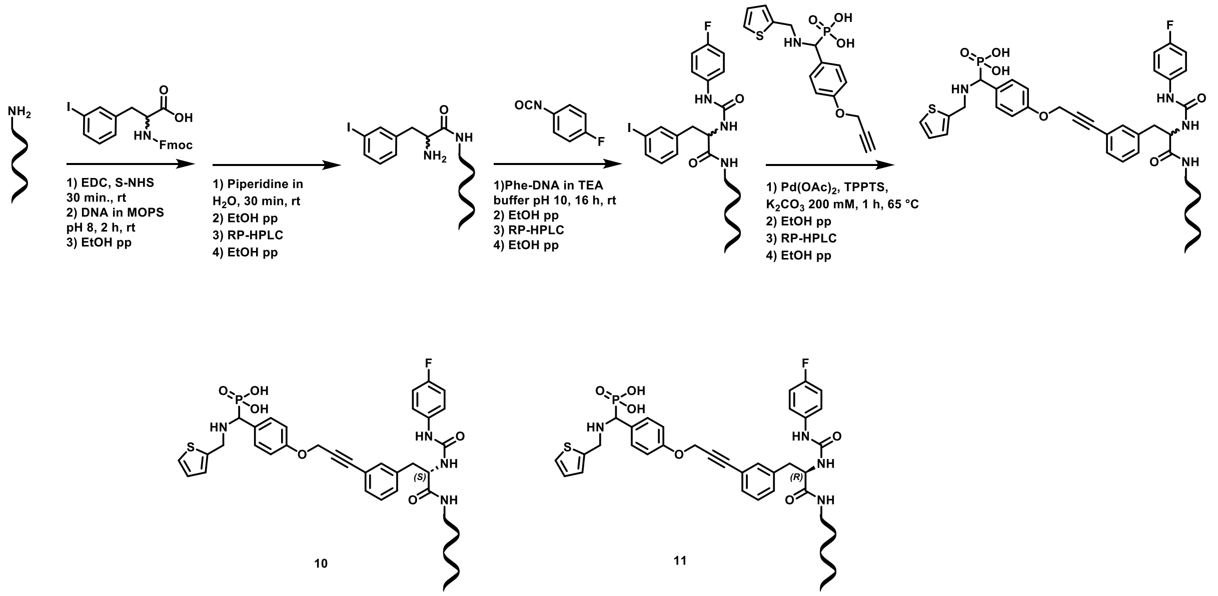

Figure S14. *On-DNA synthesis of conjugates **10** and **11**.*

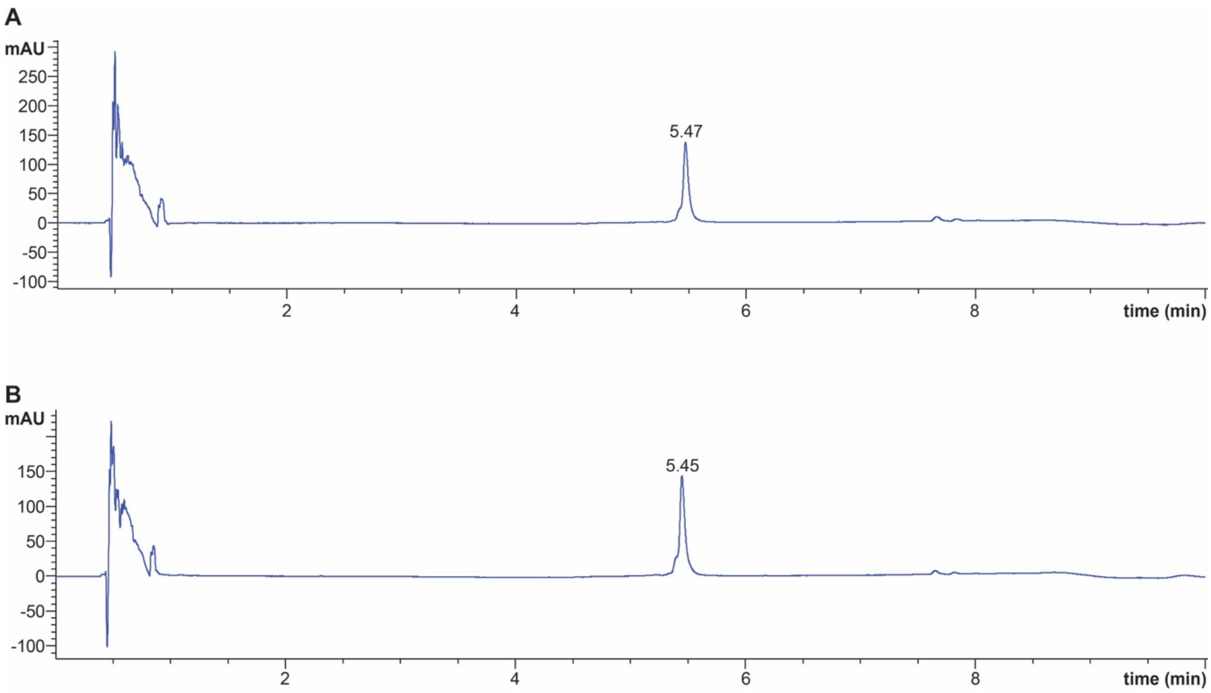

Figure S15. *Analytical LC chromatograms (mAU at λ = 260 nm) of the purified DNA conjugates (**A**) **10**, and (**B**) **11**. Method A.*

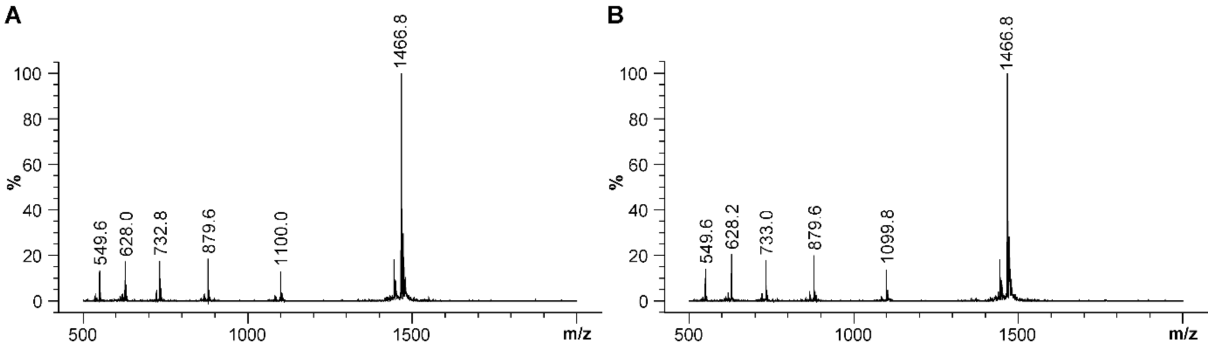

Figure S16. *Mass spectrum of (**A**) **10**, and (**B**) **11** synthesized on amino-modified a 12-mer oligonucleotide (5' C_6-amino-TAGTAGCCATCC). Deconvolution consistently resulted in an observed mass of 4403.6 (calculated mass: 4403.1 Da).*

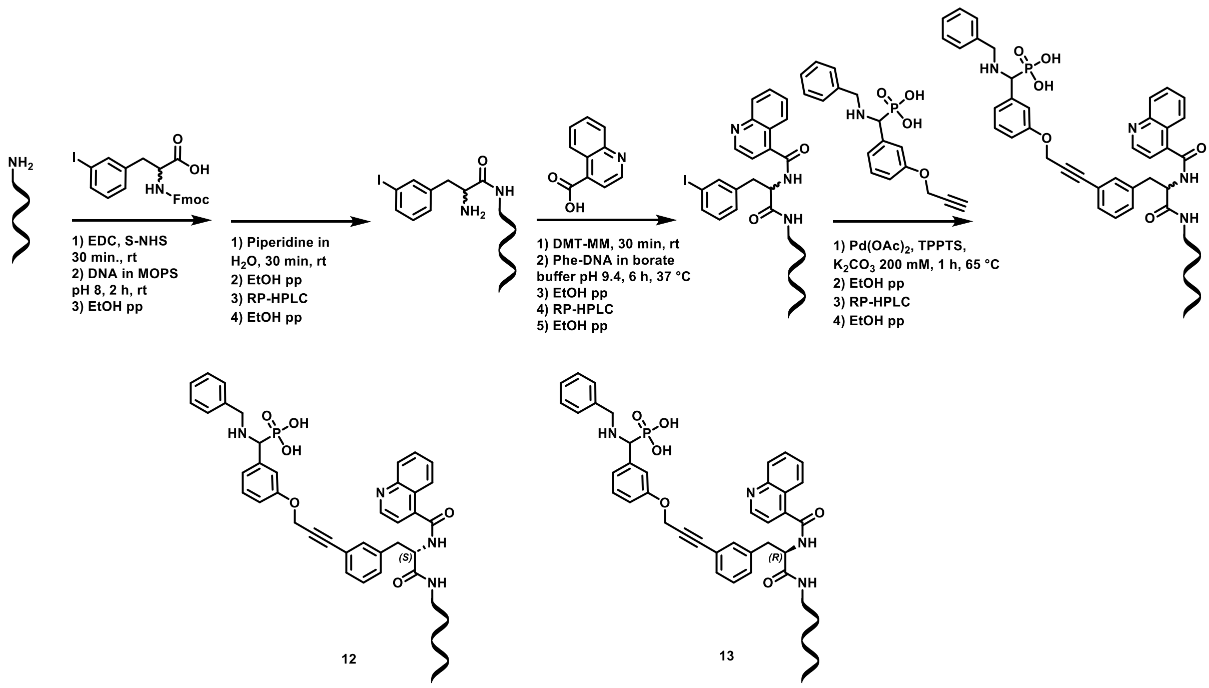

Figure S17. *On-DNA synthesis of conjugates **12** and **13**.*

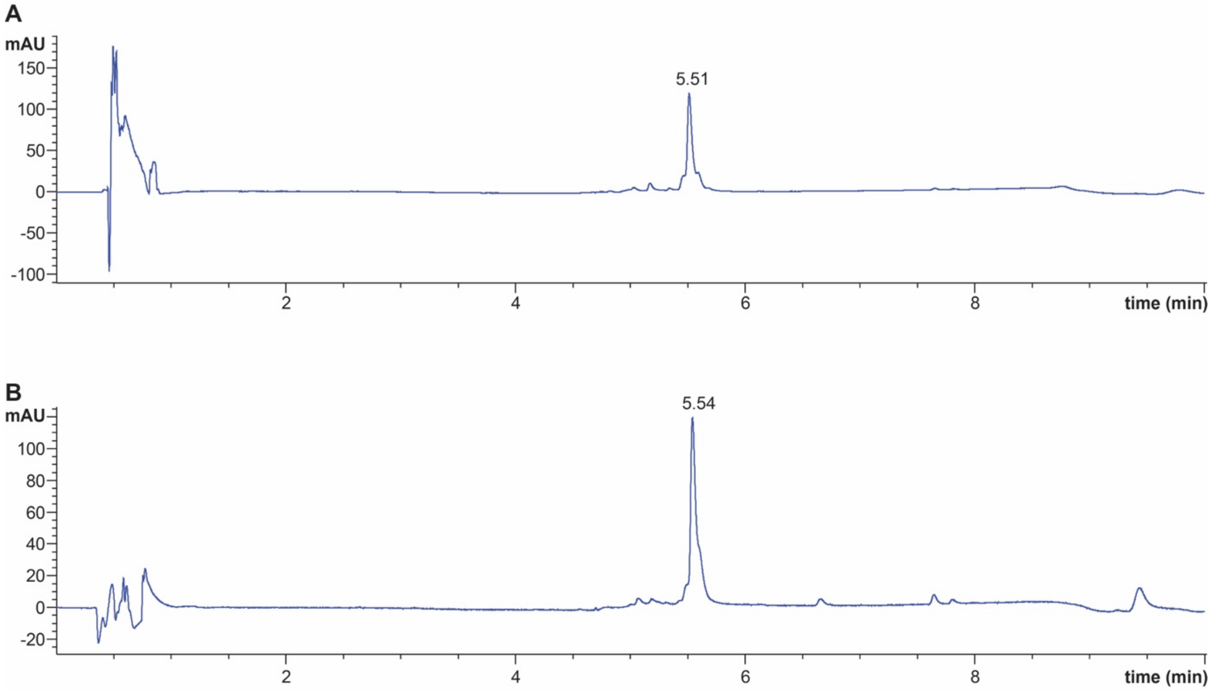

Figure S18. *Analytical LC chromatograms (mAU at λ = 260 nm) of the purified DNA conjugates (**A**) **12**, and (**B**) **13**. Method A.*

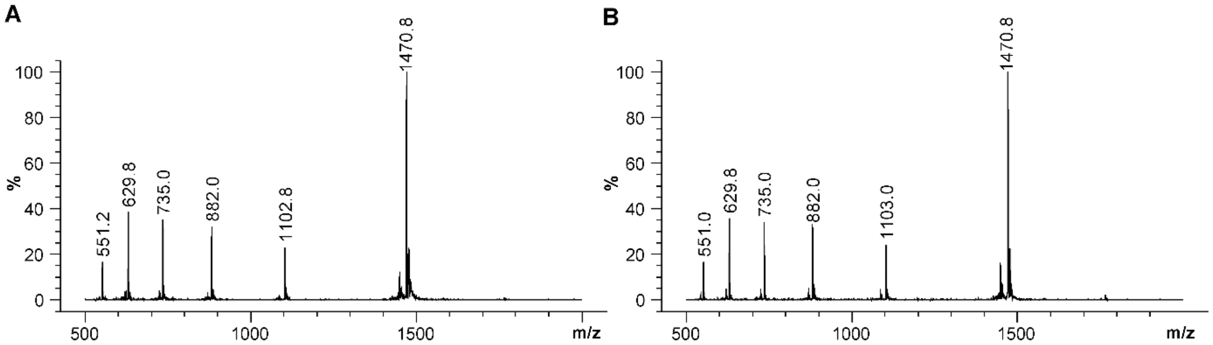

Figure S19. *Mass spectrum of (**A**) **12**, and (**B**) **13** synthesized on amino-modified a 12-mer oligonucleotide (5' C_6-amino-TAGTAGCCATCC). Deconvolution consistently resulted in an observed mass of 4415.5 Da (calculated mass: 4415.2 Da).*

5.2 General synthetic procedures for small molecules

*General solid-phase coupling reaction (**GP1**)*

Solid-phase synthesis was performed with Wang resin (100-200 mesh, 1.1 mmol/g) following standard Solid-Phase Peptide Synthesis (SPPS) protocols (Fmoc strategy). The resin was swollen for 15 min in DMF before any reaction steps. The carboxylic acids (3.00 equiv.), HATU (3.00 equiv.) and DIPEA (6.00 equiv.) were dissolved in DMF (0.1 M) and added to the resin. Incubations were performed in 10 mL reaction columns on a rotator mixer at rt for 2 h. After incubation, the resin was subsequently washed five times with DMF. Coupling efficiency was monitored by LC-MS after mini cleavage.

*Fmoc deprotection (**GP2**)*

Resin was incubated twice (15 min) with 20% piperidine in DMF at rt for 15 min. After deprotection, the resin was washed 5 times with DMF to remove residual piperidine. Deprotection efficiency was monitored by LC-MS after mini cleavage.

*Mini cleavage test for LC-MS analysis (**GP3**)*

A small portion of resin was transferred to an Eppendorf tube and incubated with 40 μL TFA for 15 min at rt. The cleavage was quenched by addition of 80 μL DMF to centrifuge the suspension (1 min at 10´000 rcf) before LC-MS analysis. This method was used to monitor the synthesis after each reaction step on resin.

*Resin cleavage and purification (**GP4**)*

Cleavage solution was prepared as follows: 95% TFA, 2.5% water, and 2.5% triisopropylsilane (TIPS). Two consecutive cleavage steps (1 h at rt each step) were performed. Cleavage fractions were combined, peptides were precipitated by adding 5-10 volumes of ice-cold diethyl ether after most of the TFA was removed under reduced pressure. Precipitation proceeded for 30 min at -20 °C to obtain the peptide as a pellet by centrifugation (3´200 rcf, 5 min, 4 °C).

*Compound functionalization with DOTAGA or (R)-DOTAGA-NHS (**GP5**)*

Amine (1.00 equiv.) and DOTAGA anhydride or (*R*)-DOTAGA-NHS (1.00 equiv.) were dissolved in DMSO (0.01 M). DIPEA (3.00 equiv.) was added, and the reaction was incubated at 40 °C for 1 h. The crude mixture was diluted with DMSO (3 x volume) for purification *via* RP-HPLC.

*Compound functionalization with fluorescein isothiocyanate (FITC) (**GP6**)*

Amine (1.00 equiv.) and fluorescein isothiocyanate (1.00 equiv.) were dissolved in DMSO (0.01 M). DIPEA (3.00 equiv.) was added, and the reaction was incubated at 40 °C for 1 h. The crude mixture was diluted with DMSO (3 x volume) for purification *via* RP-HPLC.

*Radiolabeling of (R)-DOTAGA compounds with Lutetium-177 (**GP7**)*

(*R*)-DOTAGA derivatives (15 nmol) were dissolved in 15 μL of PBS 4% DMSO and diluted with sodium acetate (100 μL, 1 M in water, pH 4.5). 50 MBq of $^{177}LuCl_3$ solution (25 μL at an activity of 2 MBq/μL) were added and the mixture was heated at 90 °C for 10 min and passively cooled down to rt for 10 min. After cooling to room temperature, an aliquot was analyzed by RP-HPLC (XTerra C18, 5% MeCN in 0.1% aq. TFA to 80% over 20 min on a Merck-Hitachi D-7000 HPLC system equipped with a Raytest Gabi Star radiodetector). ^{177}Lu incorporations > 95% were routinely achieved.

5.3 Chemical synthesis of hit compounds, and intermediates

*5.3.1 Synthesis of PSMA hit compounds **1-7***

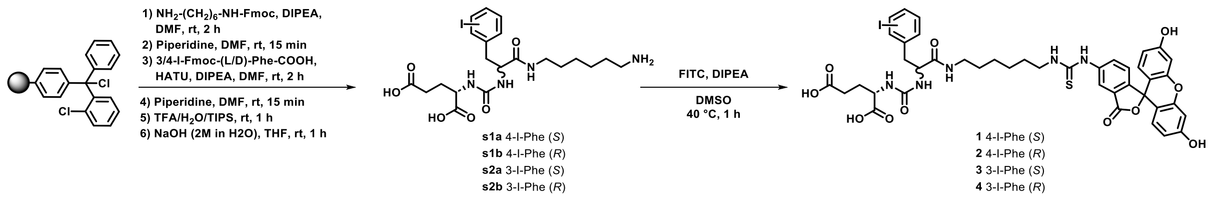

In four separated reaction columns equipped with a filter, commercially available 2-chlortrityl chloride resin (1.00 g, 0.19 mmol, 1.00 equiv.) was swollen in DMF (5 mL) for 15 min. A solution of DIPEA (133.15 µL, 0.76 mmol, 4.00 equiv.) and (9H-fluoren-9-yl)methyl (6-aminohexyl)carbamate (192.66 mg, 0.57 mmol, 3.00 eq) in DMF (5 mL) were added to each column and reacted at rt for 2 h. The resin was washed with DMF (3 x 10 mL) and Fmoc deprotected according to **GP2.** In each column was added a solution of DIPEA (199.73 µL, 1.14 mmol, 6.00 equiv.), HATU (216.60 mg, 0.57 mmol, 3.00 equiv.), and the corresponding isomer of the Fmoc-*iodo*-phenyl alanine (292.41 mg, 0.57 mmol, 3.00 equiv.) in DMF (5 mL) according to **GP1** and Fmoc deprotected according to **GP2**. After washing steps in DMF and DCM, the resins were cleaved according to **GP4**. The crudes were separately dissolved in THF (2 mL) and an aqueous solution of NaOH 2 M (0.48 mL, 0.95 mmol, 5.00 equiv.) was added. Reaction left stirred for 1 h and then solvents evaporated under reduced pressure. Crude products were purified *via* preparative RP-HPLC (Method B). After lyophilization, compounds **s1a,b** and **s2a,b** were obtained as white solids.

s1a 4-I-Phe (*S*): 50.67 mg, 0.09 mmol, 48% yield.

^{1}H NMR (400 MHz, DMSO-d_6): δ 7.98 - 7.88 (m, 4H), 7.58 (d, J = 8.1 Hz, 2H), 6.97 (d, J = 8.1 Hz, 2H), 6.50 (d, J = 8.2 Hz, 1H), 6.30 (d, J = 8.2 Hz, 1H), 4.33 - 4.23 (m, 1H), 4.11 - 4.02 (m, 1H), 3.11 - 2.90 (m, 2H), 2.88 - 2.68 (m, 5H), 2.30 - 2.14 (m, 2H), 1.95 - 1.84 (m, 1H), 1.76 - 1.60 (m, 1H), 1.52 (p, J = 7.5 Hz, 2H), 1.38 - 1.21 (m, 5H), 1.20 - 1.18 (m, 2H).

HRMS (ESI, m/z) calcd. for $C_{21}H_{31}IN_4O_6$: [M + H]$^+$ 563.13610, detected: [M + H]$^+$ 563.13580.

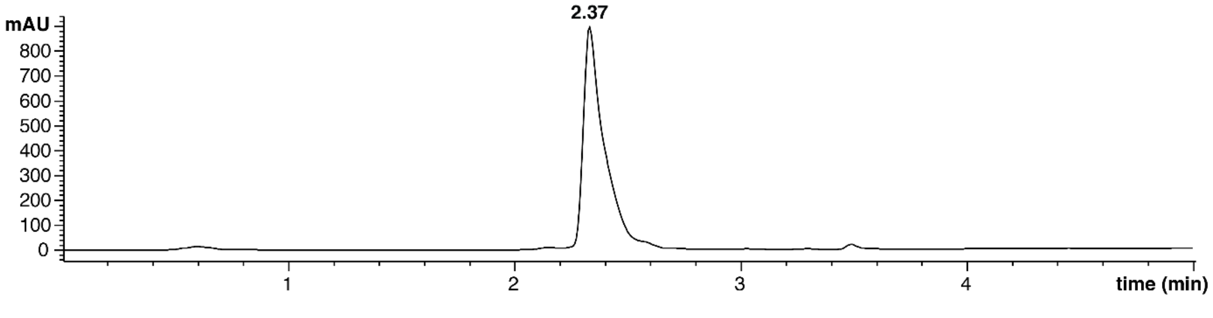

LC chromatogram of compound **s1a** (mAU at λ = 260 nm). Method D.

s1b 4-I-Phe (*R*): 56.71 mg, 0.10 mmol, 53% yield.

^{1}H NMR (400 MHz, DMSO-d_6) δ 7.96 - 7.84 (m, 4H), 7.55 (d, *J* = 8.1 Hz, 2H), 6.97 (d, *J* = 8.1 Hz, 2H), 6.49 (d, *J* = 8.4 Hz, 1H), 6.28 (d, *J* = 8.1 Hz, 1H), 4.33 - 4.23 (m, 1H), 4.11 - 4.02 (m, 1H), 3.11 - 2.90 (m, 2H), 2.88 - 2.68 (m, 5H), 2.30 - 2.14 (m, 2H), 1.95 - 1.84 (m, 1H), 1.76 - 1.60 (m, 1H), 1.54 (p, *J* = 7.6 Hz, 2H), 1.38 - 1.21 (m, 5H), 1.20 - 1.18 (m, 2H).

HRMS (ESI, m/z) calcd. for $C_{21}H_{31}IN_4O_6$: $[M + H]^+$ 563.13610, detected: $[M + H]^+$ 563.13610.

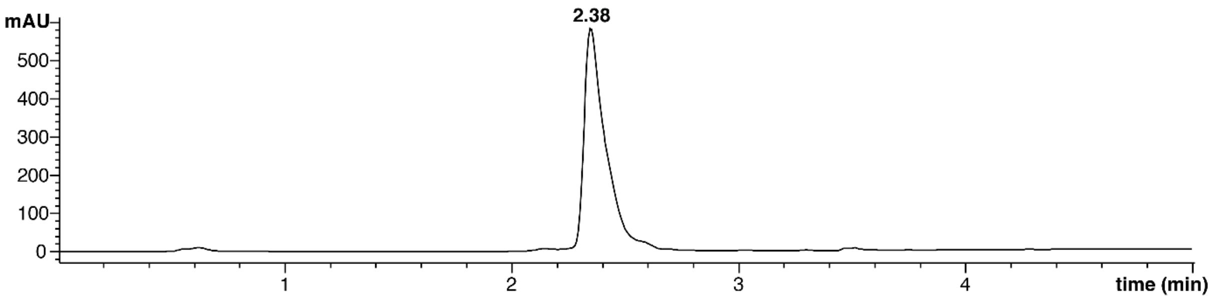

LC chromatogram of compound **s1b** (mAU at λ = 260 nm). Method D.

s2a 3-I-Phe (*S*): 41.72 mg, 0.07 mmol, 39% yield.

^{1}H NMR (400 MHz, DMSO-d_6): δ 7.93 (t, *J* = 5.7 Hz 1H), 7.77 (bs, 3H), 7.69 - 7.50 (m, 2H), 7.17 (tt, *J* = 7.7, 1.3 Hz, 1H), 7.07 (td, *J* = 7.8, 2.3 Hz, 1H), 6.47 (dd, *J* = 8.3, 2.9 Hz, 1H), 6.26 (d, *J* = 8.3 Hz, 1H), 4.35 - 4.21 (m, 1H), 4.12 - 4.01 (m, 1H), 3.11 - 3.03 (m, 1H), 2.97 - 2.86 (m, 1H), 2.85 - 2.66 (m, 4H), 2.29 - 2.10 (m, 2H), 1.95 - 1.84 (m, 1H), 1.76 - 1.62 (m, 1H), 1.50 (quin, *J* = 7.5 Hz, 2H), 1.38 - 1.22 (m, 4H), 1.17 (q, *J* = 7.1 Hz, 2H).

HRMS (ESI, m/z) calcd. for $C_{21}H_{31}IN_4O_6$: $[M + H]^+$ 563.13610, detected: $[M + H]^+$ 563.13569.

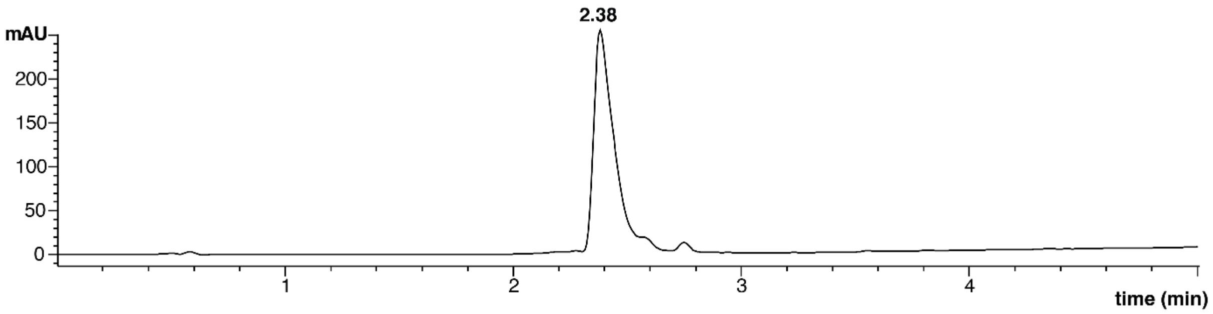

LC chromatogram of compound **s2a** (mAU at λ = 260 nm). Method D.

s2b 3-I-Phe (*R*): 56.83 mg, 0.10 mmol, 55% yield.

^{1}H NMR (400 MHz, DMSO-d_6): δ 7.93 (t, *J* = 5.6 Hz 1H), 7.77 (bs, 3H), 7.59 - 7.49 (m, 2H), 7.17 (tt, *J* = 7.8, 1.4 Hz, 1H), 7.06 (td, *J* = 7.7, 2.2 Hz, 1H), 6.47 (dd, *J* = 8.3, 2.9 Hz, 1H), 6.27 (d, *J* = 8.3 Hz, 1H), 4.33 - 4.22 (m, 1H), 4.11 - 4.02 (m, 1H), 3.12 - 3.02 (m, 1H), 2.99 - 2.88 (m, 1H), 2.86 - 2.67 (m, 4H), 2.30 - 2.11 (m, 2H), 1.95 - 1.84 (m, 1H), 1.76 - 1.62 (m, 1H), 1.50 (quin, *J* = 7.5 Hz, 2H), 1.38 - 1.22 (m, 4H), 1.17 (q, *J* = 7.1 Hz, 2H).

HRMS (ESI, m/z) calcd. for $C_{21}H_{31}IN_4O_6$: $[M + H]^+$ 563.13610, detected: $[M + H]^+$ 563.13611.

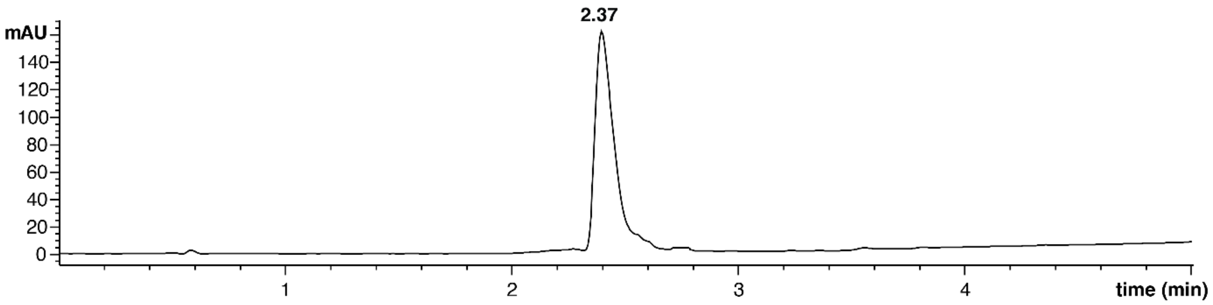

LC chromatogram of compound **s2b** (mAU at λ = 260 nm). Method D.

Compounds **1**-**4** were prepared *via* **GP6** (3.00 *μ*mol scale) from the respective amines **s1a,b** and **s2a,b**. The final compounds were purified *via* RP-HPLC (Method C) and obtained as red solid after lyophilization.

1 4-I-Phe (*S*): 1.51 mg, 1.59 µmol, 53% yield.

HRMS (ESI, m/z) calcd. for $C_{42}H_{52}IN_5O_{11}S$: $[M + H]^+$ 952.17189, detected: $[M + H]^+$ 952.16849.

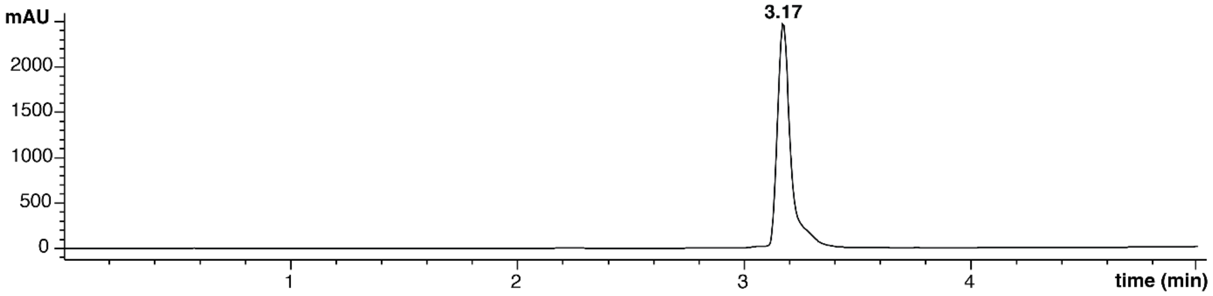

LC chromatogram of compound **1** (mAU at λ = 260 nm). Method E.

2 4-I-Phe (*R*): 1.31 mg, 1.38 µmol, 46% yield.

HRMS (ESI, m/z) calcd. for $C_{42}H_{52}IN_5O_{11}S$: $[M + H]^+$ 952.17189, detected: $[M + H]^+$ 952.16976.

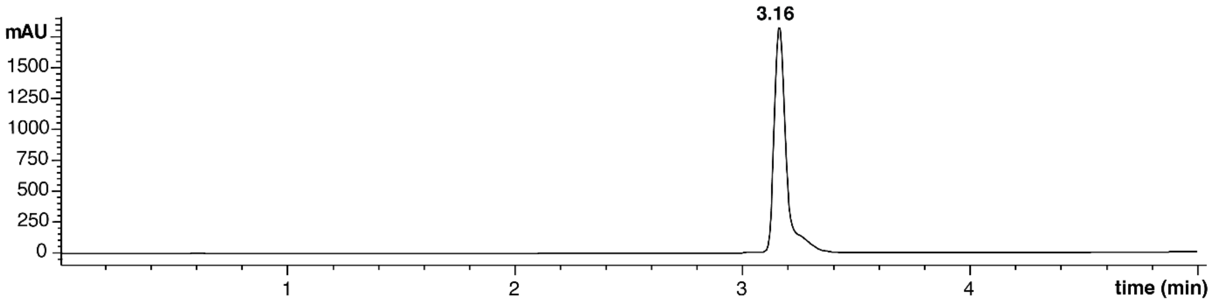

LC chromatogram of compound **2** (mAU at λ = 260 nm). Method E.

3 3-I-Phe (*S*): 1.45 mg, 1.53 µmol, 51% yield.

HRMS (ESI, m/z) calcd. for $C_{42}H_{52}IN_5O_{11}S$: $[M + H]^+$ 952.17189, detected: $[M + H]^+$ 952.16887.

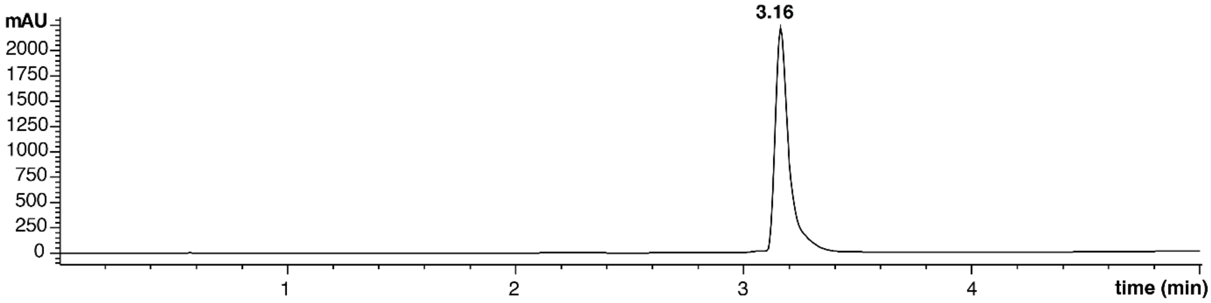

LC chromatogram of compound **3** (mAU at λ = 260 nm). Method E.

4 3-I-Phe (*R*): 1.31 mg, 1.38 µmol, 46% yield.

HRMS (ESI, m/z) calcd. for $C_{42}H_{52}IN_5O_{11}S$: $[M + H]^+$ 952.17189, detected: $[M + H]^+$ 952.16887.

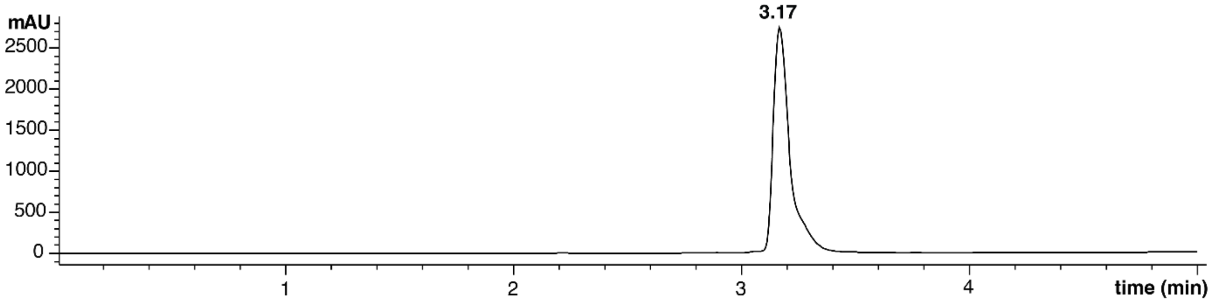

LC chromatogram of compound **4** (mAU at λ = 260 nm). Method E.

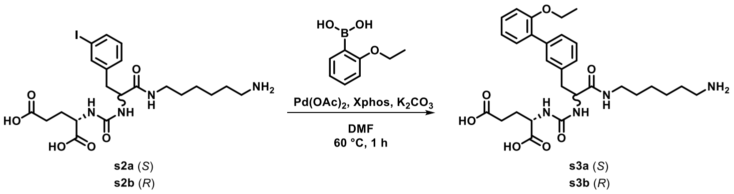

Compounds **s2a** and **s2b** (20.00 mg, 35.58 *µ*mol, 1.00 equiv.) were separately loaded in two reaction vessels and suspended in dry DMF (2 mL). K_2CO_3 (19.67 mg, 142.35 *µ*mol, 4.00 equiv), Palladium(II) acetate ($Pd(OAc)_2$, 1.59 mg, 7.12 *µ*mol, 0.2 equiv), dicyclohexyl(2',4',6'-triisopropyl-[1,1'-biphenyl]-2-yl)phosphane (XPhos, 6.79 mg, 14.23 *µ*mol, 0.4 equiv) and (2-ethoxyphenyl)boronic acid (8.85 mg, 53.37 *µ*mol, 1.50 equiv.) were added. Reactions left stirred for 1 h at 60 °C. After this time mixtures were separately diluted with EtOAc (5 mL) and washed with water (3 x 10 mL). The organic layers were dried over anhydrous sodium sulfate, filtered, and concentrated under reduced pressure.

The final compounds were purified *via* RP-HPLC (Method B) and obtained pale-yellow solids after lyophilization.

s3a (*S*): 13.85 mg, 24.91 *µ*mol, 70% yield.

^{1}H NMR (400 MHz, DMSO-d_6) δ 7.91 (q, *J* = 5.6 Hz, 1H), 7.82 - 7.73 (m, 2H), 7.45 - 7.20 (m, 6H), 7.14 - 7.04 (m, 2H), 7.00 (t, *J* = 7.4 Hz, 1H), 6.48 (dd, *J* = 8.2, 2.6 Hz, 1H), 6.30 (dd, *J* = 23.7, 8.2 Hz, 1H), 4.33 (dq, *J* = 14.4, 7.2 Hz, 1H), 4.06 (dq, *J* = 29.1, 7.4, 6.9 Hz, 3H), 3.64 - 3.52 (m, 2H), 3.48 (s,

1H), 3.11 - 3.00 (m, 1H), 3.00 - 2.85 (m, 3H), 2.85 - 2.72 (m, 3H), 2.31 - 2.11 (m, 3H), 1.95 - 1.80 (m, 1H), 1.80 - 1.60 (m, 2H), 1.56 - 1.42 (m, 3H), 1.39 - 1.10 (m, 12H).

HRMS (ESI, m/z) calcd. for $C_{27}H_{40}N_4O_7$: $[M + H]^+$ 557.29698, detected: $[M + H]^+$ 557.29654.

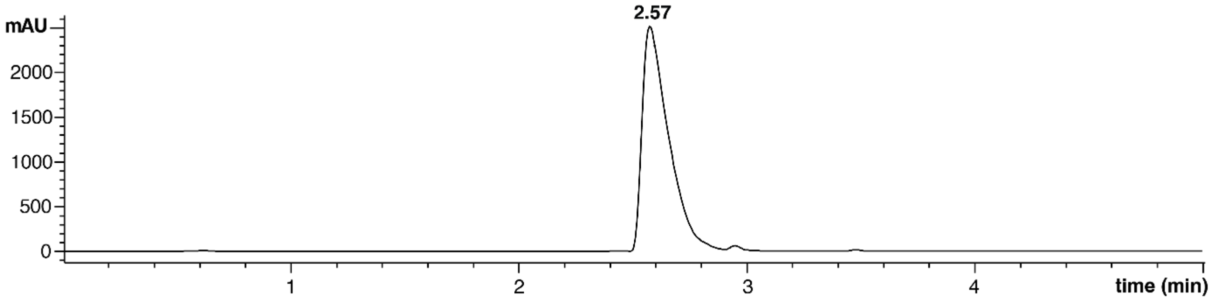

LC chromatogram of compound **s3a** (mAU at λ = 260 nm). Method D.

s3b (*R*): 15.23 mg, 37.30 μmol, 77% yield.

^{1}H NMR (400 MHz, DMSO-d_6) δ 7.93 (q, *J* = 5.6 Hz, 1H), 7.82 - 7.73 (m, 2H), 7.48 - 7.22 (m, 6H), 7.14 - 7.04 (m, 2H), 7.00 (t, *J* = 7.4 Hz, 1H), 6.49 (dd, *J* = 8.2, 2.6 Hz, 1H), 6.30 (dd, *J* = 23.7, 8.2 Hz, 1H), 4.35 (dq, *J* = 14.6, 7.8 Hz, 1H), 4.06 (dq, *J* = 29.1, 7.4, 6.9 Hz, 3H), 3.64 - 3.52 (m, 2H), 3.48 (s, 1H), 3.11 - 3.00 (m, 1H), 3.00 - 2.85 (m, 3H), 2.85 - 2.72 (m, 3H), 2.34 - 2.11 (m, 3H), 1.95 - 1.80 (m, 1H), 1.80 - 1.60 (m, 2H), 1.56 - 1.42 (m, 3H), 1.39 - 1.10 (m, 12H).

HRMS (ESI, m/z). calcd. for $C_{27}H_{40}N_4O_7$: $[M + H]^+$ 557.29698, detected: $[M + H]^+$ 557.29660.

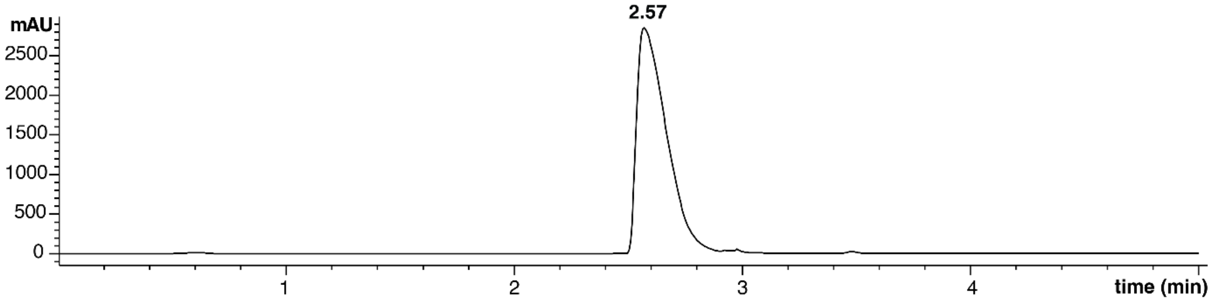

LC chromatogram of compound **s3b** (mAU at λ = 260 nm). Method D.

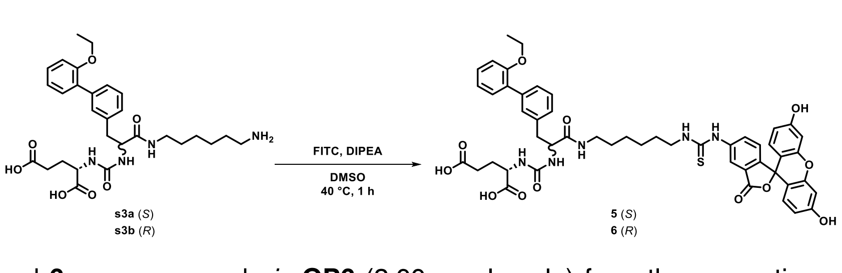

Compounds **5** and **6** were prepared *via* **GP6** (2.00 μmol scale) from the respective amines **s3a** and **s3b**. The final compounds were purified *via* RP-HPLC (Method C) and obtained as red solid after lyophilization.

5 (*S*): 1.01 mg, 1.06 μmol, 53% yield.

HRMS (ESI, m/z) calcd. for $C_{50}H_{51}N_5O_{12}S$: $[M + H]^+$ 946.33277, detected: $[M + H]^+$ 946.32936.

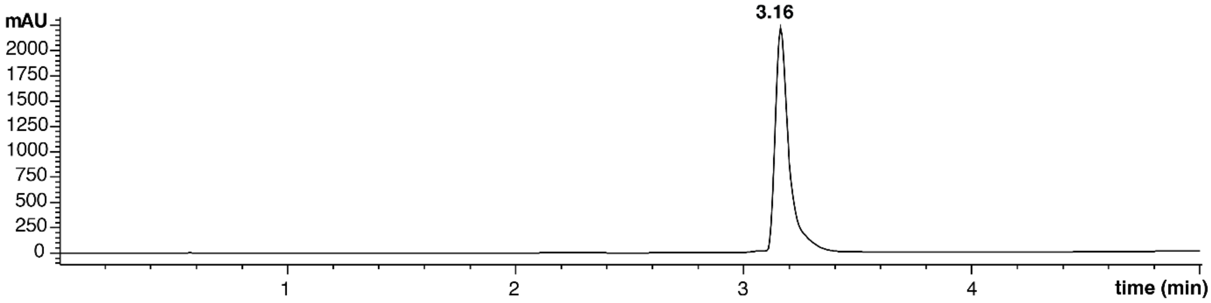

LC chromatogram of compound **5** (mAU at λ = 260 nm). Method E.

6 (*R*): 1.19 mg, 1.26 *µ*mol, 63% yield.

HRMS (ESI, m/z) calcd. for $C_{50}H_{51}N_5O_{12}S$: $[M + H]^+$ 946.33277, detected: $[M + H]^+$ 946.32935.

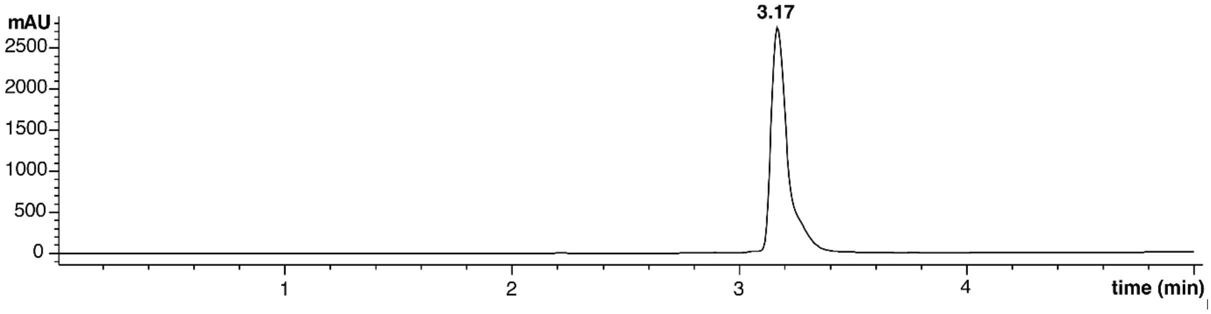

LC chromatogram of compound **6** (mAU at λ = 260 nm). Method E.

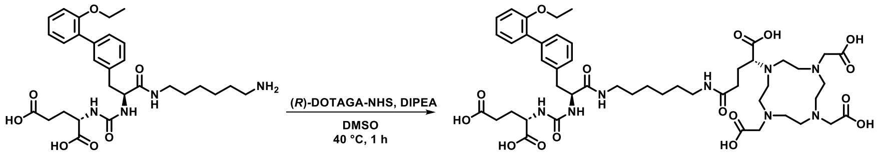

s3a 7

Compound **7** was prepared *via* **GP5** (10.00 *µ*mol scale) from the respective amine **s3a**. The final compound was purified *via* RP-HPLC (Method C) and obtained as white solid after lyophilization.

7: 5.79 mg, 5.70 *µ*mol, 57% yield.

^{1}H NMR (400 MHz, DMSO-d_6) δ 7.89 (q, *J* = 5.6 Hz, 1H), 7.82 (t, *J* = 5.6 Hz, 1H), 7.44 - 7.34 (m, 1H), 7.34 - 7.22 (m, 4H), 7.14 - 7.04 (m, 2H), 7.00 (td, *J* = 7.4, 1.0 Hz, 1H), 6.47 (dd, *J* = 8.2, 3.7 Hz, 1H), 6.28 (dd, *J* = 24.5, 8.3 Hz, 1H), 4.33 (sextet, *J* = 14.3, 1H), 4.11 - 3.96 (m, 4H), 3.83 (s, 1H), 3.31 - 2.74 (m, 17H), 2.35 (bs, 1H), 2.29 - 2.11 (m, 2H), 1.98 - 1.80 (m, 3H), 1.76 - 1.61 (m, *J* = 14.0, 8.3, 5.5 Hz, 1H), 1.38 - 1.11 (m, 11H).

HRMS (ESI, m/z) calcd. for $C_{48}H_{70}N_8O_{16}$: $[M + H]^+$ 1015.49826, detected: $[M + H]^+$ 1015.49574.

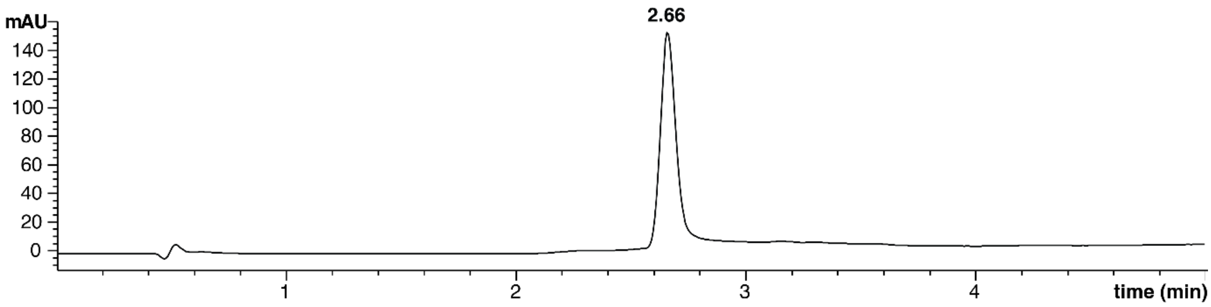

LC chromatogram of compound **7** (mAU at λ = 260 nm). Method D.

*5.3.2 Synthesis of ACP3 hit compounds **14-17***

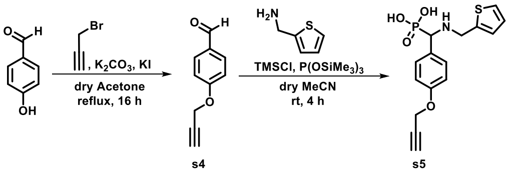

4-hydroxybenzaldehyde (1.00 g, 8.18 mmol, 1.00 equiv.), K_2CO_3 (4.40 g, 24.57 mmol, 3.00 equiv.), and KI (1.36 g, 8.18 mmol, 1.00 equiv.) were loaded into a round-bottom flask and suspended in dry acetone (50 mL). The solution was stirred at rt for 1 h, and propargyl bromide (80% in toluene, 1.09 mL, 9.83 mmol, 1.20 equiv) was added. The mixture was left to stir at 50 °C for 16 h. After the removal of the solvent under reduced pressure, the crude was dissolved in EtOAc (50 mL) and water (30 mL) and trasnferred into a separatory funnel. The organic phase was washed with water (2 x 30 mL) and brine (20 mL), dried over anhydrous sodium sulphate, filtered and concentrated under reduced pressure. The crude was directly used for the next step without further purification.

s4: 1.11 g, 6.95 mmol, 85% yield.
^{1}**H NMR** (400 MHz, DMSO-*d*$_6$) δ 9.88 (s, 1H), 7.94 - 7.85 (m, 2H), 7.22 - 7.14 (m, 2H), 4.94 (d, *J* = 2.4 Hz, 2H), 3.64 (t, *J* = 2.4 Hz, 1H).
HRMS (ESI, m/z) calcd. for $C_{10}H_8O_2$: $[M + H]^+$ 161.05971, detected: $[M + H]^+$ 165.05979.

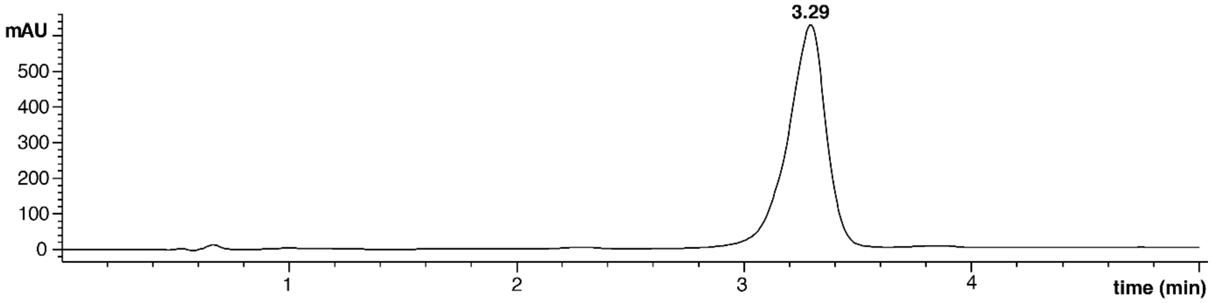

LC chromatogram of compound **s4** (mAU at λ = 260 nm). Method E.

Intermediate **s4** (500.00 mg, 3.11 mmol, 1.00 equiv.) and benzylamine (339.69 μL, 3.11 mmol, 1.00 equiv.) was dissolved in dry acetonitrile (50 mL) within a reaction vessel. Chlorotrimethylsilane

(789.42μL, 6.22 mmol, 2.00 equiv.) was added dropwise, and the cloudy solution was sonicated for 2 h at rt. After this time, tris(trimethylsilyl) phosphite [$P(OSiMe_3)_3$] (399.89 μL, 4.67 mmol, 1.5 equiv.) and chlorotrimethylsilane (789.42μL, 6.22 mmol, 2.00 equiv.) were added, and the mixture was sonicated for an additional 2 h at rt. The solvents were removed under reduced pressure, the residue was diluted in DMF or DMSO, and the products were purified *via* RP-HPLC (Method B)

s5: 524.03 mg, 1.56 mmol, 50% yield.

^{1}H NMR (400 MHz, DMSO-d_6) δ 7.62 (dd, J = 5.1, 1.2 Hz, 1H), 7.45 - 7.38 (m, 2H), 7.19 (dd, J = 3.6, 1.2 Hz, 1H), 7.07 (dd, J = 5.1, 3.5 Hz, 1H), 7.01 (d, J = 8.5 Hz, 2H), 4.83 (d, J = 2.4 Hz, 2H), 4.34 (d, J = 14.2 Hz, 1H), 4.15 (dd, J = 22.5, 15.4 Hz, 2H), 3.59 (t, J = 2.3 Hz, 1H).

HRMS (ESI, m/z) calcd. for $C_{15}H_{16}NO_4PS$: $[M + H]^+$ 338.06105, detected: $[M + H]^+$ 338.06067.

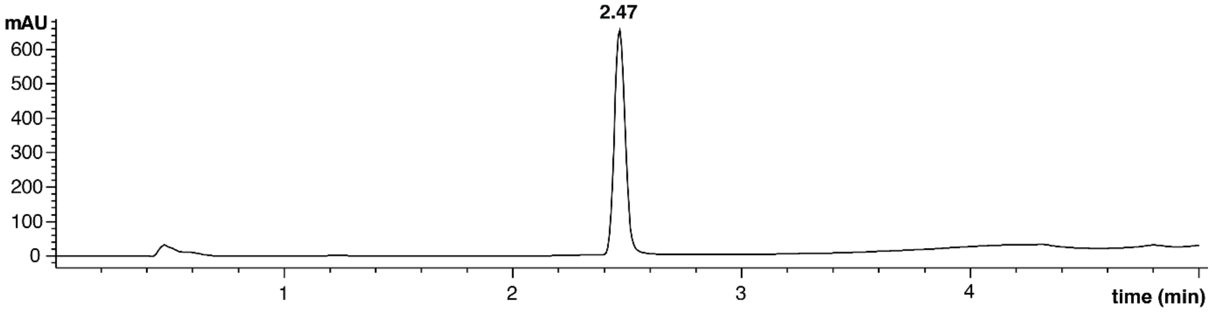

LC chromatogram of compound **s5** (mAU at λ = 260 nm). Method E.

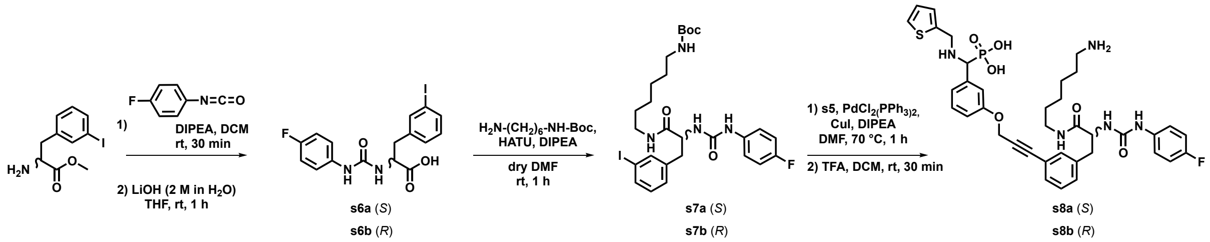

Methyl (S)-2-amino-3-(3-iodophenyl)propanoate or methyl (R)-2-amino-3-(3-iodophenyl)propanoate (500 mg, 1.64 mmol, 1.0 equiv.) were loaded into the respective reaction vessel and dissolved in DMF (5 mL). 4-Fluorophenyl isocyanate (225 mg, 1.64 mmol, 1.0 equiv.) and DIPEA (855 µL, 4.92 mmol, 3.0 equiv.) were added and the mixture was stirred at rt for 30 min. The reactions were concentrated under reduced pressure. The resulting residues were diluted in EtOAc (30 mL) and washed with sat. aq. sodium bicarbonate (2 x 15 mL) and brine (20 mL). Each organic layer was dried over anhydrous sodium sulfate, filtered, and concentrated under reduced pressure. The crudes were directly used for the next step, dissolved in THF (2 mL) and hydrolyzed with LiOH (1.64 mL, 2 M in water, 3.28 mmol, 2 equiv.) for 1 h at room temperature. The THF was removed under reduced pressure. The aqueous layer was further diluted with HCl 1 N (20 mL) and extracted with EtOAc (2 x 30 mL). Each organic layer was dried over anhydrous sodium sulfate, filtered, and concentrated under reduced pressure. The compounds were purified *via* RP-HPLC (Method B) to obtain the pure products as white solids after lyophilization.

s6a (*S*): 554.52 mg, 1.30 mmol, 79% yield.

^{1}H NMR (400 MHz, DMSO-d_6) δ 8.78 (s, 1H), 7.58 (dt, J = 5.9, 1.6 Hz, 2H), 7.41 - 7.32 (m, 2H), 7.24 (dt, J = 7.7, 1.4 Hz, 1H), 7.14 - 7.00 (m, 3H), 6.40 (d, J = 7.8 Hz, 1H), 4.42 (td, J = 7.6, 5.2 Hz, 1H), 3.06 (dd, J = 13.9, 5.2 Hz, 1H), 2.93 (dd, J = 13.8, 7.5 Hz, 1H).

HRMS (ESI, m/z) calcd. for $C_{16}H_{14}FIN_2O_3$: $[M + H]^+$ 429.01059, detected: $[M + H]^+$ 429.00992.

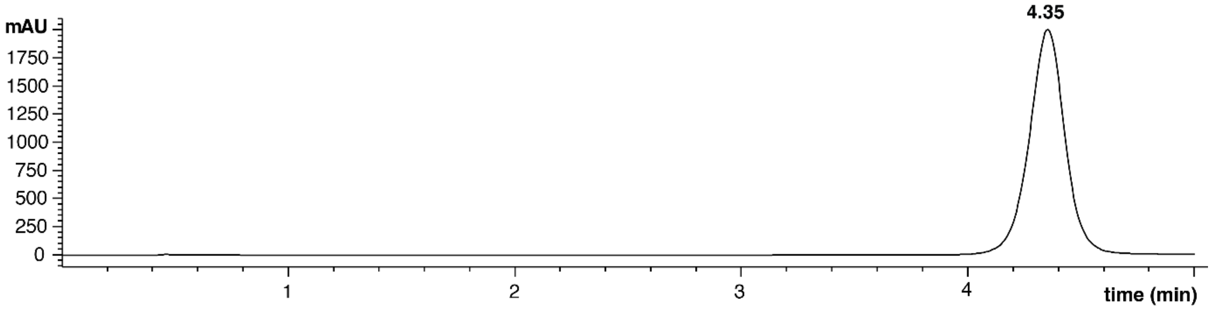

LC chromatogram of compound **s6a** (mAU at λ = 260 nm). Method D.

s6b (*R*): 540.47mg, 1.26 mmol, 77% yield.

^{1}H NMR (400 MHz, DMSO-d_6) δ 8.78 (s, 1H), 7.58 (dt, J = 5.8, 1.7 Hz, 2H), 7.41 - 7.32 (m, 2H), 7.24 (dt, J = 7.6, 1.3 Hz, 1H), 7.14 - 7.00 (m, 3H), 6.40 (d, J = 7.8 Hz, 1H), 4.42 (td, J = 7.6, 5.2 Hz, 1H), 3.06 (dd, J = 13.9, 5.2 Hz, 1H), 2.93 (dd, J = 13.8, 7.5 Hz, 1H).

HRMS (ESI, m/z) calcd. for $C_{16}H_{14}FIN_2O_3$: $[M + H]^+$ 429.01059, detected: $[M + H]^+$ 429.01013.

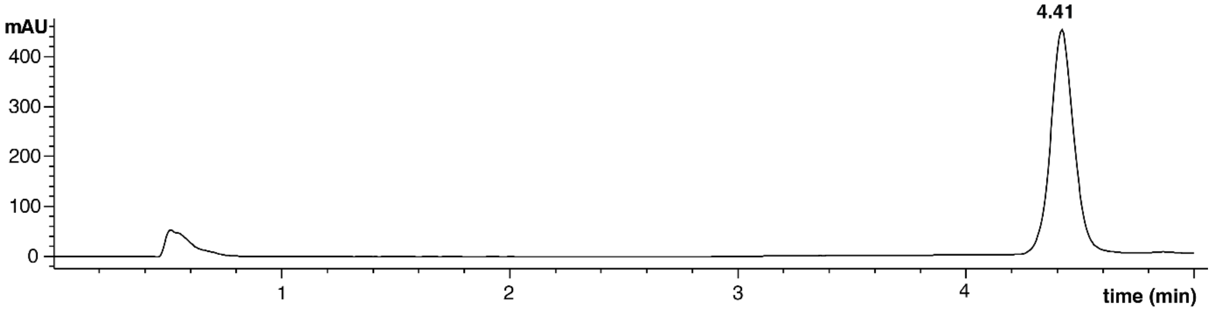

LC chromatogram of compound **s6b** (mAU at λ = 260 nm). Method D.

Intermediates **s6a,b** (500.00 mg, 1.17 mmol, 1.00 equiv.) were loaded into the respective reaction vessels, and dissolved in dry DMF (4 mL). HATU (444.16 mg, 1.17 mmol, 1.00 equiv.) and DIPEA (614.55 *µ*L, 3.51 mmol, 3.00 equiv.) were added to the round-bottom flasks and the reactions were allowed to proceed at rt for 10 min. N-Boc-1,6-hexandiamin (277.96 mg, 1.29 mmol, 1.10 equiv.) was added to both mixtures, and the reactions were stirred at rt for additional 50 min. The crudes were separately diluted with 30 mL of EtOAc and washed with water (15mL), sat. aq. ammonium chloride (2 x 15 mL), and brine (15 mL). Each organic layer was dried over anhydrous sodium sulphate, filtered, and concentrated under reduced pressure. The crude products were purified *via* RP-HPLC (Method B) to obtain **s7a,b** as white solids after lyophilization.

s7a (*S*): 75.12 mg, 0.12 mmol, 10% yield.

^{1}H NMR (400 MHz, DMSO-d_6) δ 8.69 (s, 1H), 8.05 (t, *J* = 5.6 Hz, 1H), 7.59 - 7.52 (m, 2H), 7.38 - 7.30 (m, 2H), 7.20 (d, *J* = 7.7 Hz, 1H), 7.10 - 6.98 (m, 3H), 6.75 (t, *J* = 5.8 Hz, 1H), 6.34 (d, *J* = 8.4 Hz, 1H), 4.41 (q, *J* = 7.2 Hz, 1H), 3.13 - 3.00 (m, 1H), 3.00 - 2.83 (m, 4H), 2.82 - 2.73 (m, 1H), 1.44 - 1.25 (m, 14H), 1.25 - 1.11 (m, 5H).

HRMS (ESI, m/z) calcd. for $C_{27}H_{36}FIN_4O_4$: $[M + H]^+$ 627.18380, detected: $[M + H]^+$ 627.18332.

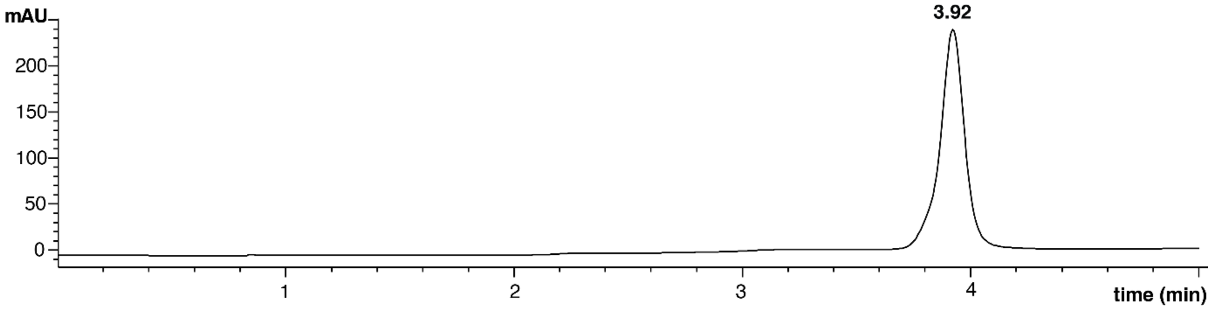

LC chromatogram of compound **s7a** (mAU at λ = 260 nm). Method E.

s7b (*S*): 87.64 mg, 0.14 mmol, 12% yield.

^{1}H NMR (400 MHz, DMSO-d_6) δ 8.69 (s, 1H), 8.05 (t, *J* = 5.6 Hz, 1H), 7.59 - 7.52 (m, 2H), 7.38 - 7.30 (m, 2H), 7.20 (d, *J* = 7.7 Hz, 1H), 7.10 - 6.98 (m, 3H), 6.75 (t, *J* = 5.8 Hz, 1H), 6.34 (d, *J* = 8.4 Hz, 1H), 4.41 (q, *J* = 7.2 Hz, 1H), 3.13 - 3.00 (m, 1H), 3.00 - 2.83 (m, 4H), 2.82 - 2.73 (m, 1H), 1.44 - 1.25 (m, 14H), 1.25 - 1.11 (m, 5H).

HRMS (ESI, m/z) calcd. for $C_{27}H_{36}FIN_4O_4$: $[M + H]^+$ 627.18380, detected: $[M + H]^+$ 627.18320.

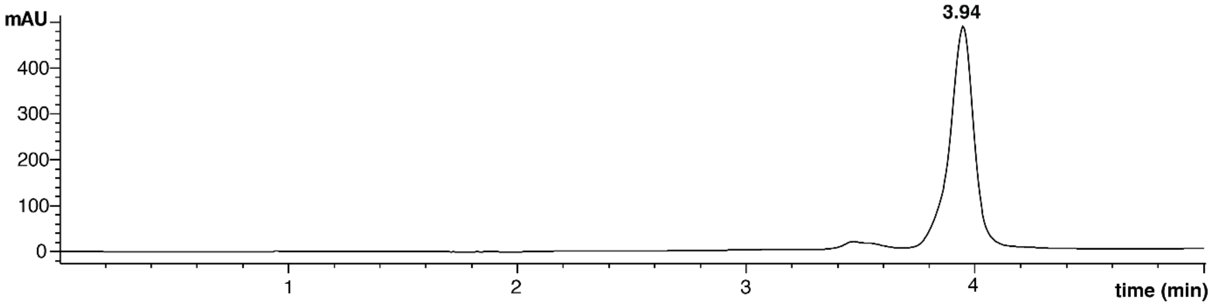

LC chromatogram of compound **s7b** (mAU at λ = 260 nm). Method E.

Intermediates **s7a,b** (50.00 mg, 0.08 mmol, 1.00 equiv.), CuI (6.08 mg, 0.03 mmol, 0.40 equiv.), bis(triphenylphosphine)palladium chloride ($PdCl_2(PPh_3)_2$, 8.41 mg, 0.01 mmol, 0.15 equiv.), **s5** (32.30 mg, 0.10 mmol, 1.20 equiv.), and DIPEA (111 *µ*L, 0.64 mmol, 8.00 equiv.) were dissolved in dry DMF (1 mL). The reactions were heated at 70 °C for 1 h. The reactions were cooled to rt, diluted with MeOH (6 mL) and filtered through celite. The solvents were evaporated under reduced pressure and the crudes were suspended in DCM (2 mL), and TFA (61.26 *µ*L, 0.8 mmol, 10.00 equiv.) was added. The reactions were stirred for 30 min and then concentrated under reduced pressure. The crude products were dissolved in DMF and purified *via* RP-HPLC (Method B). The compounds were obtained as white solids after lyophilization.

s8a (*S*): 7.08 mg, 0.01 mmol, 12% yield.

^{1}H NMR (400 MHz, DMSO-d_6) δ 8.96 (bs, 1H), 8.17 (s, 1H), 8.08 (bs, 1H), 7.46 - 7.14 (m, 10H), 7.06 - 6.87 (m, 7H), 5.00 (s, 2H), 4.42 - 4.32 (m, 1H), 3.96 (d, *J* = 14.5 Hz, 1H), 3.77 (d, *J* = 16.8 Hz, 2H), 3.19 - 3.08 (m, 1H), 3.02 (q, *J* = 7.2 Hz, 2H), 2.93 - 2.78 (m, 3H), 2.38 (bs, 1H), 1.38 - 0.97 (m, 12H).

HRMS (ESI, m/z) calcd. for $C_{37}H_{43}FN_5O_6PS$: $[M + H]^+$ 736.27285, detected: $[M + H]^+$ 736.27179.

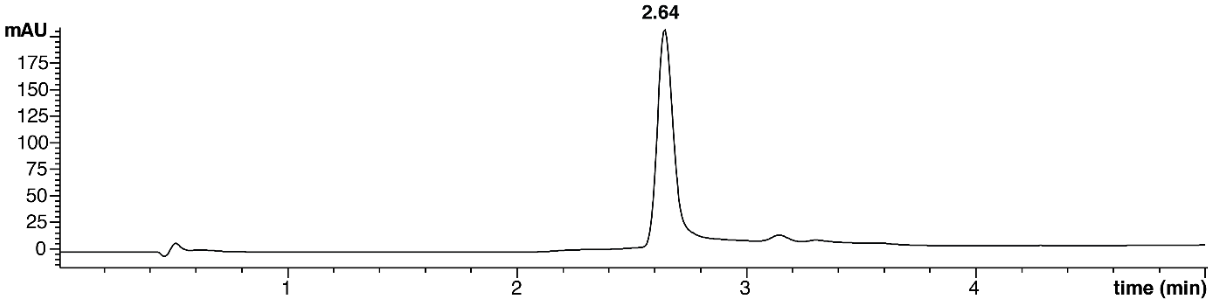

LC chromatogram of compound **s8a** (mAU at λ = 260 nm). Method D.

s8b (*R*): 8.31 mg, 0.01 mmol, 14% yield.

^{1}H NMR (400 MHz, DMSO-d_6) δ 8.94 (bs, 1H), 8.18 (s, 2H), 8.10 (bs, 2H), 7.44 - 7.11 (m, 12H), 7.09 - 6.78 (m, 8H), 5.04 (s, 2H), 4.41 - 4.31 (m, 1H), 3.95 (d, *J* = 14.5 Hz, 1H), 3.79 (d, *J* = 16.3 Hz, 2H), 3.19 - 3.08 (m, 1H), 3.03 (q, *J* = 7.4 Hz, 2H), 2.95 - 2.76 (m, 3H), 2.36 (bs, 2H), 1.35 - 0.97 (m, 14H).

HRMS (ESI, m/z) calcd. for $C_{37}H_{43}FN_5O_6PS$: $[M + H]^+$ 736.27285, detected: $[M + H]^+$ 736.27182.

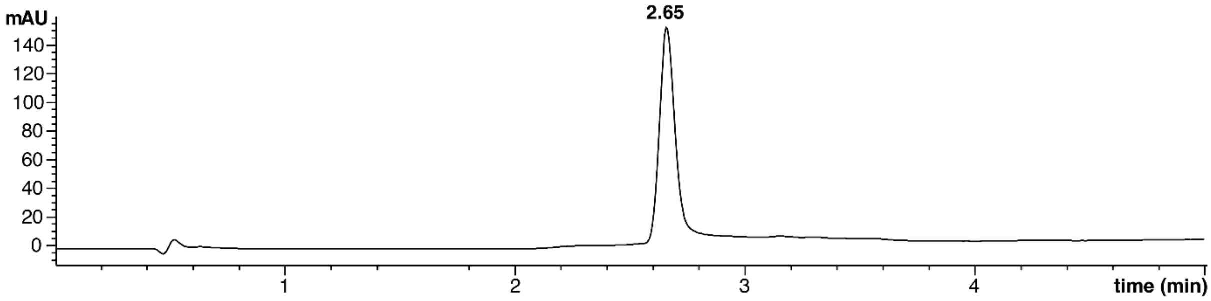

LC chromatogram of compound **s8b** (mAU at λ = 260 nm). Method E.

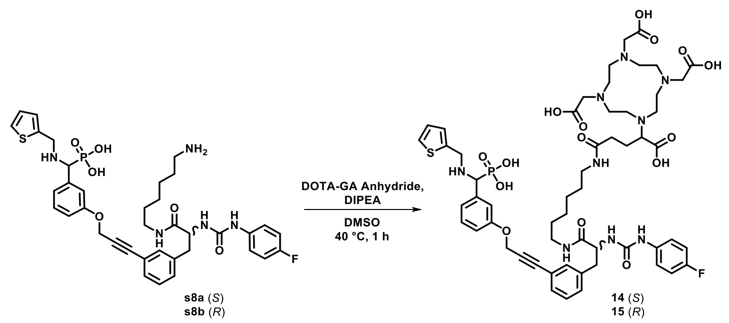

Compounds **14** and **15** were prepared via **GP5** (15.00 *µ*mol scale) from the respective amines **s8a** and **s8b**. The final compounds were purified *via* RP-HPLC (Method C) and obtained as white solid after lyophilization.

14 (*S*): 6.65 mg, 5.57 µmol, 37% yield.

^{1}H NMR (400 MHz, DMSO-d_6) δ 8.78 (d, J = 2.4 Hz, 1H), 8.08 - 8.02 (m, 1H), 7.84 (t, J = 5.4 Hz, 1H), 7.62 (dd, J = 5.1, 1.2 Hz, 1H), 7.45 - 7.38 (m, 2H), 7.38 - 7.28 (m, 5H), 7.28 - 7.19 (m, 3H), 7.15 (d, J = 3.5 Hz, 1H), 7.1 - 7.05 (m, 4H), 7.05 - 6.98 (m, 2H), 6.44 (t, J = 8.5 Hz, 1H), 5.06 (s, 2H), 4.44 - 4.28 (m, 3H), 4.24 - 4.08 (m, 3H), 4.02 (bs, 1H), 3.86 – 3.81 (m, 2H), 3.53 - 3.43 (m, 3H), 3.07 (dt, J = 12.5, 6.8 Hz, 5H), 3.03 - 2.86 (m, 9H), 2.83 - 2.73 (m, 2H), 2.34 (t, J = 8.2 Hz, 2H), 1.97 - 1.85 (m, 2H), 1.41 - 1.28 (m, 5H), 1.27 - 1.13 (m, 6H).

HRMS (ESI, m/z) calcd. for $C_{56}H_{73}FN_9O_{15}PS$: $[M + H]^+$ 1194.47413, detected: $[M + H]^+$ 1194.47053.

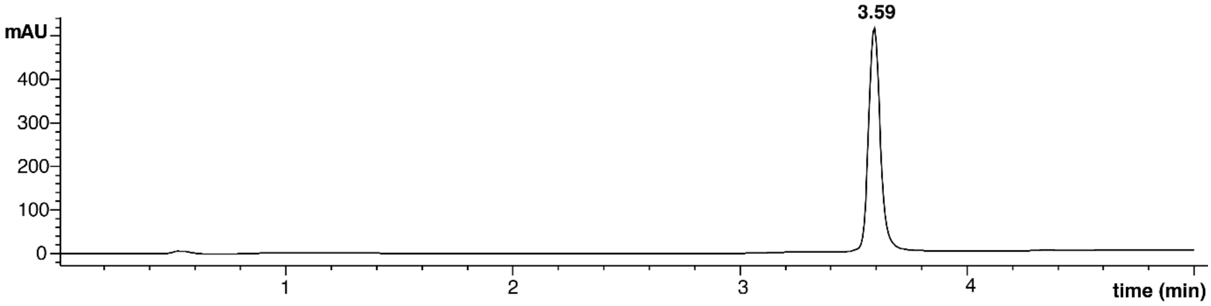

LC chromatogram of compound **14** (mAU at λ = 260 nm). Method D.

15 (*R*): 7.65 mg, 6.41 µmol, 43% yield.

^{1}H NMR (400 MHz, DMSO-d_6) δ 8.79 (d, J = 2.4 Hz, 1H), 8.07 - 8.02 (m, 1H), 7.85 (t, J = 5.4 Hz, 1H), 7.62 (dd, J = 5.1, 1.2 Hz, 1H), 7.41 (d, J = 7.7 Hz, 2H), 7.38 - 7.28 (m, 4H), 7.28 - 7.19 (m, 2H), 7.15 (d, J = 3.5 Hz, 1H), 7.10 - 6.97 (m, 5H), 6.44 (t, J = 8.5 Hz, 1H), 5.06 (s, 2H), 4.39 (q, J = 7.4 Hz, 1H), 4.32 (d, J = 14.2 Hz, 1H), 4.20 (d, J = 14.1 Hz, 1H), 4.13 (d, J = 16.6 Hz, 1H), 4.02 (bs, 1H), 3.90 - 3.73 (m, 4H), 3.53 - 3.43 (m, 3H), 3.30 - 3.14 (m, 4H), 3.12 - 2.72 (m, 14H), 2.34 (t, J = 8.2 Hz, 2H), 1.97 - 1.85 (m, 2H), 1.41 - 1.28 (m, 4H), 1.27 - 1.12 (m, 5H).

HRMS (ESI, m/z): calcd. for $C_{56}H_{73}FN_9O_{15}PS$: $[M + H]^+$ 1194.47413, detected: $[M + H]^+$ 1194.47025.

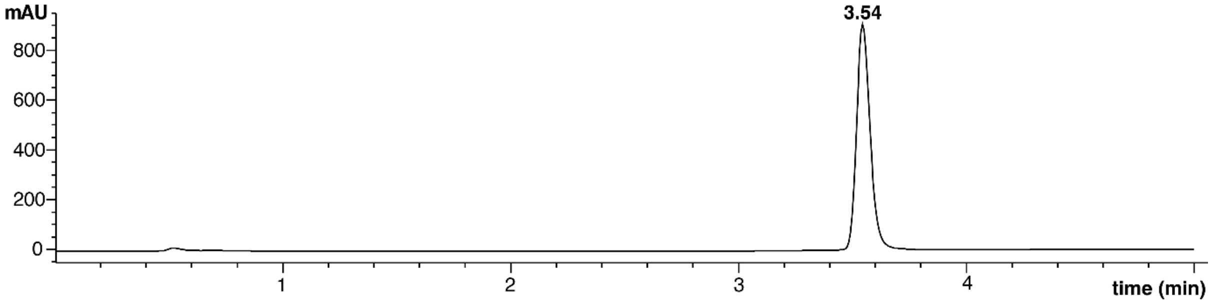

LC chromatogram of compound **15** (mAU at λ = 260 nm). Method D.

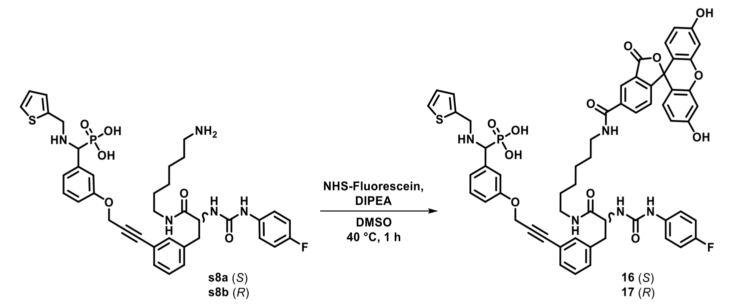

Intermediates **s8a** and **s8b** (1.50 mg, 2.04 μmol, 1.00 equiv.) were loaded into the respective Eppendorf tubes and dissolved in DMSO (204 μL, final concentration 0.01M). DIPEA (7.24 μL, 40.82 μmol, 20.00 equiv.) and a stock solution of 5-carboxyfluorescein-*N*-hydroxysuccinimide ester (NHS-Fluorescein, 49.21 μL, 2.45 μmol, 0.05 M in DMSO, 1.20 equiv.) were added to both mixtures. The reactions were shaken in a ThermoMixer at 40 °C for 1 h. The crude products were filtered and directly purified *via* RP-HPLC (Method C). The products were obtained as yellow solid after lyophilization.

16 (*S*): 0.91 mg, 0.83 μmol, 41% yield.

HRMS (ESI, m/z) calcd. for $C_{58}H_{53}FN_5O_{12}PS$: [M + H]$^+$ 1094.32059, detected: [M + H]$^+$ 1094.31907.

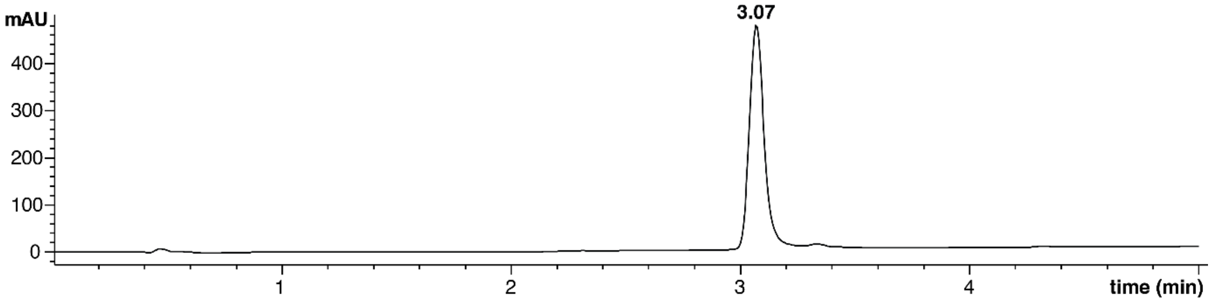

LC chromatogram of compound **16** (mAU at λ = 260 nm). Method E.

17 (*R*): 0.75 mg, 0.69 μmol, 34% yield.

HRMS (ESI, m/z) calcd. for $C_{58}H_{53}FN_5O_{12}PS$: [M + H]$^+$ 1094.32059, detected: [M + H]$^+$ 1094.31931.

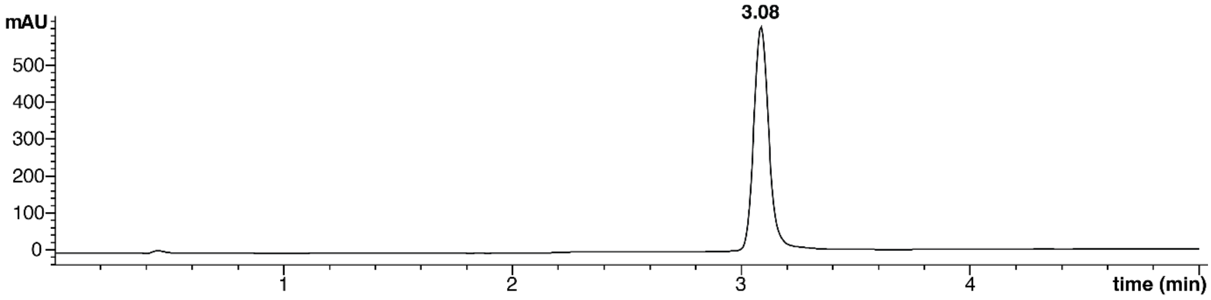

LC chromatogram of compound **17** (mAU at λ = 260 nm). Method E.

5.3.3 *Synthesis of CAIX hit compounds* ***18-25***

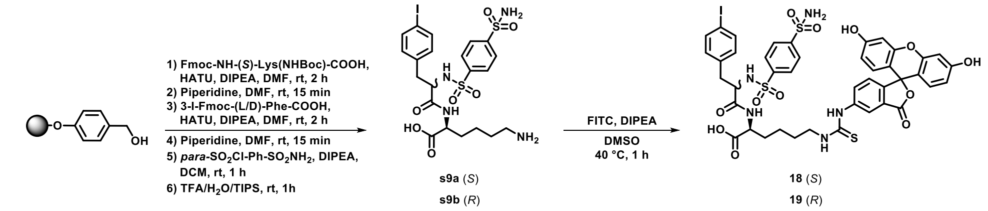

Commercially available Wang resin (500.00 mg, 0.55 mmol, 1.00 equiv.) was coupled to N2-(((9H-fluoren-9-yl)methoxy)carbonyl)-N6-(tert-butoxycarbonyl)-(*S*)-lysine (773.10 mg, 1.65 mmol, 3.00 equiv.) as described in **GP1** and Fmoc-deprotected as described in **GP2**. Then the resin was swollen in DMF and was separately coupled to the desired Fmoc-4-*iodo*-phenylalanin enantiomer (846.91 mg, 0.55 mmol, 1.00 equiv.) as described in **GP1**, and Fmoc-deprotected following **GP2**. After washes of the resins with DCM, a solution of 4-(aminosulfonyl)benzenesulfonyl chloride (421.92 mg, 1.65 mmol, 3.00 equiv.) and DIPEA (578.20 μL, 3.30 mmol, 6.00 equiv.) in DCM (5 mL) was separately added to the resins and reacted at rt for 1 h. After washing steps in DMF and DCM, the resins were cleaved as described in **GP4** and crude products were purified *via* RP-HPLC (Method B). After lyophilization, compounds **s9a,b** were obtained as white solids.

s9a (*S*): 52.70 mg, 0.09 mmol, 15% yield.

^{1}**H NMR** (400 MHz, DMSO-d_6) δ 8.06 (t, J = 5.4 Hz, 1H), 7.93 (d, J = 8.2 Hz, 2H), 7.77 (d, J = 8.2 Hz, 2H), 7.60 (d, J = 7.9 Hz, 2H), 6.98 (d, J = 7.8 Hz, 2H), 3.94 (dd, J = 9.3, 5.5 Hz, 1H), 3.38 (t, J = 6.3 Hz, 1H), 2.79 (dd, J = 13.5, 5.4 Hz, 1H), 2.745 - 2.62 (m, 3H), 1.73 - 1.54 (m, 2H), 1.28 - 1.10 (m, 4H).
HRMS (ESI, m/z): calcd. for $C_{21}H_{27}IN_4O_7S_2$: [M + H]$^+$ 639.04385, detected: [M + H]$^+$ 639.04314.

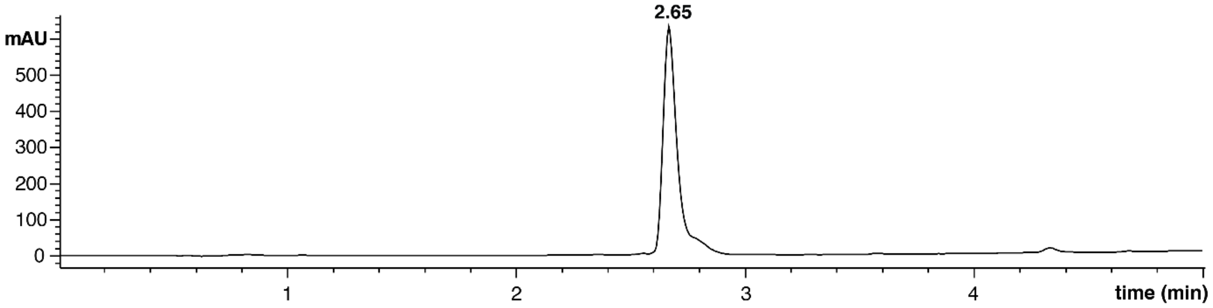

LC chromatogram of **s9a** (mAU at λ = 260 nm). Method E.

s9b (*R*): 42.12 mg, 0.07 mmol, 12% yield.

^{1}**H NMR** (400 MHz, DMSO-d_6) δ 8.05 (t, J = 5.4 Hz, 1H), 7.92 (d, J = 8.2 Hz, 2H), 7.78 (d, J = 8.2 Hz, 2H), 7.60 (d, J = 7.9 Hz, 2H), 6.98 (d, J = 8.0 Hz, 2H), 3.94 (dd, J = 9.1, 5.8 Hz, 1H), 3.38 (t, J = 6.1 Hz, 1H), 2.79 (dd, J = 13.7, 5.6 Hz, 1H), 2.74 - 2.64 (m, 3H), 1.74 - 1.53 (m, 2H), 1.33 - 1.12 (m, 4H).
HRMS (ESI, m/z) calcd. for $C_{21}H_{27}IN_4O_7S_2$: [M + H]$^+$ 639.04385, detected: [M + H]$^+$ 639.04311.

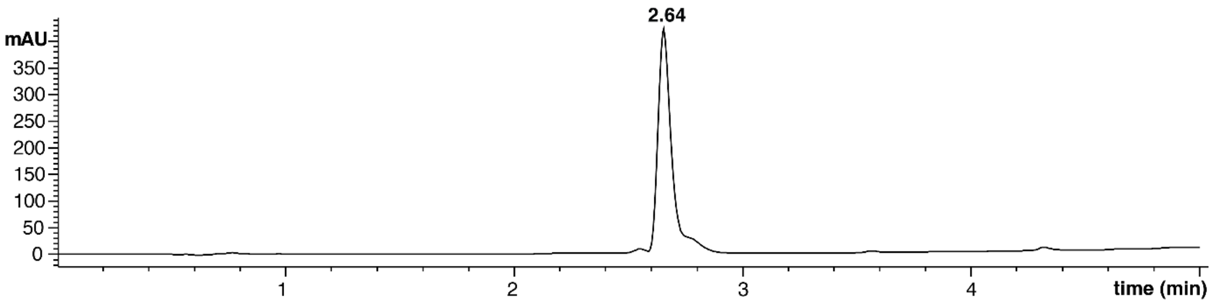

LC chromatogram of **s9b** (mAU at λ = 260 nm). Method E.

Compounds **18** and **19** were prepared *via* **GP6** (3.00 *μ*mol scale) from the respective amines **s9a** and **s9b**. The final compounds were purified by RP-HPLC and obtained as red solid after lyophilization.

18 (*S*): 1.93 mg, 1.82 μmol, 60% yield.

HRMS (ESI, m/z) calcd. for $C_{42}H_{38}IN_5O_{12}S$: $[M + H]^+$ 1028.07965, detected $[M + H]^+$ 1028.07936.

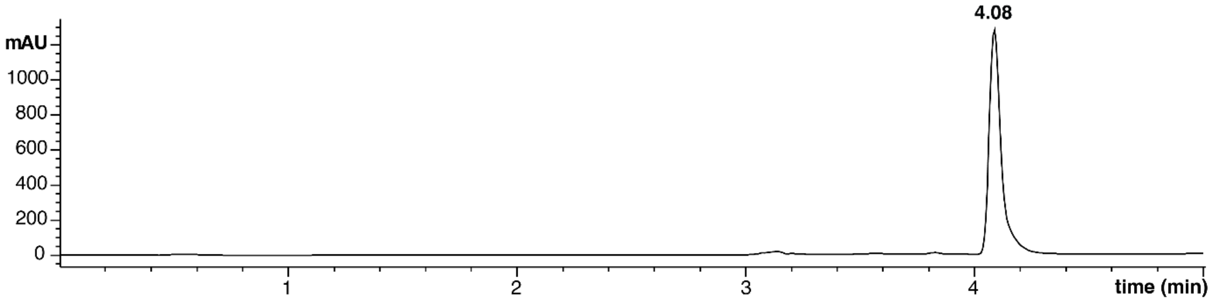

LC chromatogram of compound **18** (mAU at λ = 260 nm). Method D.

19 (*R*): 2.04 mg, 1.91 μmol, 65% yield.

HRMS (ESI, m/z) calcd. for $C_{42}H_{38}IN_5O_{12}S_3$: $[M + H]^+$ 1028.07965, detected $[M + H]^+$ 1028.08005.

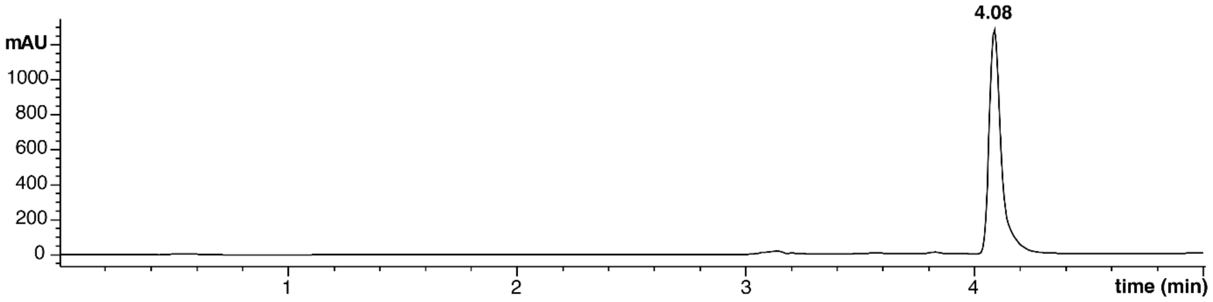

LC chromatogram of compound **19** (mAU at λ = 260 nm). Method D.

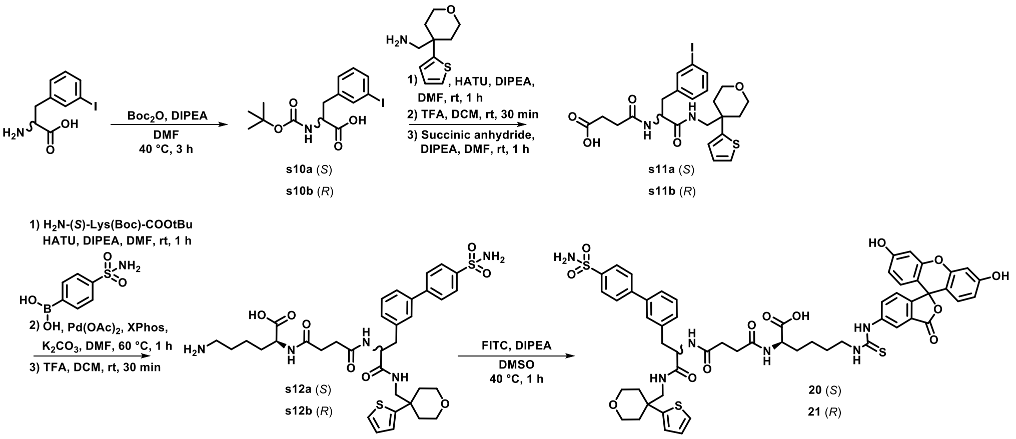

3(*S*)-*iodo*-phenylalanine and 3(*R*)-*iodo*-phenylalanine (500.00 mg, 1.70 mmol, 1.00 equiv.) were loaded into the respective reaction vessel and dissolved in DMF (5 mL). Di-*tert*-butyldicarbonat (444.72 mg, 2.04 mmol, 1.20 equiv.) and DIPEA (1.22 mL, 6.0 mmol, 4.00 equiv.) were added to the solutions and the mixtures were stirred for 3 h. The reaction mixtures were worked up by extraction with EtOAc (30 mL). The organic phases were separately washed with sat. aq. ammonium chloride (3 x 20 mL) and brine (3 x 20 mL). The washed organic phases were dried over anhydrous sodium sulphate, filtered and concentrated under reduced pressure. The crude mixtures were used without further purification for the next step.

S10a,b: m/z calculated for $C_{14}H_{18}INO_2$ $[M + H]^+$ 392.03; detected $[M + H]^+$ 392.1

Intermediates **s10a,b** (675.00 mg, 1.73 mmol, 1.00 equiv.) were loaded into the respective reaction vessel and dissolved in DMF (5 mL). HATU (657.40 mg, 1.73 mmol, 1.00 equiv.) and DIPEA (904.00 μL, 5.19 mmol, 3.00 equiv.) were added and reactions stirred for 10 minutes. (4-(thiophen-2-yl)tetrahydro-2H-pyran-4-yl)methanamine (346.70 mg, 1.73 mmol, 1.00 equiv.) was added to the reaction vessels and the mixtures were stirred for 50 min. The reactions were acidified with an aqueous solution of HCl 2M until pH ~ 6 and separately diluted by adding EtOAc (20 mL). Both mixtures were separately washed with sat. aq. ammonium chloride (3 x 20 mL) and sat. aq. sodium chloride (2 x 20 mL). The organic layers were dried over anhydrous sodium sulfate, filtered, and concentrated under reduced pressure. The crudes were separately dissolved in DCM (5 mL). TFA (1.30 mL, 17.30 mmol, 10.00 equiv.) was added and mixtures let stir for 30 min. The reactions were concentrated under reduced pressure. The crudes were separately dissolved in DMF (5 mL), succinic anhydride (173.10 mg, 1.73 mmol, 1.00 equiv.) and DIPEA (904.30 μL, 5.19 mmol, 3.00 equiv.) were added and the mixtures were stirred at rt for 1 h. After this time, the reactions were acidified with an aqueous solution of HCl 2M until pH ~ 3 and separately diluted by adding EtOAc (20 mL). Both mixtures were washed with sat. aq. ammonium chloride (3 x 20 mL) and sat. aq. sodium chloride (2 x 20 mL). The organic layers were dried over anhydrous sodium sulfate, filtered, concentrated under reduced pressure and purified *via* RP-HPLC (Method B) to obtain the products as pale-yellow solids after lyophilization.

s11a (*S*): 490.62 mg, 0.91 mmol, 50 % yield.

^{1}H NMR (400 MHz, DMSO-d_6) δ 8.28 (d, *J* = 8.4 Hz, 1H), 7.88 (t, *J* = 6.3 Hz, 1H), 7.76 (t, *J* = 1.7 Hz, 1H), 7.68 (dt, *J* = 7.8, 1.4 Hz, 1H), 7.54 (dd, *J* = 5.1, 1.1 Hz, 1H), 7.37 (dt, *J* = 7.7, 1.3 Hz, 1H), 7.19 (t, *J* = 7.7 Hz, 1H), 7.13 (dd, *J* = 5.1, 3.5 Hz, 1H), 7.03 (dd, *J* = 3.6, 1.2 Hz, 1H), 4.61 (td, *J* = 9.1, 5.0 Hz, 1H), 3.84 (dt, *J* = 11.6, 4.0 Hz, 2H), 3.56 - 3.49 (m, 2H), 3.46 (dd, *J* = 13.6, 6.9 Hz, 2H), 3.25 (dd, *J* = 13.3, 5.8 Hz, 1H), 3.00 (dd, *J* = 13.7, 5.0 Hz, 1H), 2.78 (dd, *J* = 13.6, 9.7 Hz, 1H), 2.51 - 2.32 (m, 4H), 2.05 - 1.96 (m, 1H), 1.89 (ddt, *J* = 13.9, 6.1, 3.9 Hz, 3H), 1.42 (ddd, *J* = 14.3, 6.8, 2.8 Hz, 2H).

HRMS (ESI, m/z) calcd. for $C_{23}H_{27}IN_2O_5S$: [M + H]$^+$ 571.07851, detected [M + H]$^+$ 571.07496.

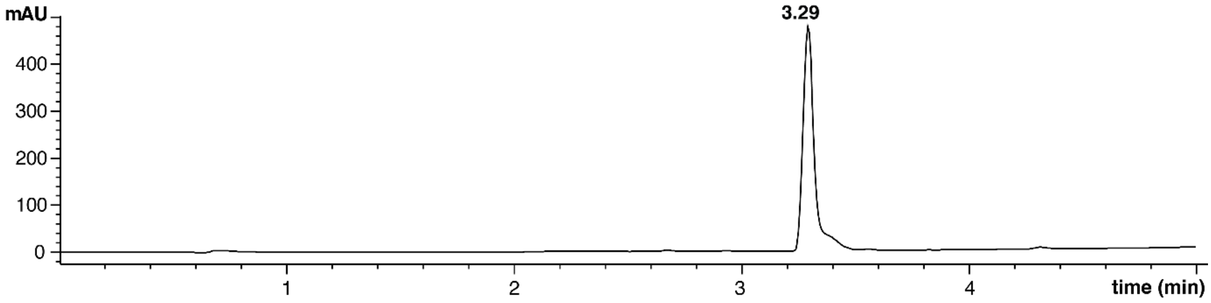

LC chromatogram of **s11a** (mAU at λ = 260 nm). Method E.

s11b (*R*): 421.92 mg, 0.81 mmol, 43% yield.

^{1}H NMR (400 MHz, DMSO-d_6) δ 8.30 (d, *J* = 8.4 Hz, 1H), 7.90 (t, *J* = 6.3 Hz, 1H), 7.78 (t, *J* = 1.7 Hz, 1H), 7.70 (dt, *J* = 7.9, 1.4 Hz, 1H), 7.56 (dd, *J* = 5.1, 1.1 Hz, 1H), 7.39 (dt, *J* = 7.7, 1.3 Hz, 1H), 7.21 (t, *J* = 7.7 Hz, 1H), 7.15 (dd, *J* = 5.1, 3.5 Hz, 1H), 7.05 (dd, *J* = 3.6, 1.2 Hz, 1H), 4.63 (td, *J* = 9.1, 5.0 Hz, 1H), 3.86 (dt, *J* = 11.6, 4.0 Hz, 2H), 3.58 - 3.43 (m, 4H), 3.27 (dd, *J* = 13.3, 5.8 Hz, 1H), 3.02 (dd, *J* = 13.7, 5.0 Hz, 1H), 2.81 (dd, *J* = 13.7, 9.7 Hz, 1H), 2.56 - 2.34 (m, 4H), 2.07 - 1.98 (m, 1H), 1.98 - 1.84 (m, 3H), 1.44 (ddd, *J* = 14.2, 6.8, 2.8 Hz, 2H).

HRMS (ESI, m/z) calcd. for $C_{23}H_{27}IN_2O_5S$: [M + H]$^+$ 571.07851, detected [M + H]$^+$ 571.07520.

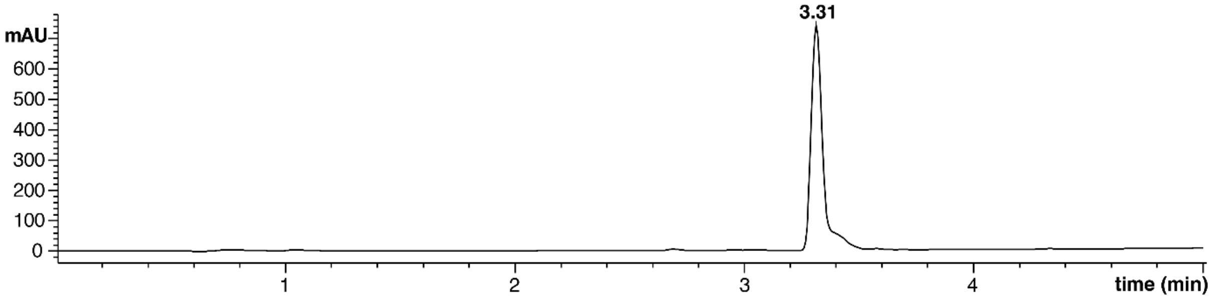

LC chromatogram of **s11b** (mAU at λ = 260 nm). Method E.

Compounds **s11a,b** (200.00 mg, 0.47 mmol, 1.00 equiv.) were loaded into the respective reaction vessel and dissolved in DMF (5 mL). HATU (178.60 mg, 0.47 mmol, 1.00 equiv.) and DIPEA (247.03 μL, 1.41 mmol, 3.00 equiv.) were added and the reactions were left stirred for 10 min. After this time N_6-(*tert*-butoxycarbonyl)-(*S*)-lysine (142.03 mg, 0.47 mmol, 1.00 equiv.) was added to the reaction vessels and let stir at rt for 1 h. The reactions were concentrated under reduced pressure. The resulting

residues were separately diluted in EtOAc (5 mL) and washed with sat. aq. sodium bicarbonate (2 x 15 mL) and brine (20 mL). The organic layers were dried over anhydrous sodium sulfate, filtered, and concentrated under reduced pressure. The two mixtures were separately dissolved in dry DMF (2 mL), K_2CO_3 (259.44 mg, 1.88 mmol, 4.00 equiv.), $Pd(OAc)_2$ (21.10 mg, 0.09 mmol, 0.20 equiv.), XPhos (89.62 mg, 0.19 mmol, 0.40 equiv.) and (4-sulfamoylphenyl)boronic acid (141.77 mg, 0.71 mmol, 1.50 equiv.) were added. Reactions were stirred for 1 h at 60 °C and then diluted with EtOAc (15 mL) and washed with water (3 x 15 mL). The organic layers were dried over anhydrous sodium sulfate, filtered, and concentrated under reduced pressure. The crudes were separately dissolved in DCM (3 mL). TFA (359.59 µL, 4.7 mmol, 10.00 equiv.) was added to the mixtures and reactions allowed to proceed at rt for 30 min. Both reactions were concentrated under reduced pressure. The crude products were purified *via* RP-HPLC (Method B) to obtain the **s12a,b** as pale-yellow solids after lyophilization.

s12a (S): 102.51 mg, 0.14 mmol, 30% yield.

^{1}H NMR (400 MHz, DMSO-*d*$_6$) 8.24 (d, *J* = 8.3 Hz, 1H), 7.95 - 7.75 (m, 8H), 7.62 (bs, 1H), 7.58 - 7.53 (m, 1H), 7.42 - 7.37 (m, 2H), 7.28 (d, *J* = 7.6 Hz, 1H), 6.98 (dd, *J* = 5.1, 3.5 Hz, 1H), 6.90 - 6.84 (m, 1H), 4.55 (m, 1H), 4.07 (s, 1H), 3.67 (m, 3H), 3.40 - 3.26 (m, 4H), 3.13 (dd, *J* = 13.3, 5.8 Hz, 1H), 3.04 (dd, *J* = 13.9, 4.9 Hz, 1H), 2.85 - 2.68 (m, 4H), 2.36 - 2.17 (m, 5H), 1.93 - 1.63 (m, 6H), 1.52 (sm 3H), 1.30 (m, 2H).

HRMS (ESI, m/z) calcd. for $C_{35}H_{45}N_5O_8S_2$: [M + H]$^+$ 728.27823, detected: [M + H]$^+$ 728.27733.

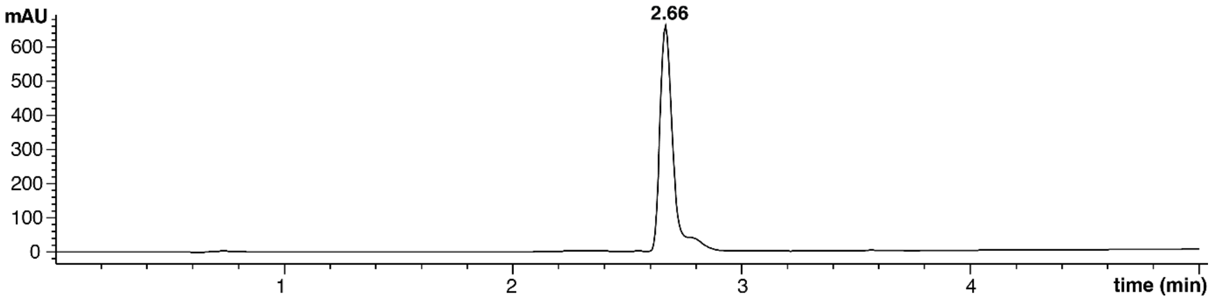

LC chromatogram of **s12a** (mAU at λ = 260 nm). Method E.

s12b (S): 50.9 mg, 0.07 mmol, 20 % yield.

^{1}H NMR (400 MHz, DMSO-*d*$_6$) δ 8.24 (d, *J* = 8.3 Hz, 1H), 7.97 - 7.74 (m, 8H), 7.62 (bs, 1H), 7.56 (dt, *J* = 7.8, 1.4 Hz, 1H), 7.41 - 7.39 (m, 2H), 7.28 (d, *J* = 7.6 Hz, 1H), 6.98 (dd, *J* = 5.1, 3.5 Hz, 1H), 6.88 - 6.85 (m, 1H), 4.54 (m, 1H), 4.07 (s, 1H), 3.68 (m, 3H), 3.39 - 3.25 (m, 4H), 3.18 - 3.10 (m, 1H), 3.04 (dd, *J* = 13.8, 4.9 Hz, 1H), 2.84 - 2.68 (m, 4H), 2.36 - 2.18 (m, 5H), 1.88 - 1.62 (m, 6H), 1.52 (m, 3H), 1.30 (m, 2H).

HRMS (ESI, m/z) calcd. for $C_{35}H_{45}N_5O_8S_2$: [M + H]$^+$ 728.27823, detected: [M + H]$^+$ 728.27724.

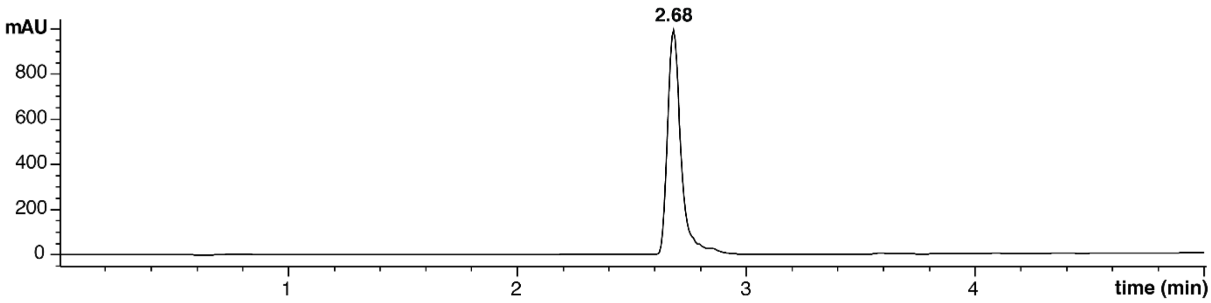

LC chromatogram of **s12b** (mAU at λ = 260 nm). Method E.

Compounds **20** and **21** were prepared *via* **GP6** (3.00 *μ*mol scale) from the respective amines **s12a** and **s12b**. The final compounds were purified *via* RP-HPLC (Method C) and obtained as red solid after lyophilization.

20 (*S*): 2.23 mg, 2.00 µmol, 66% yield.

HRMS (ESI, m/z) calcd. for $C_{56}H_{56}N_6O_{13}S_3$: $[M + H]^+$ 1117.31403, detected: $[M + H]^+$ 1117.31382.

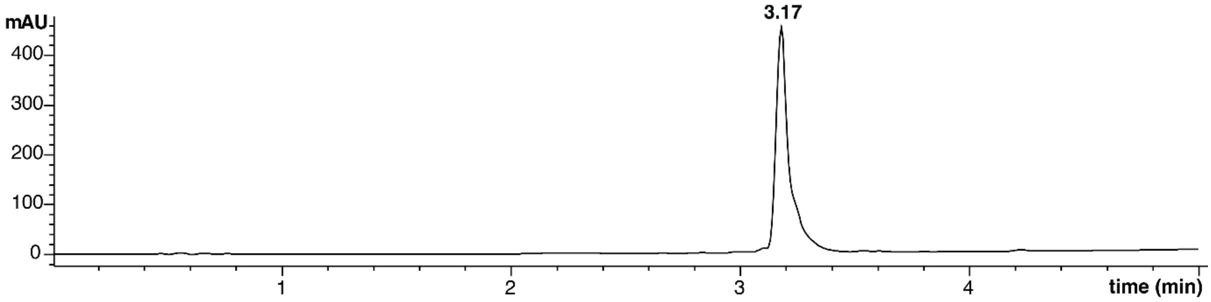

LC chromatogram of compound **20** (mAU at λ = 260 nm). Method D.

21 (*R*): 2.00 mg, 1.80 µmol, 60% yield.

HRMS (ESI, m/z) calcd. for $C_{56}H_{56}N_6O_{13}S_3$: $[M + H]^+$ 1117.31403, detected: $[M + H]^+$ 1117.31467

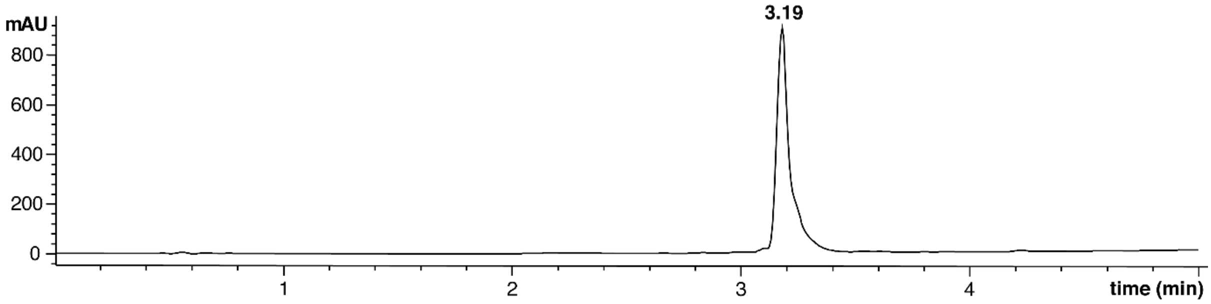

LC chromatogram of compound **21** (mAU at λ = 260 nm). Method D.

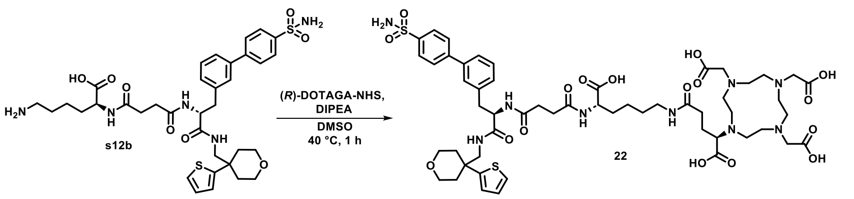

Compound **22** was prepared *via* **GP5** (20.00 µmol scale) from the respective amines **s12b**. The final compounds were purified *via* RP-HPLC (Method C) and obtained as white solid after lyophilization.

22: 12.57 mg, 10.60 µmol, 53% yield.

^{1}**H NMR** (400 MHz, DMSO-d_6) δ 8.21 (d, J = 8.2 Hz, 1H), 8.11 (d, J = 7.8 Hz, 1H), 7.96 - 7.71 (m, 6H), 7.62 (t, J = 1.9 Hz, 1H), 7.56 (dt, J = 7.7, 1.3 Hz, 1H), 7.49 - 7.35 (m, 4H), 7.31 - 7.25 (m, 1H), 7.03 - 6.94 (m, 1H), 6.87 (dd, J = 3.6, 1.2 Hz, 1H), 4.53 (td, J = 8.8, 5.0 Hz, 1H), 4.12 (m, 2H), 3.96 – 3.76 (m, 4H), 3.71 – 3.41 (m, H), 3.40 – 3.22 (m, 8H), 3.17 – 2.73 (m, 14H), 2.44 – 2.31 (m, 3H), 2.31 (s, 1H), 2.30 - 2.12 (m, 3H), 1.98 - 1.91 (m, 1H), 1.91 - 1.62 (m, 6H), 1.61 - 1.47 (m, 1H), 1.36 (t, J = 7.7 Hz, 2H), 1.32 - 1.20 (m, 2H).

HRMS (ESI, m/z) calcd. for $C_{54}H_{75}N_9O_{17}S_2$: $[M + H]^+$ 1186.47951, detected: $[M + H]^+$ 1186.47655.

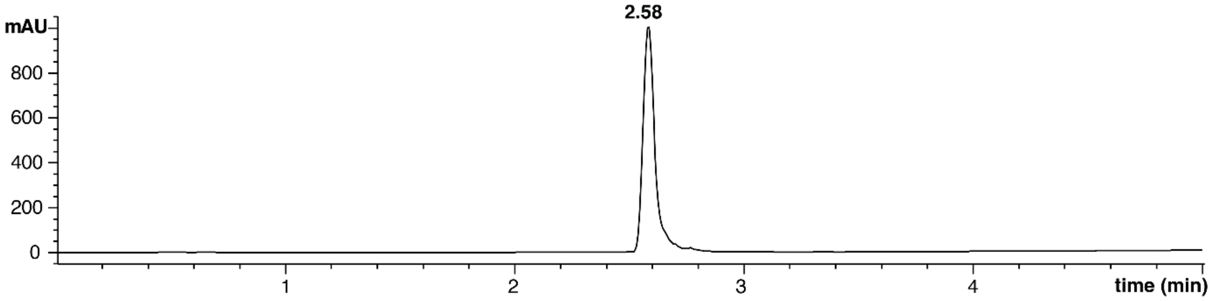

LC chromatogram of compound **22** (mAU at λ = 260 nm). Method D.

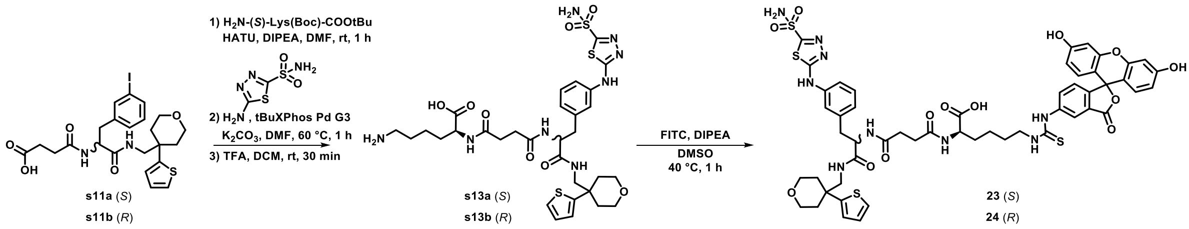

Compounds **s11a,b** (200.00 mg, 0.47 mmol, 1.00 equiv.) were loaded into the respective reaction vessel and dissolved in DMF (5 mL). HATU (1378.60 mg, 0.47 mmol, 1.00 equiv.) and DIPEA (247.03 µL, 1.41 mmol, 3.00 equiv.) were added and the reactions were allowed to proceed at rt for 10 min. N_6-(*tert*-butoxycarbonyl)-(*S*)-lysine (142.03 mg, 0.47 mmol, 1.00 equiv.) was added to the reaction vessels and let react at rt for 1 h. The reactions were concentrated under reduced pressure. The resulting residues were separately diluted in EtOAc (5 mL) and washed with sat. aq. sodium bicarbonate (2 x 15 mL) and brine (20 mL). The organic layers were dried over anhydrous sodium sulfate, filtered, and concentrated under reduced pressure. The mixtures were separately dissolved in dry DMF (2 mL), K_2CO_3 (259.44 mg, 1.88 mmol, 4.00 equiv), [(2-di-*tert*-butylphosphino-2′,4′,6′-triisopropyl-1,1′-biphenyl)-2-(2′-amino-1,1′-biphenyl)] palladium(II) methanesulfonate (tBuXPhos Pd G3, 74.77 mg,

0.09 mmol, 0.20 equiv.) and 5-amino-1,3,4-thiadiazole-2-sulfonamide (126.90 mg, 0.71 mmol, 1.50 equiv.) were added. Reactions were stirred at 90 °C for 2 h. Both mixtures were separately diluted with EtOAc (15 mL) and washed with water (3 x 15 mL). The organic layers were dried over anhydrous sodium sulfate, filtered, and concentrated under reduced pressure. The crudes were dissolved in DCM (3 mL), TFA (359.59 µL, 4.7 mmol, 10.00 equiv) was added and the mixtures were stirred at rt for 30 min. The reactions were concentrated under reduced pressure. The crude products were purified *via* RP-HPLC (Method B) to obtain compounds **s13a,b** as pale-yellow solids after lyophilization.

s13a (*S*): 52.96 mg, 0.07 mmol, 15% yield.

^{1}H NMR (400 MHz, DMSO-d_6) δ 10.77 (s, 1H), 8.26 (s, 2H), 8.22 - 8.09 (m, 2H), 7.78 - 7.61 (m, 4H), 7.51 (dd, J = 8.1, 2.5 Hz, 1H), 7.45 - 7.37 (m, 2H), 7.28 (t, J = 7.9 Hz, 1H), 7.00 (dd, J = 5.1, 3.5 Hz, 1H), 6.95 (d, J = 7.6 Hz, 1H), 6.88 (td, J = 4.2, 3.6, 1.1 Hz, 1H), 4.45 (tt, J = 8.4, 2.8 Hz, 1H), 4.19 - 4.09 (m, 1H), 3.74 - 3.63 (m, 3H), 3.13 (td, J = 13.2, 12.7, 6.1 Hz, 2H), 2.99 - 2.90 (m, 1H), 2.80 - 2.67 (m, 4H), 2.41 - 2.16 (m, 4H), 1.86 - 1.63 (m, 7H), 1.62 - 1.45 (m, 4H), 1.39 - 1.25 (m, 2H).

HRMS (ESI, m/z) calcd. for $C_{31}H_{42}N_8O_8S_3$: [M + H]$^+$ 751.23605, detected: [M + H]$^+$ 751.23660.

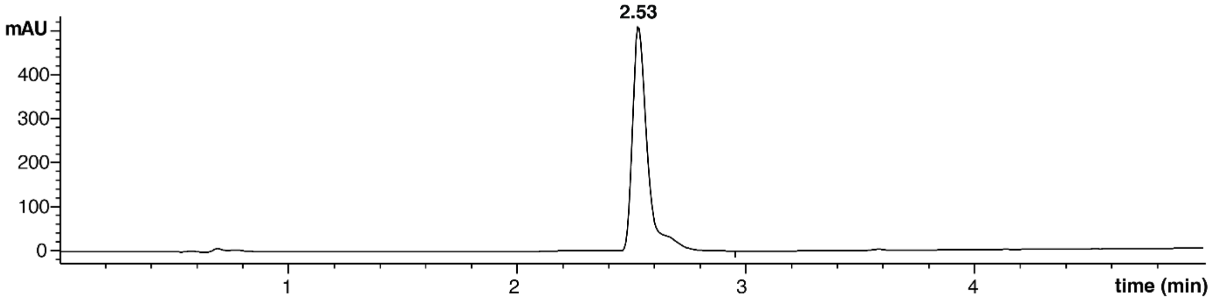

LC chromatogram of compound **s13a** (mAU at λ = 260 nm). Method D.

s13b (*R*): 37.55 mg, 0.05 mmol, 10% yield.

^{1}H NMR (400 MHz, DMSO-d_6) δ 10.76 (s, 1H), 8.25 (s, 2H), 8.23 - 8.08 (m, 2H), 7.76 - 7.58 (m, 4H), 7.51 (dd, J = 8.1, 2.5 Hz, 1H), 7.47 - 7.39 (m, 2H), 7.29 (t, J = 7.9 Hz, 1H), 7.00 (dd, J = 5.1, 3.5 Hz, 1H), 6.96 (d, J = 7.6 Hz, 1H), 6.84 (td, J = 4.2, 3.6, 1.1 Hz, 1H), 4.45 (tt, J = 8.8, 2.6 Hz, 1H), 4.19 - 4.09 (m, 1H), 3.74 - 3.63 (m, 3H), 3.13 (td, J = 13.5, 12.2, 6.3 Hz, 2H), 2.99 - 2.90 (m, 1H), 2.80 - 2.67 (m, 4H), 2.41 - 2.16 (m, 4H), 1.86 - 1.63 (m, 7H), 1.62 - 1.45 (m, 4H), 1.39 - 1.25 (m, 2H).

HRMS (ESI, m/z) calcd. for $C_{31}H_{42}N_8O_8S_3$: [M + H]$^+$ 751.23605, detected: [M + H]$^+$ 751.23640.

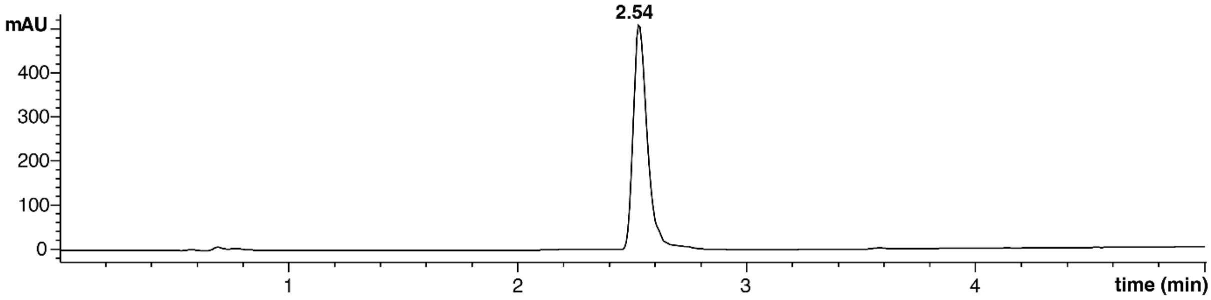

LC chromatogram of compound **s13b** (mAU at λ = 260 nm). Method D.

Compounds **23** and **24** were prepared *via* **GP6** (3.00 μmol scale) from the respective amines **s13a** and **s13b**. The final compounds were purified *via* RP-HPLC (Method C) and obtained as red solid after lyophilization.

23 (*S*): 2.22 mg, 1.95 µmol, 65% yield.

HRMS (ESI, m/z) calcd. for $C_{52}H_{53}N_9O_{13}S_4$: [M + H]$^+$ 1140.27185, detected: [M + OH]$^+$ 1156.26527.

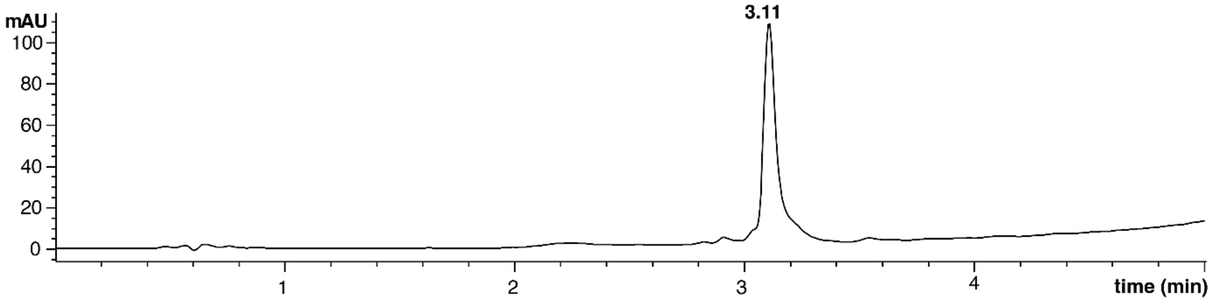

LC chromatogram of compound **23** (mAU at λ = 260 nm). Method D.

24 (*S*): 1.88 mg, 1.65 µmol, 55% yield.

HRMS (ESI, m/z) calcd. for $C_{52}H_{53}N_9O_{13}S_4$ [M + H]$^+$ 1140.27185, detected: [M + OH]$^+$ 1156.26537.

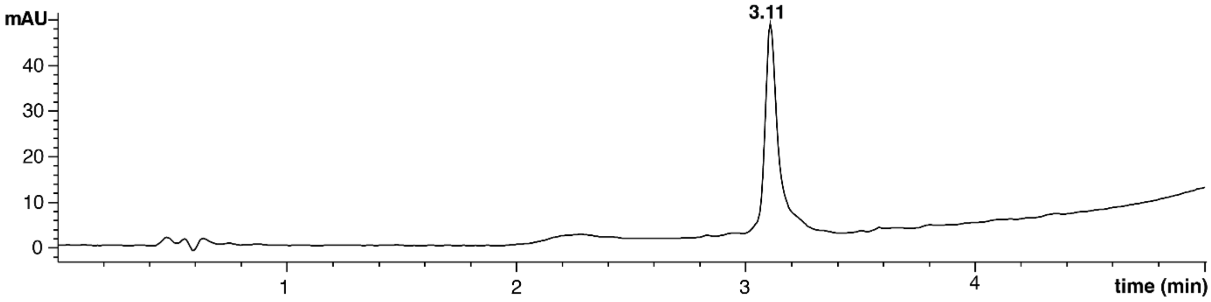

LC chromatogram of compound **24** (mAU at λ = 260 nm). Method D.

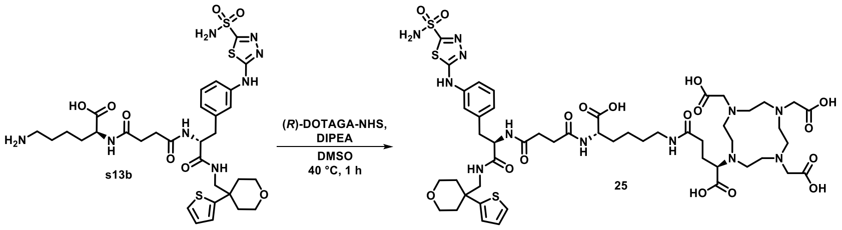

Compound **25** were prepared *via* **GP5** (20.00 μmol scale) from the respective amines **s13b**. The final compounds were purified *via* RP-HPLC (Method C) and obtained as white solid after lyophilization.

25: 10.88 mg, 9.00 µmol, 45% yield.

^{1}H NMR (400 MHz, DMSO-d_6) δ 10.77 (s, 1H), 8.26 (s, 2H), 8.14 (dd, J = 17.2, 8.0 Hz, 2H), 7.85 (t, J = 5.6 Hz, 1H), 7.73 (t, J = 6.4 Hz, 1H), 7.51 (dd, J = 8.3, 2.2 Hz, 1H), 7.44 - 7.37 (m, 2H), 7.28 (t, J = 7.8 Hz, 1H), 7.00 (dd, J = 5.1, 3.5 Hz, 1H), 6.97 - 6.93 (m, 1H), 6.87 (dd, J = 3.6, 1.2 Hz, 1H), 4.45 (td,

J = 8.8, 5.2 Hz, 1H), 4.13 (td, J = 8.4, 5.4 Hz, 2H), 3.95 - 3.74 (m, 9H) 3.72 - 3.64 (m, 3H), 3.41 - 3.20 (m, 9H), 3.15 - 2.64 (m, 15H), 2.40 - 2.15 (m, 7H), 2.34 - 2.30 (m, 2H), 2.30 - 2.13 (m, 2H), 1.97 - 1.58 (m, 7H), 1.61 - 1.44 (h, J = 8.0 Hz, 1H), 1.41 - 1.22 (m, 4H).

HRMS (ESI, m/z): calcd. for $C_{54}H_{57}N_9O_{17}S_2$: $[M + H]^+$ 1209.43733, detected: $[M + H]^+$ 1209.43477.

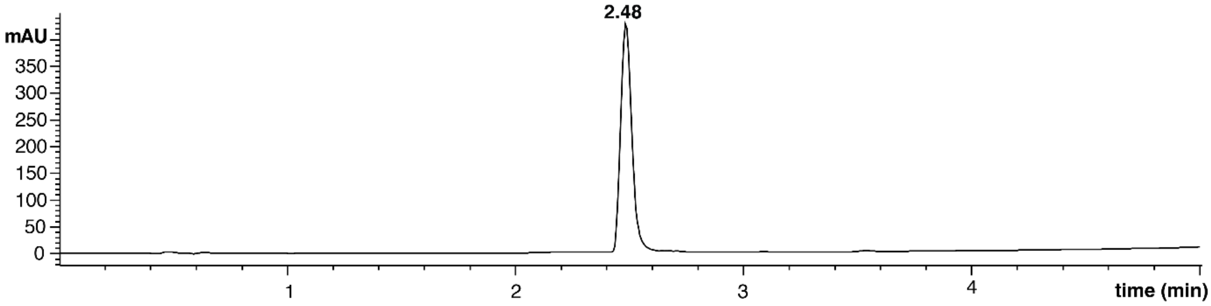

LC chromatogram of compound **25** (mAU at λ = 260 nm). Method D.

*5.3.4 Synthesis of NKG2D hit compounds **26**-**33***

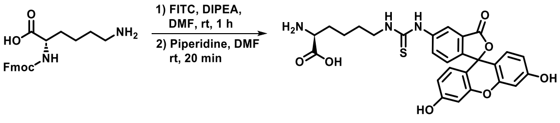

(((9H-fluoren-9-yl)methoxy)carbonyl)-(*S*)-lysine (200.00 mg, 0.54 mmol, 1.00 equiv.) was loaded in the reaction vessel and dissolved in DMF (3 mL). DIPEA (189.21 µL, 1.08 mmol, 2.00 equiv.) and fluorescein isothiocyanate (210.26 mg, 0.54 mmol, 1.00 equiv) were added and reaction was stirred at rt for 1 h. Piperidine (208.92 µL, 2.16 mmol, 4.0 equiv.) was added to the mixture that was reacted at rt for 20 minutes. Compound **L1** was directly purified *via* preparative RP-HPLC (Method B).

L1: 100.55 mg, 0.19 mmol, 35% yield.

^{1}H NMR (400 MHz, DMSO-d_6) δ 11.35 (s, 1H), 9.44 (s, 1H), 8.40 (s, 1H), 7.91 (dd, J = 8.4, 2.0 Hz, 1H), 7.14 (d, J = 8.3 Hz, 1H), 6.67 (d, J = 2.1 Hz, 2H), 6.60 - 6.55 (m, 3H), 3.49 (bs, 3H), 1.83 - 1.67 (m, 2H), 1.65 - 1.51 (m, 3H), 1.50 - 1.34 (q, J = 8.3, 7.8 Hz, 2H).

HRMS (ESI, m/z) calcd. for $C_{27}H_{25}N_3O_7S$: $[M + H]^+$ 536.14860, detected: $[M + H]^+$ 536.14814.

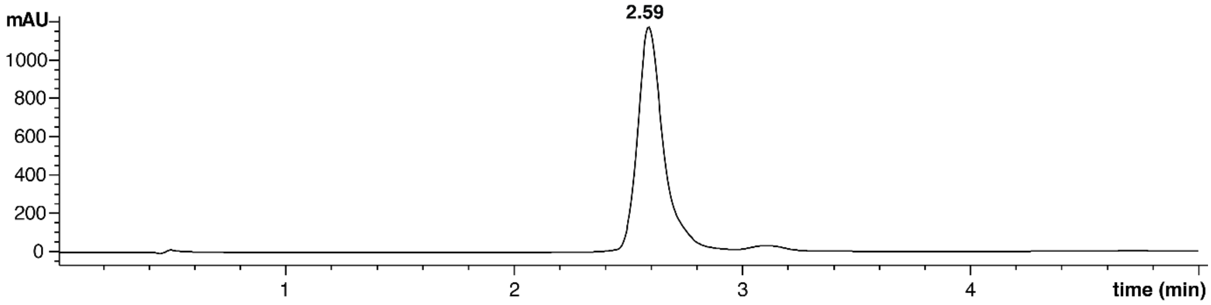

LC chromatogram of compound **L1** (mAU at λ = 260 nm). Method E.

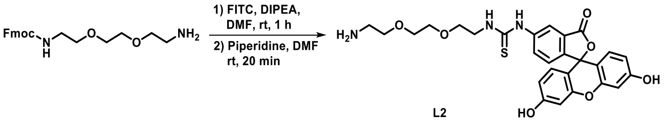

(9*H*-fluoren-9-yl)methyl (2-(2-(2-aminoethoxy)ethoxy)ethyl)carbamate (200.00 mg, 0.53 mmol, 1.00 equiv.) was loaded in the reaction vessel and dissolved in DMF (3 mL). DIPEA (185.71 µL, 1.06 mmol, 2.00 equiv.) and fluorescein isothiocyanate (206.37 mg, 0.53 mmol, 1.00 equiv) were added and reaction was stirred at rt for 1 h. Piperidine (204.33 µL, 2.12 mmol, 4.00 equiv.) was added. The mixture was reacted at rt for 20 min and compound **L2** was directly purified *via* preparative RP-HPLC (Method B).

L2: 57.02 mg, 0.11 mmol, 20% yield.

^{1}H NMR (400 MHz, DMSO-d_6) δ 10.31 (s, 1H), 8.35 (t, J = 5.2 Hz, 1H), 8.31 (s, 1H), 7.84 (bs, 2H), 7.76 (d, J = 8.4, 1H), 7.19 (d, J = 8.3 Hz, 1H), 6.69 (d, J = 2.2 Hz, 2H), 6.59 (s, 1H), 6.58 (d, J = 2.2 Hz, 1H), 3.72 – 3.65 (m, 2H), 3.64 – 3.57 (m, 7H), 2.99 (p, J = 5.6 Hz, 2H).

HRMS (ESI, m/z) calcd. for $C_{27}H_{27}N_3O_7S$: [M + H]$^+$ 538.16425, detected: [M + H]$^+$ 538.16412.

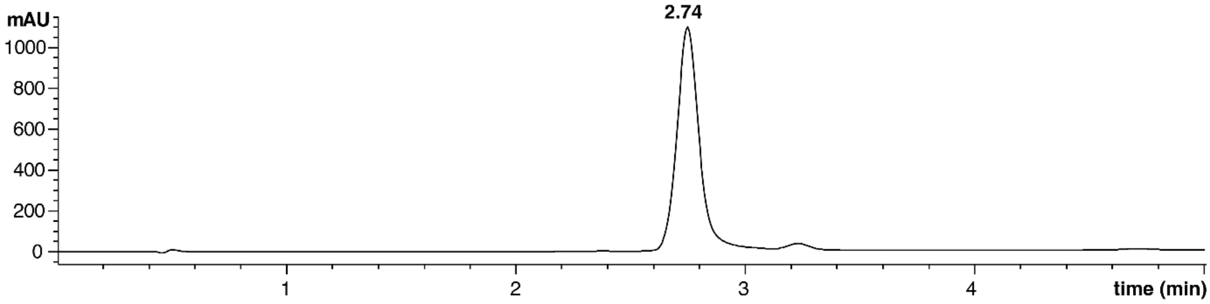

LC chromatogram of compound **L2** (mAU at λ = 260 nm). Method E.

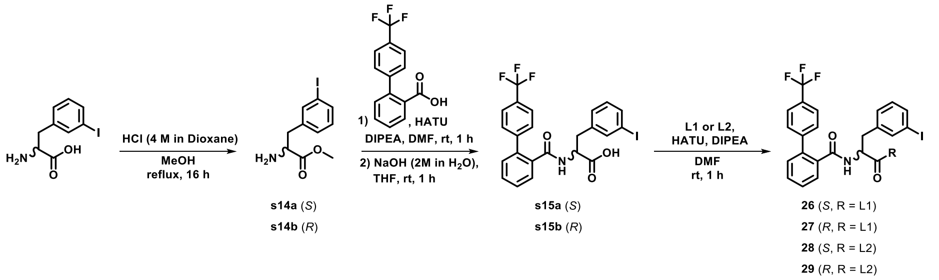

(*S*)-2-amino-3-(3-iodophenyl)propanoic acid and (*R*)-2-amino-3-(3-iodophenyl)propanoic acid (250 mg, 0.86 mmol, 1.00 equiv.) were loaded into the respective reaction vessel and suspended in dry MeOH (10 mL). A solution of HCl 4M in dioxane (0.9 mL, 3.44 mmol, 4.00 equiv.) was added dropwise to the reactions. The mixtures were heated to reflux for 16 h. The solvents were removed under reduced pressure, and the crudes were directly used for further reactions.

s14a,b m/z calculated for $C_{10}H_{13}INO_2$ [M + H]$^+$ 305.99; detected: 306.0.

4'-(trifluoromethyl)-[1,1'-biphenyl]-2-carboxylic acid (150.0 mg, 0.56 mmol, 1.00 equiv.) was loaded in two different reaction vessels and dissolved in DMF (2 mL). DIPEA (197.59 µL, 1.12 mmol, 2.00 equiv.)

and HATU (212.80 mg, 0.56 mmol, 1.00 equiv.) were added to the reaction vessels and the mixture were allowed to proceed at rt for 10 min. The intermediates **s14a,b** (170.24 *µ*L, 0.56 mmol, 1.00 equiv.) were separately added to the respective reaction vessel and reacted at rt for 50 min. An aqueous solution of NaOH 2M (0.60 mL, 1.12 mmol, 2.00 equiv.) was added to the reactions and mixtures were stirred at rt for 1 h. A water solution of HCl 2M was added to reach pH ~ 3 to quench the reactions. The mixtures were diluted with EtOAc (5 mL) and washed with water (3 x 10 mL). The organic layers were dried over anhydrous sodium sulfate, filtered, and concentrated under reduced pressure. The products were purified by RP-HPLC (Method B) and obtained as pale-yellow solids after lyophilization.

s15a (*S*): 136.08 mg, 0.25 mmol, 45 % yield.

^{1}H NMR (400 MHz, DMSO-d_6) δ 8.95 (d, *J* = 8.5 Hz, 1H), 7.76 (t, *J* = 1.7 Hz, 1H), 7.66 (dt, *J* = 7.9, 1.4 Hz, 1H), 7.58 - 7.51 (m, 3H), 7.49 (td, *J* = 7.5, 1.4 Hz, 1H), 7.46 - 7.40 (m, 3H), 7.37 - 7.32 (m, 2H), 7.15 (t, *J* = 7.8 Hz, 1H), 4.48 (ddd, *J* = 11.2, 8.5, 4.0 Hz, 1H), 3.16 (dd, *J* = 13.7, 4.1 Hz, 1H), 2.88 (dd, *J* = 13.7, 11.3 Hz, 1H).

HRMS (ESI, m/z) calcd. for $C_{23}H_{17}F_3INO_3$ $[M + H]^+$ 540.02779; detected: $[M + H]^+$ 540.02774.

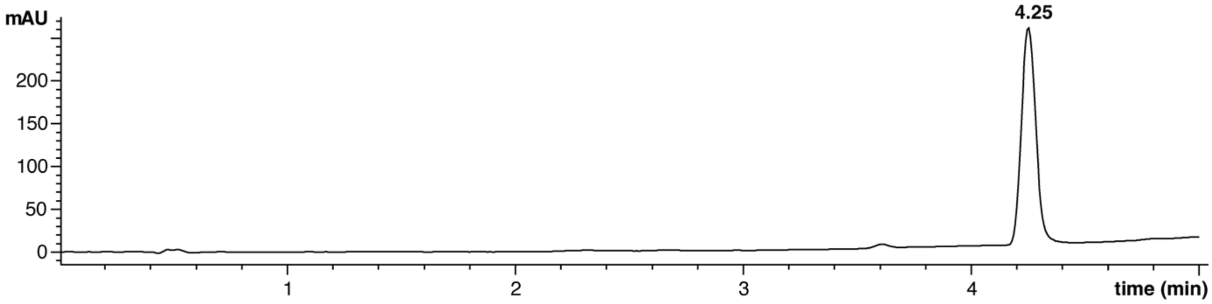

LC chromatogram of compound **s15a** (mAU at λ = 260 nm). Method D.

s15b (*S*): 142.13 mg, 0.26 mmol, 45 % yield.

^{1}H NMR (400 MHz, DMSO-d_6) δ 8.95 (d, *J* = 8.5 Hz, 1H), 7.76 (t, *J* = 1.7 Hz, 1H), 7.66 (dt, *J* = 7.9, 1.4 Hz, 1H), 7.58 - 7.51 (m, 3H), 7.49 (td, *J* = 7.5, 1.4 Hz, 1H), 7.46 - 7.40 (m, 3H), 7.37 - 7.32 (m, 2H), 7.15 (t, *J* = 7.8 Hz, 1H), 4.48 (ddd, *J* = 11.2, 8.5, 4.0 Hz, 1H), 3.16 (dd, *J* = 13.7, 4.1 Hz, 1H), 2.88 (dd, *J* = 13.7, 11.3 Hz, 1H).

HRMS (ESI, m/z) calcd. for $C_{23}H_{17}F_3INO_3$: $[M + H]^+$ 540.02779; detected: $[M + H]^+$ 540.02760.

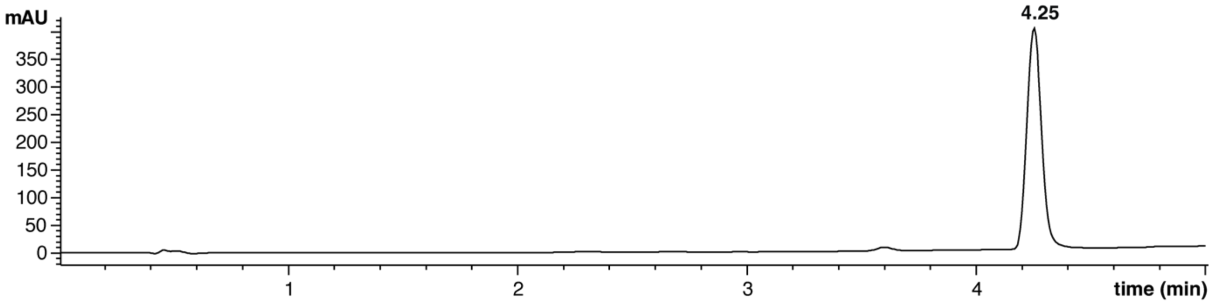

LC chromatogram of compound **s15b** (mAU at λ = 260 nm). Method D.

Intermediate **s15a,b** (5.00 mg, 9.25 µmol, 1.00 equiv.) were separately dissolved in dry DMF (0.5 mL). DIPEA (6.48 µL, 37.04 µmol, 4.00 equiv) and HATU (3.52 mg , 9.25 µmol, 1.00 equiv) were added and the mixtures were stirred at rt for 10 minutes. After this time, intermediate **L1** (5.94 mg, 11.10 mmol, 1.2 equiv.) or **L2** (5.96 mg, 11.10 mmol, 1.2 equiv.) were separately added to the corresponding mixtures and the reactions were stirred at rt for 50 min. The crude mixtures were diluted with DMF (0.5 mL) and the products were purified *via* RP-HPLC (Method C) with the obtainment of red solids after lyophilization.

26 (*S*, R = L1): 2.44 mg, 2.31 µmol, 25% yield.

HRMS (ESI, m/z) calcd. for $C_{50}H_{40}F_3IN_4O_9S$: [M + H]$^+$ 1057.15855; detected: [M + H]$^+$ 1057.15925.

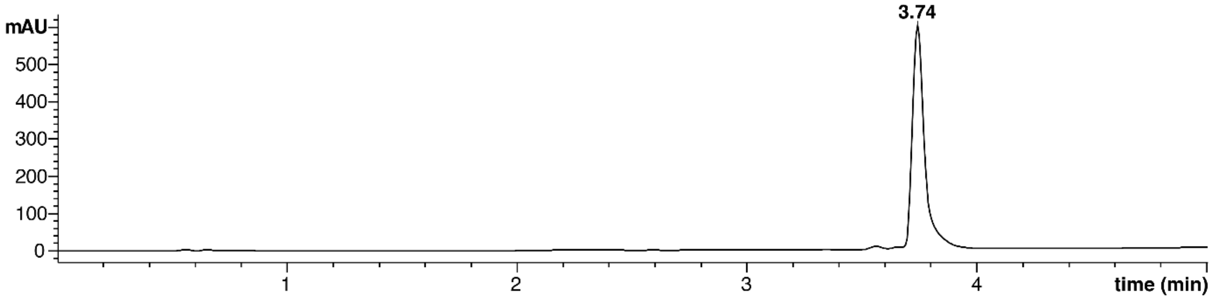

LC chromatogram of compound **26** (mAU at λ = 260 nm). Method E.

27 (*R*, R = L1): 2.05 mg, 1.94 µmol, 21% yield.

HRMS (ESI, m/z) calcd. for $C_{50}H_{40}F_3IN_4O_9S$: [M + H]$^+$ 1057.15855; detected: [M + H]$^+$ 1057.15783.

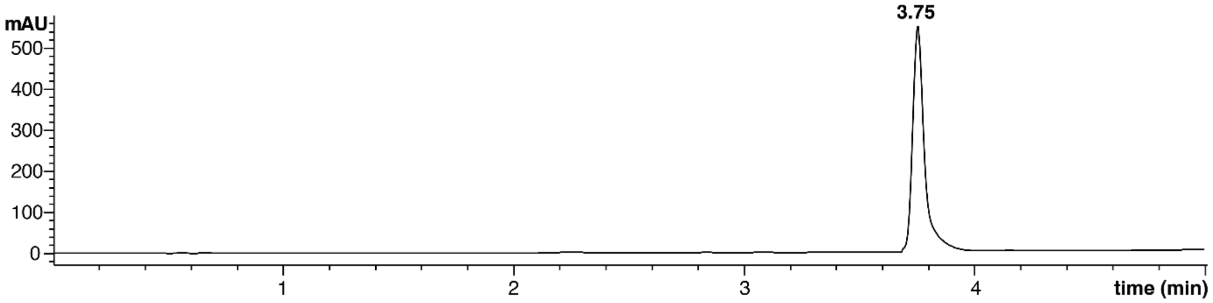

LC chromatogram of compound **27** (mAU at λ = 260 nm). Method E.

28 (*S*, R = L2): 2.25 mg, 2.13 µmol, 23% yield.

HRMS (ESI, m/z) calcd. for $C_{50}H_{42}F_3IN_4O_9S$: [M + H]$^+$ 1059.17420; detected: [M + H]$^+$ 1059.17413.

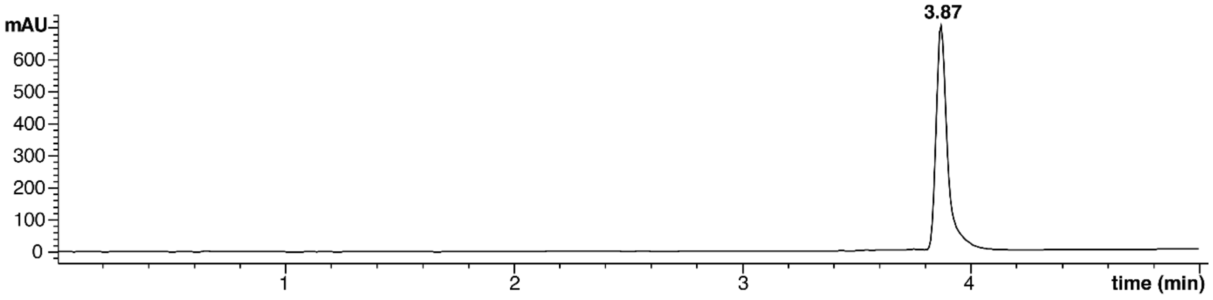

LC chromatogram of compound **28** (mAU at λ = 260 nm). Method E.

29 (*R*, R = L2): 2.64 mg, 2.50 µmol, 27% yield.

HRMS (ESI, m/z) calcd. for $C_{50}H_{42}F_3IN_4O_9S$: $[M + H]^+$ 1059.17420; detected: $[M + H]^+$ 1059.17418.

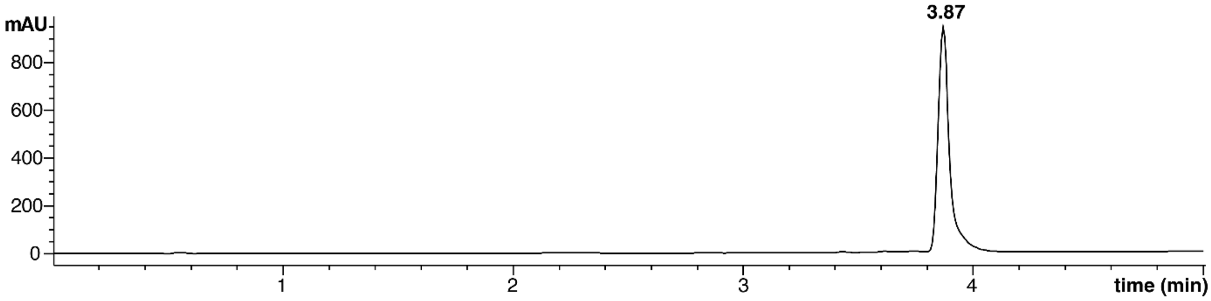

LC chromatogram of compound **29** (mAU at λ = 260 nm). Method E.

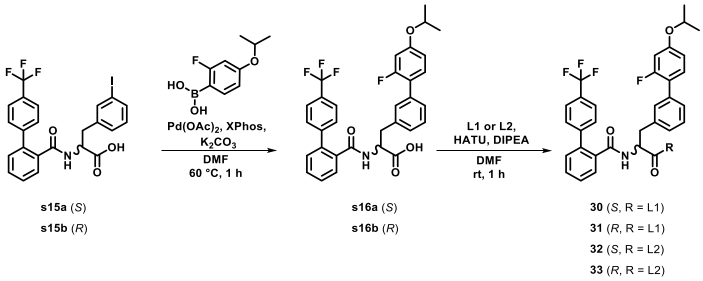

Intermediates **s15a,b** (100.00 mg, 0.19 mmol, 1.00 equiv.) were loaded in two separate reaction vessels and dissolved in dry DMF (2 mL). K_2CO_3 (104.88 mg, 0.76 mmol, 4.00 equiv), $Pd(OAc)_2$ (8.53 mg, 0.04 mmol, 0.20 equiv), XPhos (36.23 mg, 0.08 mmol, 0.40 equiv) and (2-fluoro-4-isopropoxyphenyl)boronic acid (56.43 mg, 0.29 mmol, 1.50 equiv.) were added to the mixtures. The reactions were stirred at 60 °C for 1 h. After this time, the mixtures were diluted with EtOAc (5 mL) and washed with water (3 x 10 mL). The organic layers were dried over anhydrous sodium sulfate, filtered, and concentrated under reduced pressure. the products were purified *via* RP-HPLC (Method B) to obtain pale-yellow solids after lyophilization.

s16a (*S*): 78.53 mg, 0.14 mmol, 73% yield.

^{1}H NMR (400 MHz, DMSO-d_6) δ 8.95 (d, *J* = 8.4 Hz, 1H), 7.56 - 7.47 (m, 4H), 7.47 - 7.37 (m, 7H), 7.33 - 7.27 (m, *J* = 7.7, 2.8, 1.4 Hz, 2H), 6.94 - 6.81 (m, 2H), 4.68 (hept, *J* = 6.0 Hz, 1H), 4.55 (ddd, *J* = 11.1, 8.4, 4.1 Hz, 1H), 3.24 (dd, *J* = 13.7, 4.1 Hz, 1H), 2.97 (dd, *J* = 13.8, 11.1 Hz, 1H), 1.28 (d, *J* = 6.0 Hz, 6H).

HRMS (ESI, m/z) calcd. for $C_{32}H_{27}F_4NO_4$: $[M + H]^+$ 566.19490, detected: $[M + H]^+$ 566.19437.

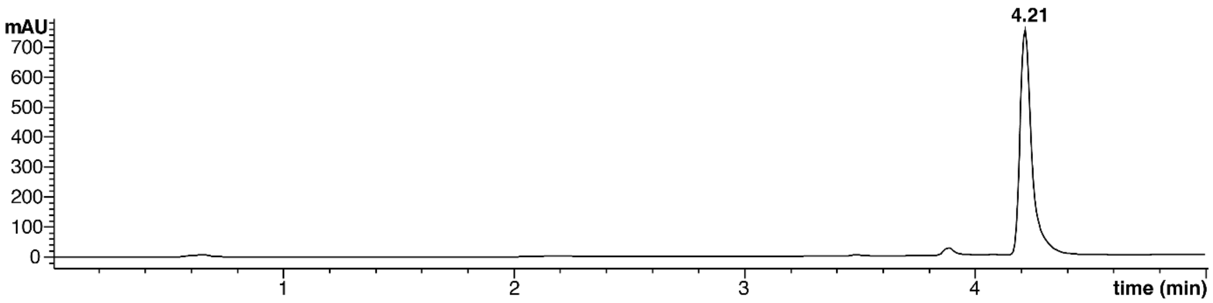

LC chromatogram of compound **s16a** (mAU at λ = 260 nm). Method D.

s16b (*R*): 72.05 mg, 0.13 mmol, 67% yield.

^{1}H NMR (400 MHz, DMSO-d_6) δ 8.95 (d, *J* = 8.4 Hz, 1H), 7.56 - 7.47 (m, 4H), 7.47 - 7.37 (m, 7H), 7.33 - 7.27 (m, *J* = 7.7, 2.8, 1.4 Hz, 2H), 6.94 - 6.81 (m, 2H), 4.68 (hept, *J* = 6.0 Hz, 1H), 4.55 (ddd, *J* = 11.1, 8.4, 4.1 Hz, 1H), 3.24 (dd, *J* = 13.7, 4.1 Hz, 1H), 2.97 (dd, *J* = 13.8, 11.1 Hz, 1H), 1.28 (d, *J* = 6.0 Hz, 6H).

HRMS (ESI, m/z): calcd. for $C_{32}H_{27}F_4NO_4$: $[M + H]^+$ 566.19490, detected: $[M + H]^+$ 566.19466

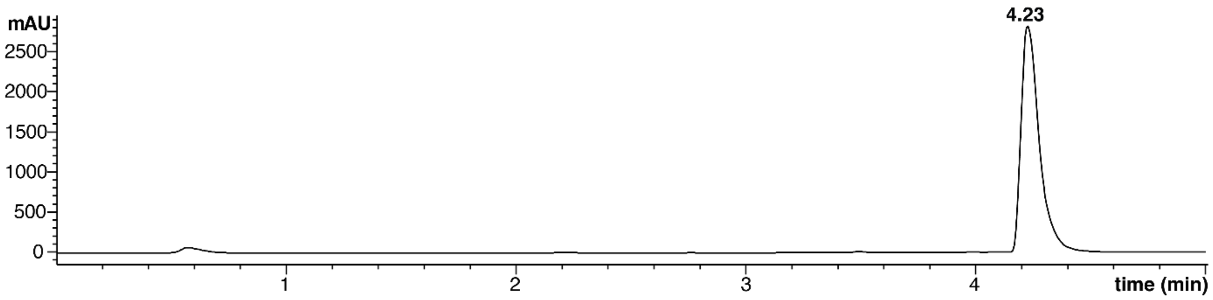

LC chromatogram of compound **s16b** (mAU at λ = 260 nm). Method D.

Intermediate **s16a,b** (5.00 mg, 8.84 µmol, 1.00 equiv.) were dissolved in dry DMF (0.5 mL). DIPEA (6.20 µL, 35.39 µmol, 4.00 equiv) and HATU (3.35 mg, 8.85 µmol, 1.00 equiv) were added and the reactions were allowed to proceed at rt for 10 min. Intermediate **L1** (5.96 mg, 10.61 mmol, 1.2 equiv.) or **L2** (5.71 mg, 10.61 mmol, 1.2 equiv.) were separately added to the corresponding mixtures and the reactions were reacted at rt for 50 min. The crude mixtures were separately diluted with DMF (0.5 mL) and the products were purified *via* RP-HPLC (Method C) to red solids after lyophilization.

30 (*S*, R = L1): 2.97 mg, 2.74 µmol, 31% yield.

HRMS (ESI, m/z) calcd. for $C_{59}H_{50}F_4N_4O_{10}S$: $[M + H]^+$ 1083.32566; detected: $[M + H]^+$ 1083.32496.

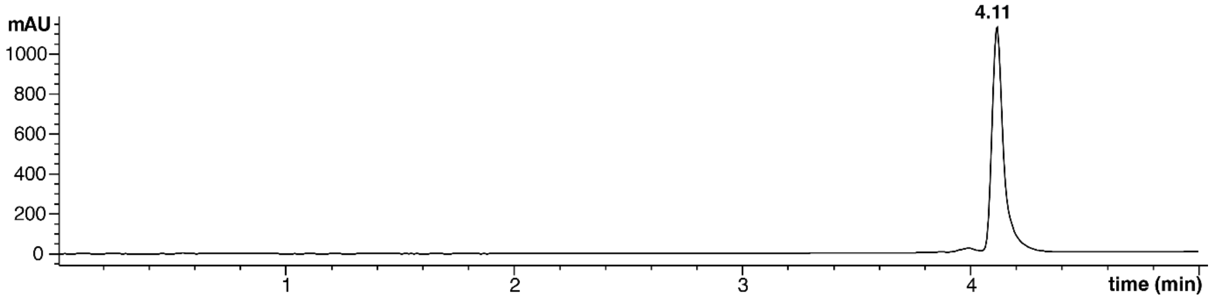

LC chromatogram of compound **30** (mAU at λ = 260 nm). Method E.

31 (*R*, R = L1): 3.54 mg, 3.27 µmol, 37% yield.

HRMS (ESI, m/z) calcd. for $C_{59}H_{50}F_4N_4O_{10}S$: $[M + H]^+$ 1083.32566; detected: $[M + H]^+$ 1083.32418.

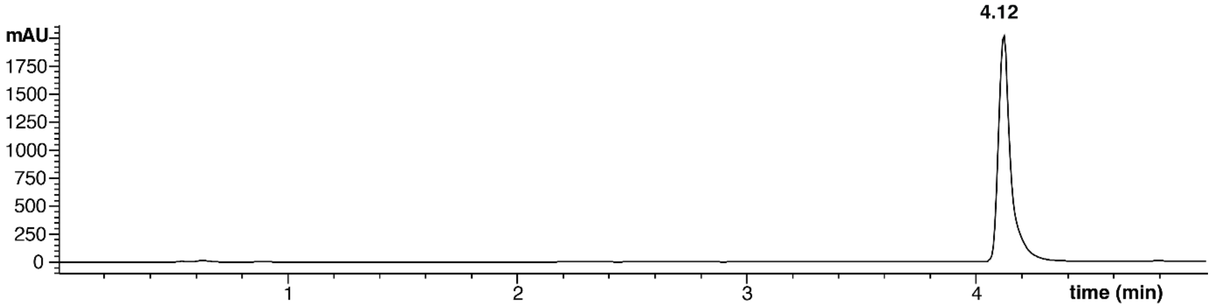

LC chromatogram of compound **31** (mAU at λ = 260 nm). Method E.

32 (*S*, R = L2): 2.30 mg, 2.12 µmol, 24% yield.

HRMS (ESI, m/z) calcd. $C_{59}H_{52}F_4N_4O_{10}S$: $[M + H]^+$ 1085.34131; detected: $[M + H]^+$ 1085.33909 .

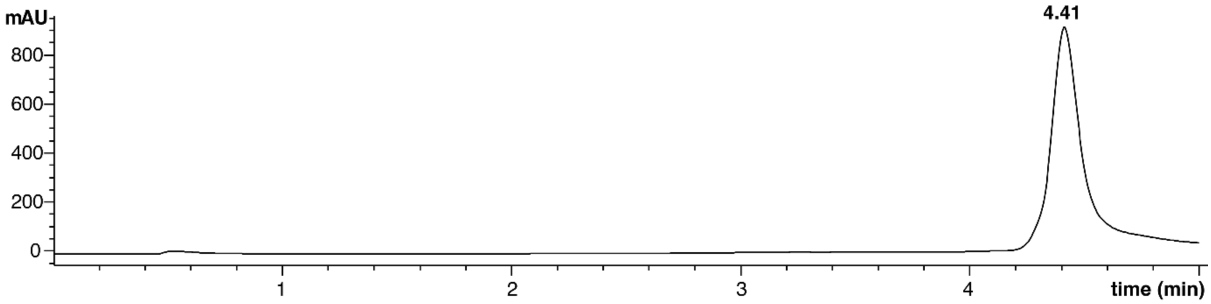

LC chromatogram of compound **32** (mAU at λ = 260 nm). Method E.

33 (*R*, R = L2): 2.78 mg, 2.56 µmol, 29% yield.

HRMS (ESI, m/z) calcd. $C_{59}H_{52}F_4N_4O_{10}S$ $[M + H]^+$ 1085.34131; detected: $[M + H]^+$ 1085.33909.

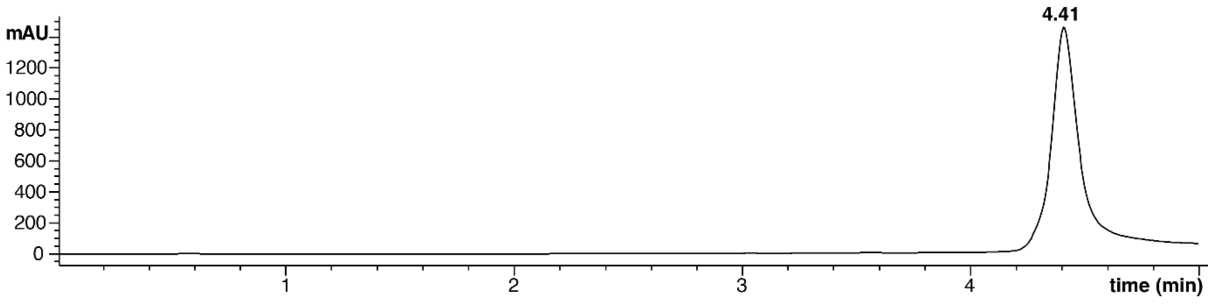

LC chromatogram of compound **33** (mAU at λ = 260 nm). Method E.

5.3.5 Radiosynthesis of PSMA and CAIX hit compounds

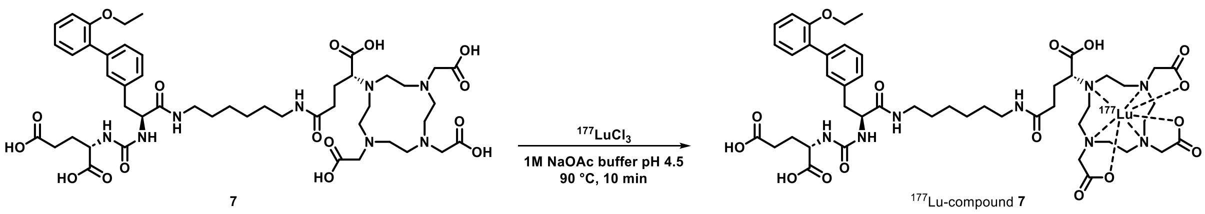

^{177}Lu-compound **7** was prepared from product **7** following the procedure reported in **Autoradiography experiments**. Labelling was performed at the molar activity of 20 MBq/nmol, and the products were used in autoradiography experiments.

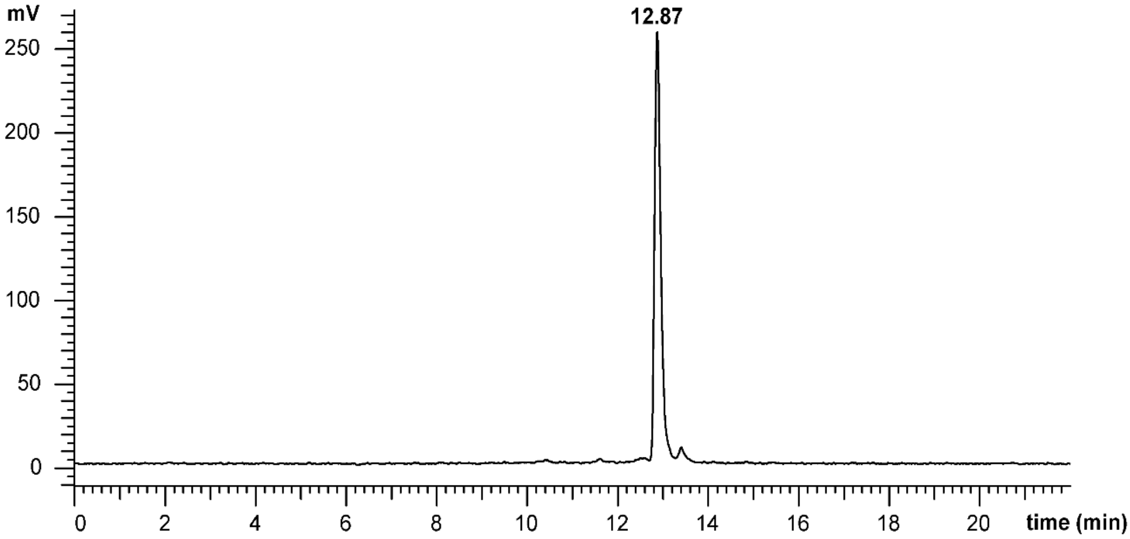

HPLC trace of ^{177}Lu-compound **7** as recorded with a radio-detector.

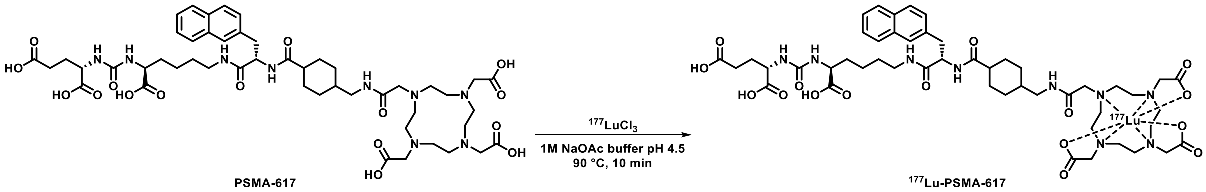

^{177}Lu-PSMA-617 was prepared from **PSMA-617** following the procedure reported in **Autoradiography experiments**. Labelling was performed at the molar activity of 20 MBq/nmol, and the products were used in autoradiography experiments.

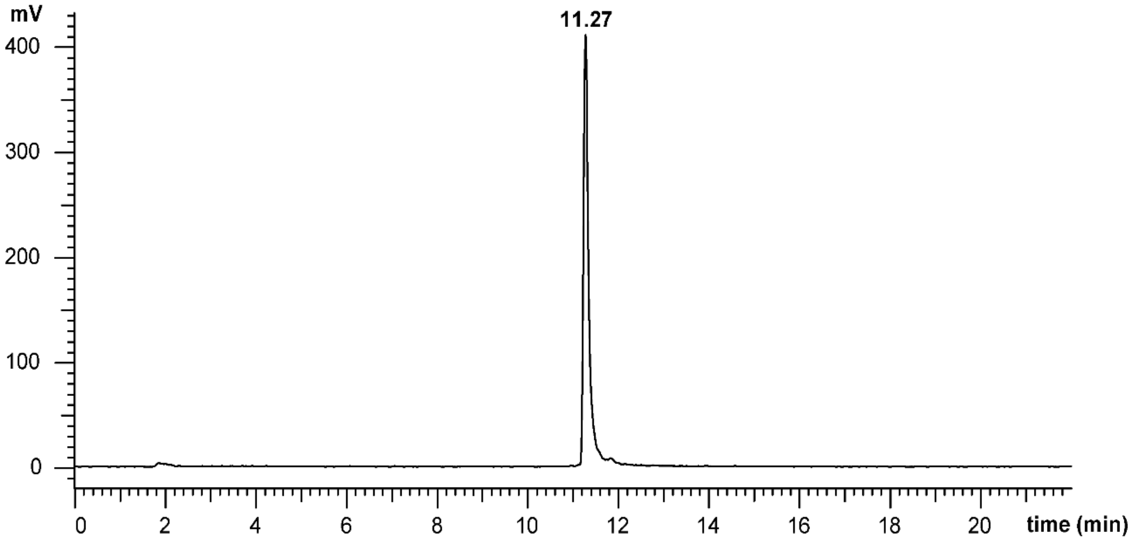

HPLC trace of ^{177}**Lu-PSMA-617** as recorded with a radio-detector.

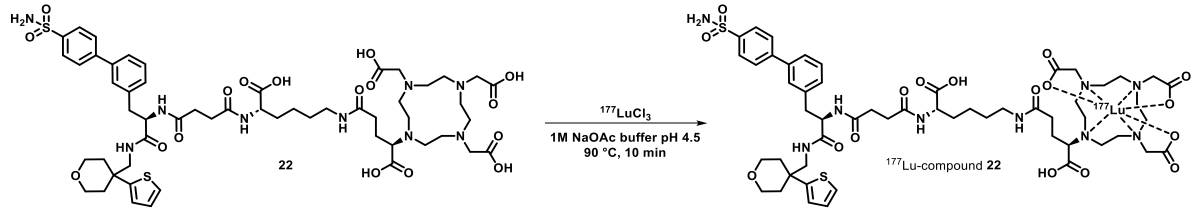

^{177}Lu-compound **22** was prepared *via* **GP7** from compound **22**. Labelling was performed at the molar activity of 3.33 MBq/nmol, and the products were used in biodistribution experiments.

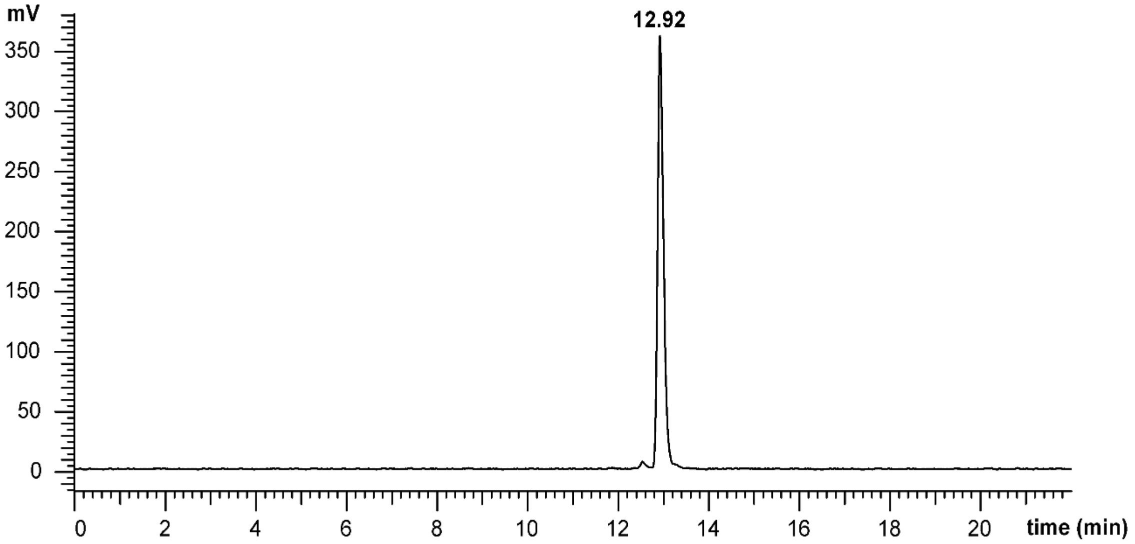

HPLC trace of ^{177}Lu-compound **22** as recorded with a radio-detector.

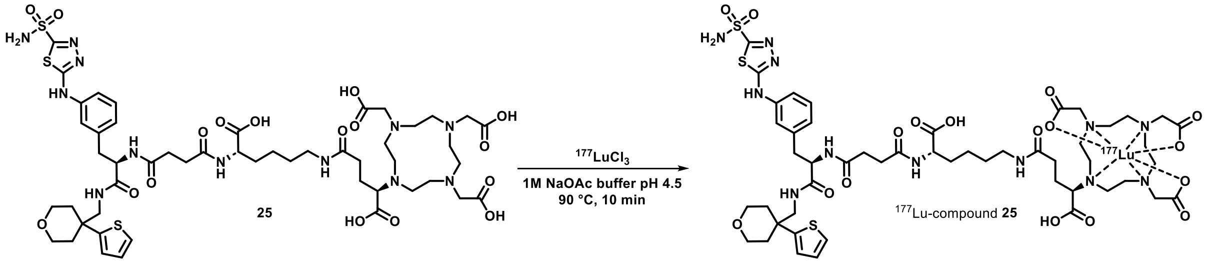

^{177}Lu-compound **25** was prepared *via* **GP7** from compound **25**. Labelling was performed at the molar activity of 3.33 MBq/nmol, and the products were used in biodistribution experiments.

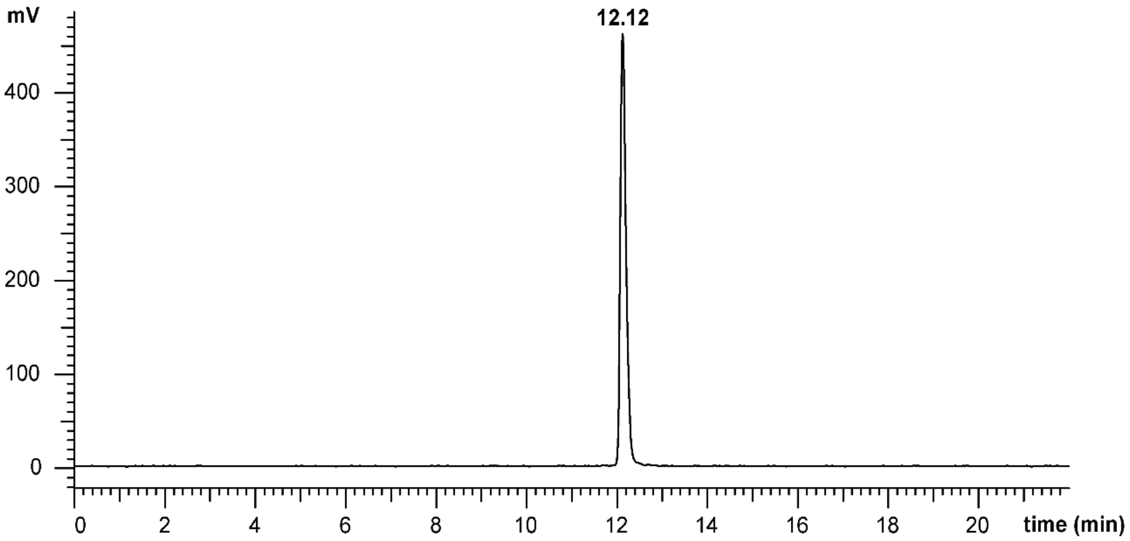

HPLC trace of ^{177}Lu-compound **25** as recorded with a radio-detector.

6 Supplementary results

6.1 DEL-selection fingerprints

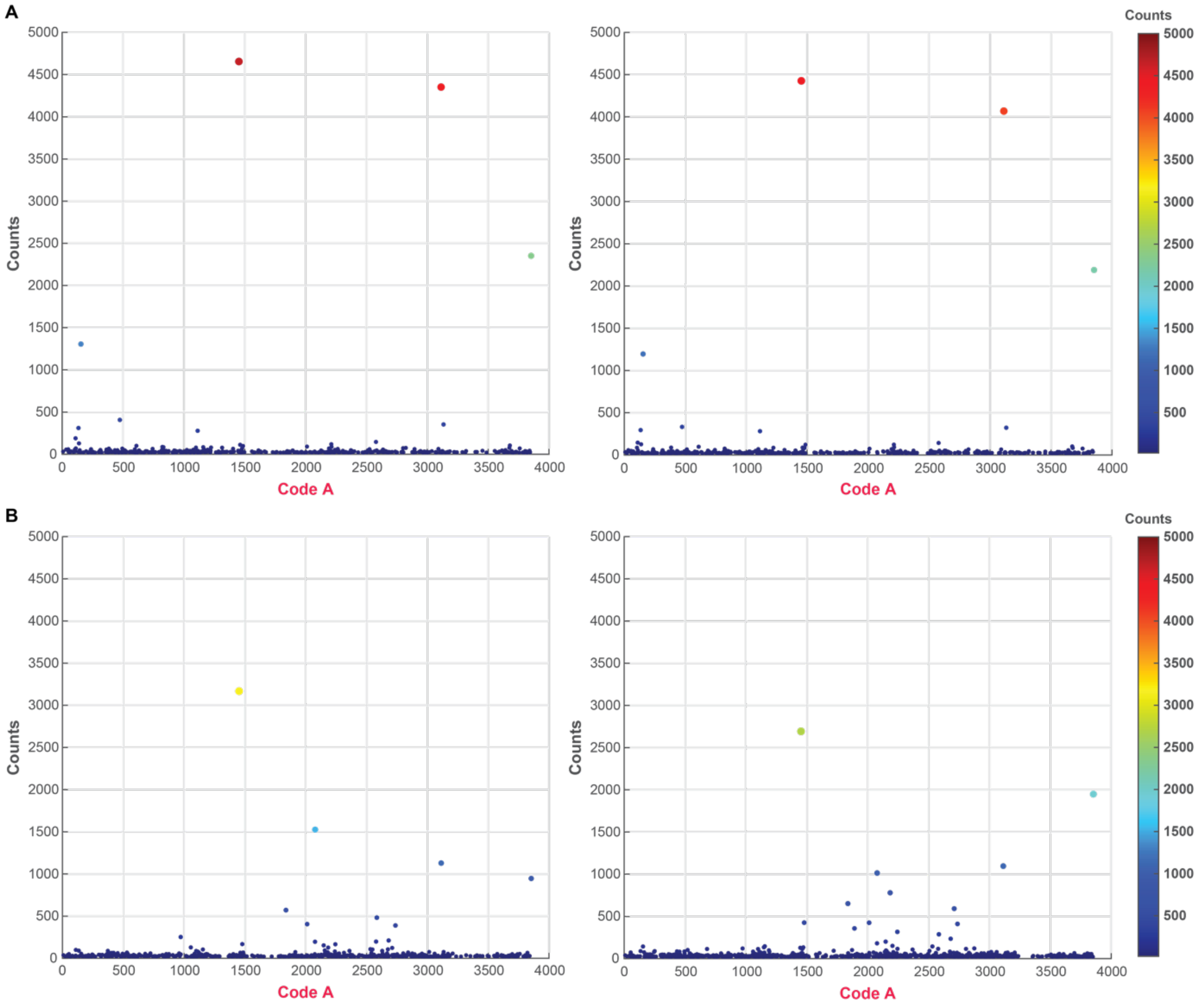

Figure S20. *Selection fingerprints after screening of FM-DEL1 against PSMA (**A**) and GCP3 (anti-target, **B**). DEL selections were performed in duplicates. Dot color and size correspond to the normalized sequence counts. **A**, left: TCs = 51'427, AC = 13, Cut-off = 20; **A** right: TCs = 48'965, AC = 13, Cut-off = 20; **B**, left: TCs = 49'445, AC = 13, Cut-off = 20; **B**, right: TCs = 63'841, AC = 17, Cut-off = 20.*

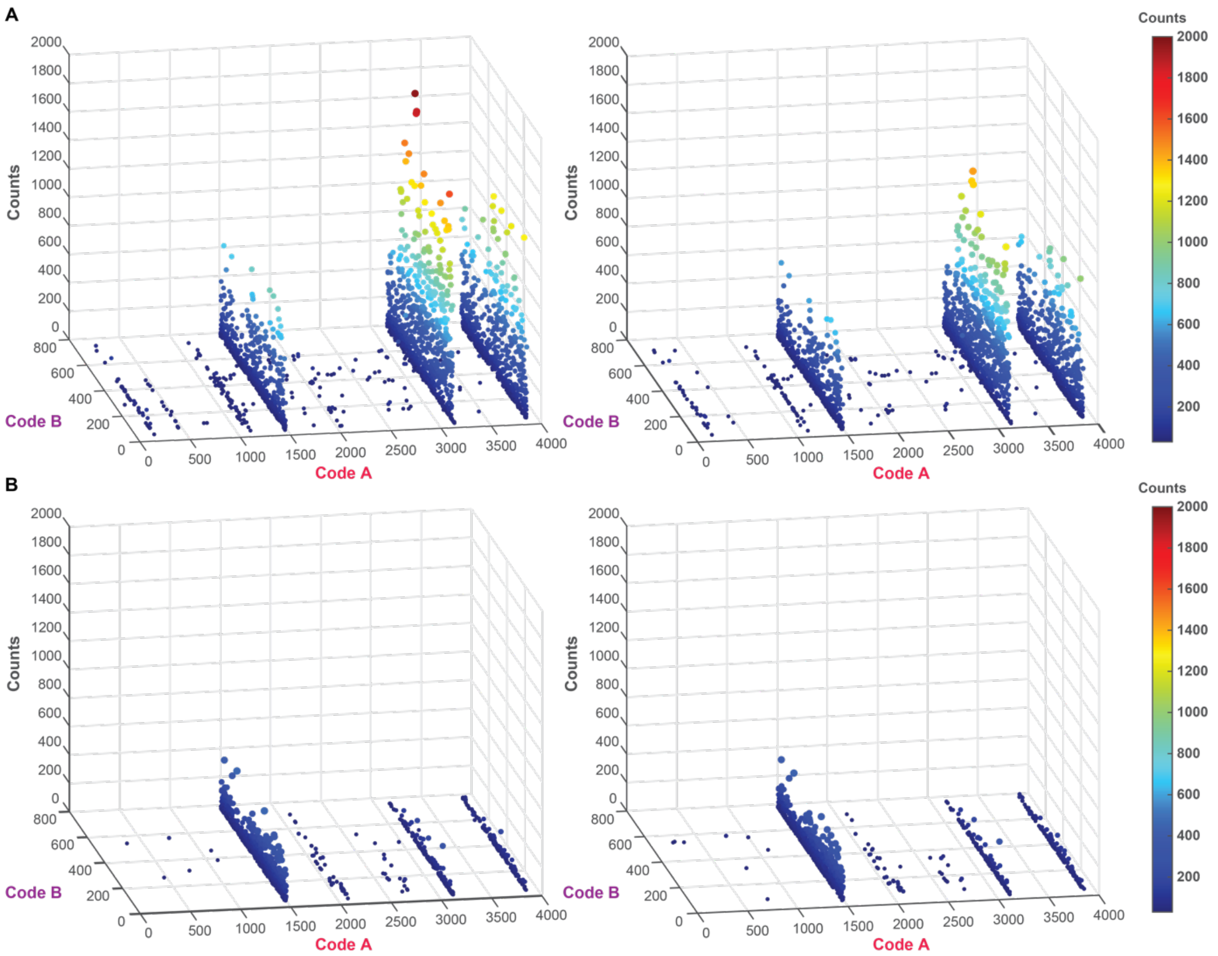

Figure S21. *Selection fingerprints after screening of FM-DEL2 against PSMA (**A**) and GCP3 (anti-target, **B**). DEL selections were performed in duplicates. Dot color and size correspond to the normalized sequence counts. **A**, left: TCs = 2'389'572, AC = 0.84, Cut-off = 30; **A** right: TCs = 2'130'130, AC = 0.75, Cut-off = 30; **B**, left: TCs = 2'440'903, AC = 0.86, Cut-off = 30; **B**, right: TCs = 2'407'360, AC = 0.85, Cut-off = 30.*

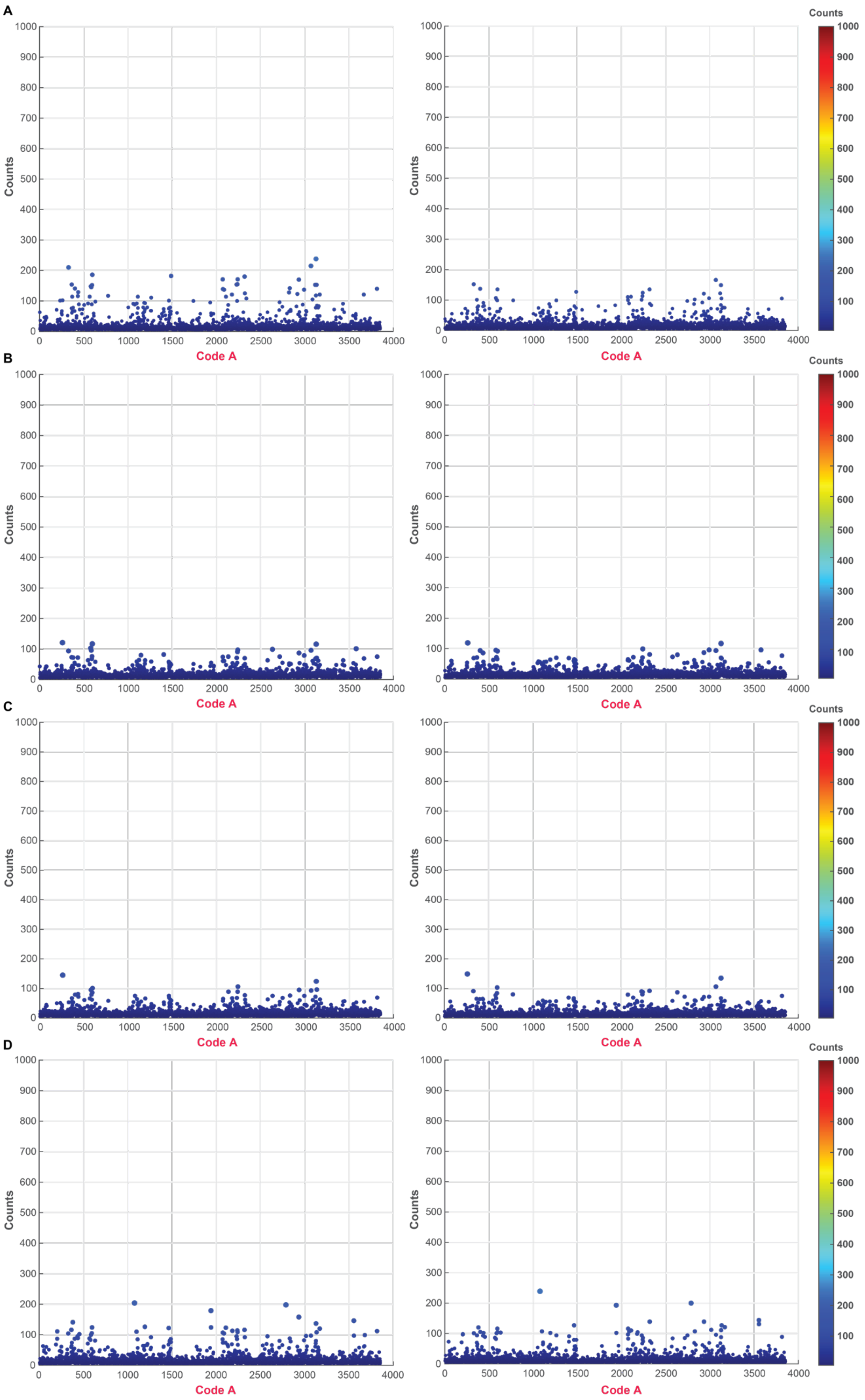
A
B
C
D
Counts
Code A
0
500
1000
1500
2000
2500
3000
3500
4000
100
200
300
400
500
600
700
800
900
1000

Figure S22. *Selection fingerprints after screening of FM-DEL1 against hACP3 (**A**), mACP3 (**B**), H44A, H289A hACP3 (**C**) and TNAP (anti-target, **D**). DEL selections were performed in duplicates. Dot color and size correspond to the normalized sequence counts. **A**, left: TCs = 59'011, AC = 15, Cut-off = 5; **A** right: TCs = 55'164, AC = 14, Cut-off = 5; **B**, left: TCs = 52'270, AC = 14, Cut-off = 5; **B**, right: TCs = 52'860, AC = 14, Cut-off = 5; **C**, left: TCs = 55'642, AC = 14, Cut-off = 5; **C** right: TCs = 51'123, AC = 13, Cut-off = 5; **D**, left: TCs = 50'359, AC = 13, Cut-off = 5; **D**, right: TCs = 46'495, AC = 12, Cut-off = 5.*

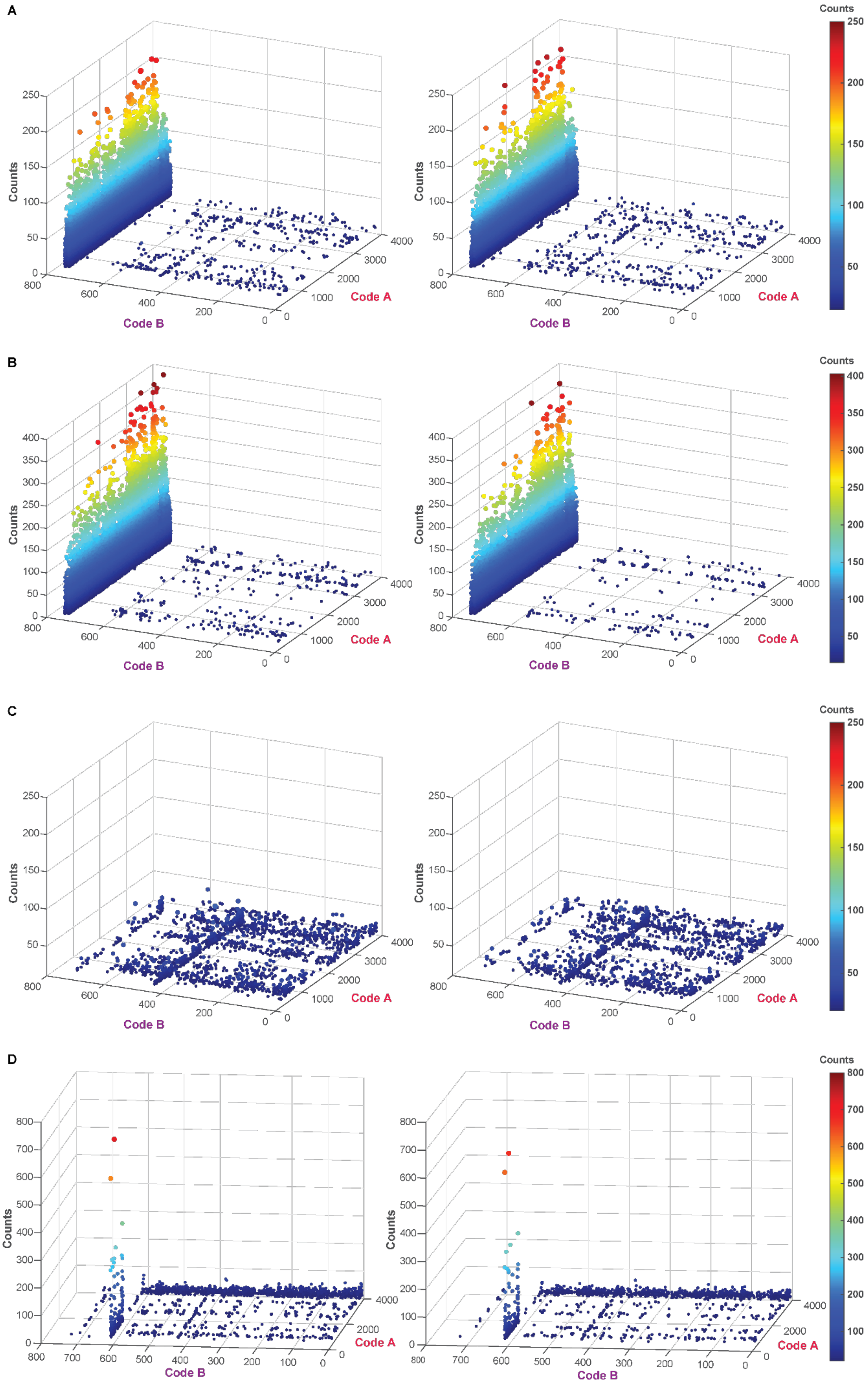
A
Counts
Code A
Code B
0
50
100
150
200
250
800
600
400
200
0
1000
2000
3000
4000
B
Counts
Code A
Code B
0
50
100
150
200
250
300
350
400
800
600
400
200
0
1000
2000
3000
4000
C
Counts
Code A
Code B
50
100
150
200
250
800
600
400
200
0
1000
2000
3000
4000
D
Counts
Code A
Code B
0
100
200
300
400
500
600
700
800
800
700
600
500
400
300
200
100
0
2000
4000

Figure S23. *Selection fingerprints after screening of FM-DEL2 against hACP3 (**A**), mACP3 (**B**), H44A, H289A hACP3 (**C**) and TNAP (anti-target, **D**). DEL selections were performed in duplicates. Dot color and size correspond to the normalized sequence counts. **A**, left: TCs = 2'254'758, AC = 0.85, Cut-off = 15; **A** right: TCs = 2'250'158, AC = 0.78, Cut-off = 15; **B**, left: TCs = 2'102'747, AC = 0.74, Cut-off = 15; **B**, right: TCs = 2'151'444, AC = 0.76, Cut-off = 15; **C**, left: TCs = 2'466'870, AC = 0.87, Cut-off = 20; **C** right: TCs = 2'419'799, AC = 0.85, Cut-off = 20; **D**, left: TCs = 2'110'399, AC = 0.74, Cut-off = 20; **D**, right: TCs = 2'013'024, AC = 0.71, Cut-off = 20.*

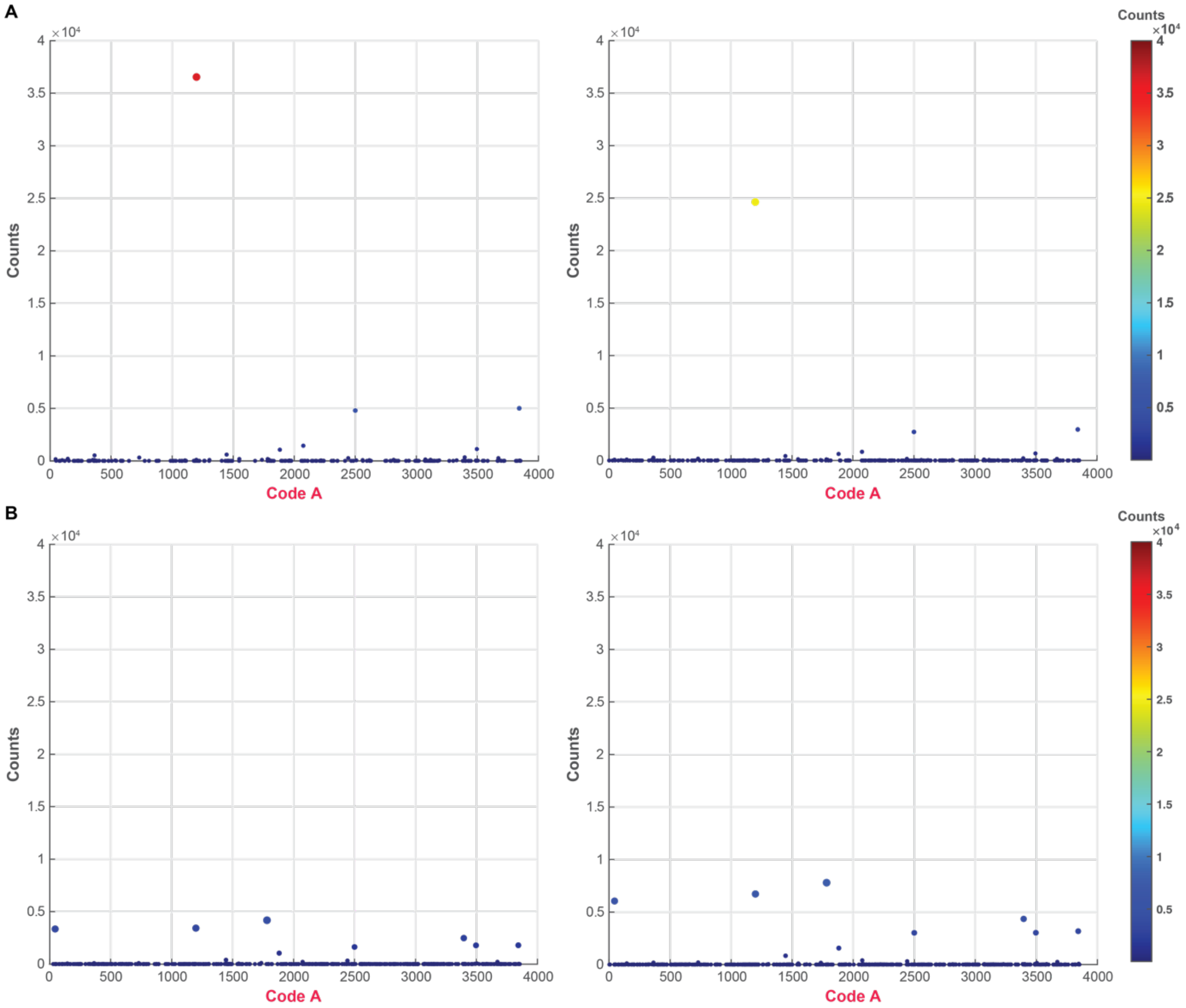

Figure S24. *Selection fingerprints after screening of FM-DEL1 against CAIX (**A**) and CAII (anti-target, **B**). DEL selections were performed in duplicates. Dot color and size correspond to the normalized sequence counts. **A**, left: TCs = 59'564, AC = 16, Cut-off = 5; **A** right: TCs = 41'549, AC = 11, Cut-off = 5; **B**, left: TCs = 30'531, AC = 8, Cut-off = 5; **B**, right: TCs = 47'702, AC = 12, Cut-off = 5.*

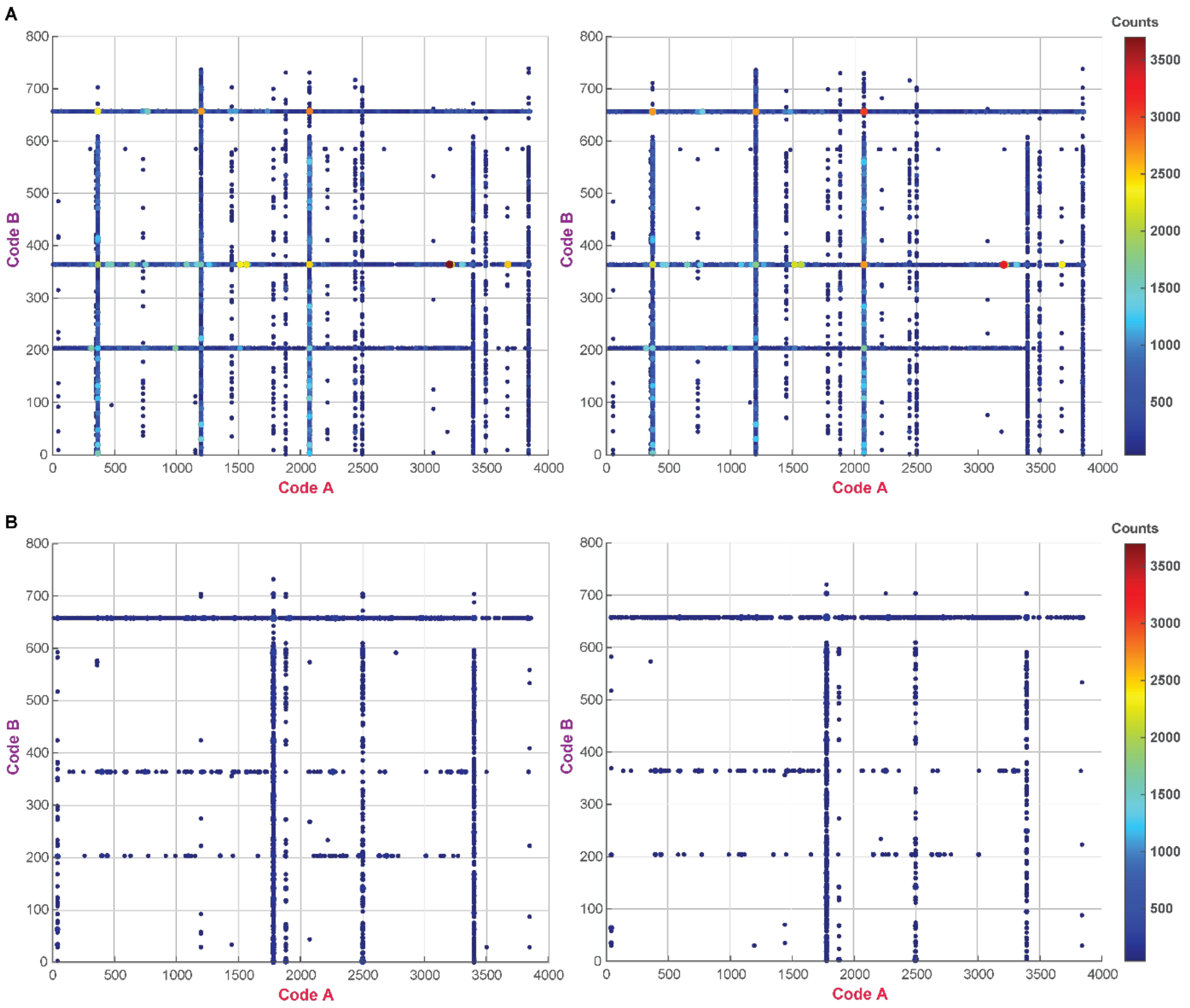

Figure S25. *Selection fingerprints after screening of FM-DEL2 against CAIX (**A**) and CAII (anti-target, **B**). DEL selections were performed in duplicates. Dot color and size correspond to the normalized sequence counts. **A**, left: TCs = 2'546'972, AC = 0.89, Cut-off = 40; **A** right: TCs = 2'477'825, AC = 0.87, Cut-off = 40; **B**, left: TCs = 2'454'675, AC = 0.86, Cut-off = 40; **B**, right: TCs = 2'580'485, AC = 0.91, Cut-off = 40.*

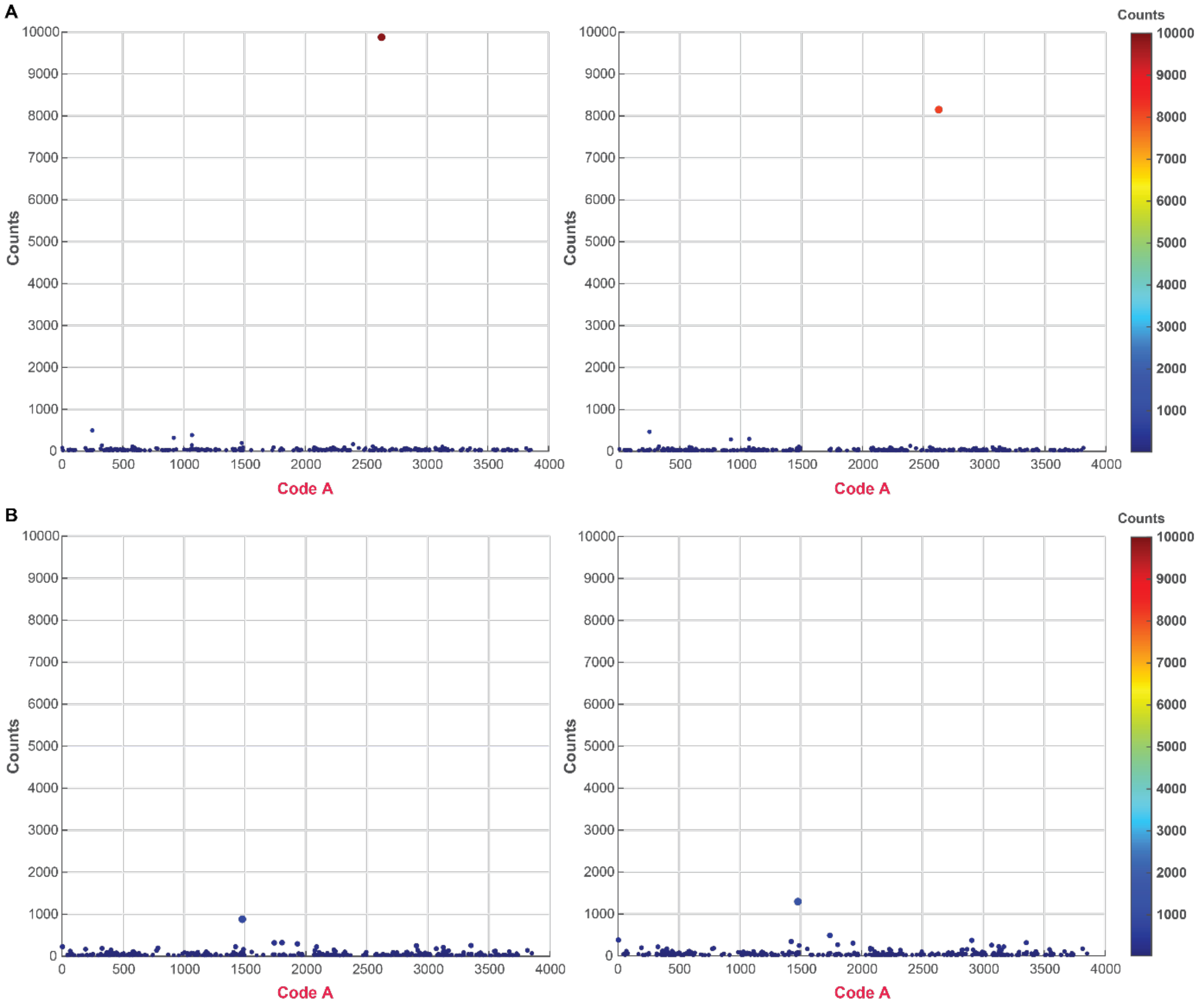

Figure S26. *Selection fingerprints after screening of FM-DEL1 against NKG2D-Fc (**A**) and Fc (negative control, **B**). DEL selections were performed in duplicates. Dot color and size correspond to the normalized sequence counts. **A**, left: TCs = 47'388, AC = 13, Cut-off = 20; **A** right: TCs = 50'204, AC = 13, Cut-off = 20; **B**, left: TCs = 46'872, AC = 12, Cut-off = 20; **B**, right: TCs = 48'930, AC = 13, Cut-off = 20.*

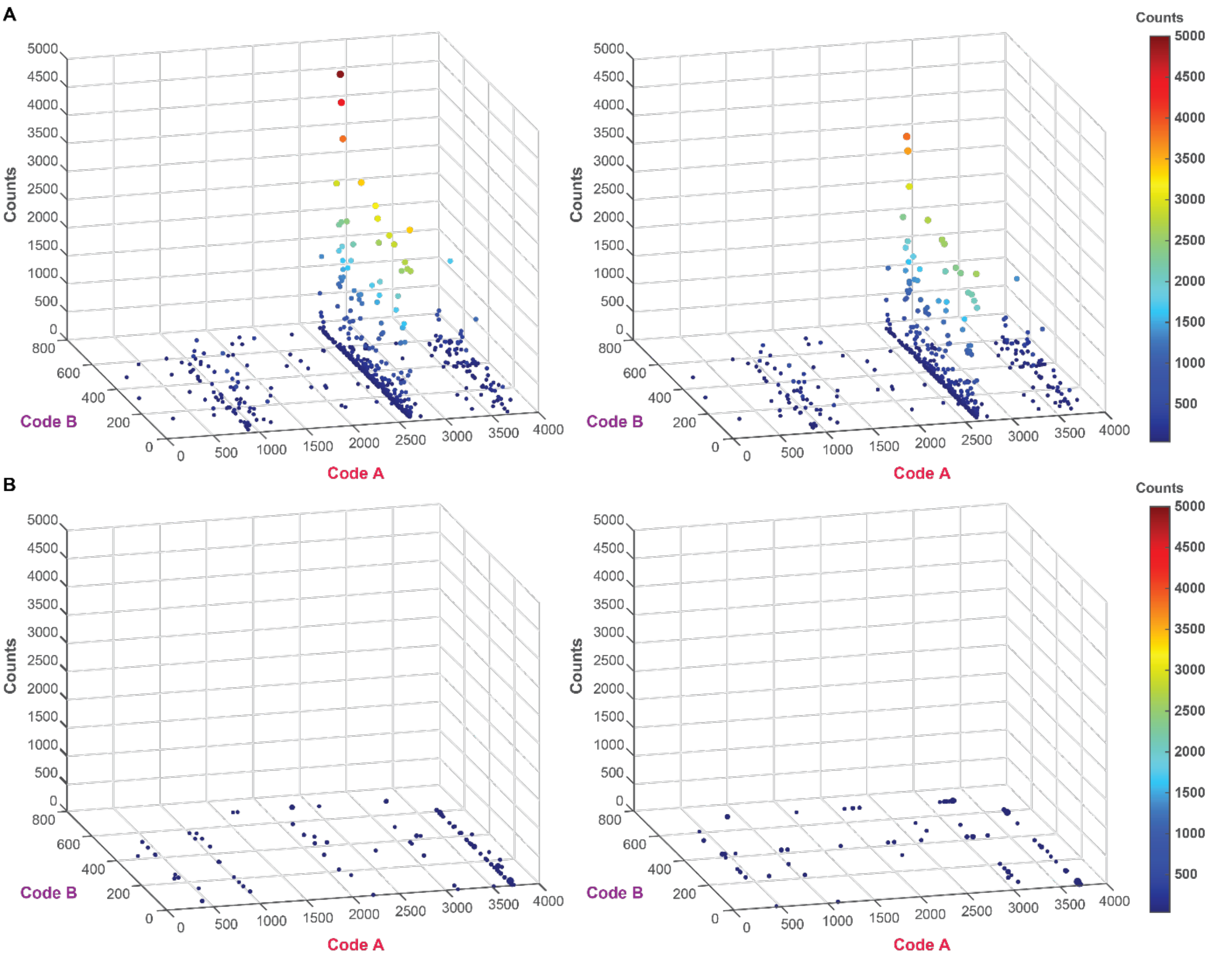

Figure S27. *Selection fingerprints after screening of FM-DEL2 against NKG2D-Fc (**A**) and Fc (negative control, **B**). DEL selections were performed in duplicates. Dot color and size correspond to the normalized sequence counts. **A**, left: TCs = 2'431'707, AC = 0.85, Cut-off = 40; **A** right: TCs = 2'332'639, AC = 0.82, Cut-off = 40; **B**, left: TCs = 2'381'058, AC = 0.84, Cut-off = 25; **B**, right: TCs = 2'282'236, AC = 0.80, Cut-off = 25.*

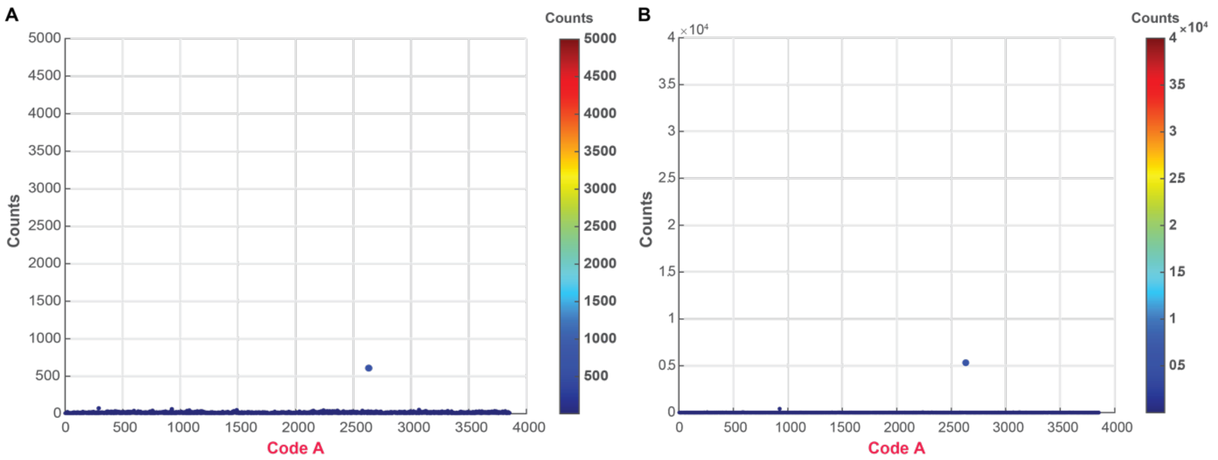

Figure S28. *Selection fingerprints after screening of FM-DEL1 against streptavidin-coated beads (Dynabeads™ MyOne™ Streptavidin C1) in HEPES buffer (no protein, negative control,* ***A****) and streptavidin-coated beads (Dynabeads™ MyOne™ Streptavidin C1) in PBS buffer (no protein, negative control,* ***B****). Dot color and size correspond to the normalized sequence counts.* ***A****: TCs = 49'017, AC = 13, Cut-off = 5;* ***B****: TCs = 39'112, AC = 10, Cut-off = 5.*

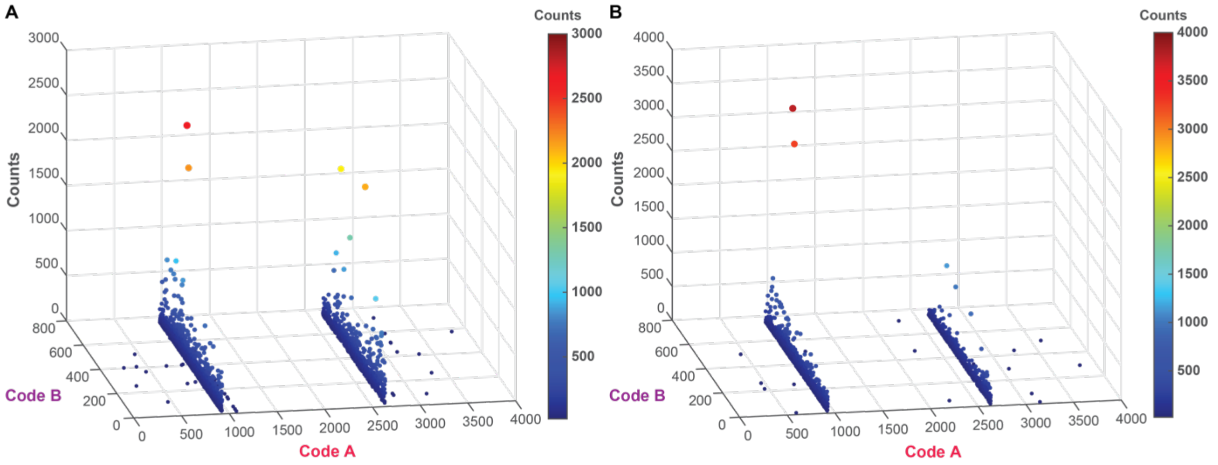

Figure S29. *Selection fingerprints after screening of FM-DEL2 against streptavidin-coated beads (Dynabeads™ MyOne™ Streptavidin C1) in HEPES buffer (no protein, negative control,* ***A****) and streptavidin-coated beads (Dynabeads™ MyOne™ Streptavidin C1) in PBS buffer (no protein, negative control,* ***B****). Dot color and size correspond to the normalized sequence counts.* ***A****: TCs = 1'871'375, AC = 0.66, Cut-off = 20;* ***B****: TCs = 1'914'065, AC = 0.67, Cut-off = 20.*

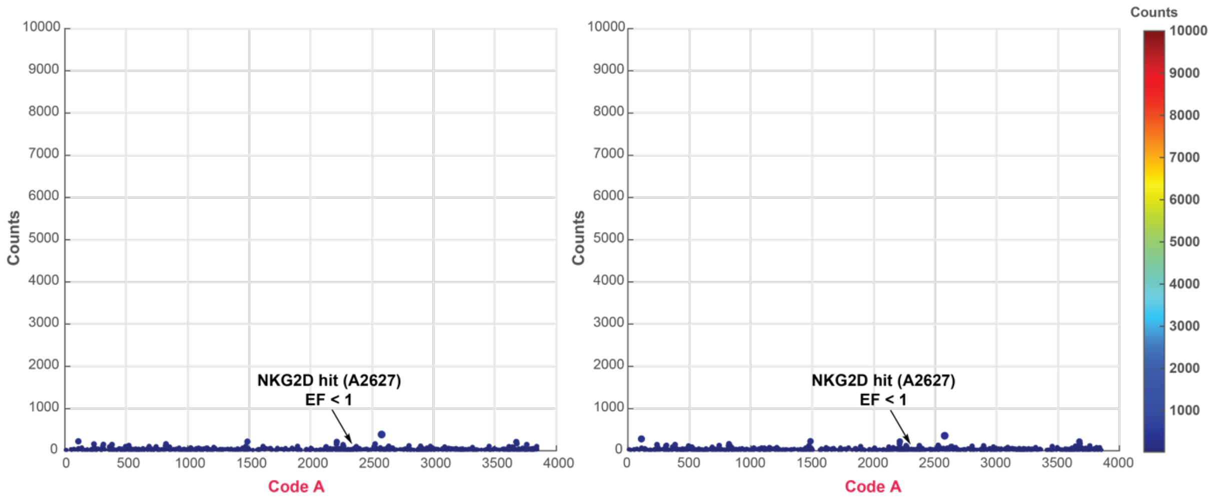

Figure S30. *Selection fingerprints after screening of FM-DEL1 against HSA (irrelevant protein, addition negative control for NKG2D-Fc screenings). HSA screenings have been performed in duplicate and are represented as 2D-plots (left, total counts TCs 53'417, average counts ACs 14, cut-off 20; right, TCs 54'806, ACs 14, cut-off 20). The most enriched NKG2D hit (A2627) identified from FM-DEL1 is indicated with an arrow together with the corresponding EFs in HSA selections.*

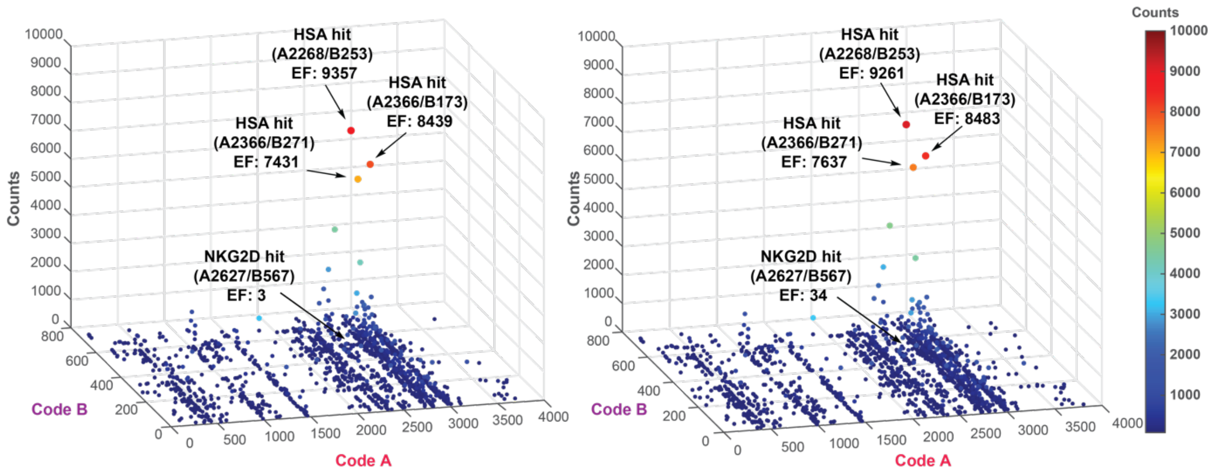

Figure S31. *Selection fingerprints after screening of FM-DEL2 against HSA (irrelevant protein, addition negative control for NKG2D-Fc screenings). HSA screenings have been performed in duplicate and are represented as 3D-plots (left, TCs 2'703'369, ACs 0.9, cut-off 40; right, TCs 2'812'181, ACs 1.0, cut-off 40). The most enriched HSA hits (A2268/B253, A2366/B173, A2366/B271) and NKG2D hit (A2627/B567) are indicated with arrows together with the corresponding EFs in HSA selections.*

6.2 Z-score analysis of DEL selections

In the following section, DEL selections reported in the main manuscript (**Figures 2A-D**, **3D-F**, **5A-D**, **6A-D**) are re-analyzed using Z-score normalization (**Figures S32-S38**) in accordance with methods and with the equation reported in the literature[14].

The top ten most enriched hits and five background library members (i.e., library members with low enrichments, randomly picked from the background), identified in the selection against the target of interest, are reported in tables with their corresponding Enrichment Factors (EFs) and Z-scores. The fifteen compounds are also evaluated against the respective anti-target (e.g., PSMA vs. GCP3), providing a comparative view for EF against Z-score values (see **Tables S1-S7**).

For ACP3, a target for which no hit was identified from FM-DEL1, only FM-DEL2 screenings have been re-analyzed with this approach (i.e., Z-score analysis).

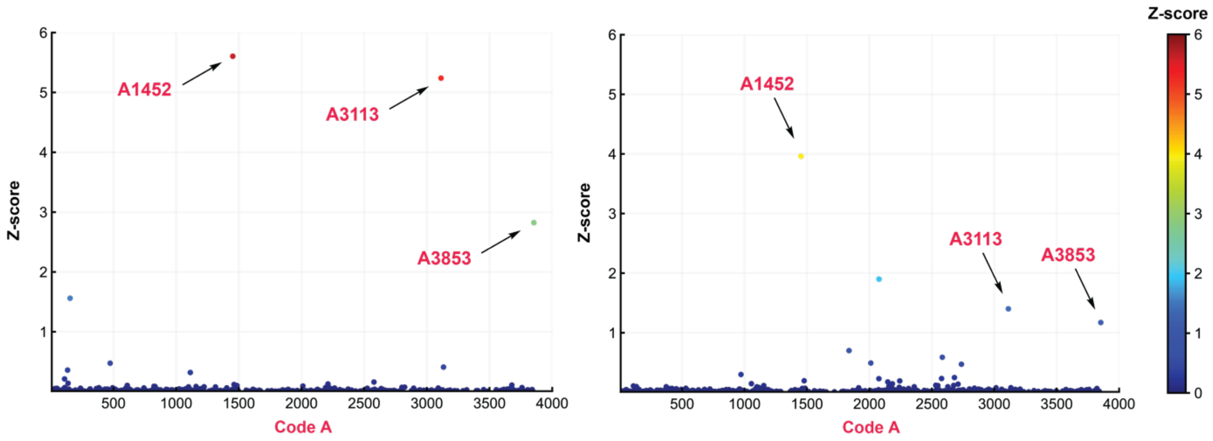

Figure S32. *FM-DEL1 screening results (HTS) are represented as 2D-plots after selections against PSMA (left) and against GCP3 (right). The x- and y-axes correspond to building blocks A and Z-score, respectively. The jet scale colour represents Z-score magnitude, with warmer colors denoting a higher enrichment. The most enriched PSMA hits identified from FM-DEL1 are indicated with arrows in both PSMA and GCP3 selections.*

Table S1

	PSMA		GCP3	
**Library member**	**EF**	**Z-score**	**EF**	**Z-score**
A1452 (PSMA hit)	358	5.6047	244	3.9625
A3113 (PSMA hit)	335	5.2388	87	1.4018
A3853	181	2.8239	73	1.1719
A154	98	1.5609	4	0.0517
A474	31	0.4753	1	0.0065
A3133	26	0.4101	6	0.0781
A134	23	0.3594	7	0.0969
A1113	21	0.3196	6	0.0831
A110	14	0.2121	8	0.1082

A2578	11	0.1602	15	0.2326
...				
A1932	0.07	-0.0149	0.39	-0.0098
A1054	0.07	-0.0149	0.86	-0.0023
A3383	0.07	-0.0149	0.39	-0.0098
A2966	0.07	-0.0149	0.00	-0.0161
A2971	0.07	-0.0149	0.70	-0.0048

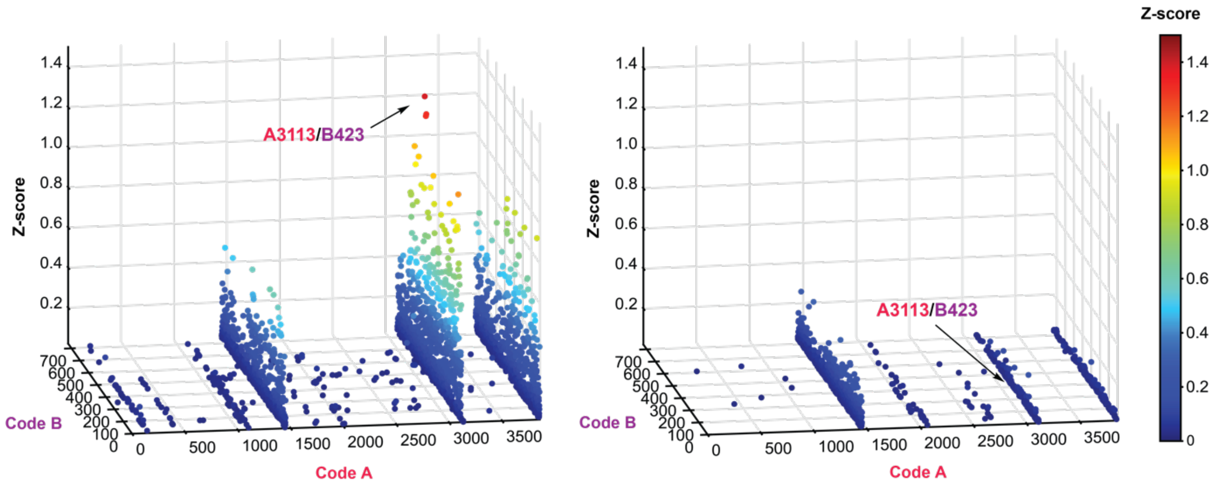

Figure S33. *FM-DEL2 screening results (HTS) are represented as 3D-plots after selections against PSMA (left) and against GCP3 (right). The x- and y-axes correspond to building blocks A and B, respectively while z-axis represents Z-score. The jet scale colour represents Z-score magnitude, with warmer colors denoting a higher enrichment. The most enriched PSMA hits identified from FM-DEL2 are indicated with arrows in both PSMA and GCP3 selections.*

Table S2

	**PSMA**		**GCP3**	
**Library member**	**EF**	**Z-score**	**EF**	**Z-score**
A3113/B423 (PSMA hit)	2342	1.3888	17	0.0098
A3113/B405	2214	1.3111	229	0.1349
A3113/B410	2192	1.2984	105	0.0616
A3113/B35	1918	1.1359	93	0.0547
A3113/B540	1806	1.0695	42	0.0243
A3113/B323	1779	1.0533	54	0.0312
A3113/B493	1767	1.0462	106	0.0623
A3113/B136	1736	1.0278	19	0.0105
A3113/B528	1666	0.9862	27	0.0153
A3113/B356	1646	0.9749	12	0.0063
…				
A3855/B739	1.19	0.0001	0.00	-0.0006
A1667/B272	1.19	0.0001	0.00	-0.0006
A1667/B273	1.19	0.0001	0.00	-0.0006
A1667/B285	1.19	0.0001	2.33	0.0008
A3016/B463	1.19	0.0001	0.00	-0.0006

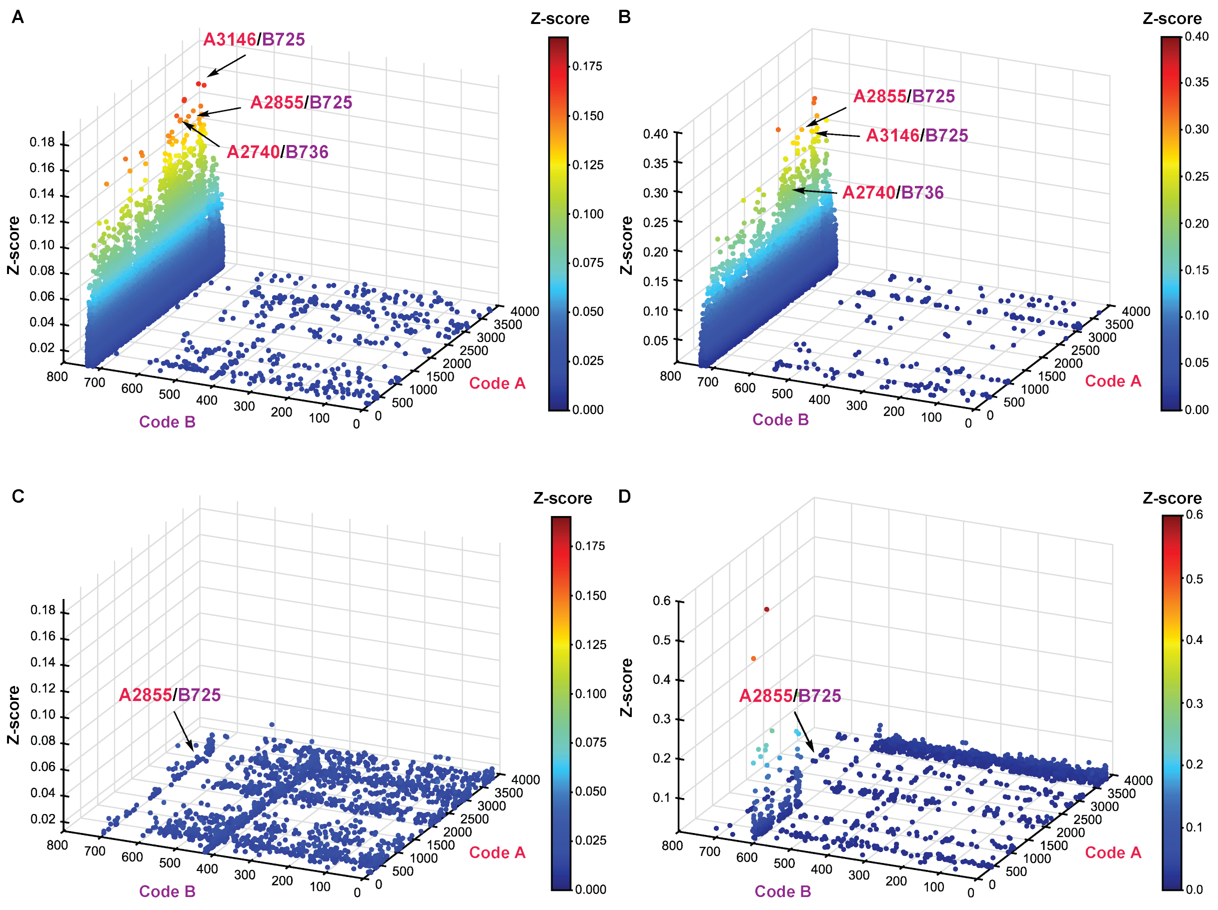

Figure S34. *FM-DEL2 screening results (HTS) are represented as 3D-plots after selections against hACP3 (A), mACP3 (B), H44A, H289A hACP3 (C) and TNAP (anti-target, D). The x- and y-axes correspond to building blocks A and B, respectively while z-axis represents Z-score. The jet scale colour represents Z-score magnitude, with warmer colors denoting a higher enrichment. In all selections, the most enriched hACP3 hits identified from FM-DEL2 are indicated with arrows.*

Table S3

	hACP3		mACP3		H44A, H289A hACP3		TNAP	
**Library member**	**EF**	**Z-score**	**EF**	**Z-score**	**EF**	**Z-score**	**EF**	**Z-score**
A3146/B725 (hACP3 hit)	284	0.1671	458	0.2715	9	0.0049	0	-0.0006
A3312/B725	275	0.1626	457	0.2699	3	0.0015	0	-0.0006
A2723/B725	275	0.1626	455	0.2691	21	0.0117	4	0.0018
A2855/B736	272	0.1604	461	0.2723	1	0.0001	3	0.0010
A2507/B725	263	0.1551	370	0.2185	17	0.0097	0	-0.0006
A1122/B736	253	0.1491	293	0.1728	7	0.0035	1	0.0002
A3213/B725	251	0.1484	535	0.3165	18	0.0104	3	0.0010
A1465/B725	251	0.1476	207	0.1222	3	0.0015	4	0.0018
A2855/B725 (hACP3 hit)	251	0.1476	484	0.2868	11	0.0063	5	0.0026
A2740/B736 (hACP3 hit)	249	0.1469	261	0.1535	2	0.0008	0	-0.0006

...								
A1/B1	1.26	0.0002	0.00	-0.0006	0.00	-0.0006	4.05	0.0018
A2238/B206	1.26	0.0002	4.06	0.0018	4.62	0.0021	5.40	0.0026
A2238/B203	1.26	0.0002	4.06	0.0018	18.48	0.0104	4.05	0.0018
A2238/B190	1.26	0.0002	2.71	0.0010	0.00	-0.0006	8.10	0.0042
A2238/B186	1.26	0.0002	2.71	0.0010	1.15	0.0001	4.05	0.0018

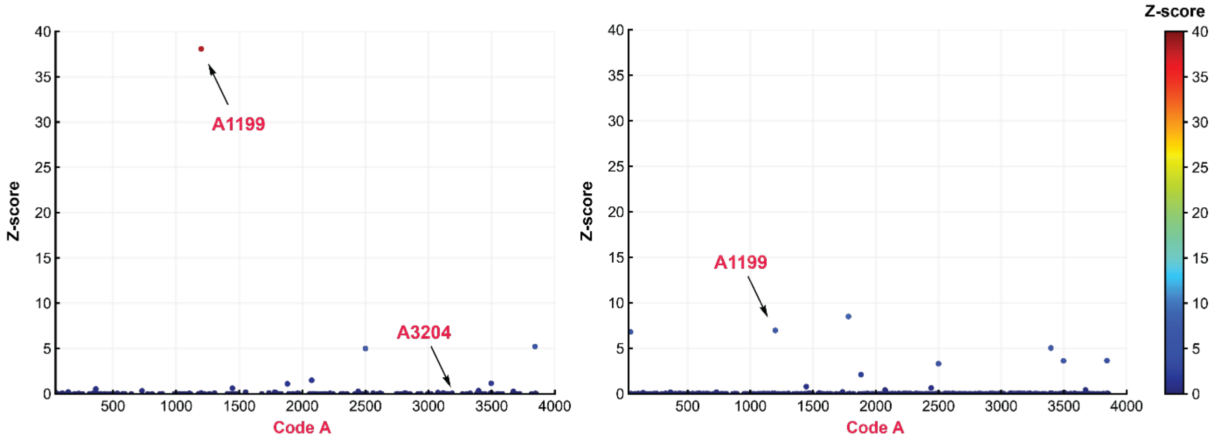

Figure S35. *FM-DEL1 screening results (HTS) are represented as 2D-plots after selections against CAIX (left) and against CAII (right). The x- and y-axes correspond to building blocks A and Z-score, respectively. The jet scale colour represents Z-score magnitude, with warmer colors denoting a higher enrichment. The most enriched CAIX hits identified from FM-DEL1 are indicated with arrows in both CAIX and CAII selections.*

Table S4

	CAIX		CAII	
**Library member**	**EF**	**Z-score**	**EF**	**Z-score**
A1199 (CAIX hit)	2358	38.0797	429	6.9601
A3843	324	5.2038	227	3.6388
A2500	310	4.9838	206	3.3052
A2074	93	1.4893	26	0.4090
A3496	73	1.1588	226	3.6205
A1882	69	1.0942	132	2.1113
A1446	39	0.6094	50	0.7832
A364	34	0.5281	11	0.1690
A3396	22	0.3394	313	5.0259
A730	21	0.3154	12	0.1791
…				
A1945	0.06	-0.0151	0.00	-0.0161
A1750	0.06	-0.0151	0.13	-0.0141
A1747	0.06	-0.0151	0.13	-0.0141
A1743	0.06	-0.0151	0.13	-0.0141
A1739	0.06	-0.0151	0.00	-0.0161

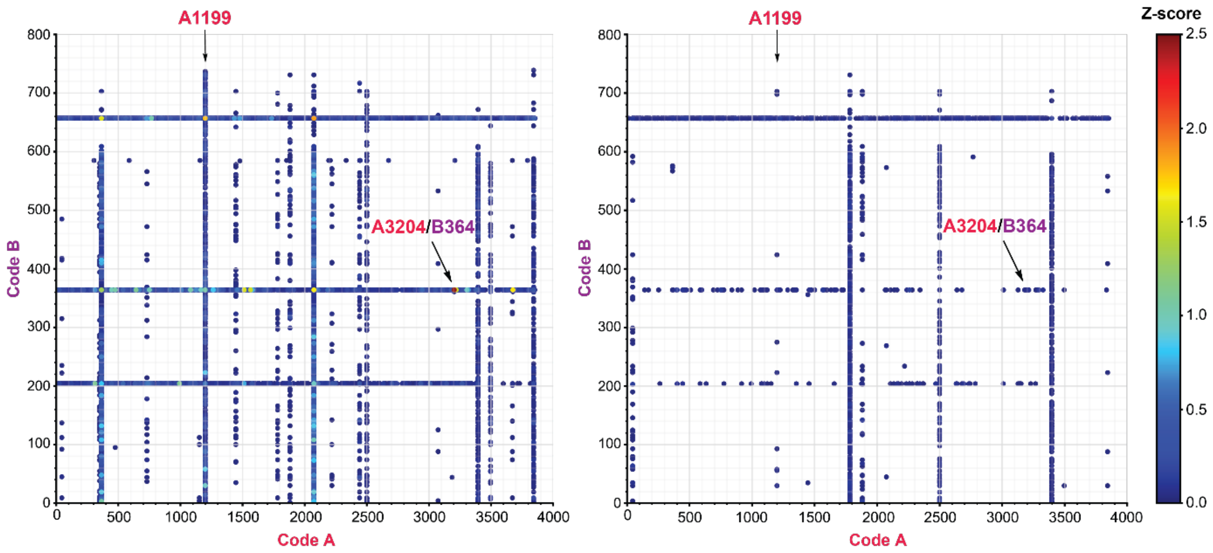

Figure S36. *FM-DEL2 screening results (HTS) are represented as 3D-plots after selections against CAIX (left) and against CAII (right). The x- and y-axes correspond to building blocks A and B, respectively while z-axis represents Z-score. The jet scale colour represents Z-score magnitude, with warmer colors denoting a higher enrichment. The most enriched CAIX hits identified from FM-DEL2 are indicated with arrows in both CAIX and CAII selections.*

Table S5

	CAIX		CAII	
**Library member**	**EF**	**Z-score**	**EF**	**Z-score**
A3204/B364 (CAIX hit)	4157	2.4514	7	0.0035
A2074/B657	3113	1.8437	130	0.0764
A1199/B657	2984	1.7675	124	0.0730
A3676/B364	2812	1.6654	3	0.0015
A2074/B364	2775	1.6435	65	0.0379
A364/B657	2644	1.5660	120	0.0702
A1513/B364	2623	1.5534	15	0.0083
A3220/B364	2580	1.5282	13	0.0070
A1564/B364	2529	1.4977	9	0.0049
A364/B364	2212	1.3102	53	0.0310
…				
A1/B2	1.12	0.0001	0.00	-0.0006
A2285/B233	1.12	0.0001	0.00	-0.0006
A2285/B237	1.12	0.0001	1.16	0.0001
A2285/B255	1.12	0.0001	2.32	0.0008
A2285/B262	1.12	0.0001	1.16	0.0001

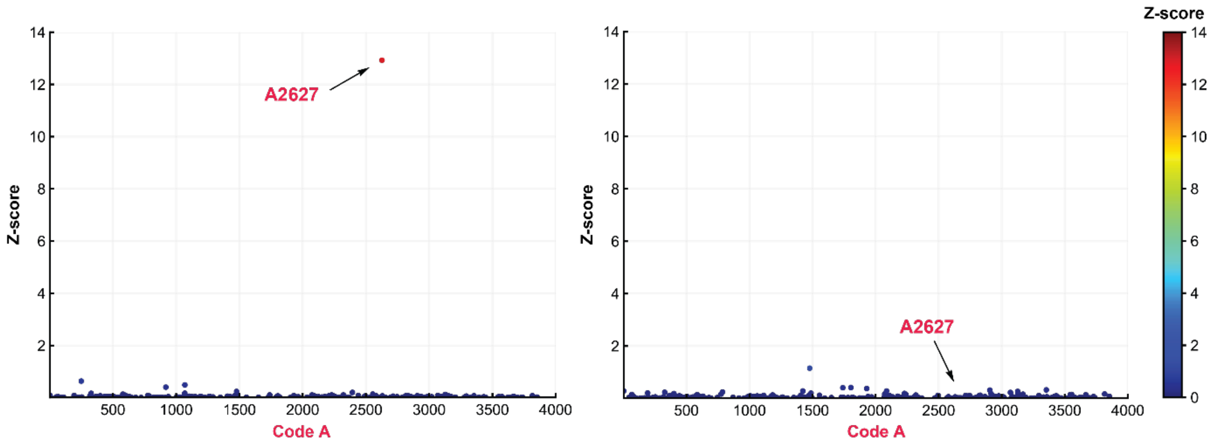

Figure S37. *FM-DEL1 screening results (HTS) are represented as 2D-plots after selections against NKG2D-Fc (left) and against hIgG-Fc (right). The x- and y-axes correspond to building blocks A and Z-score, respectively. The jet scale colour represents Z-score magnitude, with warmer colors denoting a higher enrichment. The most enriched NKG2D hits identified from FM-DEL1 are indicated with arrows in both NKG2D-Fc and hIgG-Fc selections.*

Table S6

	NKG2D-Fc		hIgG-Fc	
**Library member**	**EF**	**Z-score**	**EF**	**Z-score**
A2627 (NKG2D hit)	823	12.9279	0.66	-0.0055
A250	40	0.6352	3	0.0329
A1069	31	0.4884	1	0.0024
A919	26	0.4032	1	0.0077
A1478	16	0.2394	73	1.1524
A2393	14	0.2027	0.74	-0.0042
A1068	12	0.1726	0.25	-0.0121
A328	11	0.1673	15	0.2316
A2555	10	0.1385	1	0.0011
A579	9	0.1333	8	0.1203
...				
A2583	0.08	-0.0148	0.00	-0.0161
A159	0.08	-0.0148	0.00	-0.0161
A1253	0.08	-0.0148	0.33	-0.0108
A1758	0.08	-0.0148	0.25	-0.0121
A951	0.08	-0.0148	0.25	-0.0121

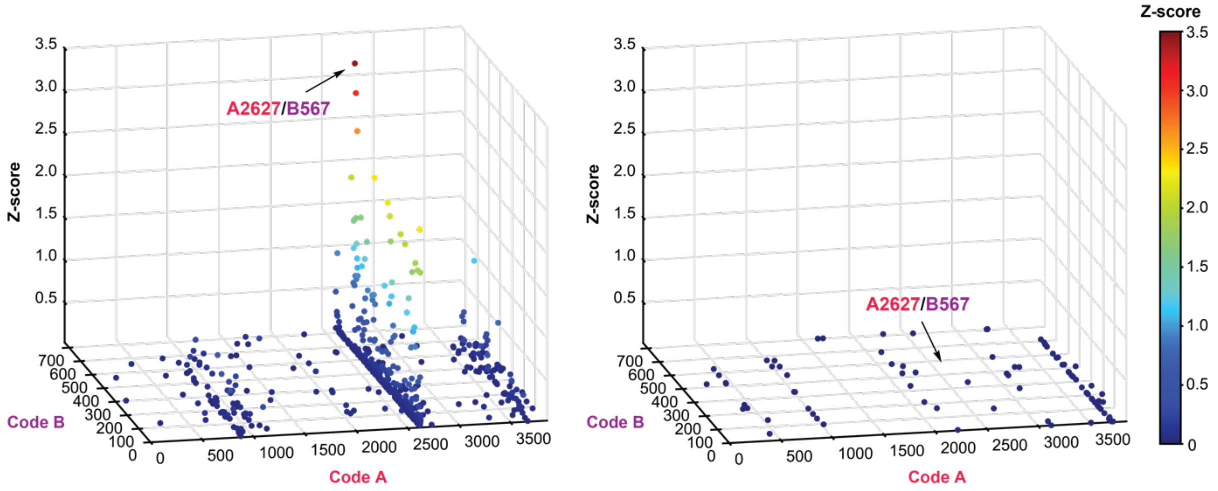

Figure S38. *FM-DEL2 screening results (HTS) are represented as 3D-plots after selections against NKG2D-Fc (left) and against hIgG-Fc (right). The x- and y-axes correspond to building blocks A and B, respectively while z-axis represents Z-score. The jet scale colour represents Z-score magnitude, with warmer colors denoting a higher enrichment. The most enriched NKG2D hits identified from FM-DEL2 are indicated with arrows in both NKG2D-Fc and hIgG-Fc selections.*

Table S7

	NKG2D		hIgG-Fc	
**Library member**	**EF**	**Z-score**	**EF**	**Z-score**
A2627/B567 (NKG2D hit)	5801	3.4220	2	0.0008
A2627/B558	5210	3.0861	7	0.0037
A2627/B546	4485	2.6564	1	0.0001
A2627/B9	3967	2.3496	5	0.0022
A2627/B398	3954	2.3420	1	0.0001
A2627/B284	3763	2.2289	4	0.0015
A2627/B266	3543	2.0984	5	0.0022
A2627/B173	3433	2.0331	10	0.0051
A2627/B597	3426	2.0290	0	-0.0006
A2627/B135	3340	1.9783	1	0.0001
…				
A1984/B200	1.17	0.0001	0.00	-0.0006
A2951/B247	1.17	0.0001	1.20	0.0001
A2951/B246	1.17	0.0001	4.79	0.0022
A2951/B243	1.17	0.0001	2.39	0.0008
A2951/B239	1.17	0.0001	1.20	0.0001

6.3 Selectivity of hits A2627 and A2627/B567 for NKG2D in DEL screenings

Enrichment factors and Z-score values of NKG2D hits for all selections reported in this study are summarized in the table below:

Table S8

NKG2D Hits	Target protein	Replicate	EF	Z-score
**Hit A2627 (FM-DEL1)**	NKG2D	1	823	12.9279
		2	626	10.0658
	hIgG-Fc	1	<1	-0.0055
		2	<1	-0.0022
	HSA	1	<1	-0.0091
		2	<1	-0.0059
	PSMA	1	<1	-0.0101
		2	<1	-0.0060
	GCP3	1	2	0.0178
		2	2	0.0092
	hACP3	1	<1	-0.0045
		2	2	0.0143
	mACP3	1	1	0.0065
		2	2	0.0133
	H44A, H289A hACP3	1	3	0.0252
		2	2	0.0118
	TNAP	1	2	0.0123
		2	2	0.0093
	CAIX	1	<1	-0.0140
		2	<1	-0.0131
	CAII	1	<1	-0.0039
		2	<1	-0.0057
**Hit A2627/B567 (FM-DEL2)**	NKG2D	1	5801	3.4220
		2	4670	2.7700
	hIgG-Fc	1	2	0.0008
		2	3	0.0009
	HSA	1	3	0.0013
		2	34	0.0198
	PSMA	1	<1	-0.0006
		2	3	0.0010
	GCP3	1	17	0.0098
		2	16	0.0092
	hACP3	1	<1	-0.0006
		2	<1	-0.0006
	mACP3	1	<1	-0.0006
		2	<1	-0.0006
		1	2	0.0008

	H44A, H289A hACP3	2	1	0.0001
	TNAP	1	1	0.0002
		2	<1	-0.0006
	CAIX	1	3	0.0014
		2	<1	-0.0006
	CAII	1	1	0.0001
		2	<1	-0.0006

6.4 Interferon-gamma release assay on human NK-92 cells

IFN-γ release assay was performed using human NK-92 cells incubated with compounds **26** and **30**. The compounds did not induce IFN-γ release, confirming that they act solely as binders of the cognate target (NKG2D). The plotted results of the experiment are shown in **Figure S39**.

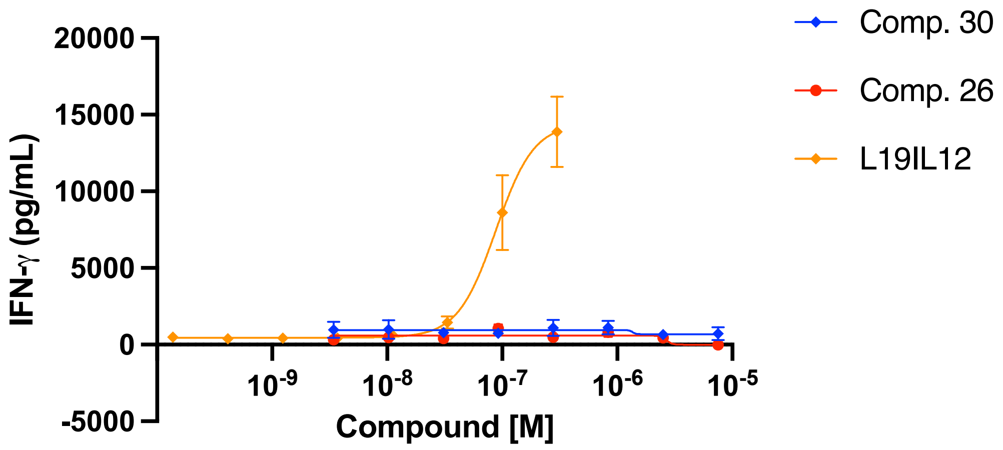

Figure S39. *Human NK-92 cells were stimulated in vitro in the presence of a serial dilution (1:3) of compounds* ***26*** *and* ***30*** *(initial concentration 7.5 µM). After 24 hours of incubation, IFN-γ levels in the culture supernatant were measured by ELISA.* An Interleukin-12-antibody fusion protein (L19IL12)*, known for its potent immunostimulatory activity*[15,16]*, was used as positive control (serial dilution starting from 30 ng/mL).*

6.5 Additional co-elution experiments based on size exclusion chromatography

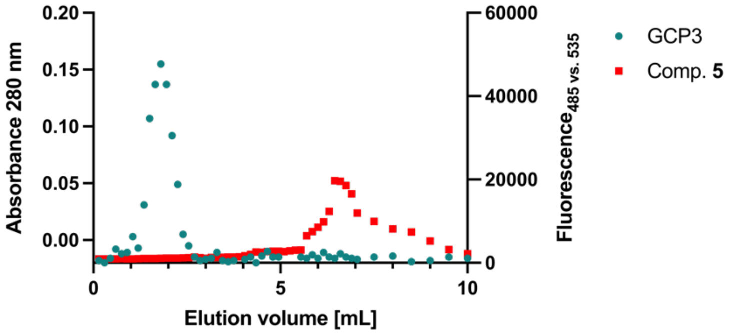

Figure S40. *Co-elution experiment based on size-exclusion chromatography (PD-10) with compound **5** (red curve, fluorescence 485 nm vs. 535 nm) pre-incubated with GCP3 (green curve, absorbance at 280 nm).*

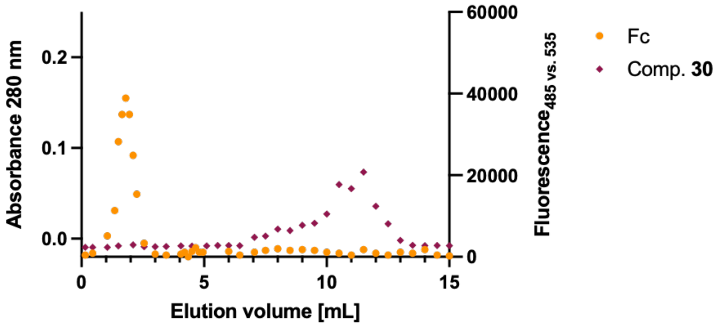

Figure S41. *Co-elution experiment based on size-exclusion chromatography (PD-10) with compound **30** (violet curve, fluorescence 485 nm vs. 535 nm) pre-incubated with Fc (orange curve, absorbance at 280 nm).*

6.6 ELISA plates (pictures)

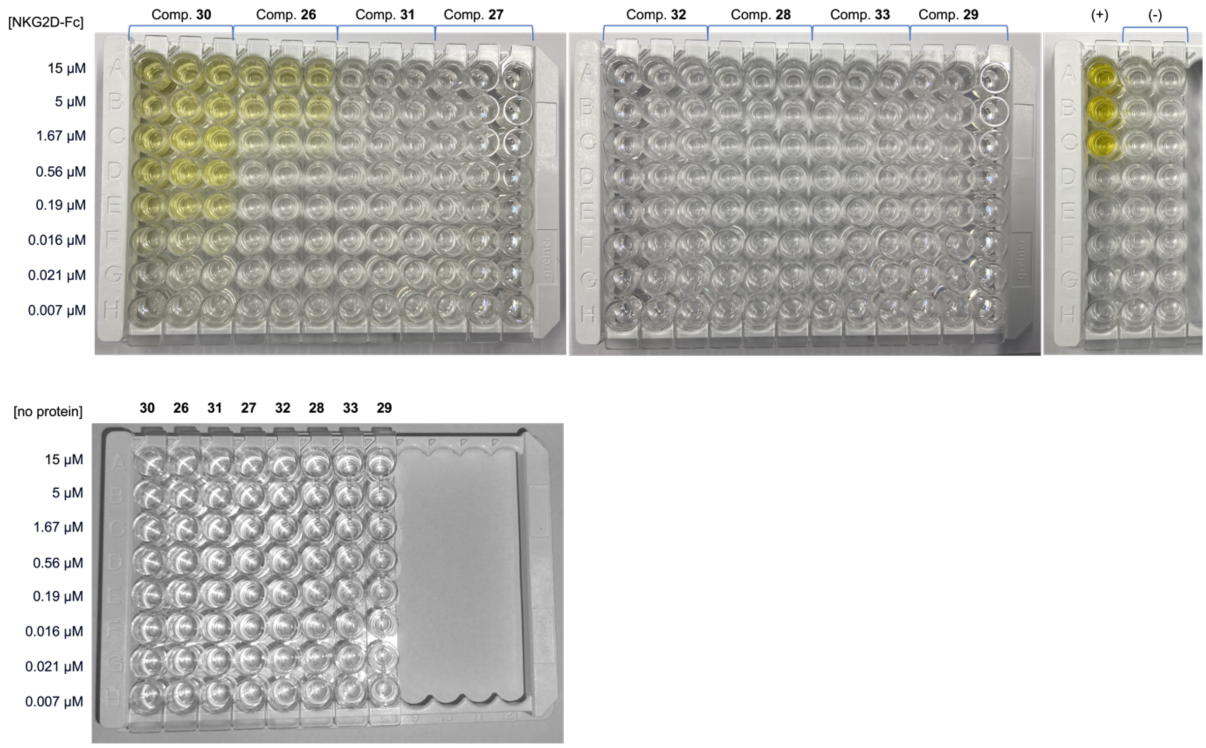

Figure S42. *Pictures of ELISA plates in which binding of compounds **27**-**33** was tested against NKG2D-Fc. NKG2D-Fc was incubated with Protein A [positive controls, (+)] or with Lys-FITC [negative control on the right, (-)]. Alternatively, fluorescence compounds were omitted [negative controls on the left, (-)]. Compounds **27**-**33** were incubated at the same concentration (serial dilution from 15 μM to 0.007 μM) in the respective wells without NKG2D-Fc (no protein control).*

6.7 Flow cytometry assay - additional data

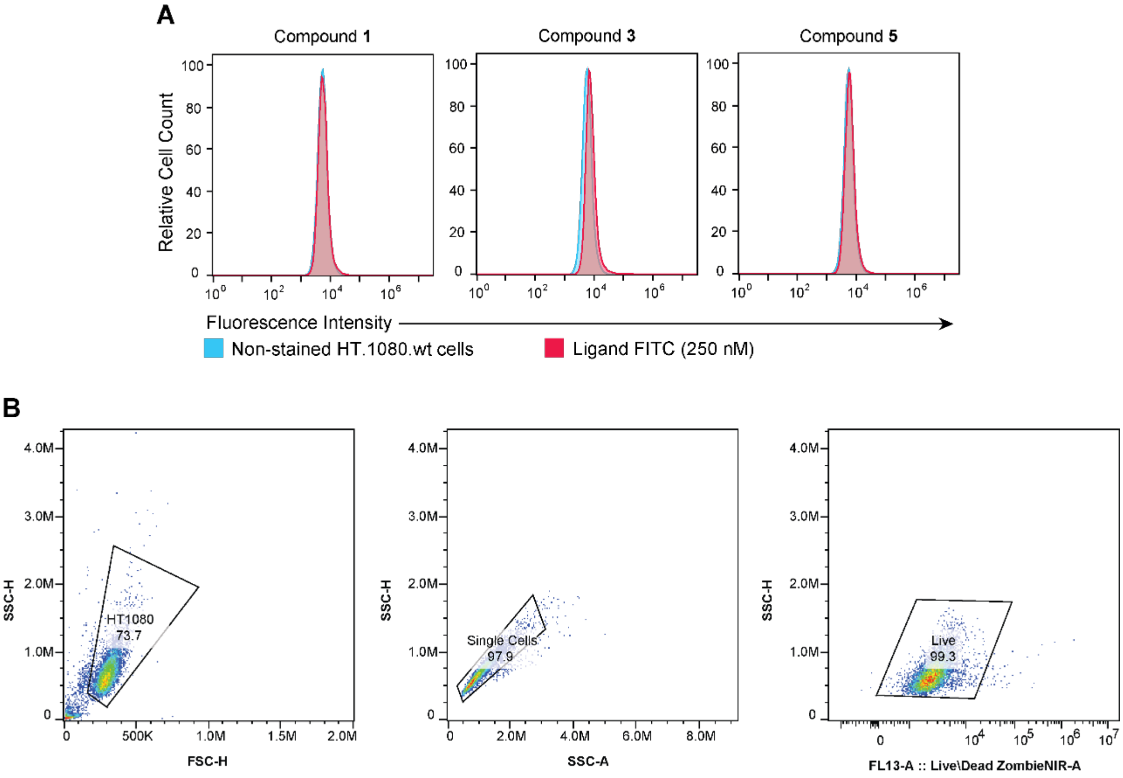

Figure S43. *(**A**) Flow cytometry analysis of HT.1080.wt tumor cells incubated with compounds **1**, **3**, and **5** in comparison with non-stained cells; (**B**) Flow cytometry gating strategy applied for the experiment.*

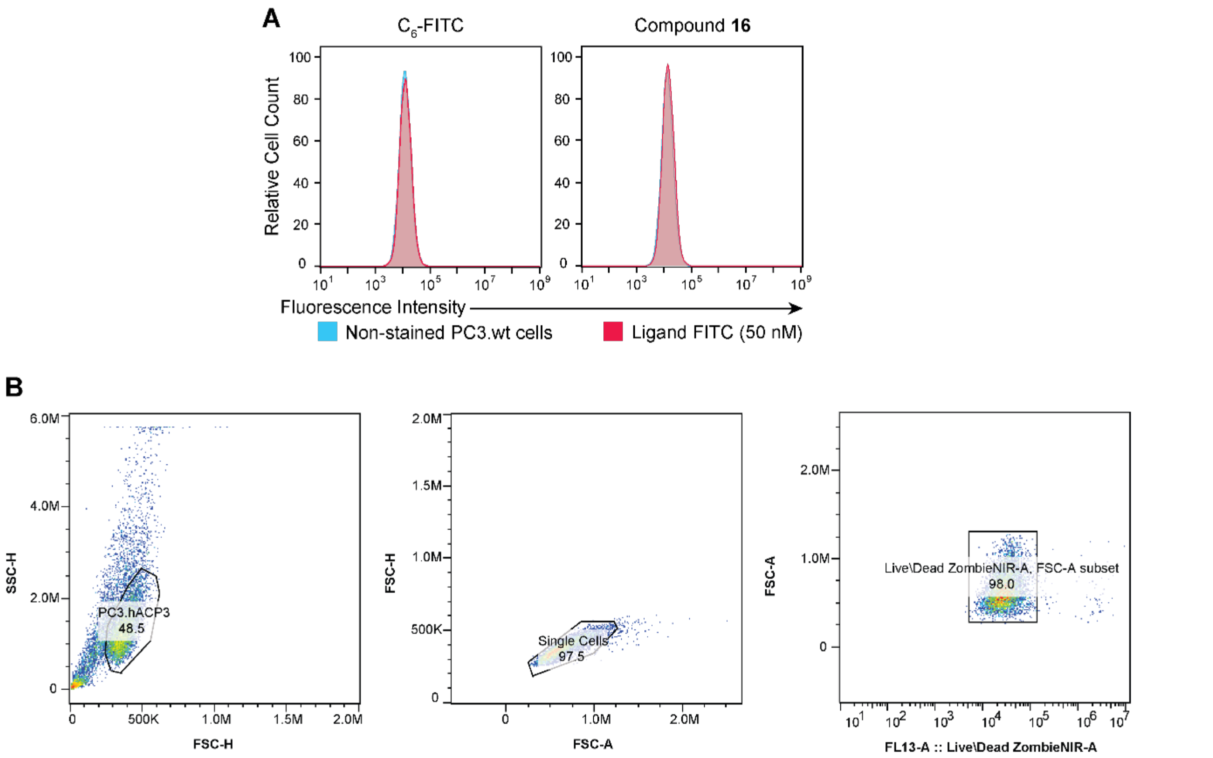

Figure S44. *(**A**) Flow cytometry analysis of PC3.wt tumor cells incubated with compounds C_6-FITC and **16**, in comparison with non-stained cells; (**B**) Flow cytometry gating strategy applied for the experiment.*

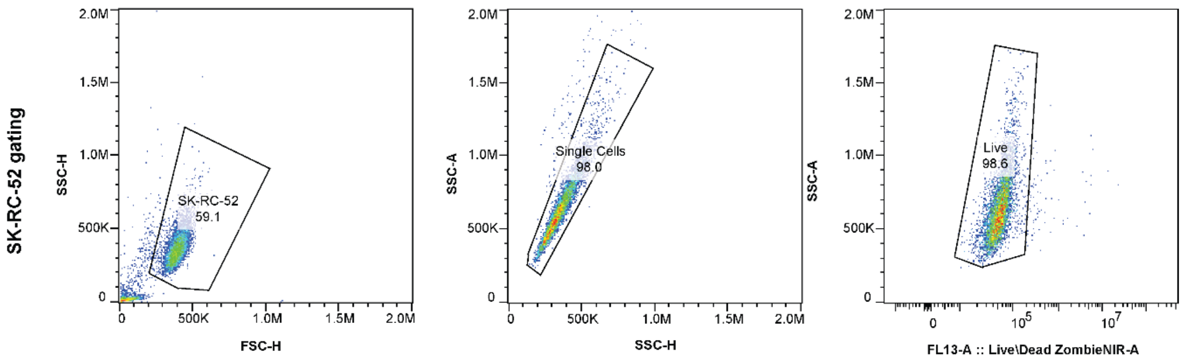

Figure S45. *Flow cytometry gating strategy applied for the flow cytometry experiment reported in **Figure 5J**.*

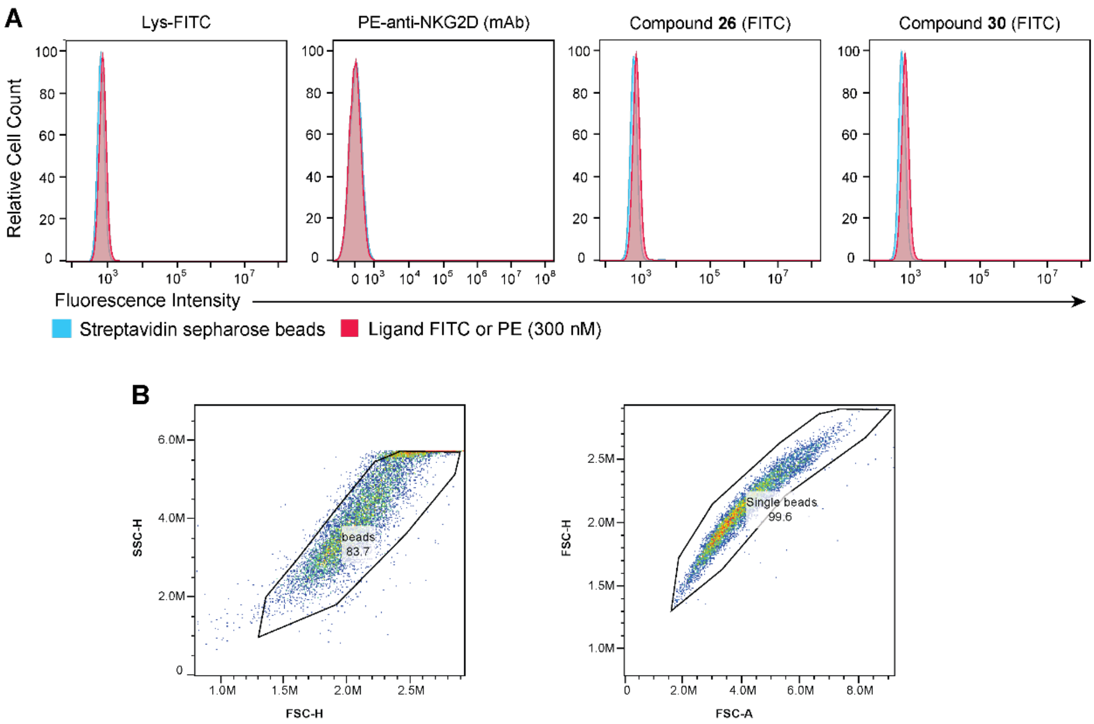

Figure S46. *(**A**) Flow cytometry analysis of streptavidin sepharose beads coated with biotin with compounds Lys-FITC, PE-anti-NKG2D (mAb), **26** and **30** in comparison with non-stained beads; (**B**) Flow cytometry gating strategy applied for the experiment.*

6.8 Molecular docking of PSMA and CAIX hit compounds

Molecular docking simulations were carried out by considering the resolved structure of PSMA in complex with the inhibitor PSMA-617 (PDB id: 8BOW)[4] and CAIX in complex with the inhibitor acetazolamide (PDB id: 3IAI)[3].

In docking simulations, truncated versions of the final compounds (see structures below) were employed, wherein the fluoresceine or DOTAGA moiety was replaced with an acetyl group. This simplification was adopted to reduce conformational complexity, as these distal groups are not expected to contribute to target binding.

PSMA

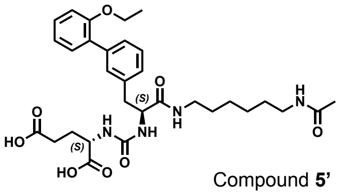

Compound **5'**

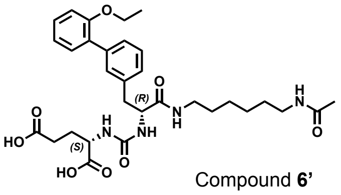

Compound **6'**

CAIX

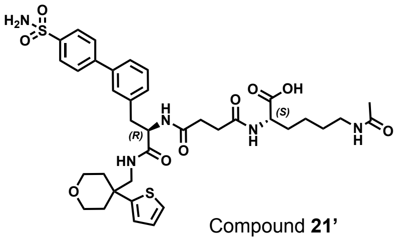

Compound **21'**

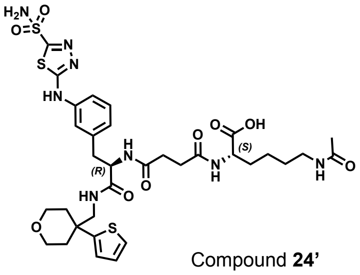

Compound **24'**

Figure S47. *Chemical structures of docked compounds.*

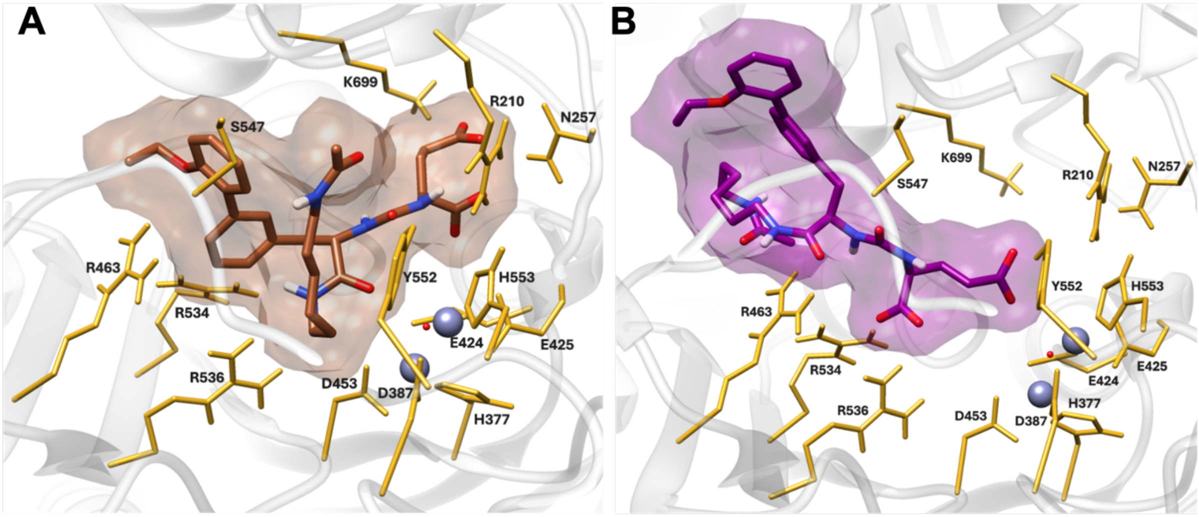

Figure S48. A*) Putative docking complex of PSMA with compound* ***5'*** *(S-configuration; brown sticks). Zinc ions are shown as dark purple spheres and the water molecule as a red sphere. Interacting PSMA*

residues are highlighted as golden sticks; the ligand surface is rendered in brown. The docking pose shows interactions of urea derivatives with PSMA, such as the carboxylic moieties that stabilize ion-pairs with Arg210 and Lys699, while the ureido group interacts with Tyr552, His553, and the zinc ion. ***B**) Putative docking complex of PSMA with compound **6'** (R-configuration; purple sticks). Zinc ions and the water molecule are shown as in panel A. Interacting PSMA residues are depicted as golden sticks; the ligand surface is shown in purple. In this configuration, the glutamate moiety of building block **A1452** forms ion pairs with the arginine patch (Arg463, Arg534, Arg536), which appears to hinder proper accommodation of the ureido group in the active site.*

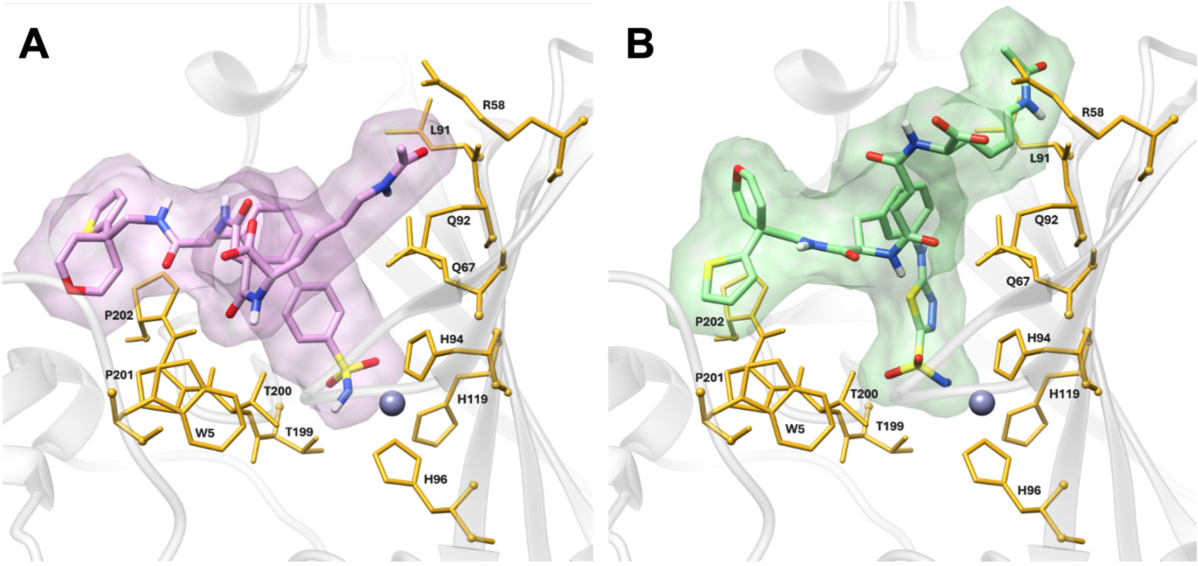

***Figure S49.** A) Predicted docking pose of CAIX in complex with compound **21'** (light pink sticks). The catalytic zinc ion is shown as a dark purple sphere; interacting CAIX residues are depicted as golden sticks, and the ligand surface is rendered in light pink. **B**) Predicted docking pose of CAIX with compound **24'** (light green sticks). Visualization conventions are as in panel A. Both compounds exhibit a coordination between the sulfonamide group and the zinc ion, further stabilized by additional hydrogen bonds with Thr200. The building block **A3204**, which extends toward the protein surface, engages in hydrophobic contacts with non-catalytic residues, such as Pro201, Pro202, and Trp5, likely contributing to the increased binding affinity through peripheral stabilization.*

7 HRMS and ^{1}H NMR spectra of compounds

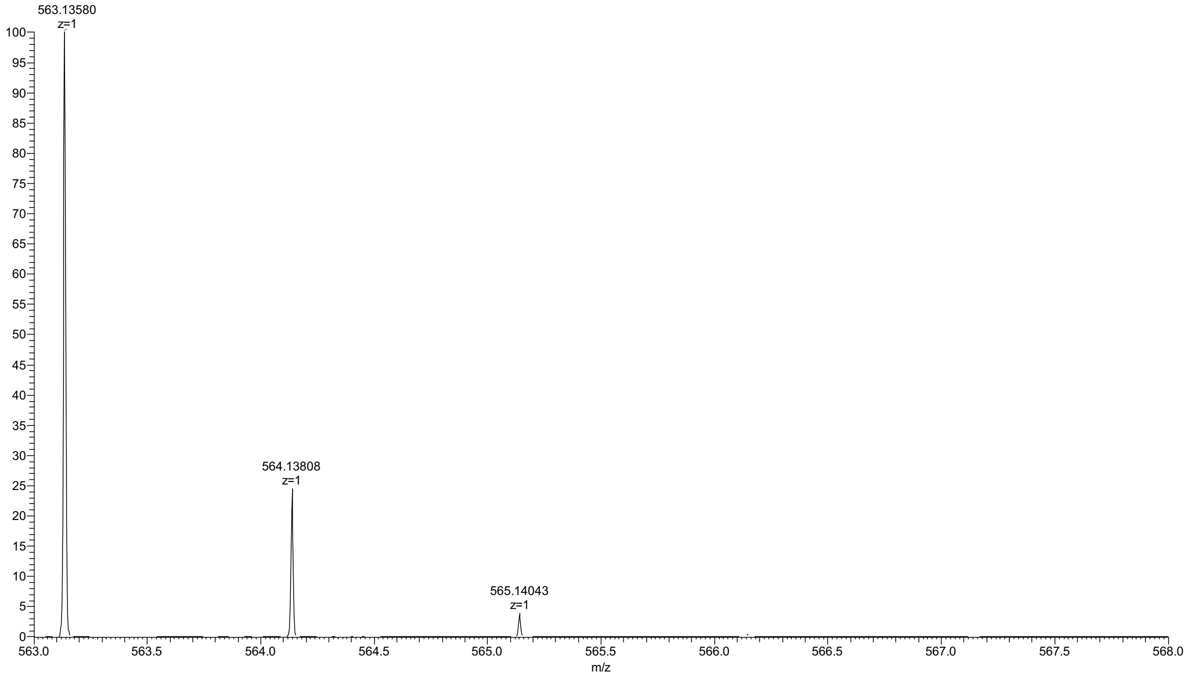

HRMS (ESI, m/z) of compound **s1a.**

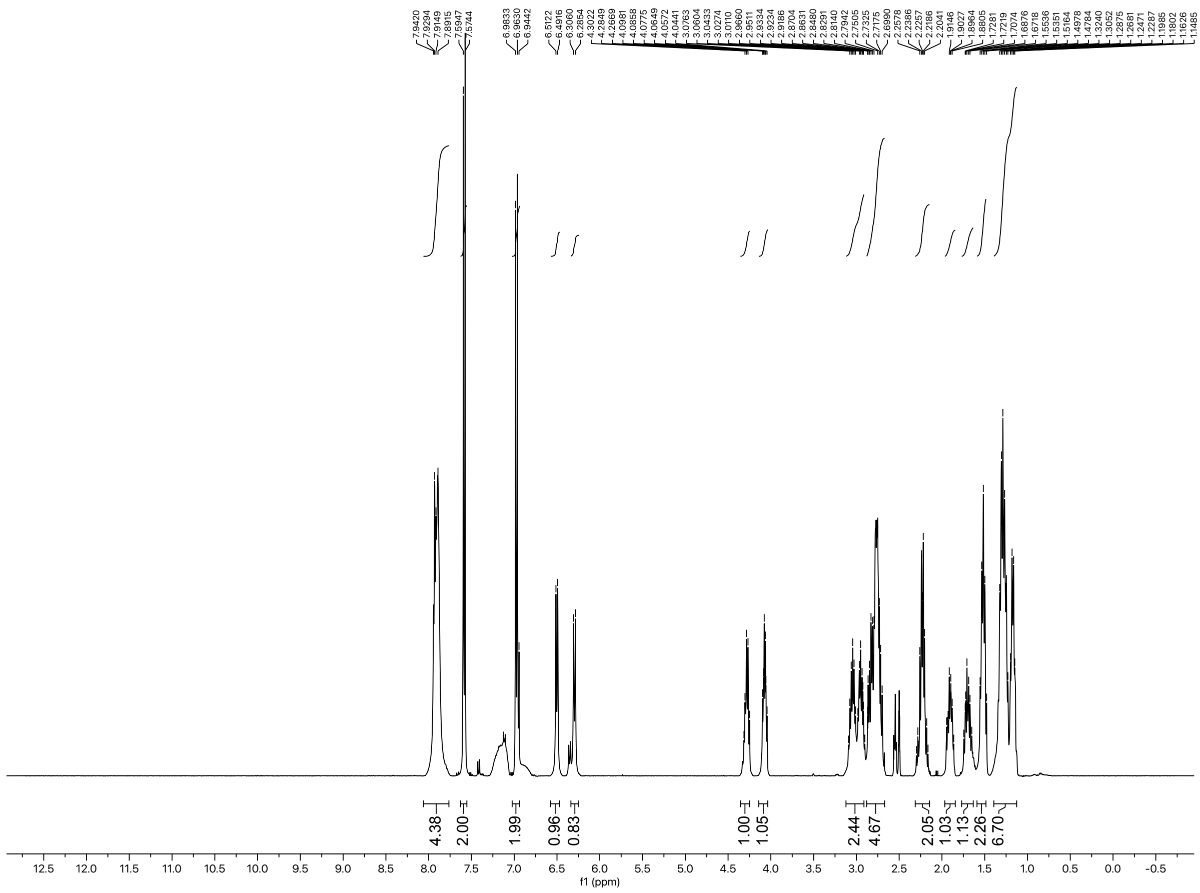

^{1}H NMR (400 MHz, DMSO-d_6) spectrum of compound **s1a**.

^{1}**H NMR** (400 MHz, DMSO-d_6): δ 7.98 - 7.88 (m, 4H), 7.58 (d, *J* = 8.1 Hz, 2H), 6.97 (d, *J* = 8.1 Hz, 2H), 6.50 (d, *J* = 8.2 Hz, 1H), 6.30 (d, *J* = 8.2 Hz, 1H), 4.33 - 4.23 (m, 1H), 4.11 - 4.02 (m, 1H), 3.11 - 2.90 (m, 2H), 2.88 - 2.68 (m, 5H), 2.30 - 2.14 (m, 2H), 1.95 - 1.84 (m, 1H), 1.76 - 1.60 (m, 1H), 1.52 (p, *J* = 7.5 Hz, 2H), 1.38 - 1.21 (m, 5H), 1.20 - 1.18 (m, 2H).

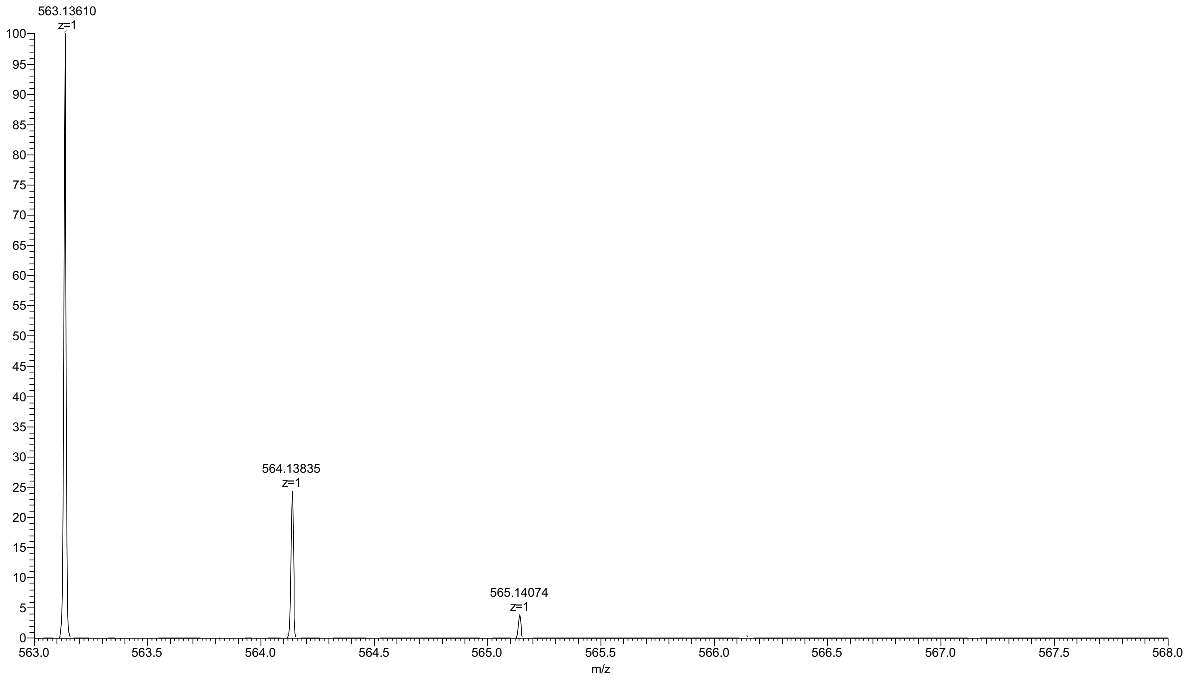

HRMS (ESI, m/z) of compound **s1b.**

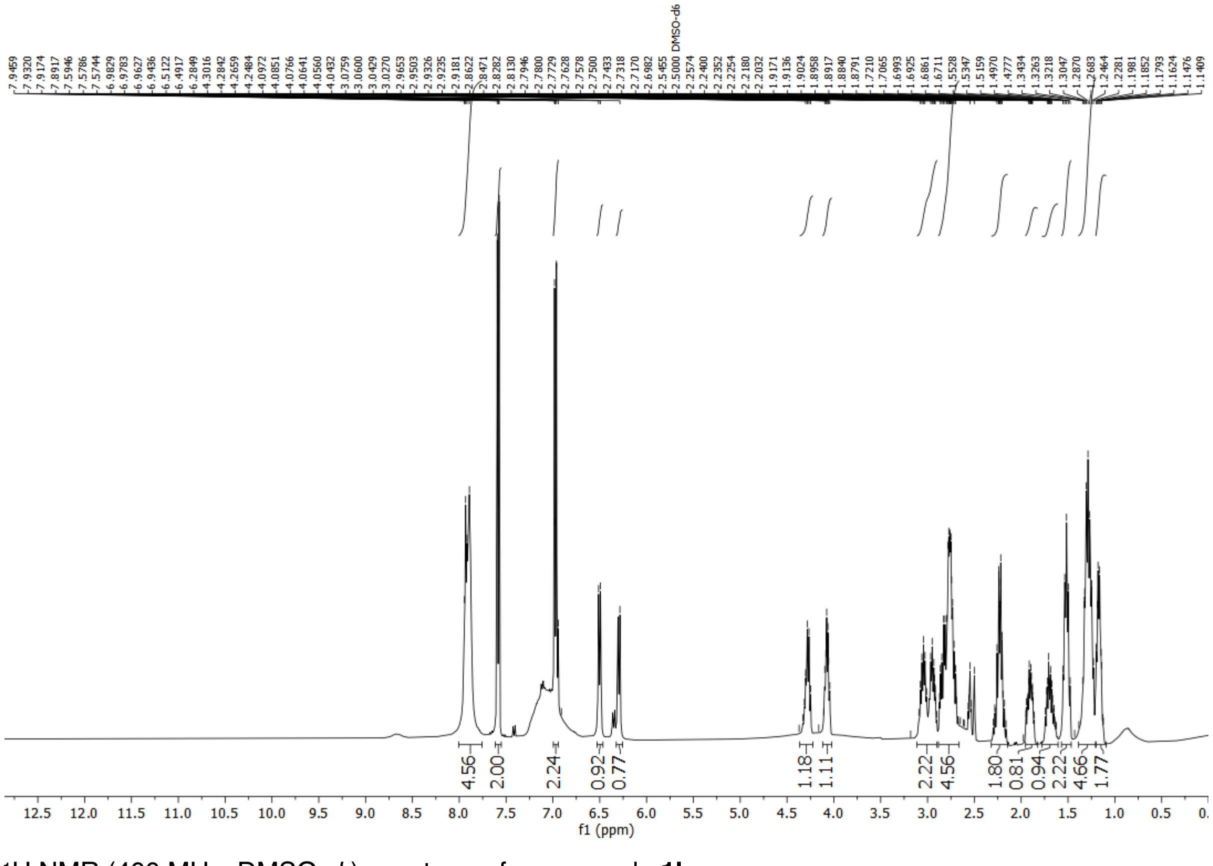

^{1}H NMR (400 MHz, DMSO-d_6) spectrum of compound **s1b**.

^{1}H NMR (400 MHz, DMSO-d_6) δ 7.96 - 7.84 (m, 4H), 7.55 (d, J = 8.1 Hz, 2H), 6.97 (d, J = 8.1 Hz, 2H), 6.49 (d, J = 8.4 Hz, 1H), 6.28 (d, J = 8.1 Hz, 1H), 4.33 - 4.23 (m, 1H), 4.11 - 4.02 (m, 1H), 3.11 - 2.90 (m, 2H), 2.88 - 2.68 (m, 5H), 2.30 - 2.14 (m, 2H), 1.95 - 1.84 (m, 1H), 1.76 - 1.60 (m, 1H), 1.54 (p, J = 7.6 Hz, 2H), 1.38 - 1.21 (m, 5H), 1.20 - 1.18 (m, 2H).

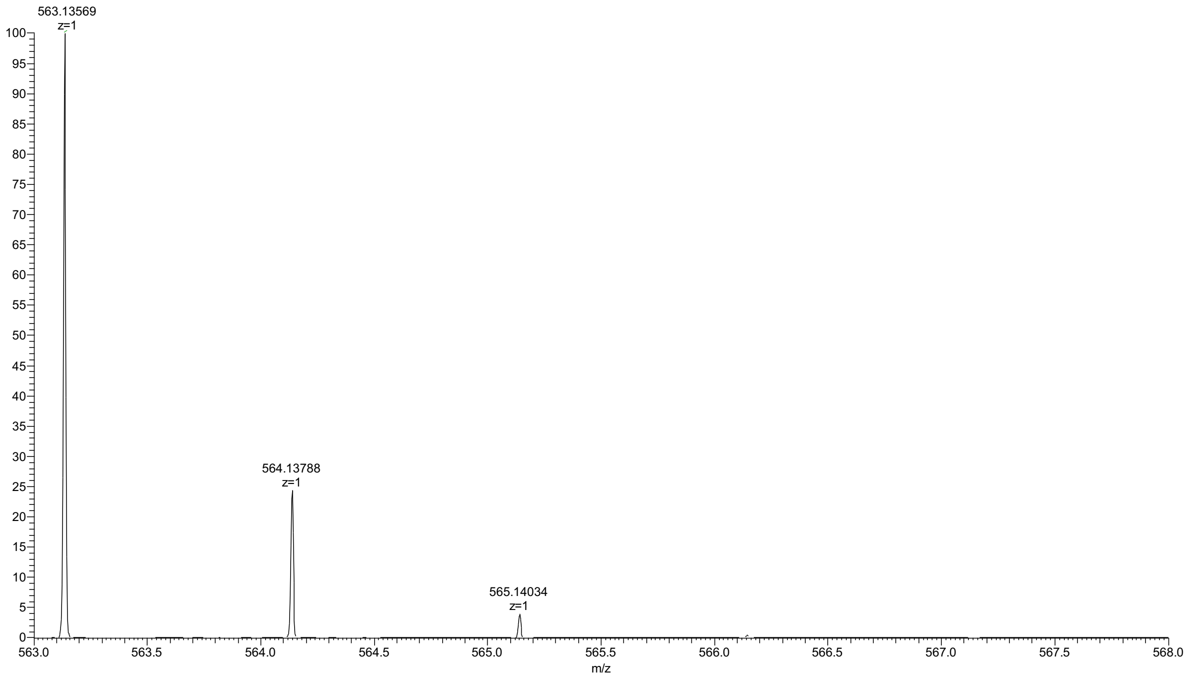

HRMS (ESI, m/z) of compound **s2a**.

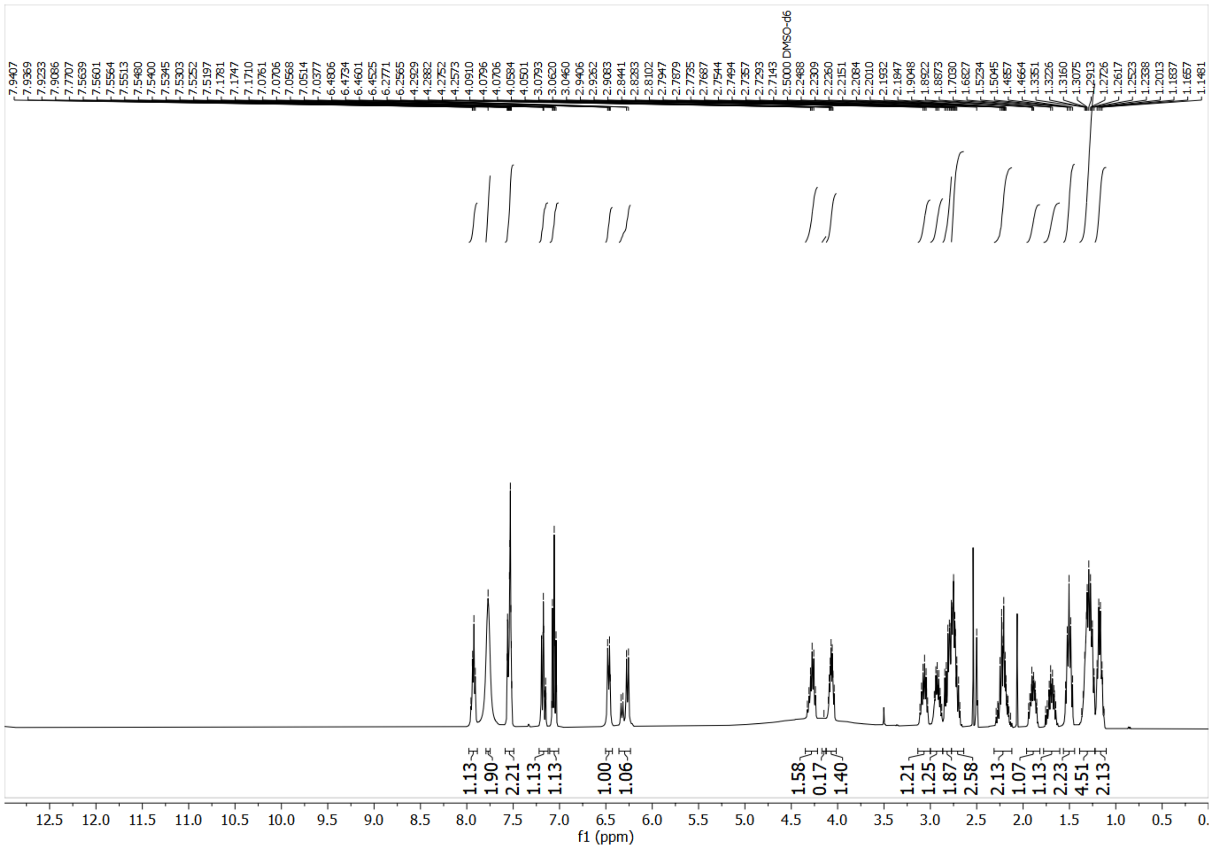

^{1}H NMR (400 MHz, DMSO-d_6) spectrum of compound **s2a**.

^{1}H NMR (400 MHz, DMSO-d_6): δ 7.93 (t, J = 5.7 Hz 1H), 7.77 (bs, 3H), 7.69 - 7.50 (m, 2H), 7.17 (tt, J = 7.7, 1.3 Hz, 1H), 7.07 (td, J = 7.8, 2.3 Hz, 1H), 6.47 (dd, J = 8.3, 2.9 Hz, 1H), 6.26 (d, J = 8.3 Hz, 1H), 4.35 - 4.21 (m, 1H), 4.12 - 4.01 (m, 1H), 3.11 - 3.03 (m, 1H), 2.97 - 2.86 (m, 1H), 2.85 - 2.66 (m,

4H), 2.29 - 2.10 (m, 2H), 1.95 - 1.84 (m, 1H), 1.76 - 1.62 (m, 1H), 1.50 (quin, *J* = 7.5 Hz, 2H), 1.38 - 1.22 (m, 4H), 1.17 (q, *J* = 7.1 Hz, 2H).

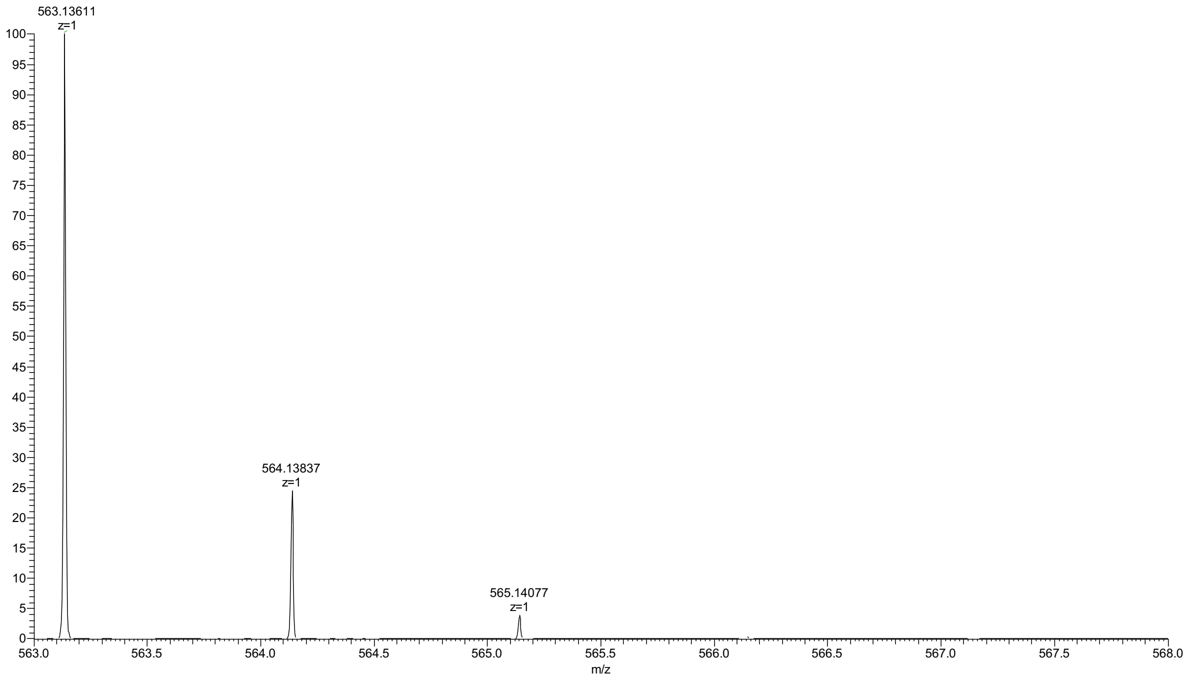

HRMS (ESI, m/z) of compound **s2b**.

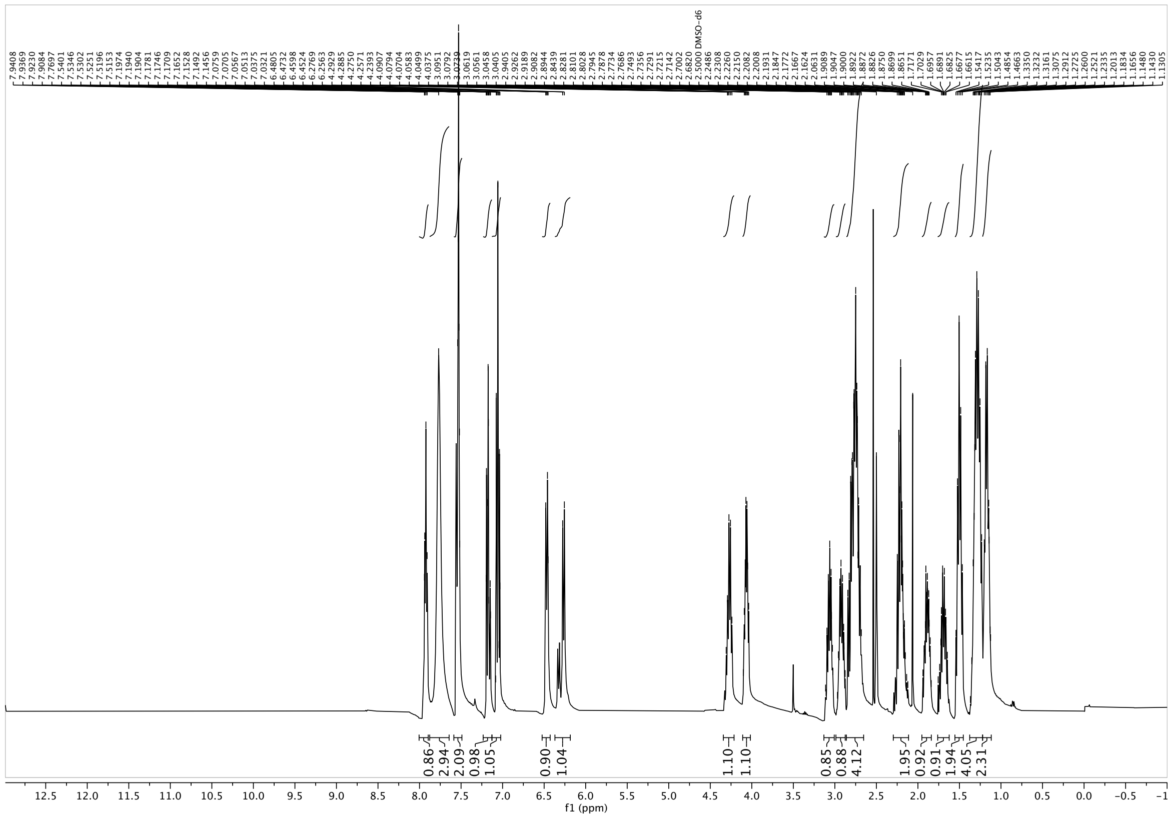

^{1}H NMR (400 MHz, DMSO-d_6) spectrum of compound **s2b**.

^{1}H NMR (400 MHz, DMSO-d_6): δ 7.93 (t, J = 5.6 Hz 1H), 7.77 (bs, 3H), 7.59 - 7.49 (m, 2H), 7.17 (tt, J = 7.8, 1.4 Hz, 1H), 7.06 (td, J = 7.7, 2.2 Hz, 1H), 6.47 (dd, J = 8.3, 2.9 Hz, 1H), 6.27 (d, J = 8.3 Hz, 1H), 4.33 - 4.22 (m, 1H), 4.11 - 4.02 (m, 1H), 3.12 - 3.02 (m, 1H), 2.99 - 2.88 (m, 1H), 2.86 - 2.67 (m, 4H),

2.30 - 2.11 (m, 2H), 1.95 - 1.84 (m, 1H), 1.76 - 1.62 (m, 1H), 1.50 (quin, J = 7.5 Hz, 2H), 1.38 - 1.22 (m, 4H), 1.17 (q, J = 7.1 Hz, 2H).

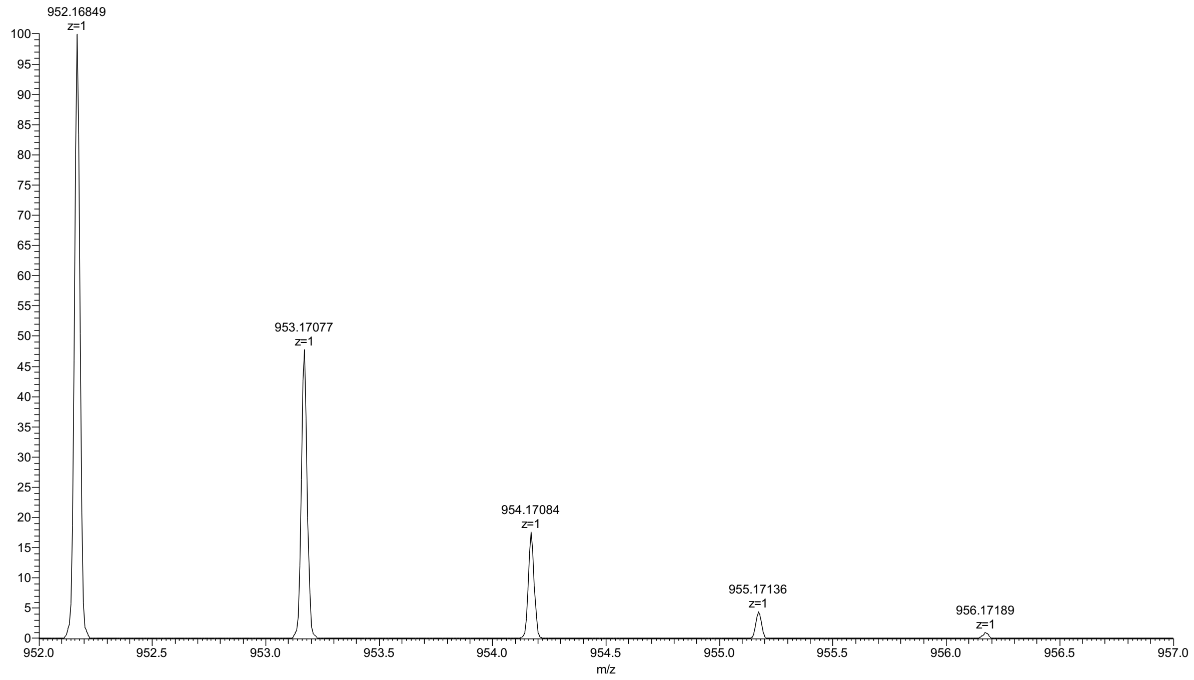

HRMS (ESI, m/z) of compound **1**.

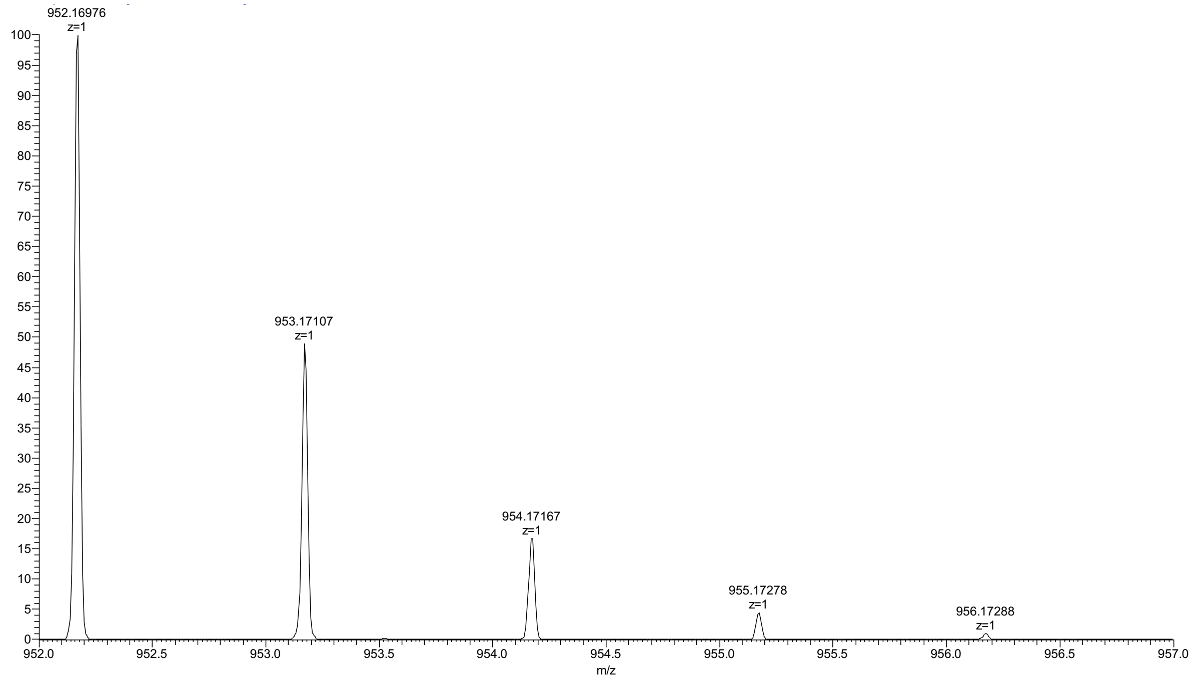

HRMS (ESI, m/z) of compound **2**.

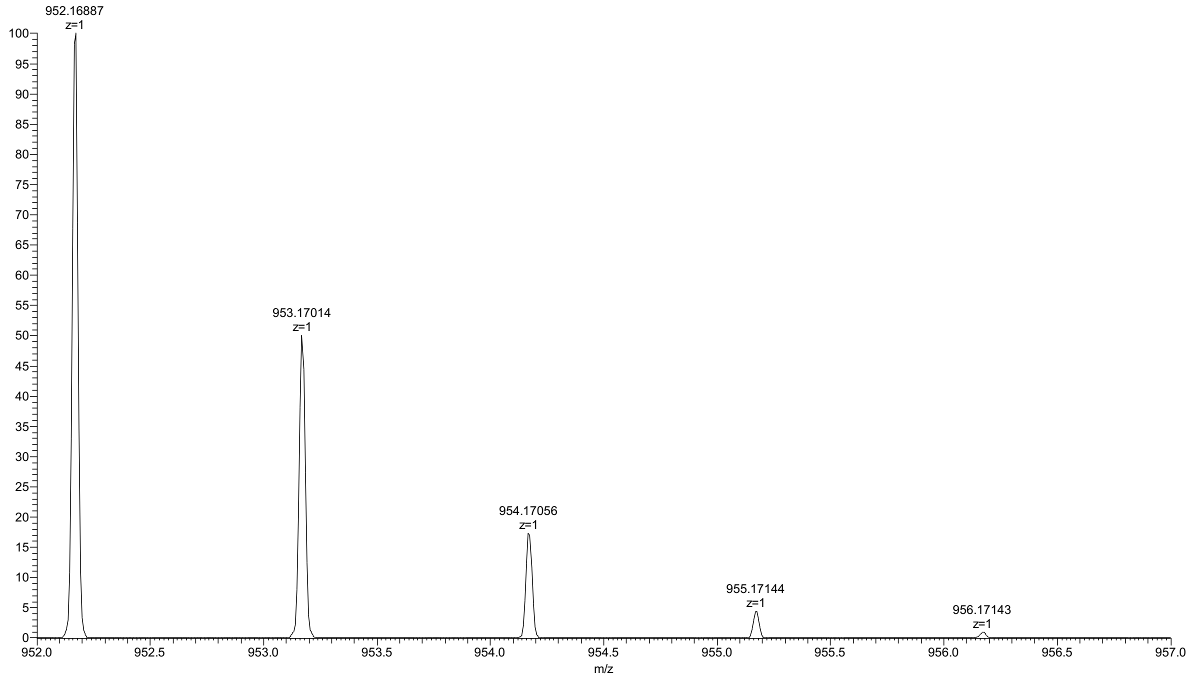

HRMS (ESI, m/z) of compound **3**.

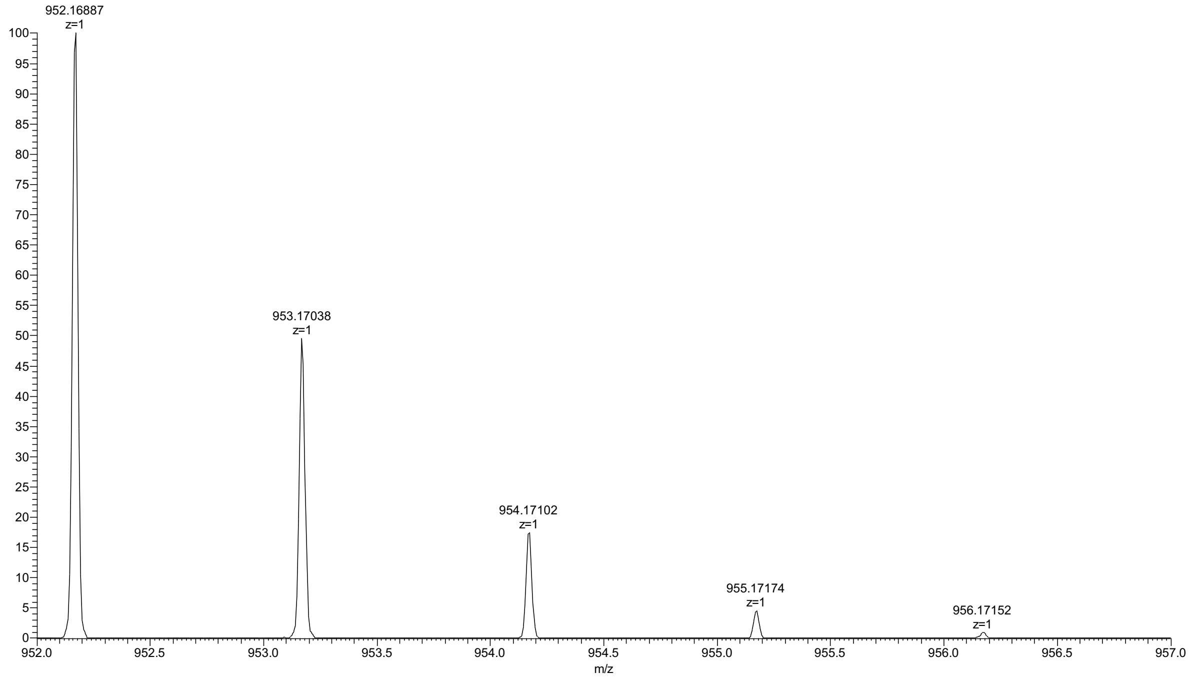

HRMS (ESI, m/z) of compound **4**.

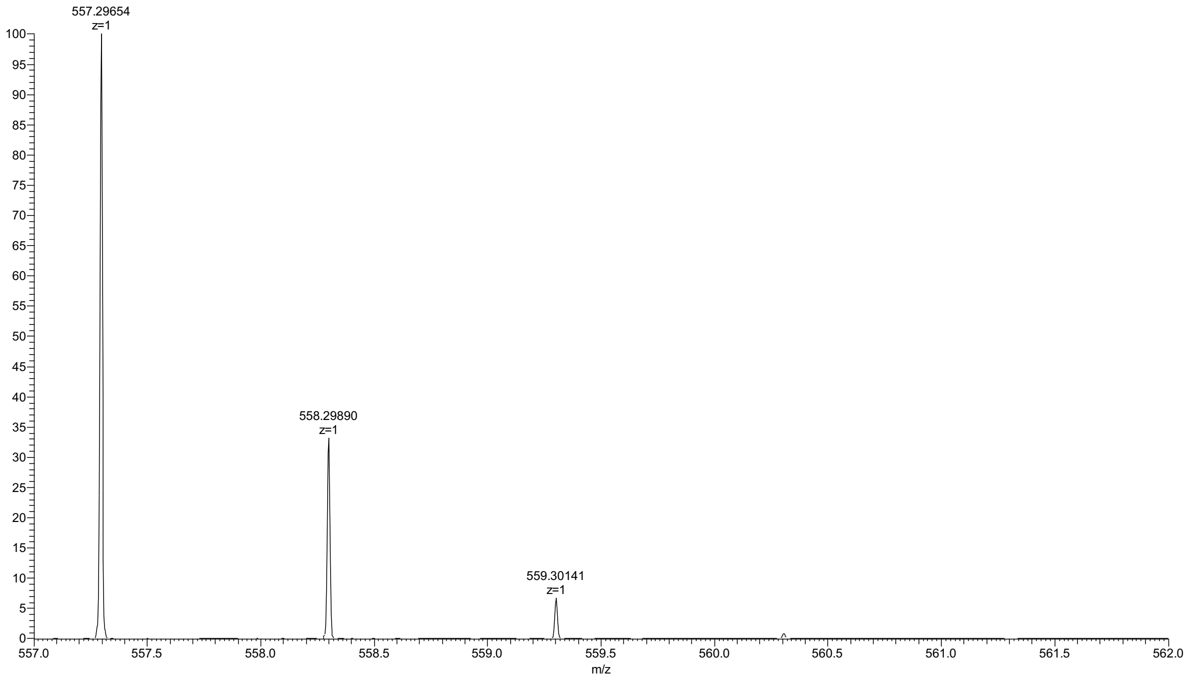

HRMS (ESI, m/z) of compound **s3a**.

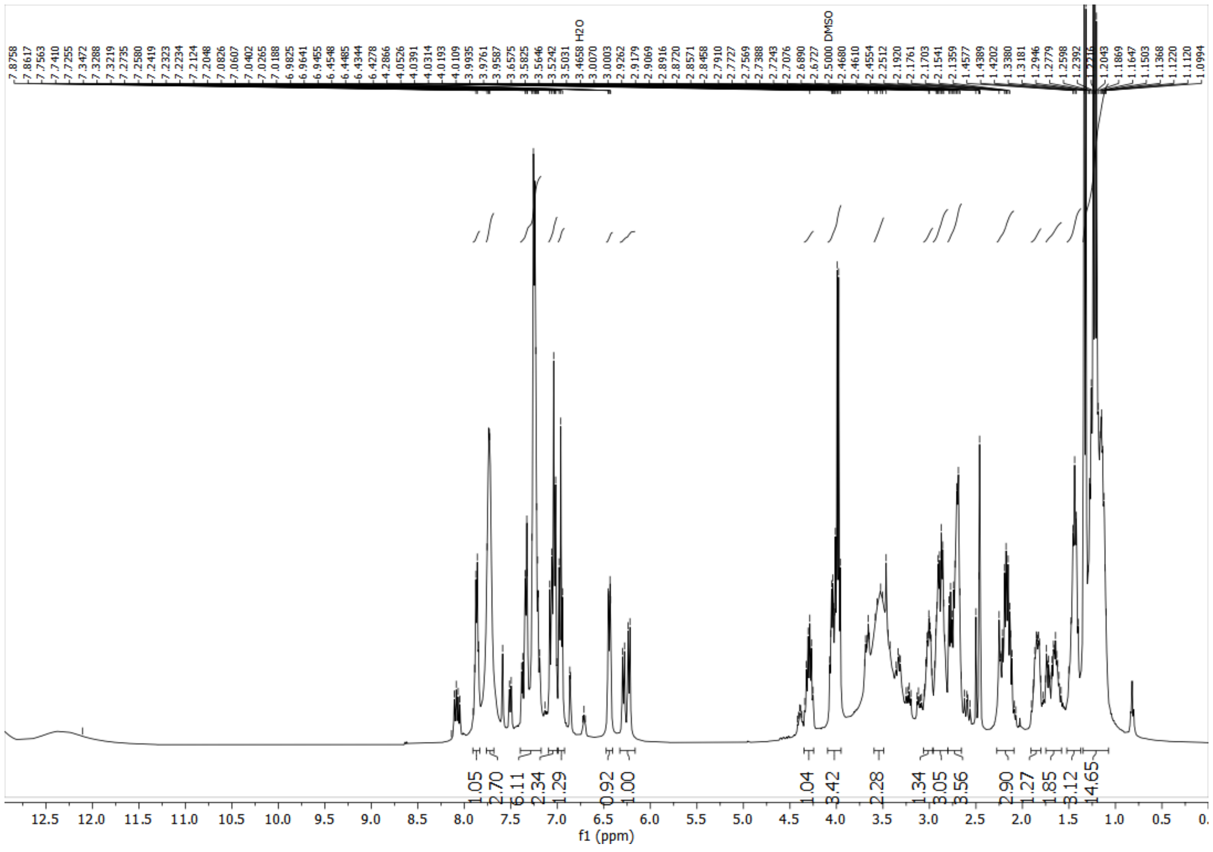

^{1}H NMR (400 MHz, DMSO-d_6) spectrum of compound **s3a**.

^{1}H NMR (400 MHz, DMSO-d_6) δ 7.91 (q, J = 5.6 Hz, 1H), 7.82 - 7.73 (m, 2H), 7.45 - 7.20 (m, 6H), 7.14 - 7.04 (m, 2H), 7.00 (t, J = 7.4 Hz, 1H), 6.48 (dd, J = 8.2, 2.6 Hz, 1H), 6.30 (dd, J = 23.7, 8.2 Hz, 1H), 4.33 (dq, J = 14.4, 7.2 Hz, 1H), 4.06 (dq, J = 29.1, 7.4, 6.9 Hz, 3H), 3.64 - 3.52 (m, 2H), 3.48 (s,

1H), 3.11 - 3.00 (m, 1H), 3.00 - 2.85 (m, 3H), 2.85 - 2.72 (m, 3H), 2.31 - 2.11 (m, 3H), 1.95 - 1.80 (m, 1H), 1.80 - 1.60 (m, 2H), 1.56 - 1.42 (m, 3H), 1.39 - 1.10 (m, 12H).

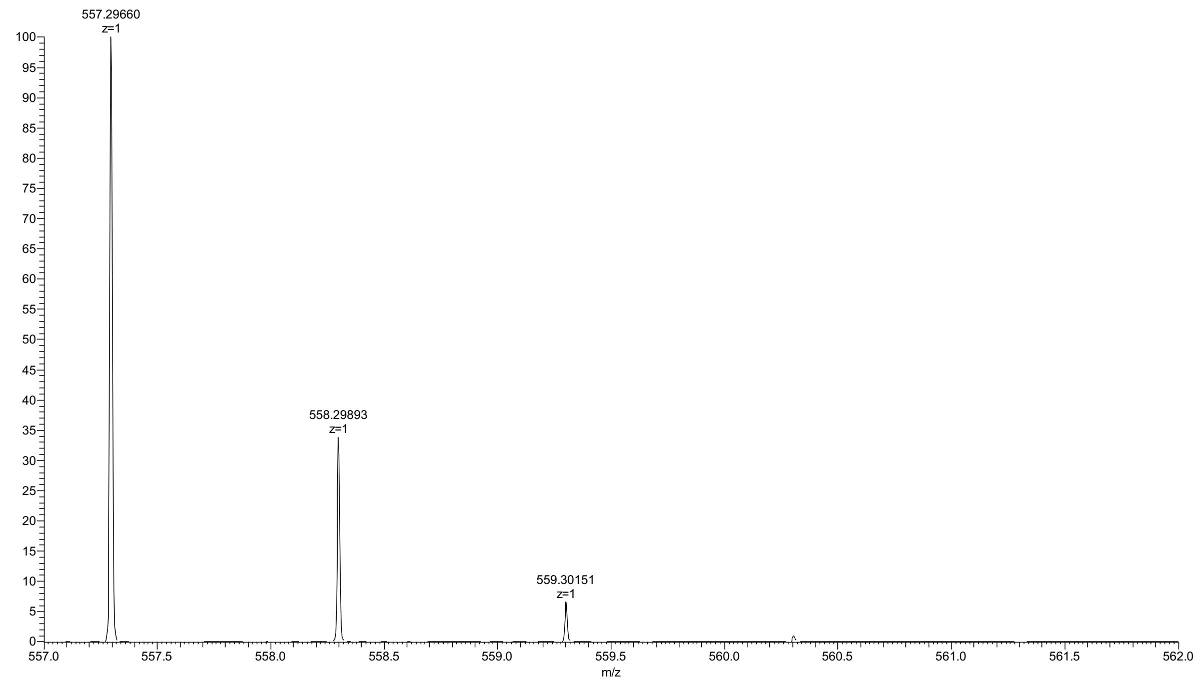

HRMS (ESI, m/z) of compound **s3b**.

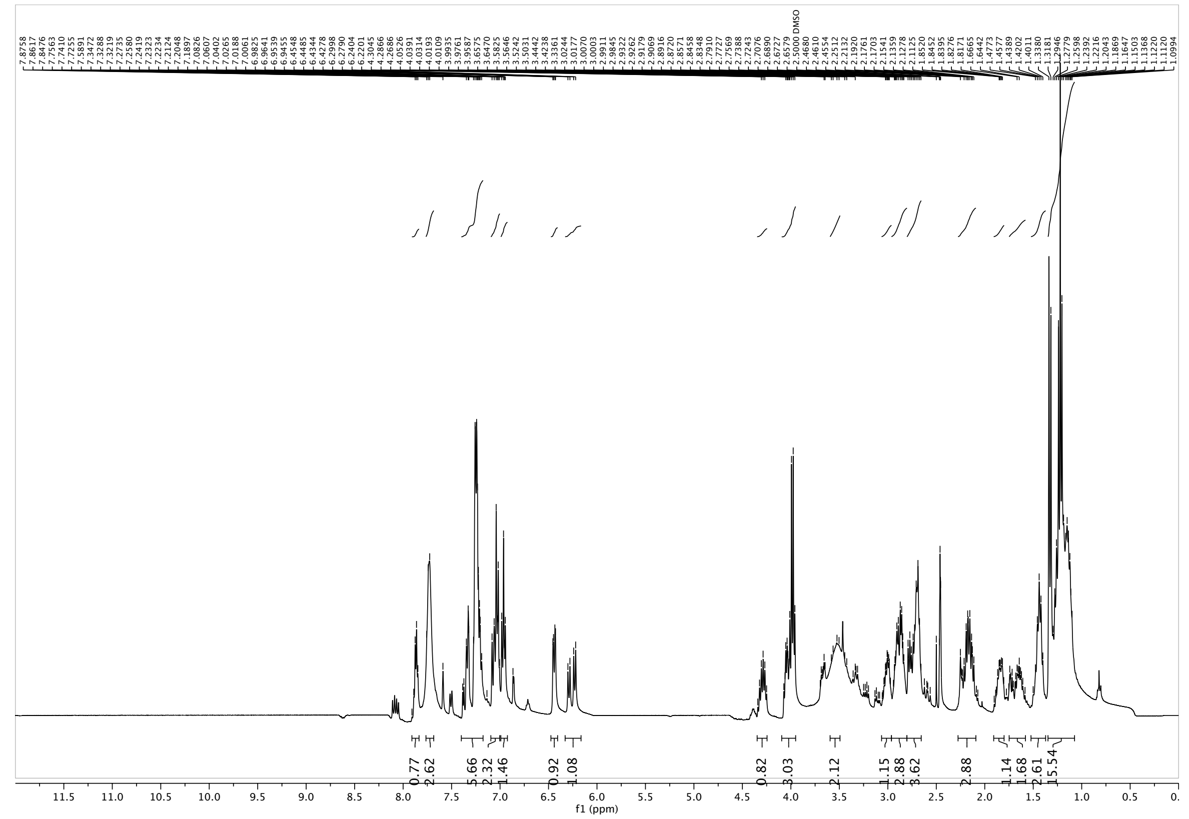

^{1}H NMR (400 MHz, DMSO-d_6) spectrum of compound **s3b**.

^{1}H NMR (400 MHz, DMSO-d_6) δ 7.93 (q, *J* = 5.6 Hz, 1H), 7.82 - 7.73 (m, 2H), 7.48 - 7.22 (m, 6H), 7.14 - 7.04 (m, 2H), 7.00 (t, *J* = 7.4 Hz, 1H), 6.49 (dd, *J* = 8.2, 2.6 Hz, 1H), 6.30 (dd, *J* = 23.7, 8.2 Hz, 1H), 4.35 (dq, *J* = 14.6, 7.8 Hz, 1H), 4.06 (dq, *J* = 29.1, 7.4, 6.9 Hz, 3H), 3.64 - 3.52 (m, 2H), 3.48 (s,

1H), 3.11 - 3.00 (m, 1H), 3.00 - 2.85 (m, 3H), 2.85 - 2.72 (m, 3H), 2.34 - 2.11 (m, 3H), 1.95 - 1.80 (m, 1H), 1.80 - 1.60 (m, 2H), 1.56 - 1.42 (m, 3H), 1.39 - 1.10 (m, 12H).

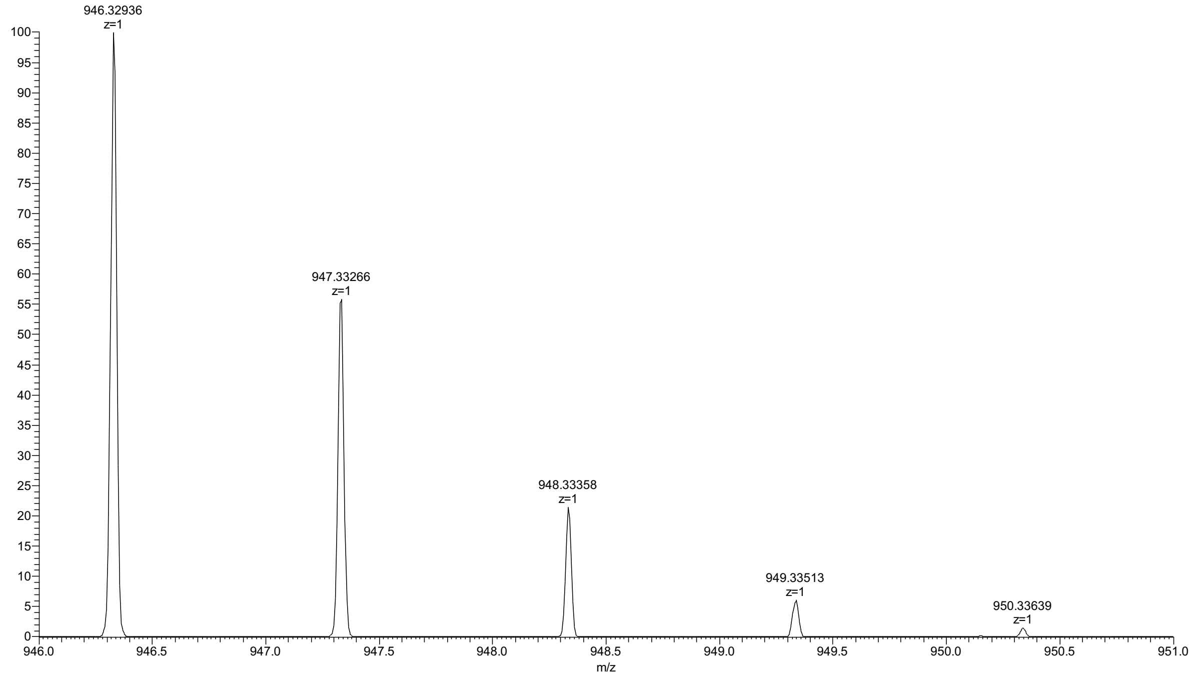

HRMS (ESI, m/z) of compound **5**.

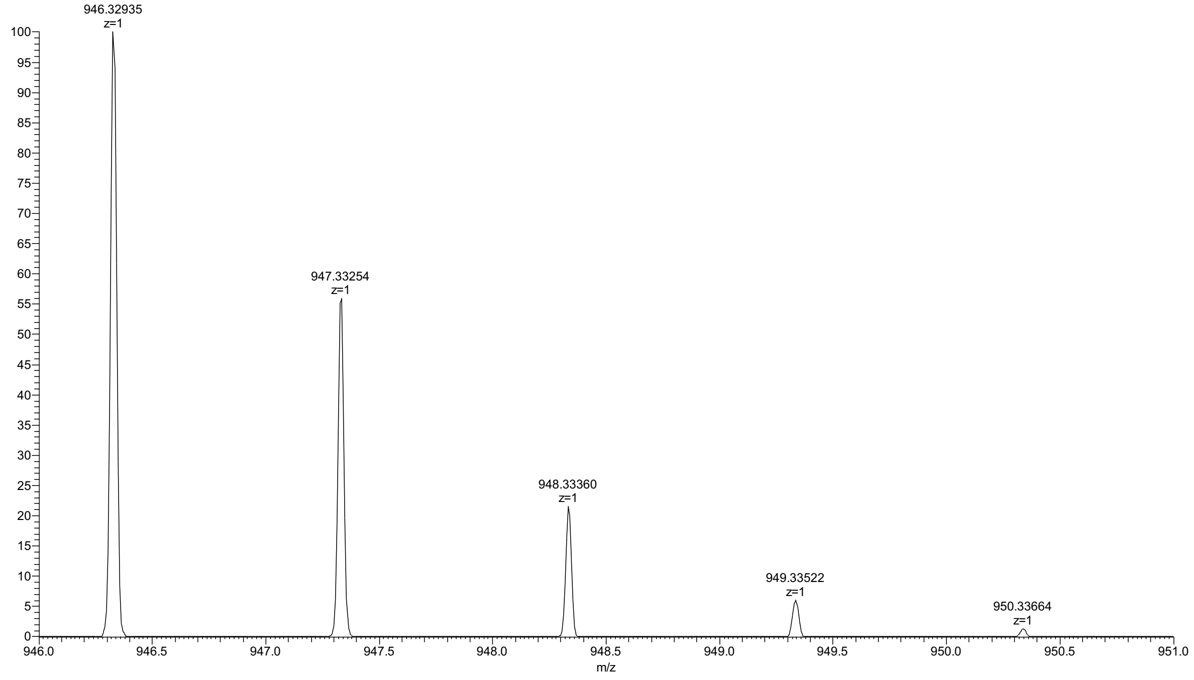

HRMS (ESI, m/z) of compound **6**.

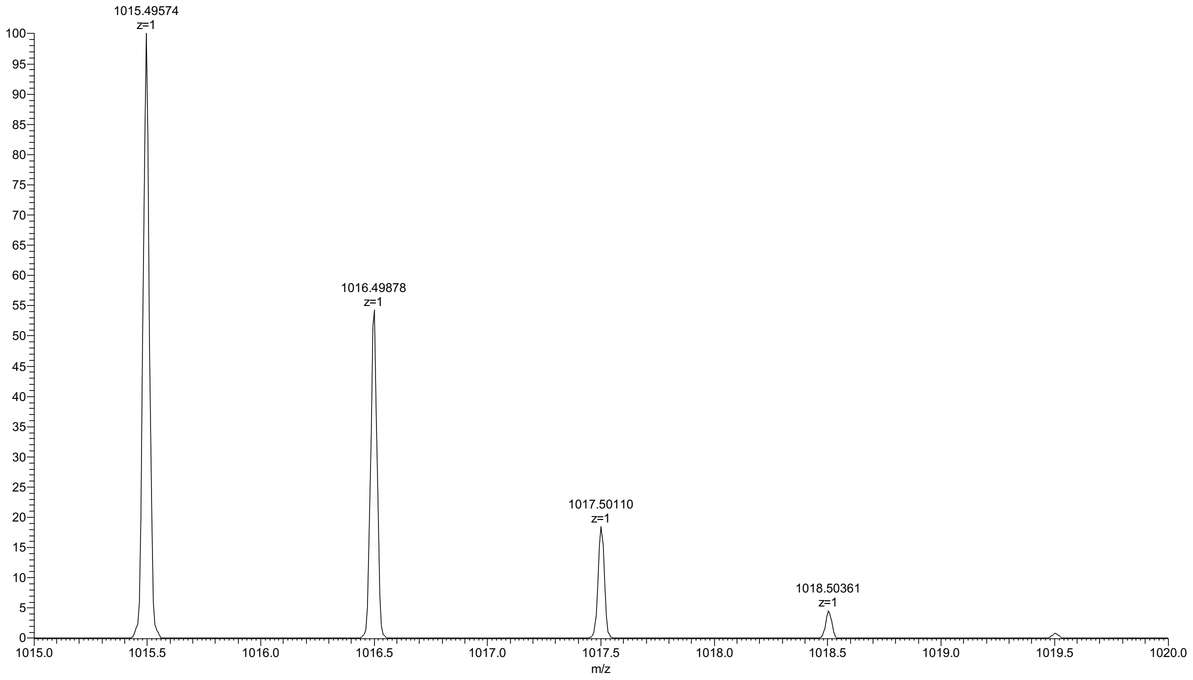

HRMS (ESI, m/z) of compound **7**.

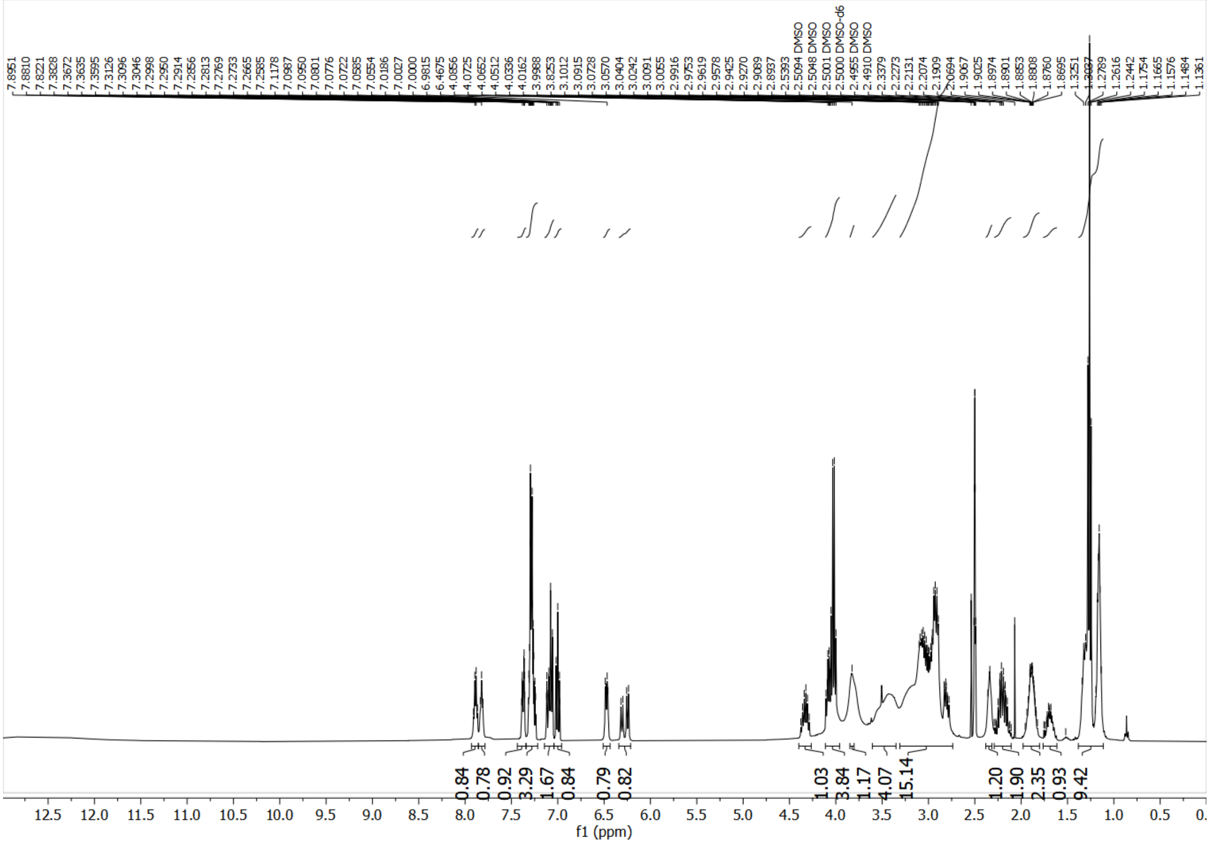

^{1}H NMR (400 MHz, DMSO-d_6) spectrum of compound **7**.

^{1}H NMR (400 MHz, DMSO-d_6) δ 7.89 (q, J = 5.6 Hz, 1H), 7.82 (t, J = 5.6 Hz, 1H), 7.44 - 7.34 (m, 1H), 7.34 - 7.22 (m, 4H), 7.14 - 7.04 (m, 2H), 7.00 (td, J = 7.4, 1.0 Hz, 1H), 6.47 (dd, J = 8.2, 3.7 Hz, 1H), 6.28 (dd, J = 24.5, 8.3 Hz, 1H), 4.33 (sextet, J = 14.3, 1H), 4.11 - 3.96 (m, 4H), 3.83 (s, 1H), 3.31 - 2.74

(m, 17H), 2.35 (bs, 1H), 2.29 - 2.11 (m, 2H), 1.98 - 1.80 (m, 3H), 1.76 - 1.61 (m, *J* = 14.0, 8.3, 5.5 Hz, 1H), 1.38 - 1.11 (m, 11H).

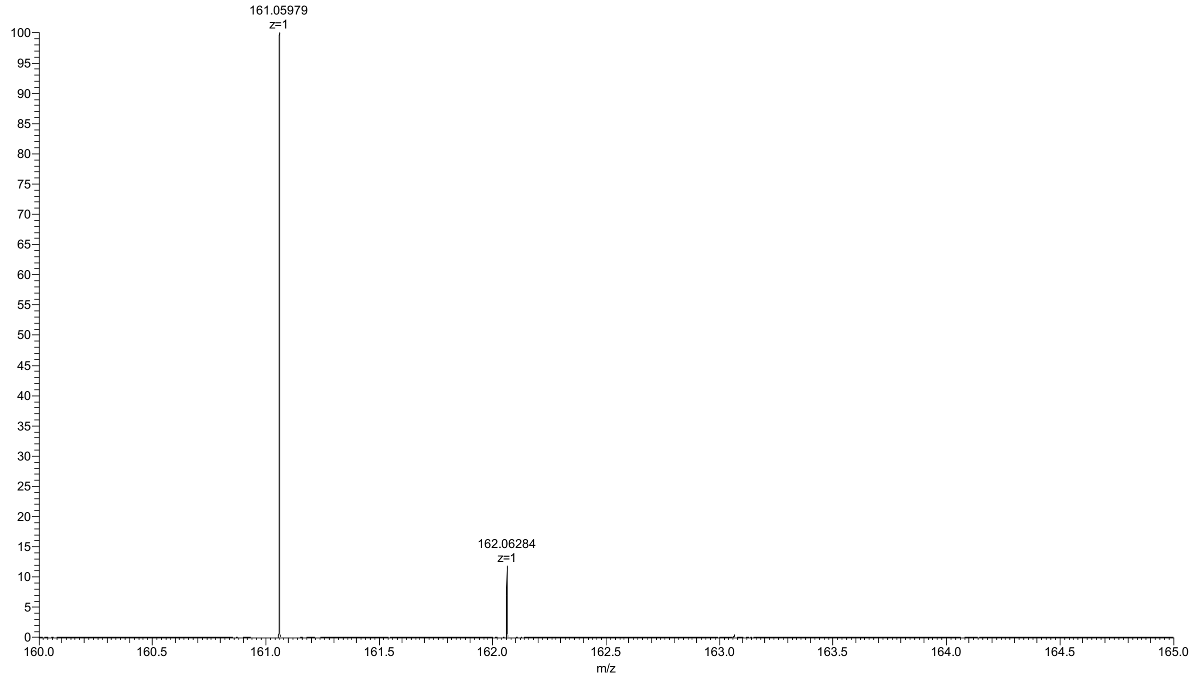

HRMS (ESI, m/z) of compound **s4**.

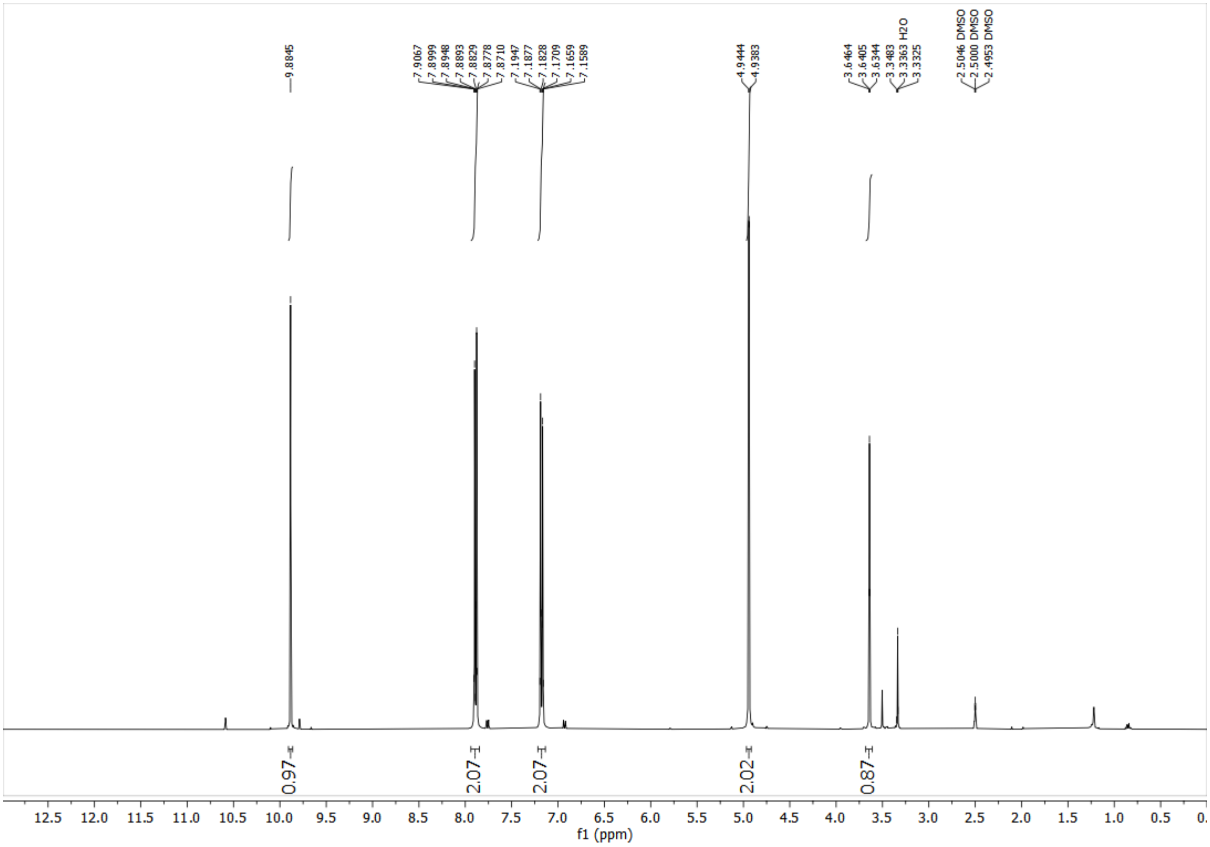

^{1}H NMR (400 MHz, DMSO-d_6) spectrum of compound **s4**.

^{1}H NMR (400 MHz, DMSO-d_6) δ 9.88 (s, 1H), 7.94 - 7.85 (m, 2H), 7.22 - 7.14 (m, 2H), 4.94 (d, J = 2.4 Hz, 2H), 3.64 (t, J = 2.4 Hz, 1H).

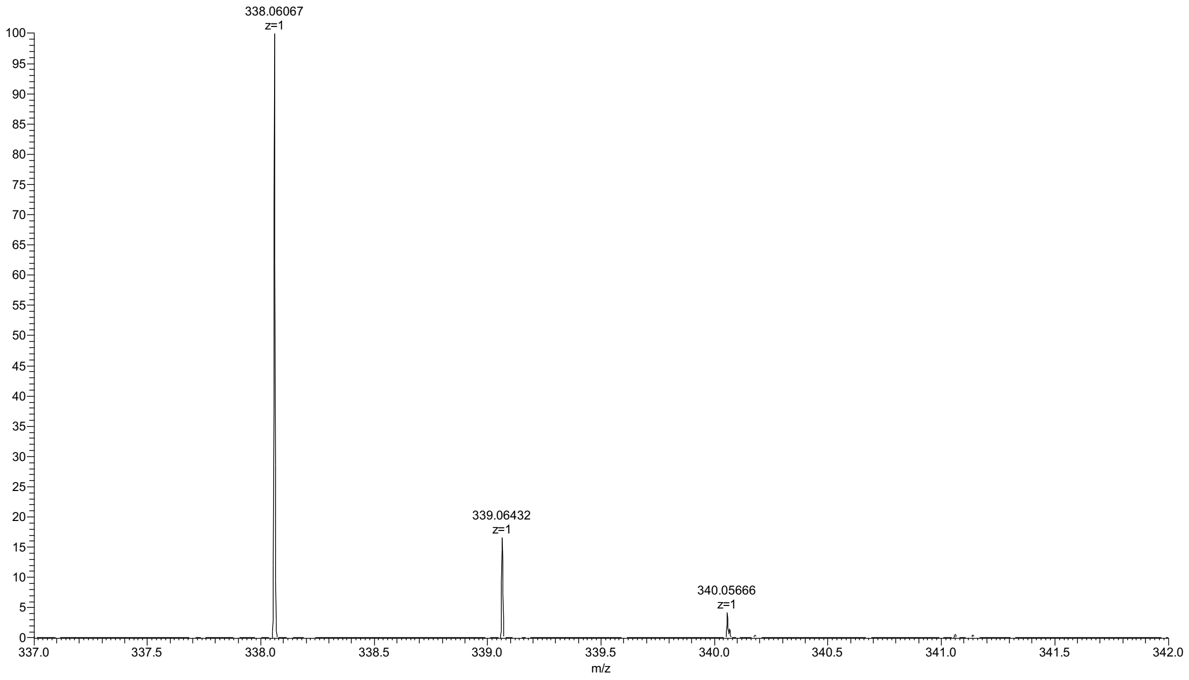

HRMS (ESI, m/z) of compound **s5**.

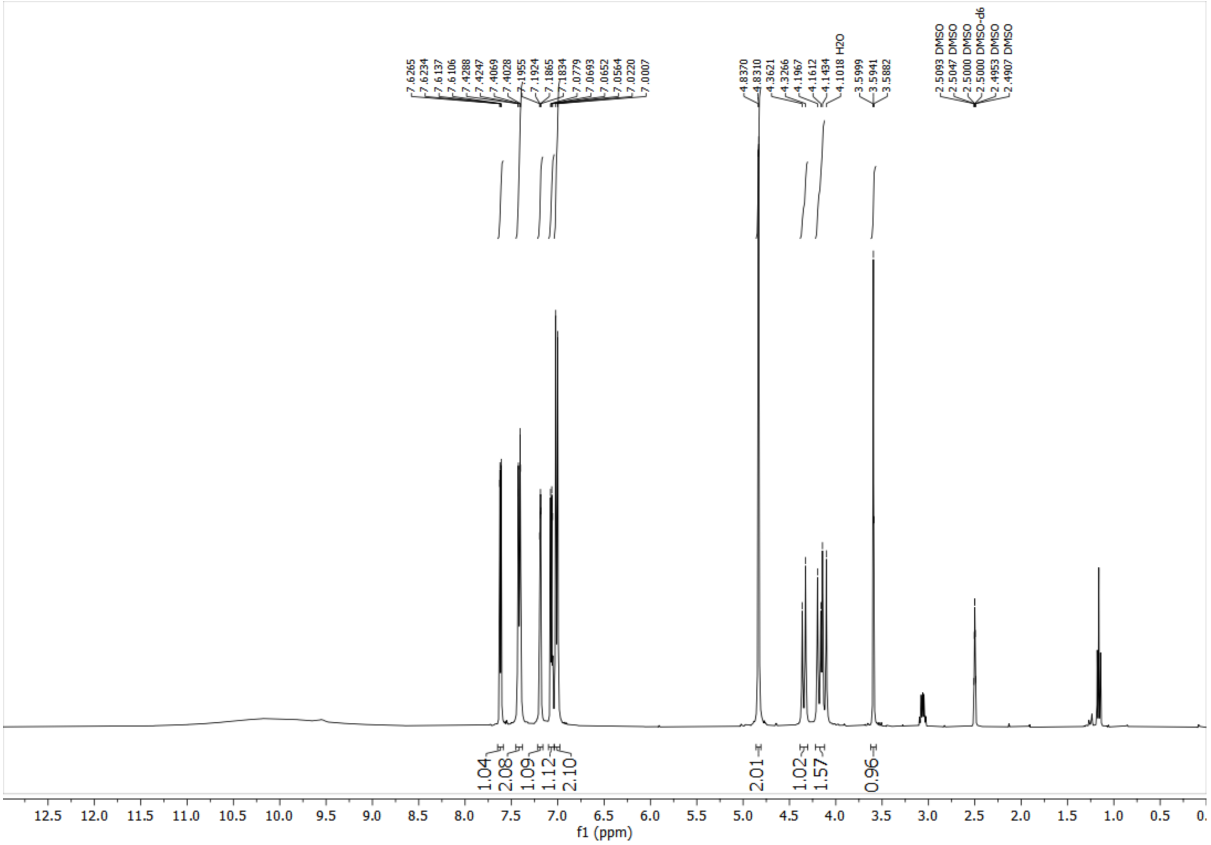

^{1}H NMR (400 MHz, DMSO-d_6) spectrum of compound **s5**.

^{1}H NMR (400 MHz, DMSO-d_6) δ 7.62 (dd, J = 5.1, 1.2 Hz, 1H), 7.45 - 7.38 (m, 2H), 7.19 (dd, J = 3.6, 1.2 Hz, 1H), 7.07 (dd, J = 5.1, 3.5 Hz, 1H), 7.01 (d, J = 8.5 Hz, 2H), 4.83 (d, J = 2.4 Hz, 2H), 4.34 (d, J = 14.2 Hz, 1H), 4.15 (dd, J = 22.5, 15.4 Hz, 2H), 3.59 (t, J = 2.3 Hz, 1H).

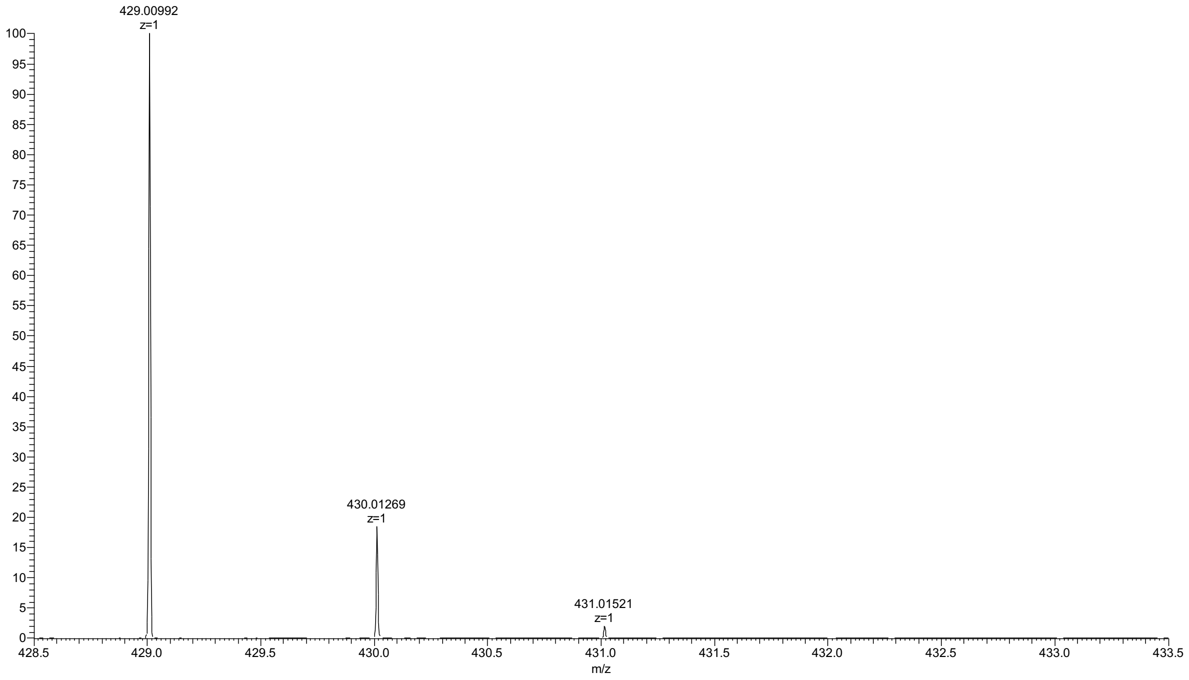

HRMS (ESI, m/z) of compound **s6a**.

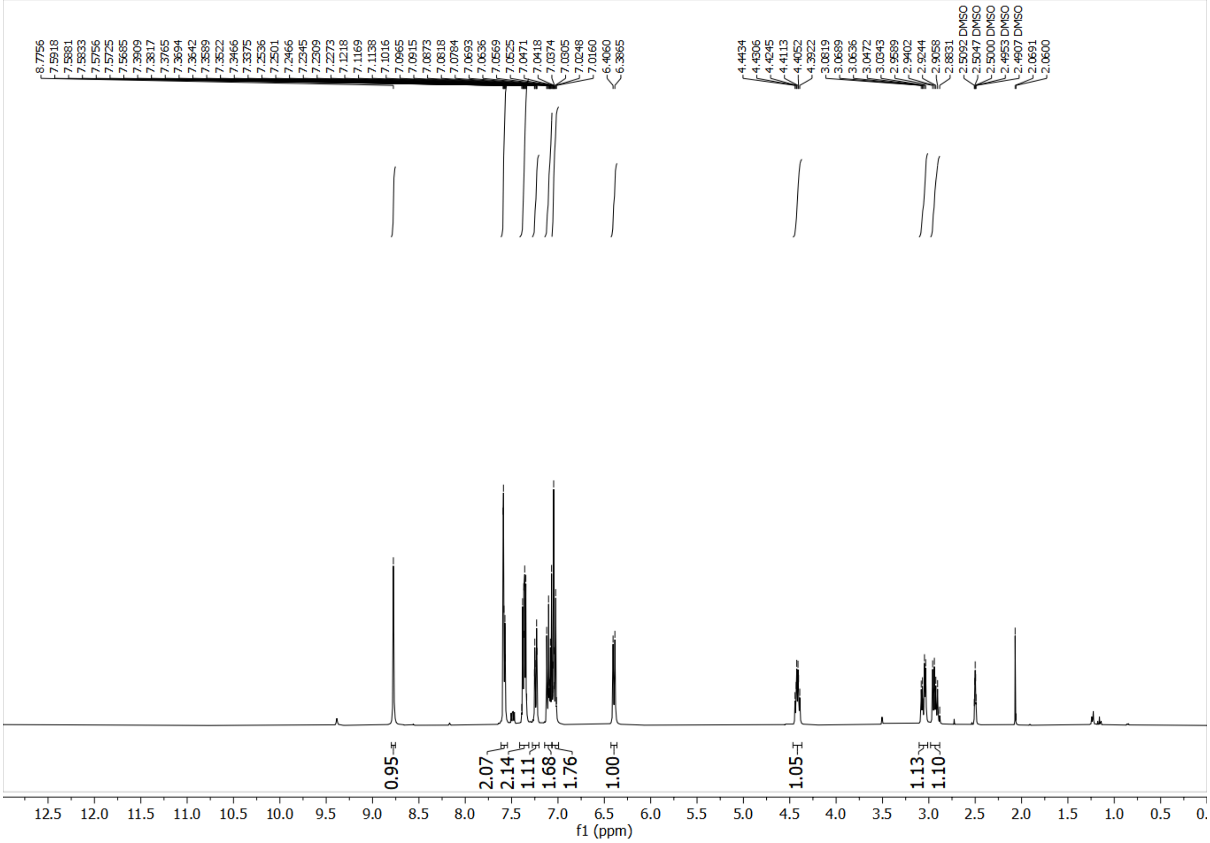

^{1}H NMR (400 MHz, DMSO-d_6) spectrum of compound **s6a**.

^{1}H NMR (400 MHz, DMSO-d_6) δ 8.78 (s, 1H), 7.58 (dt, J = 5.9, 1.6 Hz, 2H), 7.41 - 7.32 (m, 2H), 7.24 (dt, J = 7.7, 1.4 Hz, 1H), 7.14 - 7.00 (m, 3H), 6.40 (d, J = 7.8 Hz, 1H), 4.42 (td, J = 7.6, 5.2 Hz, 1H), 3.06 (dd, J = 13.9, 5.2 Hz, 1H), 2.93 (dd, J = 13.8, 7.5 Hz, 1H).

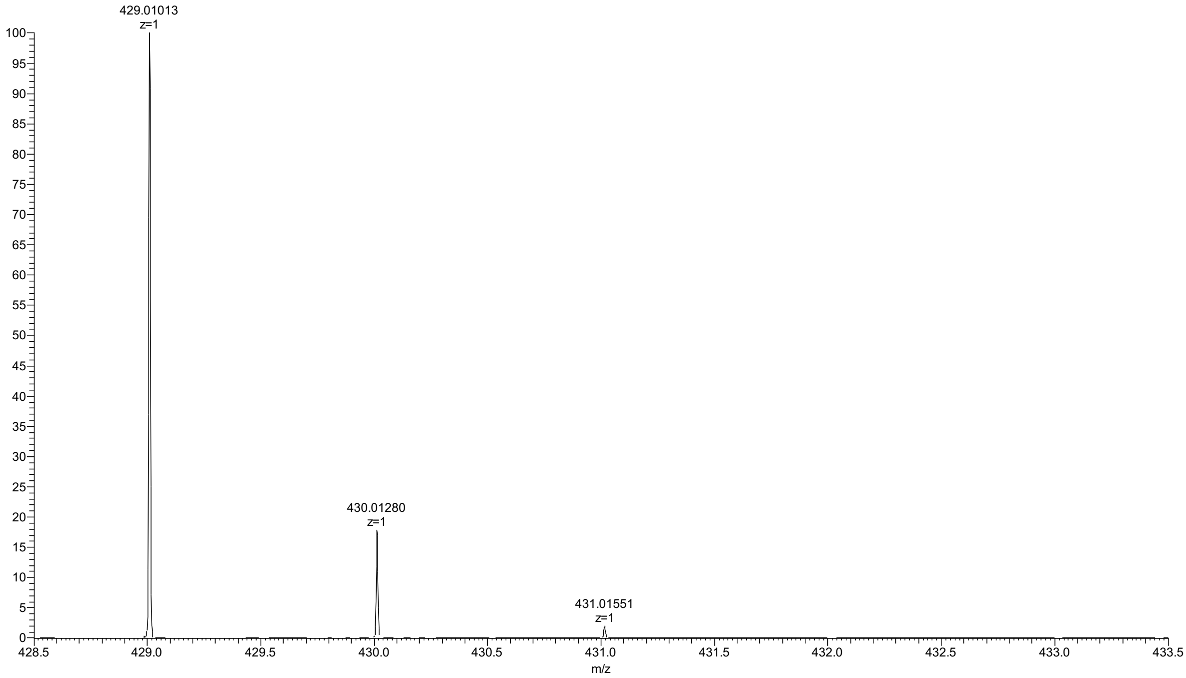

HRMS (ESI, m/z) of compound **s6b**.

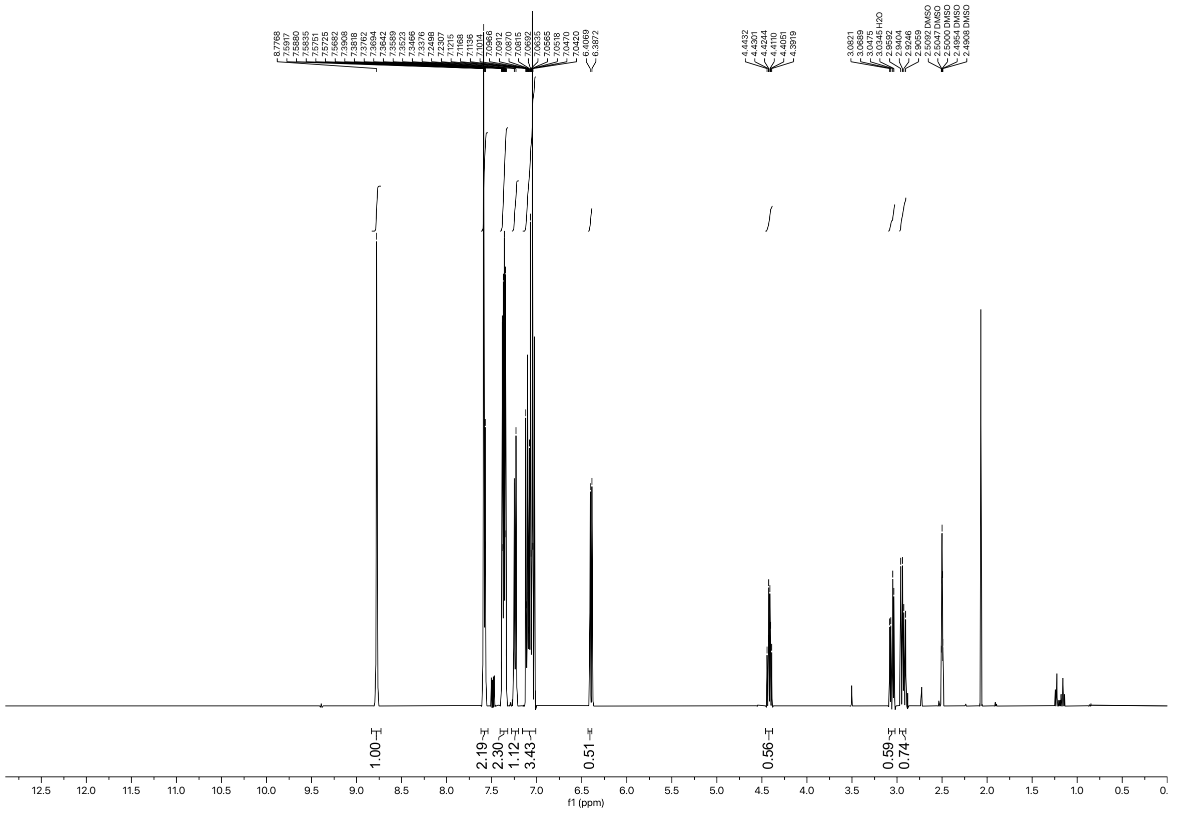

^{1}H NMR (400 MHz, DMSO-d_6) spectrum of compound **s6b**.

^{1}H NMR (400 MHz, DMSO-d_6) δ 8.78 (s, 1H), 7.58 (dt, J = 5.8, 1.7 Hz, 2H), 7.41 - 7.32 (m, 2H), 7.24 (dt, J = 7.6, 1.3 Hz, 1H), 7.14 - 7.00 (m, 3H), 6.40 (d, J = 7.8 Hz, 1H), 4.42 (td, J = 7.6, 5.2 Hz, 1H), 3.06 (dd, J = 13.9, 5.2 Hz, 1H), 2.93 (dd, J = 13.8, 7.5 Hz, 1H).

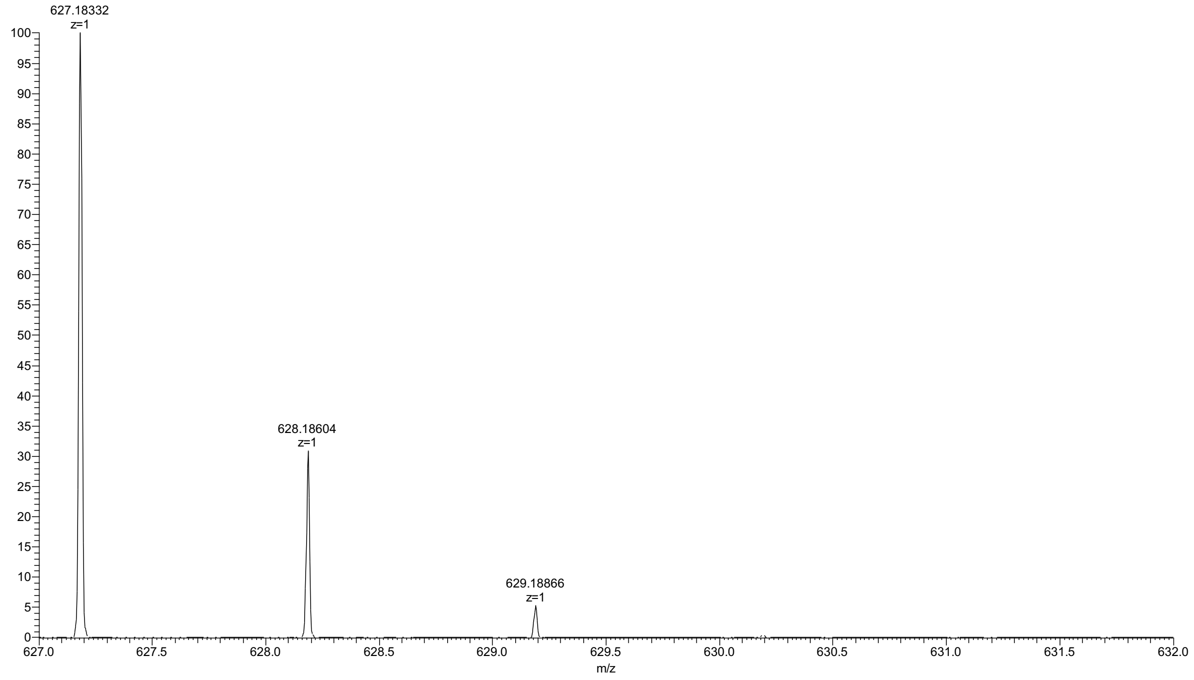

HRMS (ESI, m/z) of compound **s7a**.

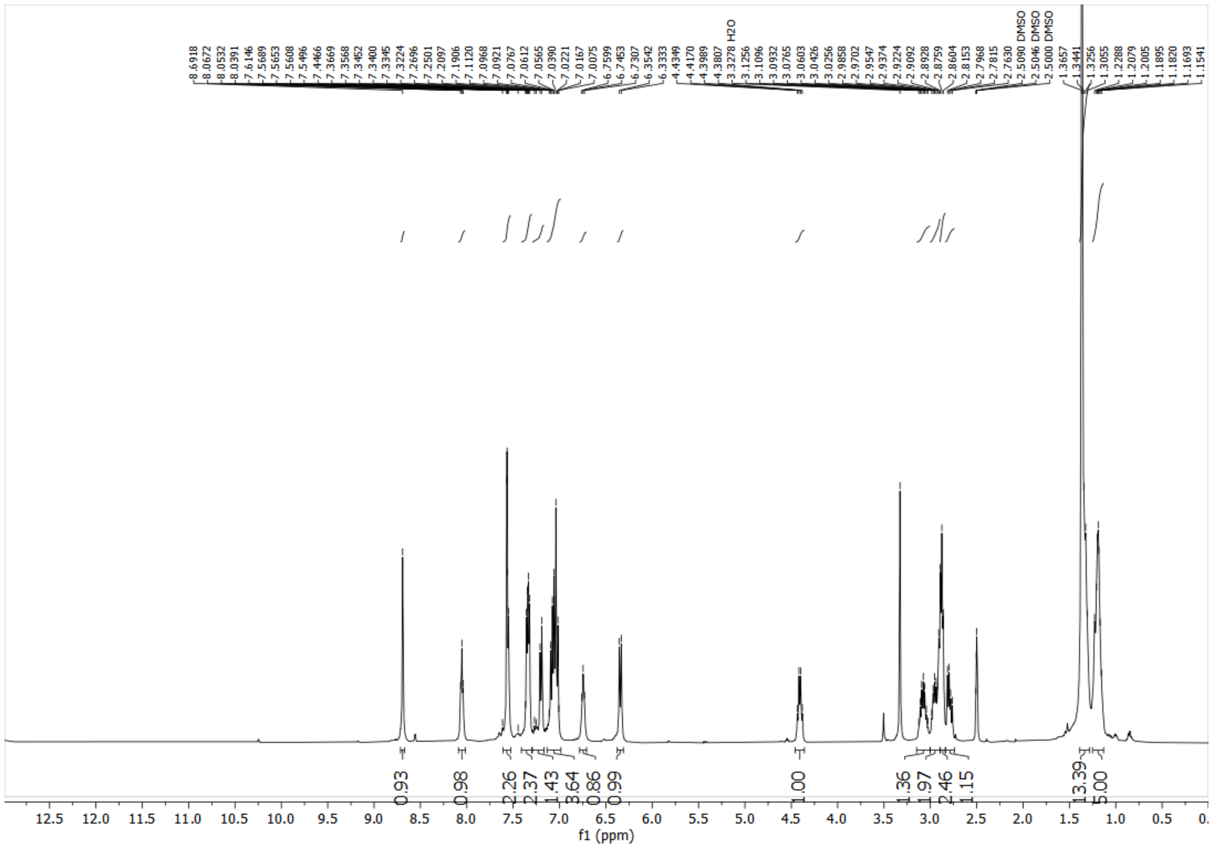

^{1}H NMR (400 MHz, DMSO-d_6) spectrum of compound **s7a**.

^{1}H NMR (400 MHz, DMSO-d_6) δ 8.69 (s, 1H), 8.05 (t, J = 5.6 Hz, 1H), 7.59 - 7.52 (m, 2H), 7.38 - 7.30 (m, 2H), 7.20 (d, J = 7.7 Hz, 1H), 7.10 - 6.98 (m, 3H), 6.75 (t, J = 5.8 Hz, 1H), 6.34 (d, J = 8.4 Hz, 1H),

4.41 (q, *J* = 7.2 Hz, 1H), 3.13 - 3.00 (m, 1H), 3.00 - 2.83 (m, 4H), 2.82 - 2.73 (m, 1H), 1.44 - 1.25 (m, 14H), 1.25 - 1.11 (m, 5H).

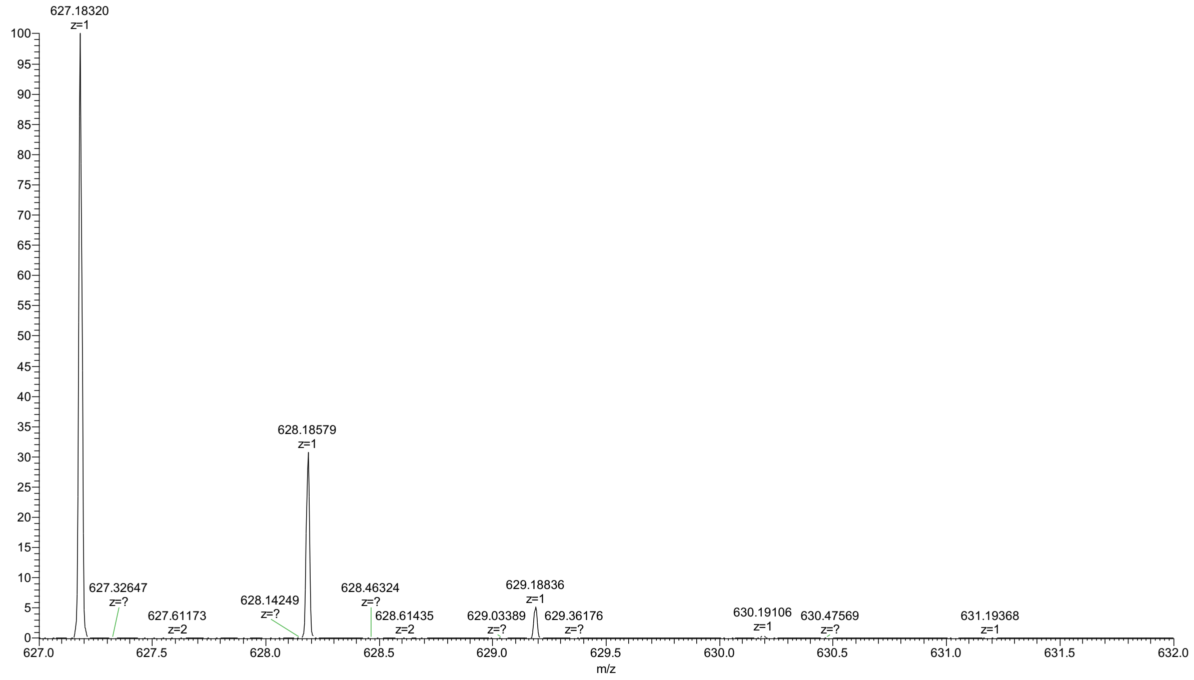

HRMS (ESI, m/z) of compound **s7b**.

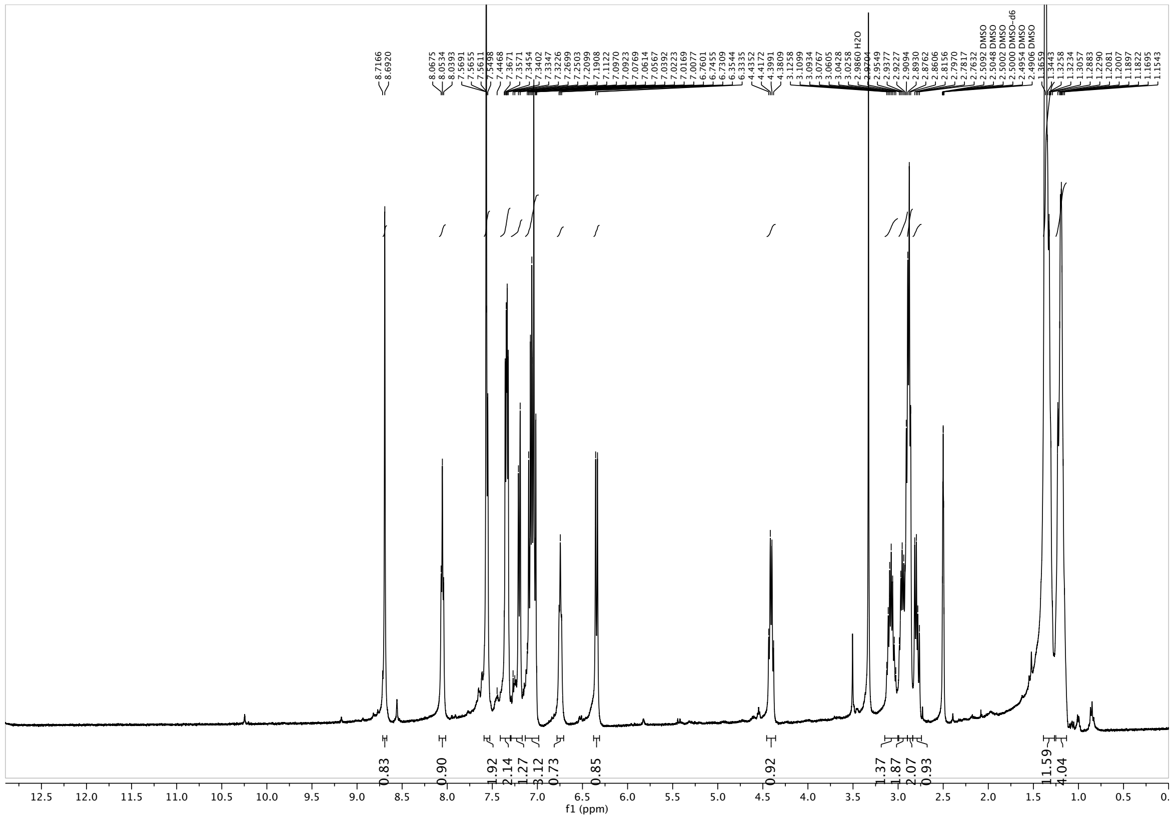

^{1}H NMR (400 MHz, DMSO-d_6) spectrum of compound **s7b**.

^{1}H NMR (400 MHz, DMSO-d_6) δ 8.69 (s, 1H), 8.05 (t, J = 5.6 Hz, 1H), 7.59 - 7.52 (m, 2H), 7.38 - 7.30 (m, 2H), 7.20 (d, J = 7.7 Hz, 1H), 7.10 - 6.98 (m, 3H), 6.75 (t, J = 5.8 Hz, 1H), 6.34 (d, J = 8.4 Hz, 1H),

4.41 (q, J = 7.2 Hz, 1H), 3.13 - 3.00 (m, 1H), 3.00 - 2.83 (m, 4H), 2.82 - 2.73 (m, 1H), 1.44 - 1.25 (m, 14H), 1.25 - 1.11 (m, 5H).

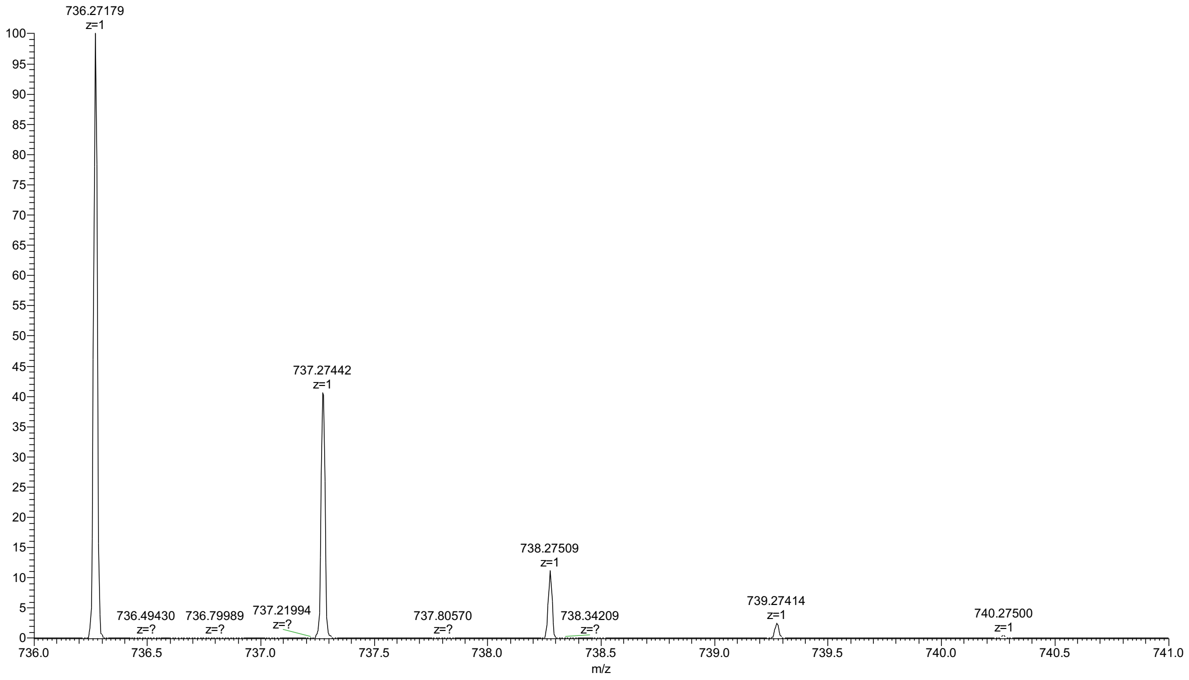

HRMS (ESI, m/z) of compound **s8a**.

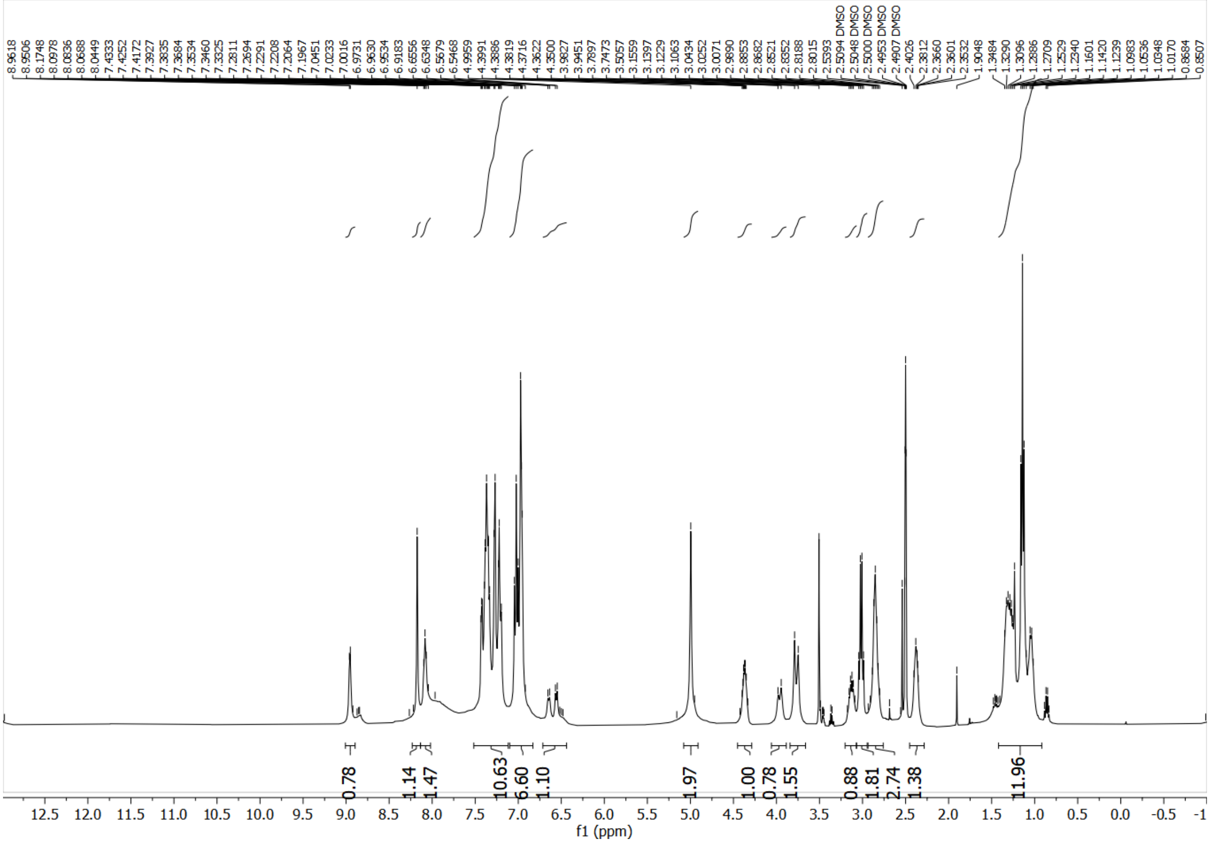

^{1}H NMR (400 MHz, DMSO-d_6) spectrum of compound **s8a**.

^{1}H NMR (400 MHz, DMSO-d_6) δ 8.96 (bs, 1H), 8.17 (s, 1H), 8.08 (bs, 1H), 7.46 - 7.14 (m, 10H), 7.06 - 6.87 (m, 7H), 5.00 (s, 2H), 4.42 - 4.32 (m, 1H), 3.96 (d, J = 14.5 Hz, 1H), 3.77 (d, J = 16.8 Hz, 2H), 3.19 - 3.08 (m, 1H), 3.02 (q, J = 7.2 Hz, 2H), 2.93 - 2.78 (m, 3H), 2.38 (bs, 1H), 1.38 - 0.97 (m, 12H).

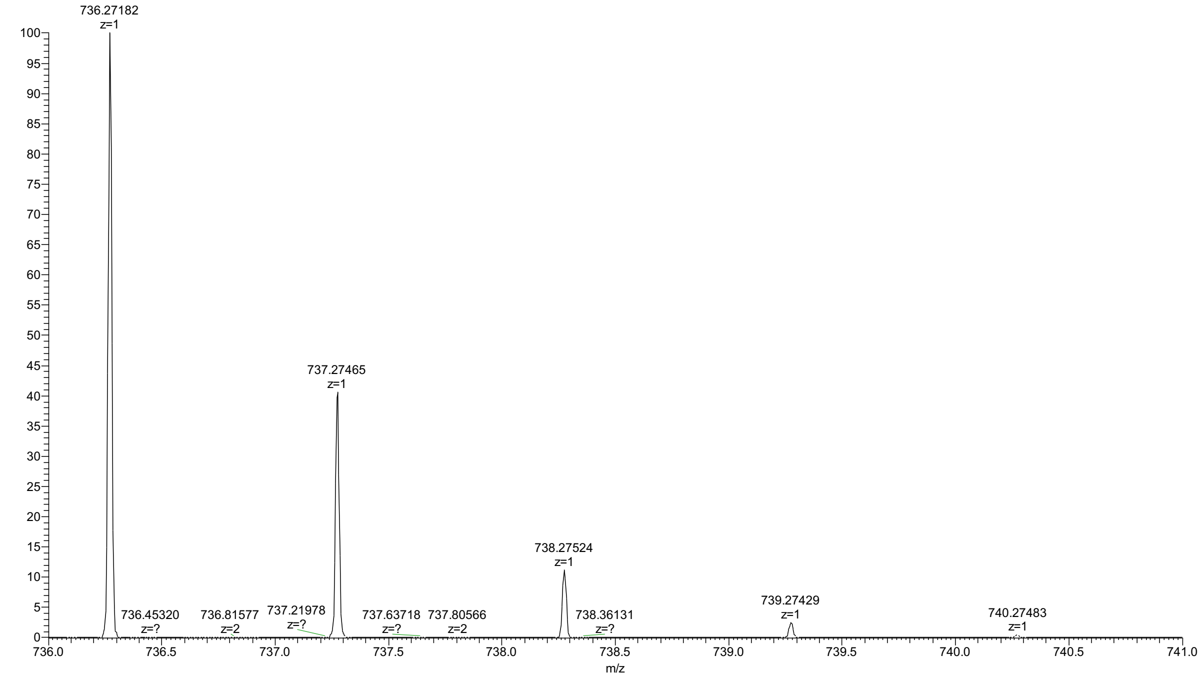

HRMS (ESI, m/z) of compound **s8b**.

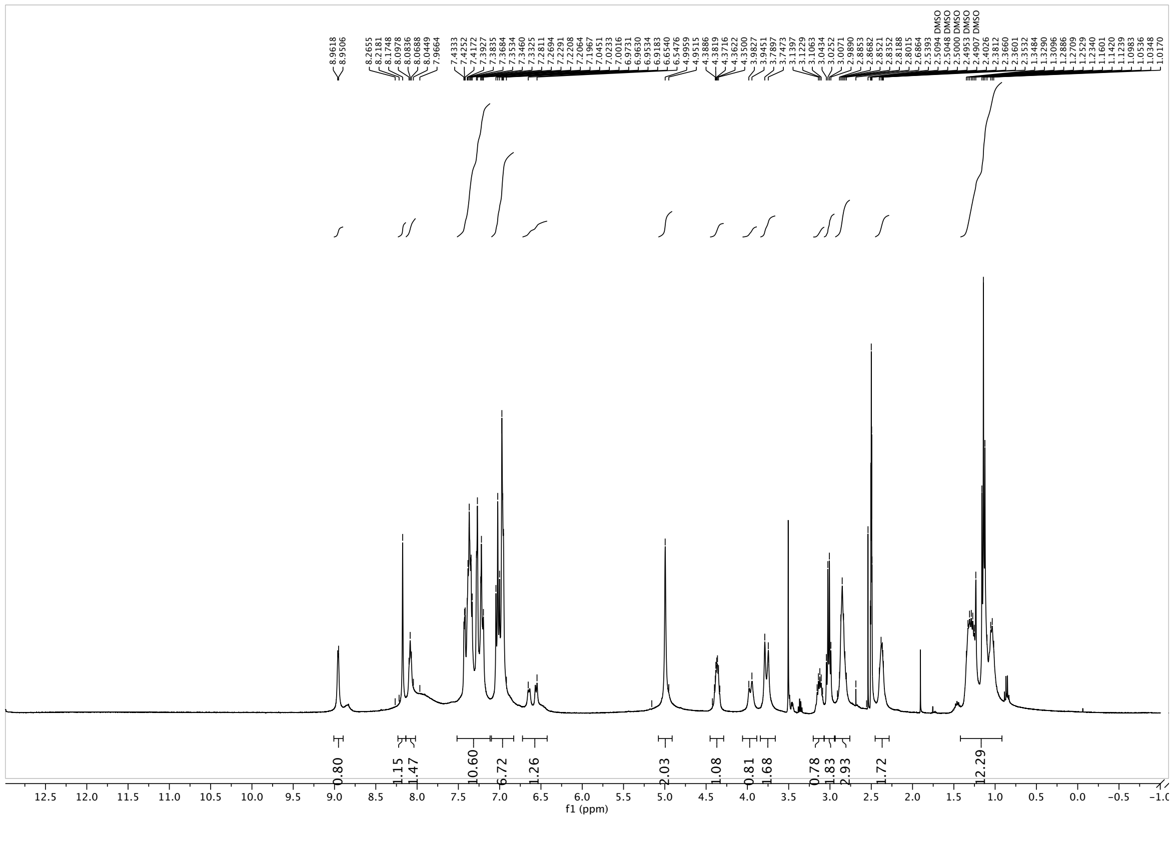

^{1}H NMR (400 MHz, DMSO-d_6) spectrum of compound **s8b**.

^{1}H NMR (400 MHz, DMSO-d_6) δ 8.94 (bs, 1H), 8.18 (s, 2H), 8.10 (bs, 2H), 7.44 - 7.11 (m, 12H), 7.09 - 6.78 (m, 8H), 5.04 (s, 2H), 4.41 - 4.31 (m, 1H), 3.95 (d, J = 14.5 Hz, 1H), 3.79 (d, J = 16.3 Hz, 2H), 3.19 - 3.08 (m, 1H), 3.03 (q, J = 7.4 Hz, 2H), 2.95 - 2.76 (m, 3H), 2.36 (bs, 2H), 1.35 - 0.97 (m, 14H).

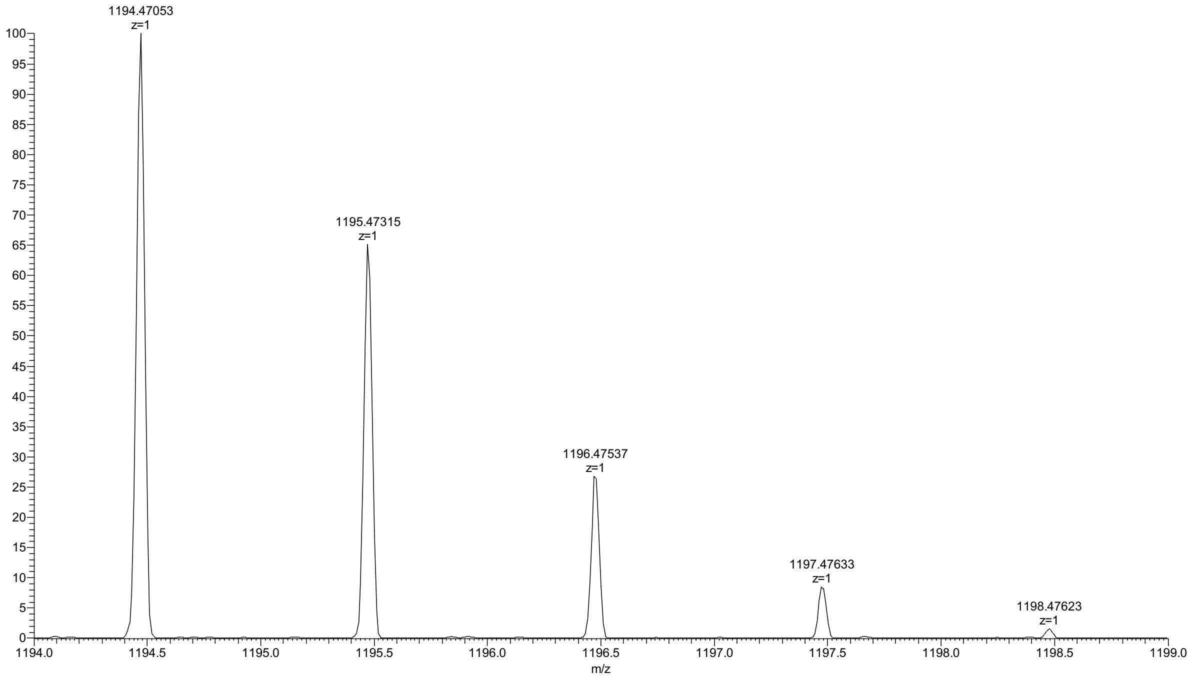

HRMS (ESI, m/z) of compound **14**.

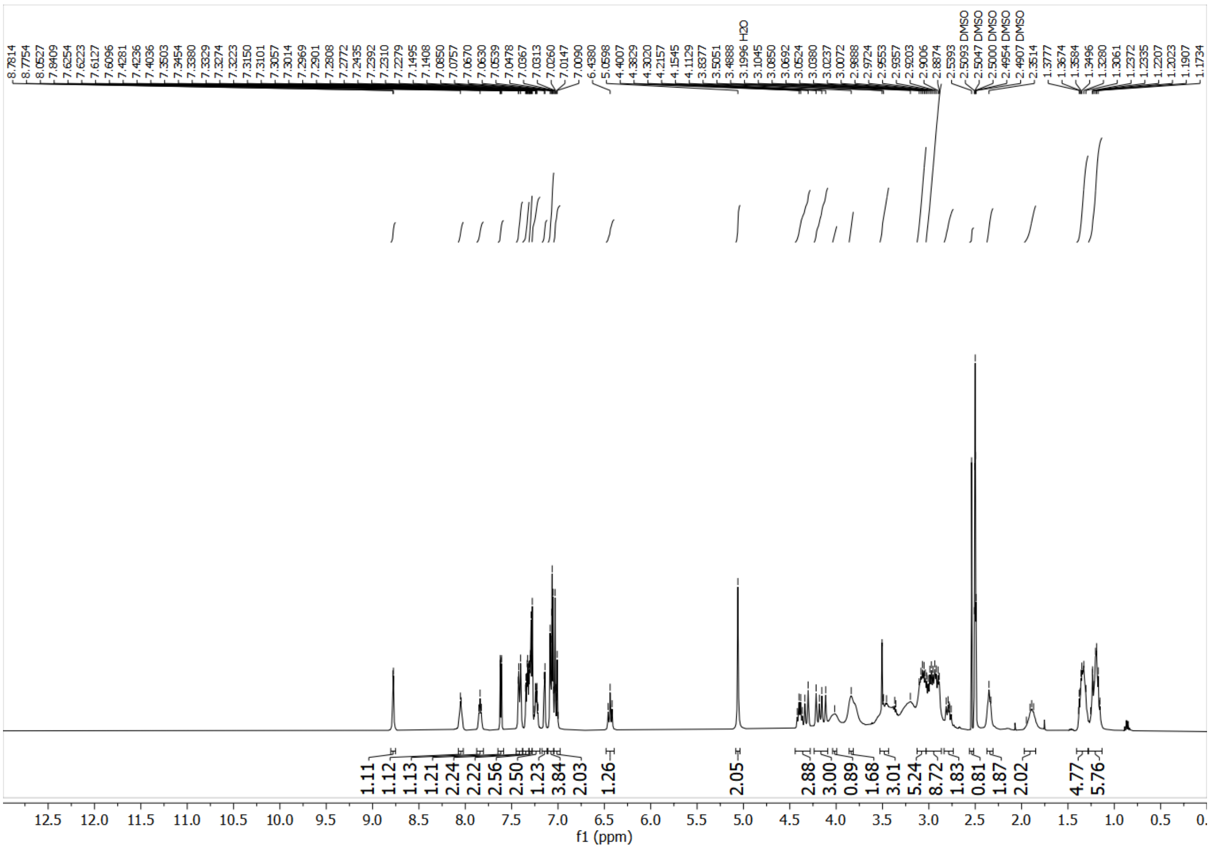

^{1}H NMR (400 MHz, DMSO-d_6) spectrum of compound **14**.

^{1}H NMR (400 MHz, DMSO-d_6) δ 8.78 (d, J = 2.4 Hz, 1H), 8.08 - 8.02 (m, 1H), 7.84 (t, J = 5.4 Hz, 1H), 7.62 (dd, J = 5.1, 1.2 Hz, 1H), 7.45 - 7.38 (m, 2H), 7.38 - 7.28 (m, 5H), 7.28 - 7.19 (m, 3H), 7.15 (d, J = 3.5 Hz, 1H), 7.1 - 7.05 (m, 4H), 7.05 - 6.98 (m, 2H), 6.44 (t, J = 8.5 Hz, 1H), 5.06 (s, 2H), 4.44 - 4.28

(m, 3H), 4.24 - 4.08 (m, 3H), 4.02 (bs, 1H), 3.86 – 3.81 (m, 2H), 3.53 - 3.43 (m, 3H), 3.07 (dt, *J* = 12.5, 6.8 Hz, 5H), 3.03 - 2.86 (m, 9H), 2.83 - 2.73 (m, 2H), 2.34 (t, *J* = 8.2 Hz, 2H), 1.97 - 1.85 (m, 2H), 1.41 - 1.28 (m, 5H), 1.27 - 1.13 (m, 6H).

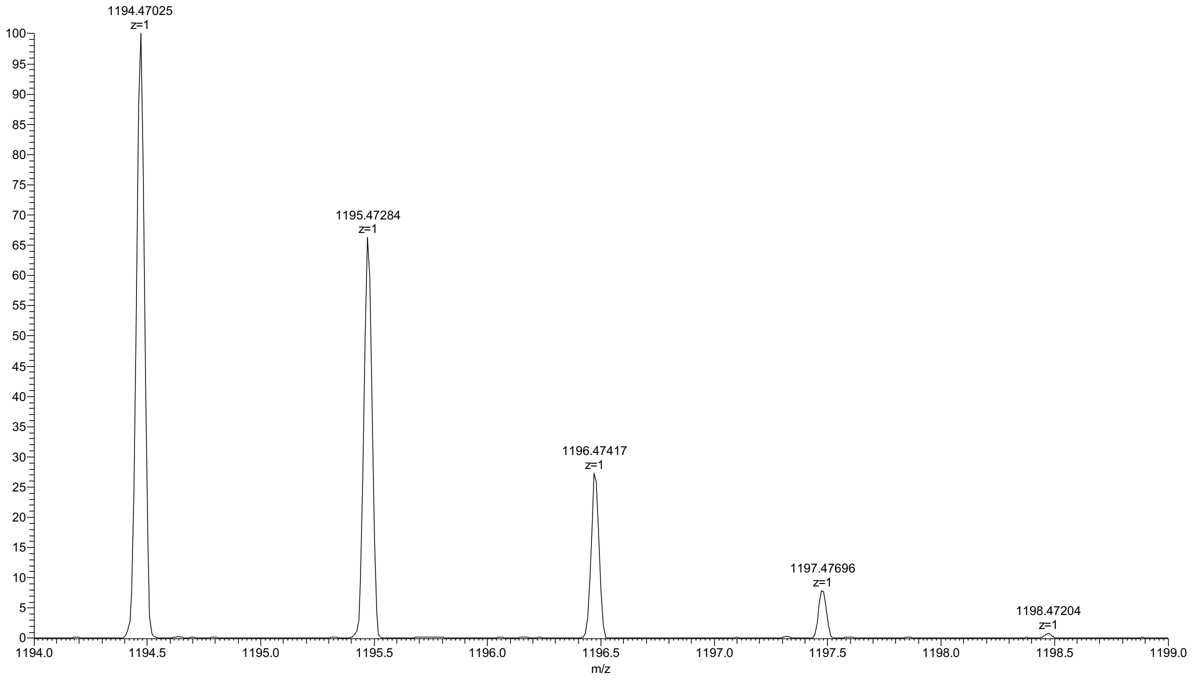

HRMS (ESI, m/z) of compound **15**.

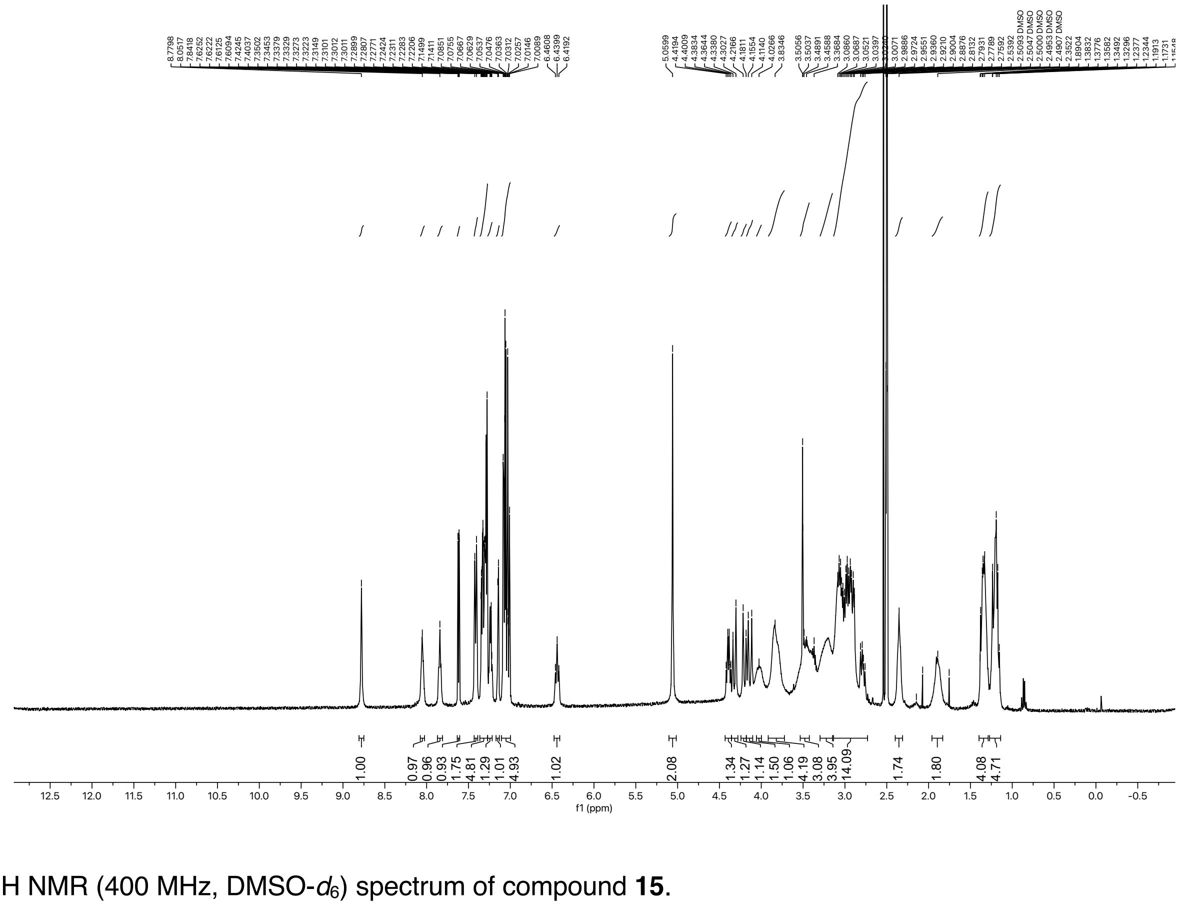

^{1}H NMR (400 MHz, DMSO-d_6) spectrum of compound **15**.

^{1}H NMR (400 MHz, DMSO-d_6) δ 8.79 (d, J = 2.4 Hz, 1H), 8.07 - 8.02 (m, 1H), 7.85 (t, J = 5.4 Hz, 1H), 7.62 (dd, J = 5.1, 1.2 Hz, 1H), 7.41 (d, J = 7.7 Hz, 2H), 7.38 - 7.28 (m, 4H), 7.28 - 7.19 (m, 2H), 7.15 (d, J = 3.5 Hz, 1H), 7.10 - 6.97 (m, 5H), 6.44 (t, J = 8.5 Hz, 1H), 5.06 (s, 2H), 4.39 (q, J = 7.4 Hz, 1H),

4.32 (d, *J* = 14.2 Hz, 1H), 4.20 (d, *J* = 14.1 Hz, 1H), 4.13 (d, *J* = 16.6 Hz, 1H), 4.02 (bs, 1H), 3.90 - 3.73 (m, 4H), 3.53 - 3.43 (m, 3H), 3.30 - 3.14 (m, 4H), 3.12 - 2.72 (m, 14H), 2.34 (t, *J* = 8.2 Hz, 2H), 1.97 - 1.85 (m, 2H), 1.41 - 1.28 (m, 4H), 1.27 - 1.12 (m, 5H).

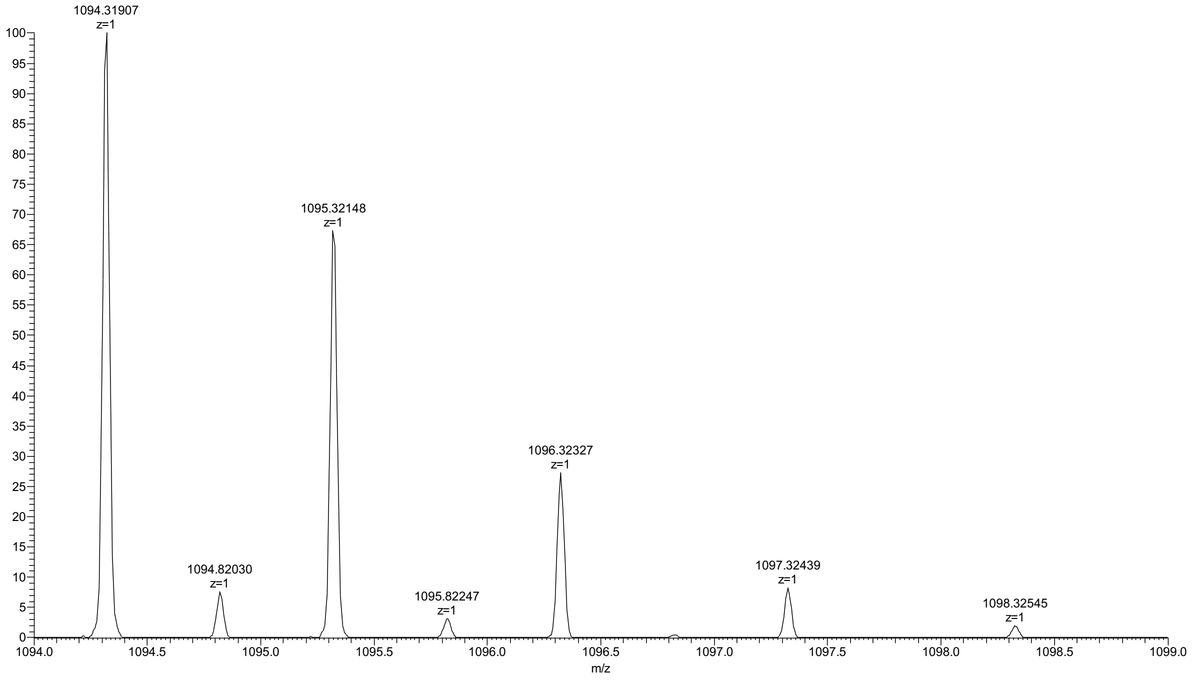

HRMS (ESI, m/z) of compound **16**.

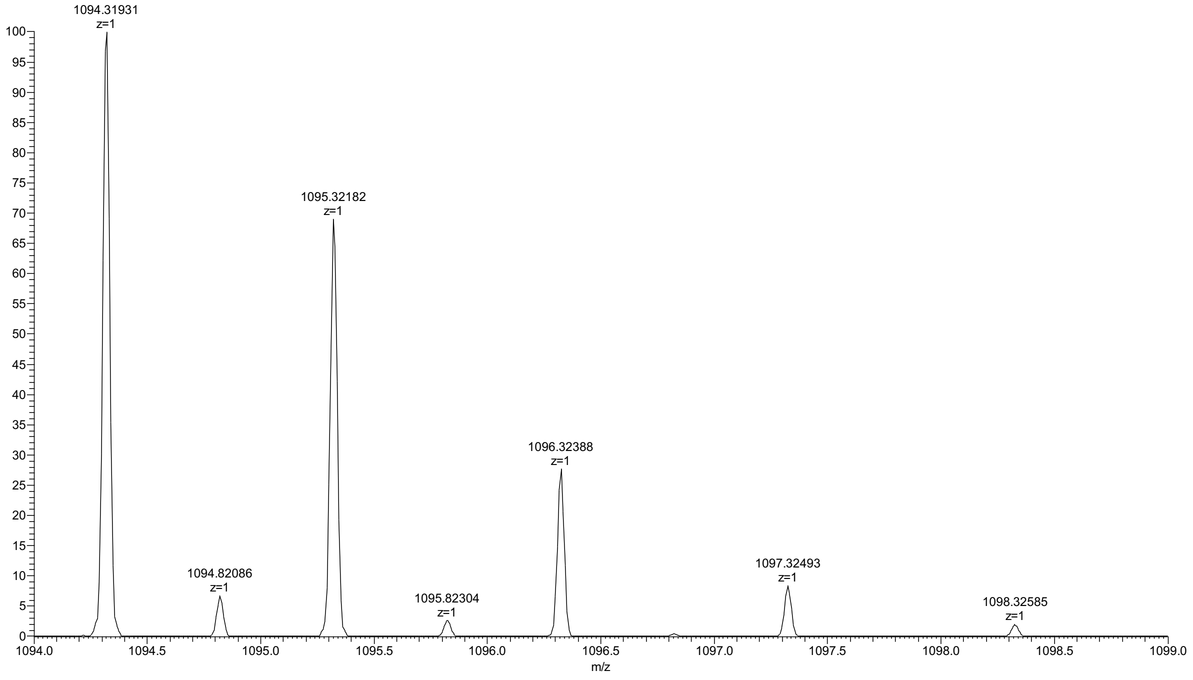

HRMS (ESI, m/z) of compound **17**.

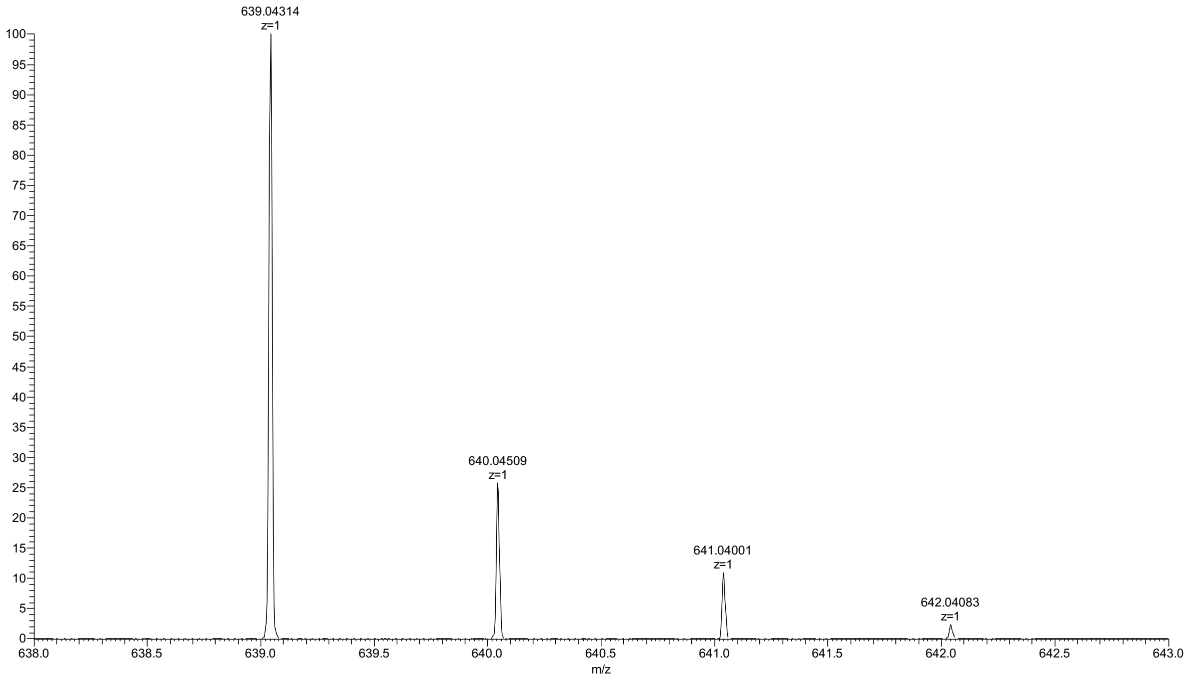

HRMS (ESI, m/z) of compound **s9a**.

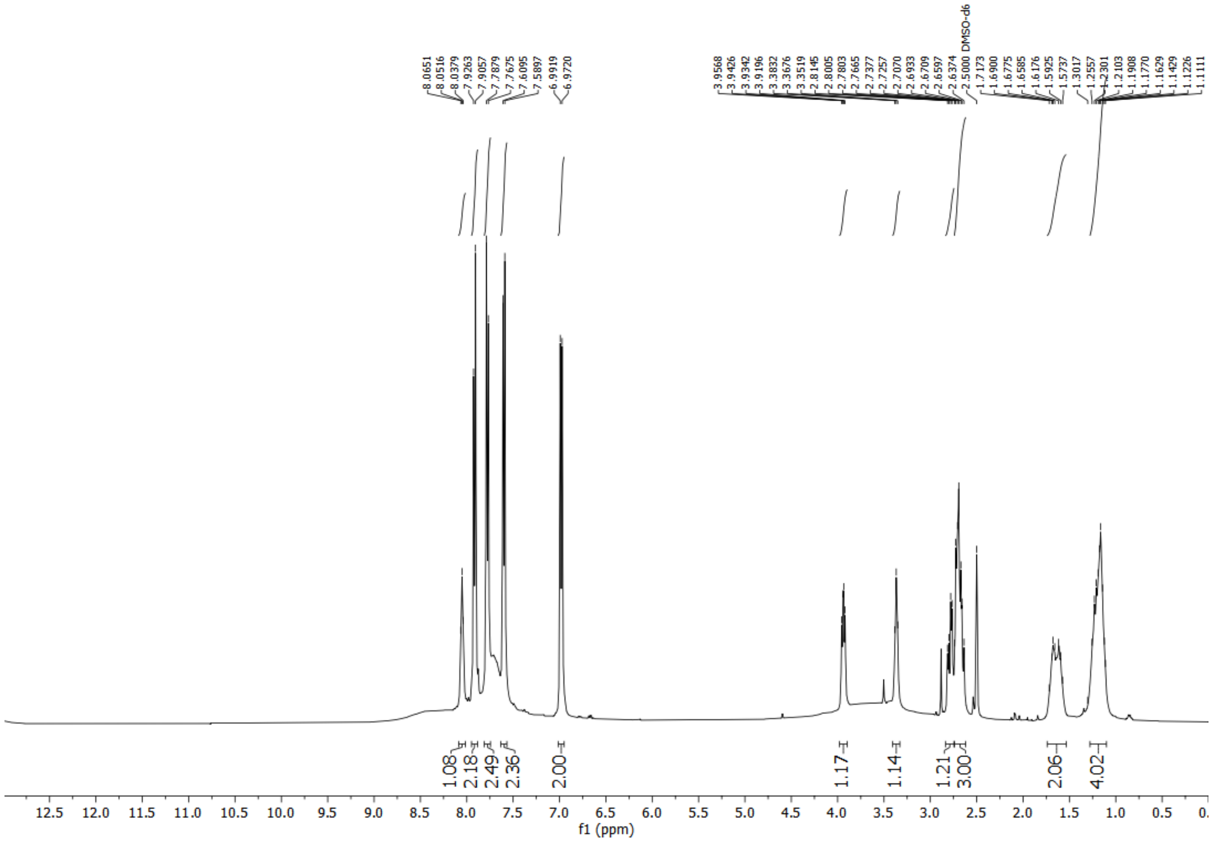

^{1}H NMR (400 MHz, DMSO-d_6) spectrum of compound **s9a**.

^{1}H NMR (400 MHz, DMSO-d_6) δ 8.06 (t, J = 5.4 Hz, 1H), 7.93 (d, J = 8.2 Hz, 2H), 7.77 (d, J = 8.2 Hz, 2H), 7.60 (d, J = 7.9 Hz, 2H), 6.98 (d, J = 7.8 Hz, 2H), 3.94 (dd, J = 9.3, 5.5 Hz, 1H), 3.38 (t, J = 6.3 Hz, 1H), 2.79 (dd, J = 13.5, 5.4 Hz, 1H), 2.745 - 2.62 (m, 3H), 1.73 - 1.54 (m, 2H), 1.28 - 1.10 (m, 4H).

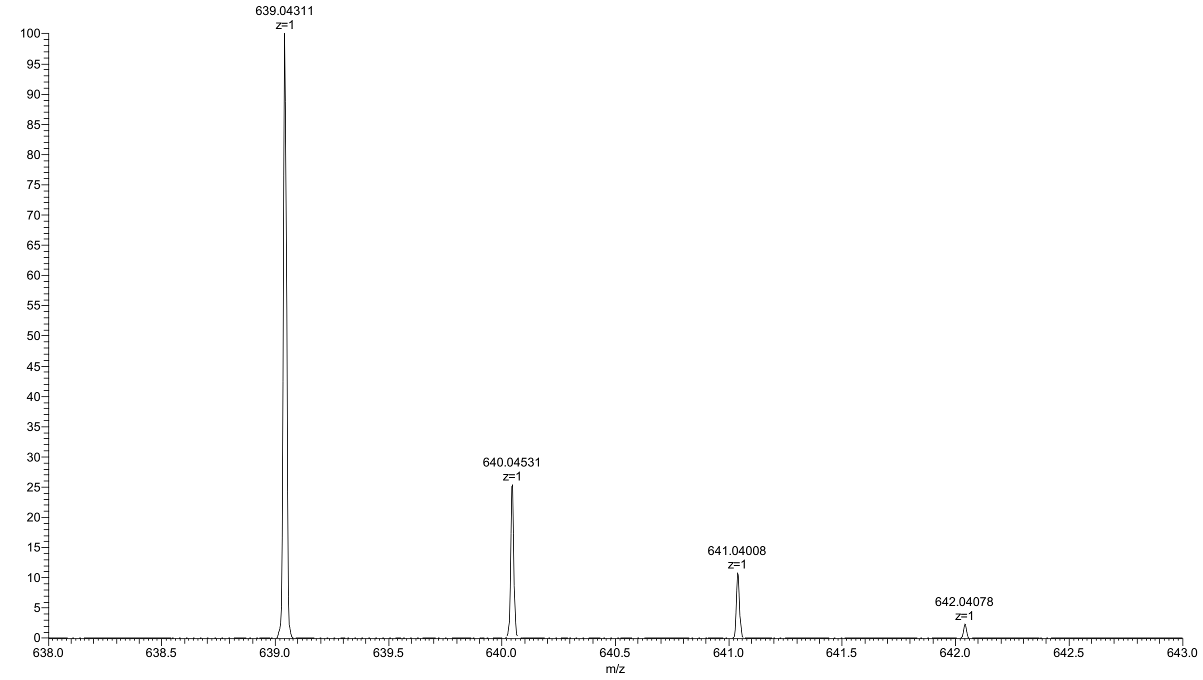

HRMS (ESI, m/z) of compound **s9b**.

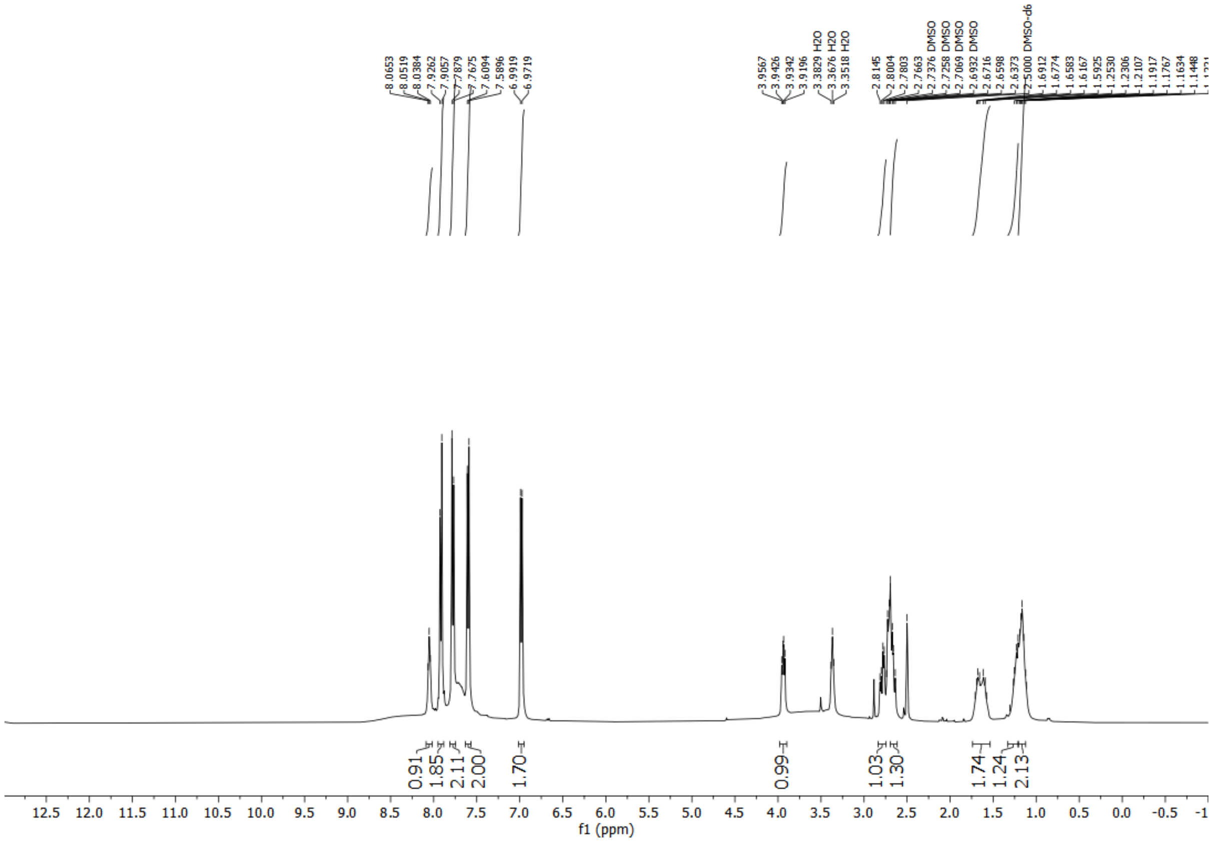

^{1}H NMR (400 MHz, DMSO-d_6) spectrum of compound **s9b**.

^{1}H NMR (400 MHz, DMSO-d_6) δ 8.05 (t, J = 5.4 Hz, 1H), 7.92 (d, J = 8.2 Hz, 2H), 7.78 (d, J = 8.2 Hz, 2H), 7.60 (d, J = 7.9 Hz, 2H), 6.98 (d, J = 8.0 Hz, 2H), 3.94 (dd, J = 9.1, 5.8 Hz, 1H), 3.38 (t, J = 6.1 Hz, 1H), 2.79 (dd, J = 13.7, 5.6 Hz, 1H), 2.74 - 2.64 (m, 3H), 1.74 - 1.53 (m, 2H), 1.33 - 1.12 (m, 4H).

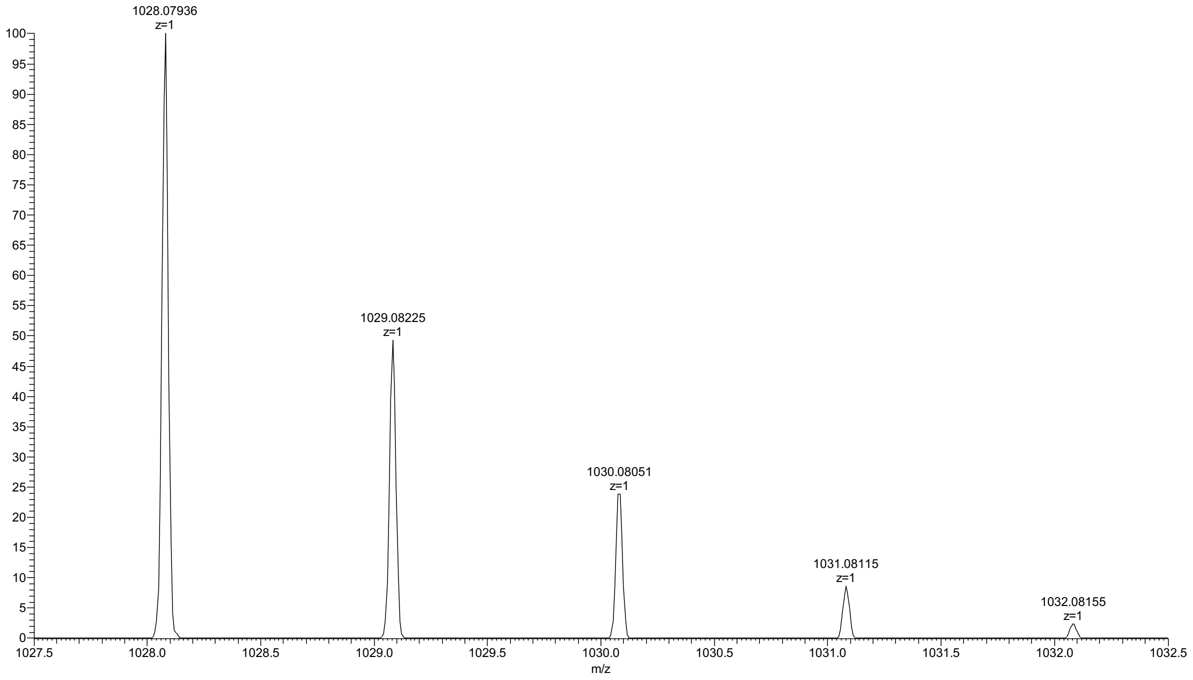

HRMS (ESI, m/z) of compound **18**.

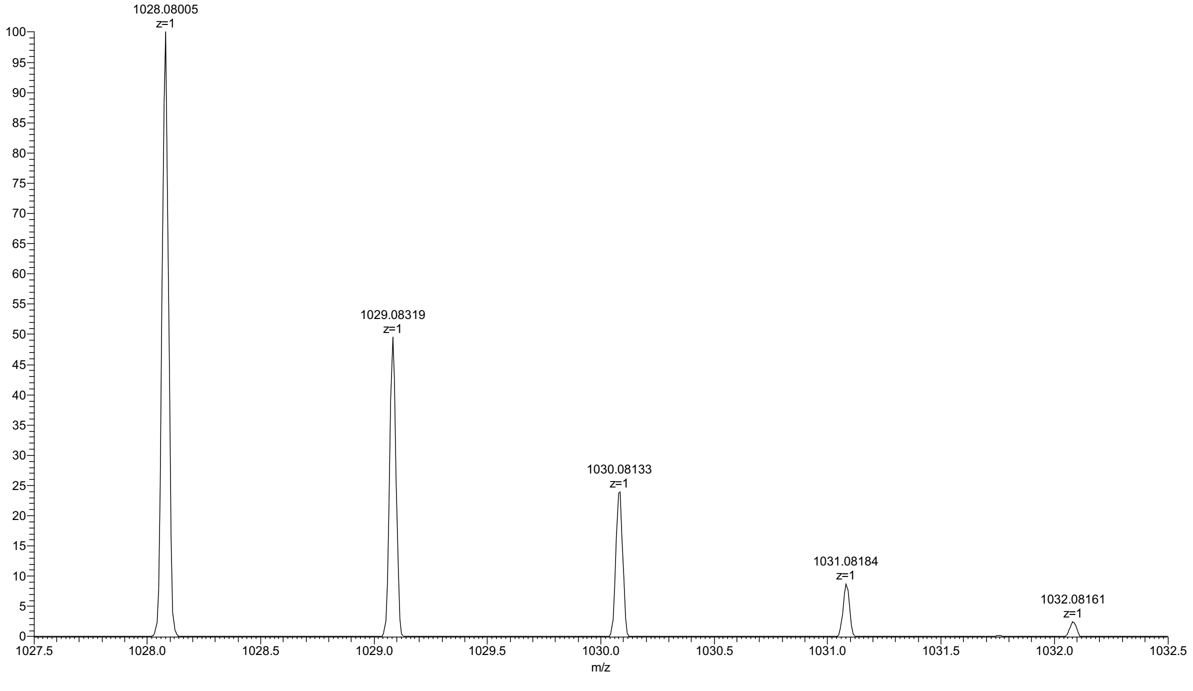

HRMS (ESI, m/z) of compound **19**.

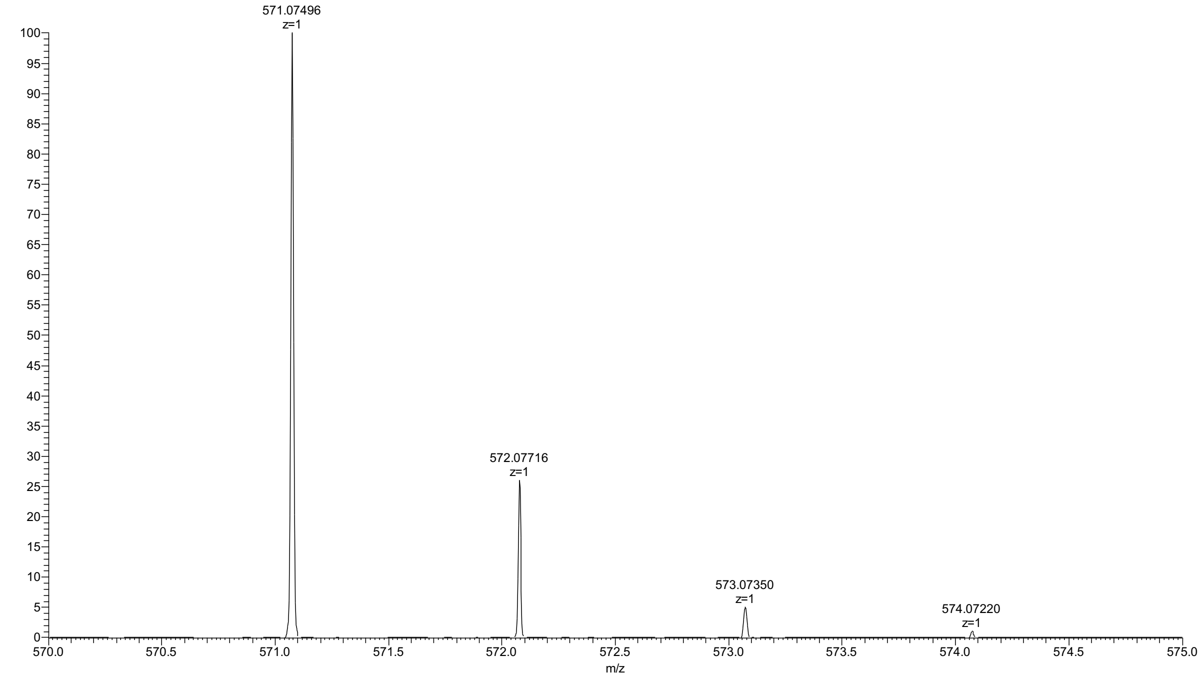

HRMS (ESI, m/z) of compound **s11a**.

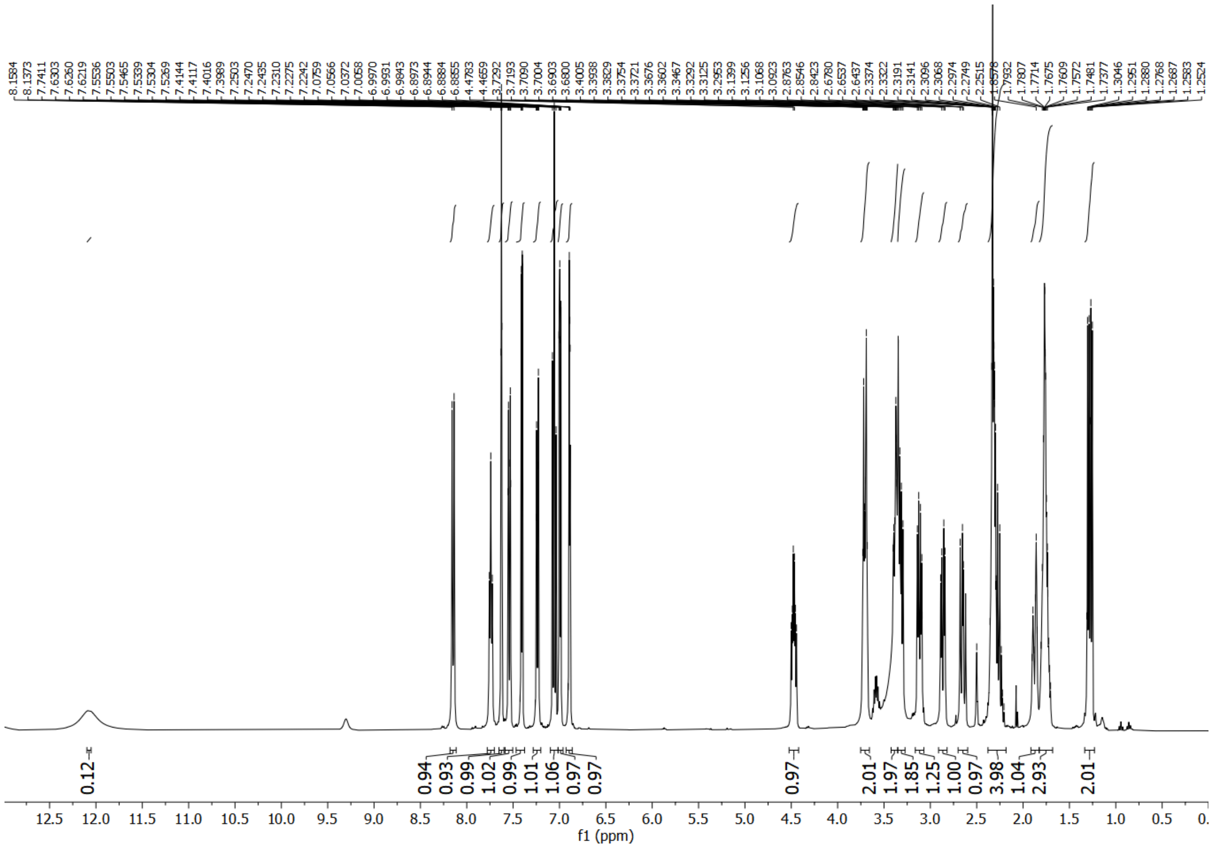

^{1}H NMR (400 MHz, DMSO-d_6) spectrum of compound **s11a**.

^{1}H NMR (400 MHz, DMSO-d_6) δ 8.28 (d, J = 8.4 Hz, 1H), 7.88 (t, J = 6.3 Hz, 1H), 7.76 (t, J = 1.7 Hz, 1H), 7.68 (dt, J = 7.8, 1.4 Hz, 1H), 7.54 (dd, J = 5.1, 1.1 Hz, 1H), 7.37 (dt, J = 7.7, 1.3 Hz, 1H), 7.19 (t, J = 7.7 Hz, 1H), 7.13 (dd, J = 5.1, 3.5 Hz, 1H), 7.03 (dd, J = 3.6, 1.2 Hz, 1H), 4.61 (td, J = 9.1, 5.0 Hz, 1H), 3.84 (dt, J = 11.6, 4.0 Hz, 2H), 3.56 - 3.49 (m, 2H), 3.46 (dd, J = 13.6, 6.9 Hz, 2H), 3.25 (dd, J =

13.3, 5.8 Hz, 1H), 3.00 (dd, *J* = 13.7, 5.0 Hz, 1H), 2.78 (dd, *J* = 13.6, 9.7 Hz, 1H), 2.51 - 2.32 (m, 4H), 2.05 - 1.96 (m, 1H), 1.89 (ddt, *J* = 13.9, 6.1, 3.9 Hz, 3H), 1.42 (ddd, *J* = 14.3, 6.8, 2.8 Hz, 2H).

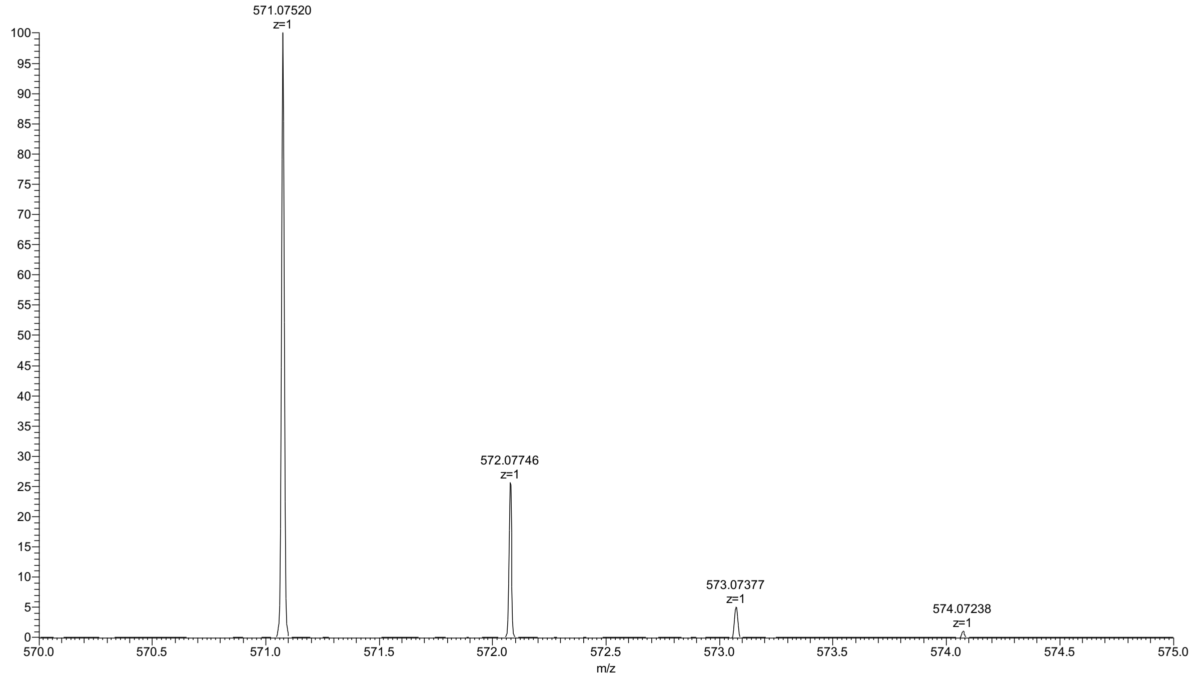

HRMS (ESI, m/z) of compound **s11b**.

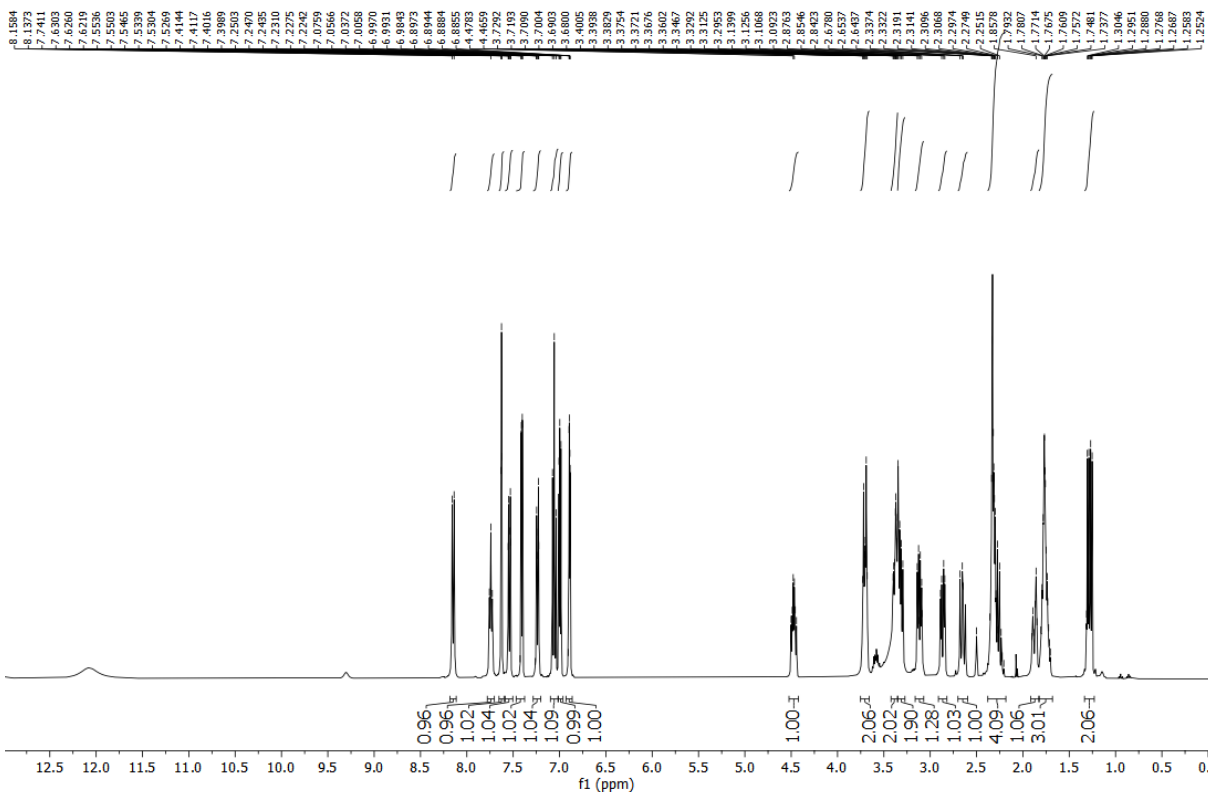

^{1}H NMR (400 MHz, DMSO-d_6) spectrum of compound **s11b**.

^{1}H NMR (400 MHz, DMSO-d_6) δ 8.30 (d, J = 8.4 Hz, 1H), 7.90 (t, J = 6.3 Hz, 1H), 7.78 (t, J = 1.7 Hz, 1H), 7.70 (dt, J = 7.9, 1.4 Hz, 1H), 7.56 (dd, J = 5.1, 1.1 Hz, 1H), 7.39 (dt, J = 7.7, 1.3 Hz, 1H), 7.21 (t, J = 7.7 Hz, 1H), 7.15 (dd, J = 5.1, 3.5 Hz, 1H), 7.05 (dd, J = 3.6, 1.2 Hz, 1H), 4.63 (td, J = 9.1, 5.0 Hz, 1H), 3.86 (dt, J = 11.6, 4.0 Hz, 2H), 3.58 - 3.43 (m, 4H), 3.27 (dd, J = 13.3, 5.8 Hz, 1H), 3.02 (dd, J =

13.7, 5.0 Hz, 1H), 2.81 (dd, *J* = 13.7, 9.7 Hz, 1H), 2.56 - 2.34 (m, 4H), 2.07 - 1.98 (m, 1H), 1.98 - 1.84 (m, 3H), 1.44 (ddd, *J* = 14.2, 6.8, 2.8 Hz, 2H).

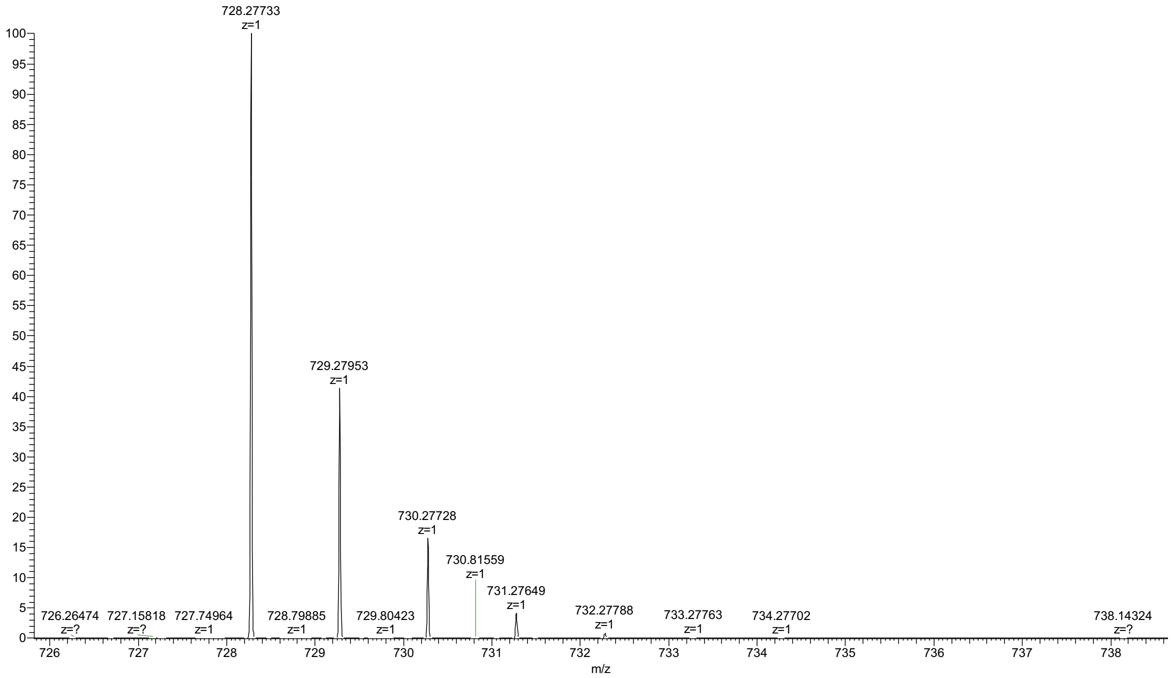

HRMS (ESI, m/z) of compound **s12a**.

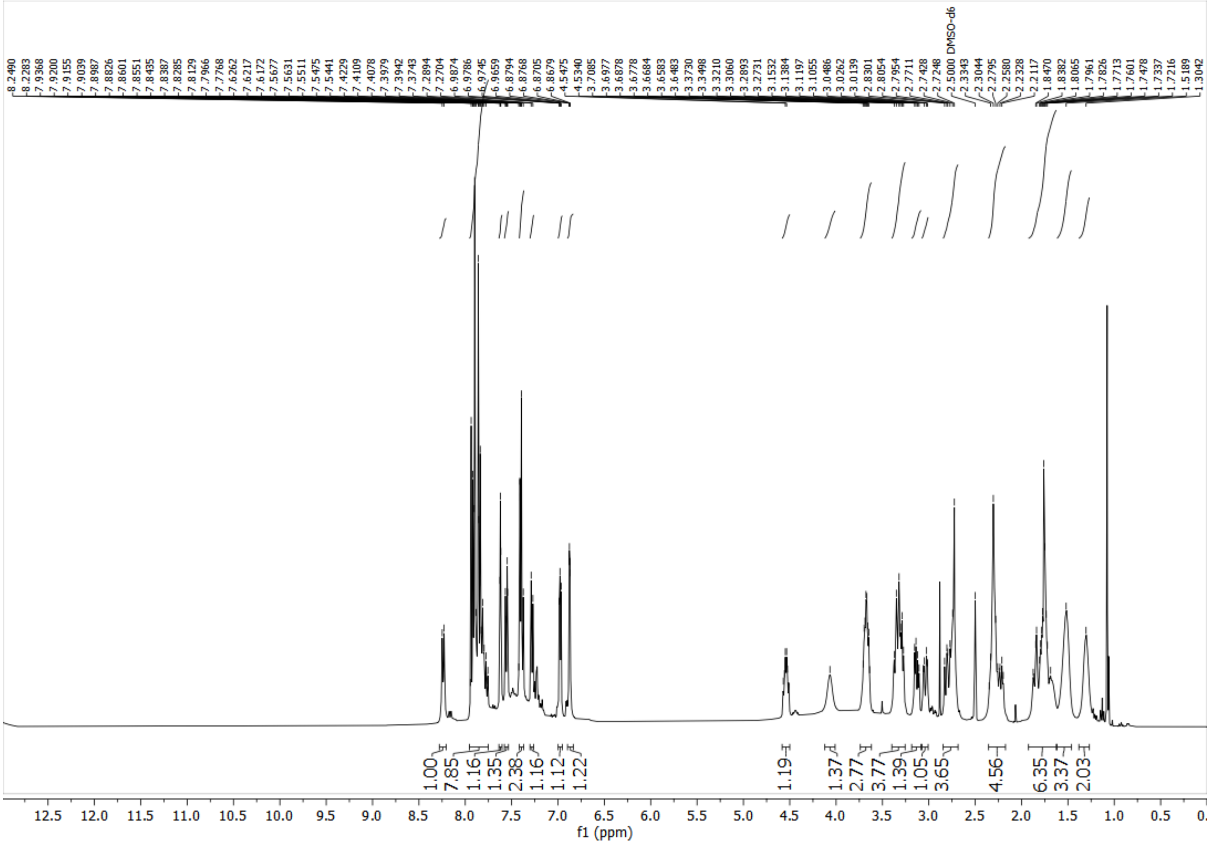

^{1}H NMR (400 MHz, DMSO-d_6) spectrum of compound **s12a**.

^{1}H NMR (400 MHz, DMSO-d_6) 8.24 (d, J = 8.3 Hz, 1H), 7.95 - 7.75 (m, 8H), 7.62 (bs, 1H), 7.58 - 7.53 (m, 1H), 7.42 - 7.37 (m, 2H), 7.28 (d, J = 7.6 Hz, 1H), 6.98 (dd, J = 5.1, 3.5 Hz, 1H), 6.90 - 6.84 (m, 1H), 4.55 (m, 1H), 4.07 (s, 1H), 3.67 (m, 3H), 3.40 - 3.26 (m, 4H), 3.13 (dd, J = 13.3, 5.8 Hz, 1H), 3.04

(dd, J = 13.9, 4.9 Hz, 1H), 2.85 - 2.68 (m, 4H), 2.36 - 2.17 (m, 5H), 1.93 - 1.63 (m, 6H), 1.52 (sm 3H), 1.30 (m, 2H).

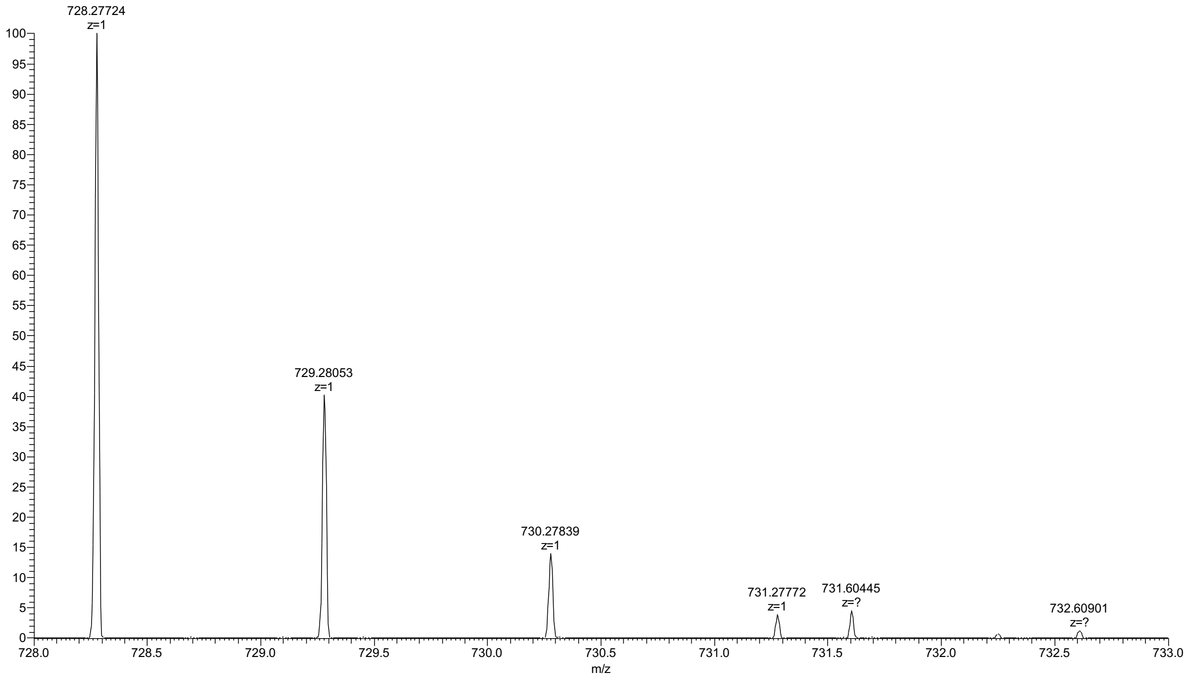

HRMS (ESI, m/z) of compound **s12b**.

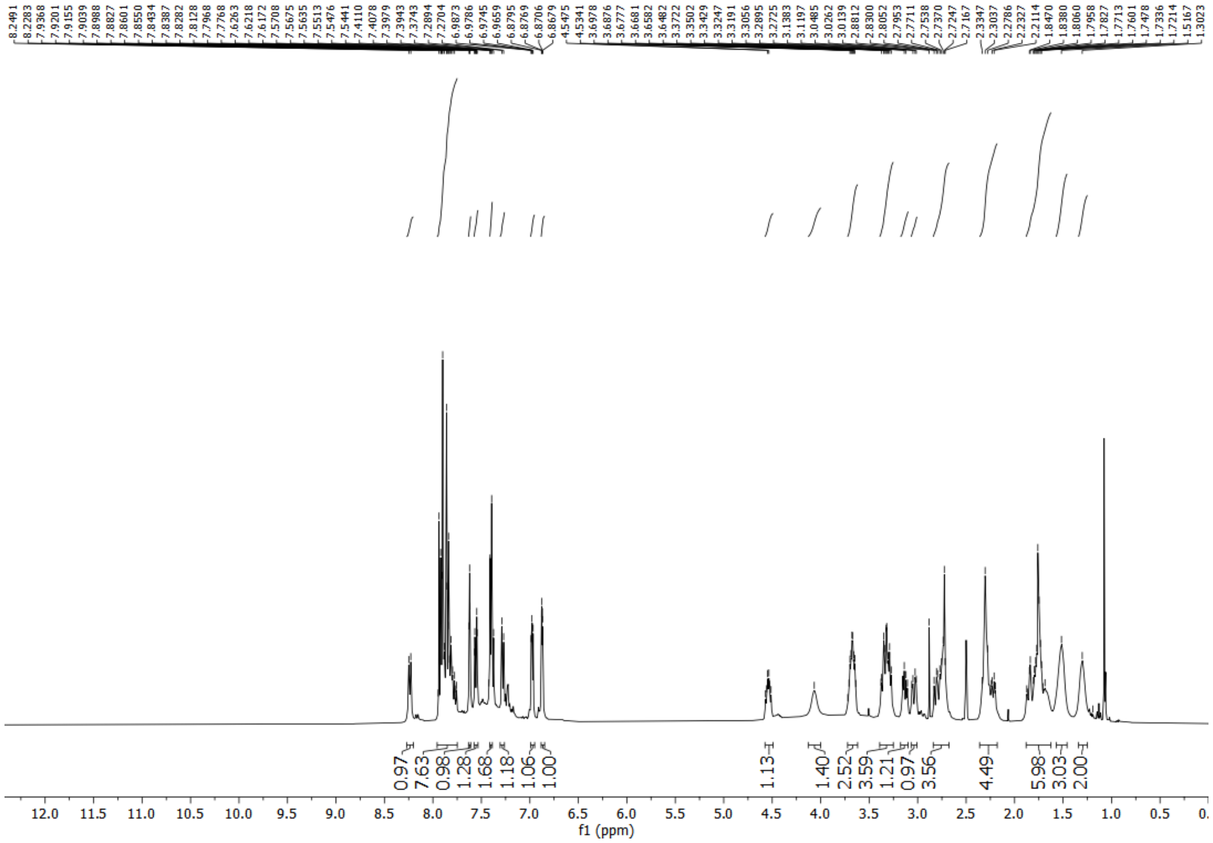

^1H NMR (400 MHz, DMSO-d_6) spectrum of compound **s12b**.

^{1}H NMR (400 MHz, DMSO-d_6) δ 8.24 (d, *J* = 8.3 Hz, 1H), 7.97 - 7.74 (m, 8H), 7.62 (bs, 1H), 7.56 (dt, *J* = 7.8, 1.4 Hz, 1H), 7.41 - 7.39 (m, 2H), 7.28 (d, *J* = 7.6 Hz, 1H), 6.98 (dd, *J* = 5.1, 3.5 Hz, 1H), 6.88 - 6.85 (m, 1H), 4.54 (m, 1H), 4.07 (s, 1H), 3.68 (m, 3H), 3.39 - 3.25 (m, 4H), 3.18 - 3.10 (m, 1H), 3.04

(dd, J = 13.8, 4.9 Hz, 1H), 2.84 - 2.68 (m, 4H), 2.36 - 2.18 (m, 5H), 1.88 - 1.62 (m, 6H), 1.52 (m, 3H), 1.30 (m, 2H).

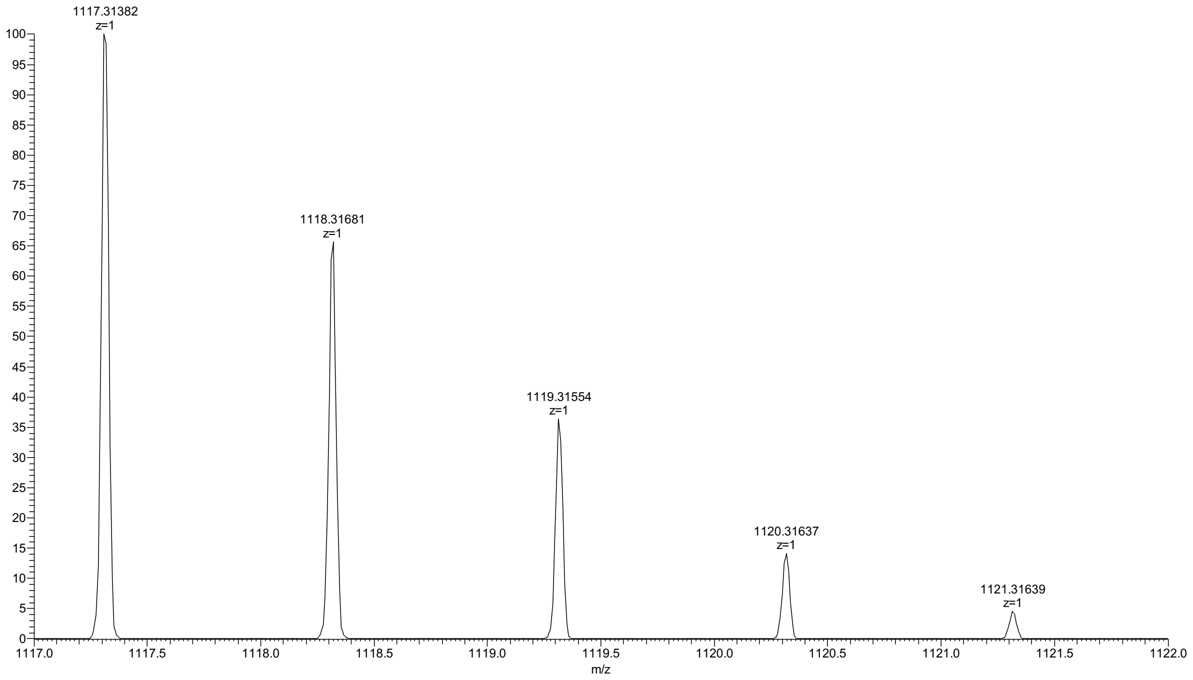

HRMS (ESI, m/z) of compound **20**.

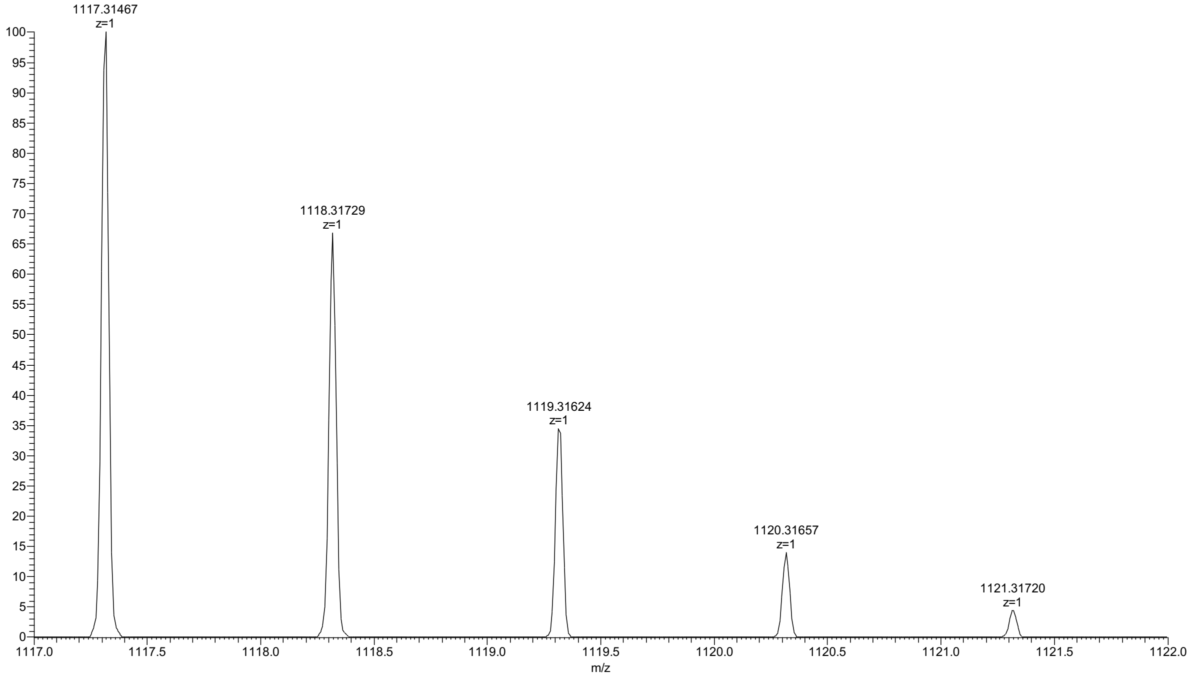

HRMS (ESI, m/z) of compound **21**.

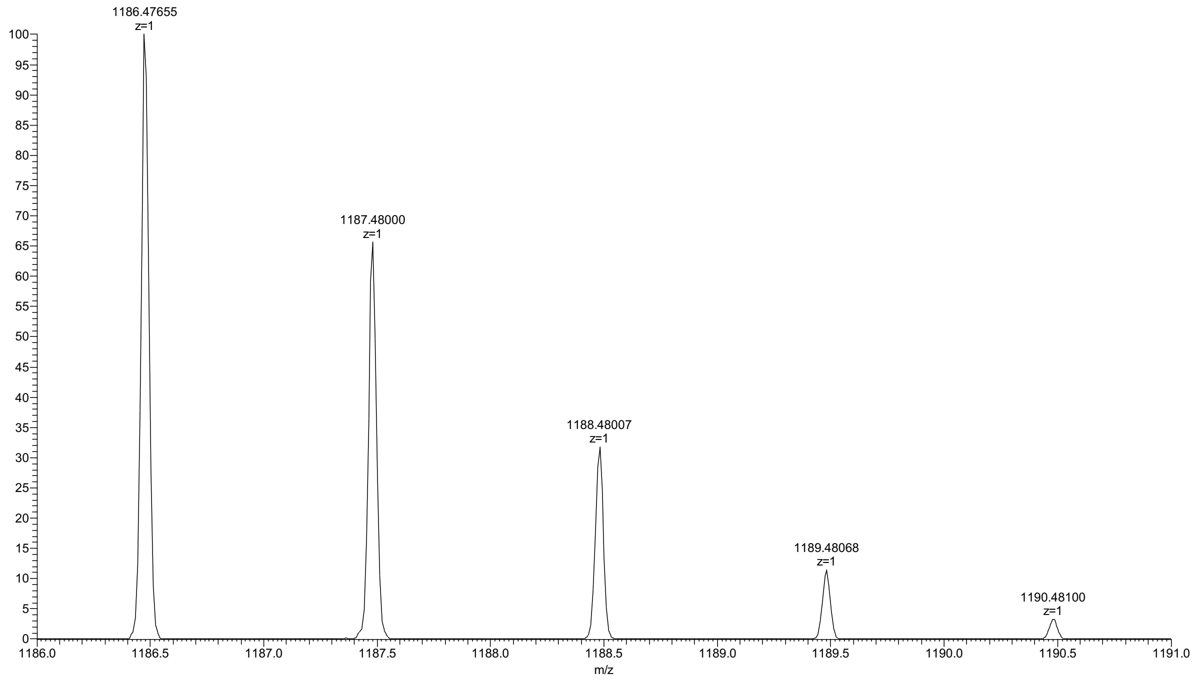

HRMS (ESI, m/z) of compound **22**.

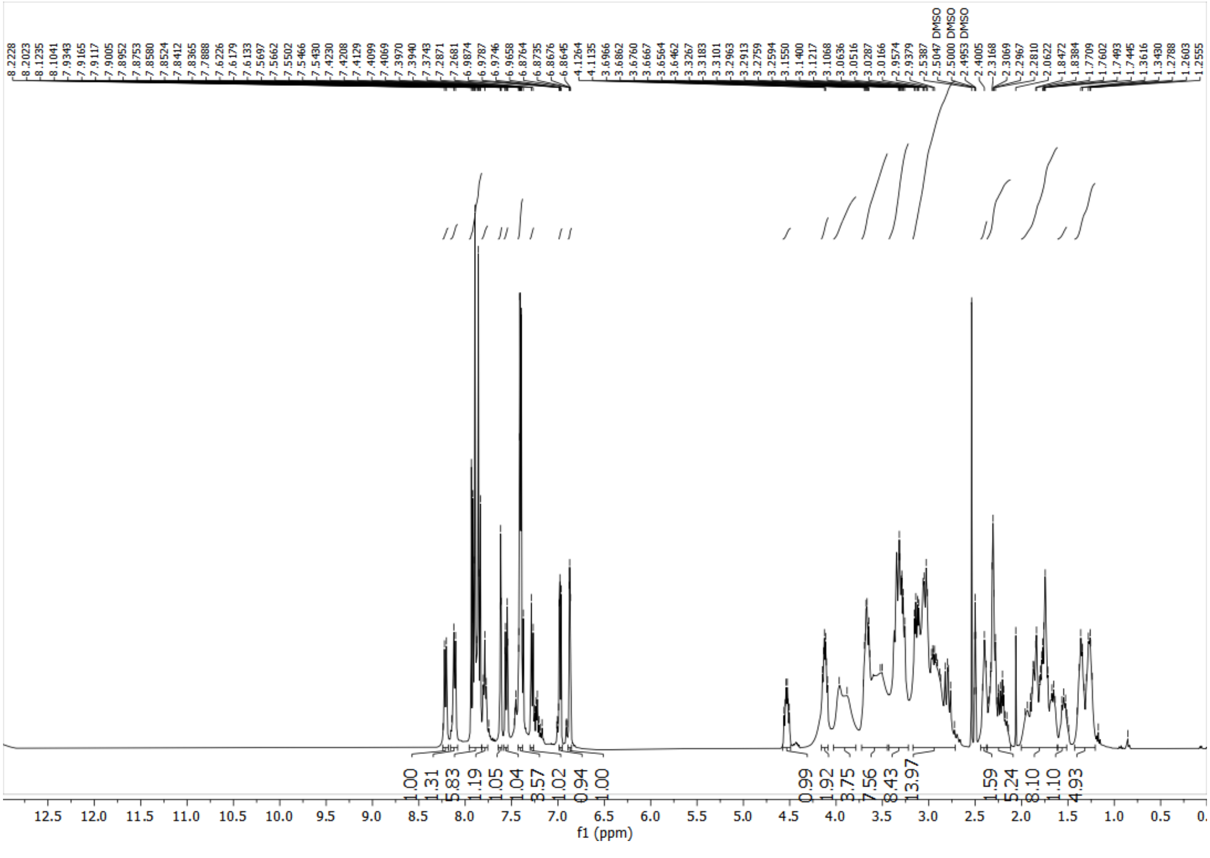

^{1}H NMR (400 MHz, DMSO-d_6) spectrum of compound **22**.

^{1}H NMR (400 MHz, DMSO-d_6) δ 8.21 (d, J = 8.2 Hz, 1H), 8.11 (d, J = 7.8 Hz, 1H), 7.96 - 7.71 (m, 6H), 7.62 (t, J = 1.9 Hz, 1H), 7.56 (dt, J = 7.7, 1.3 Hz, 1H), 7.49 - 7.35 (m, 4H), 7.31 - 7.25 (m, 1H), 7.03 - 6.94 (m, 1H), 6.87 (dd, J = 3.6, 1.2 Hz, 1H), 4.53 (td, J = 8.8, 5.0 Hz, 1H), 4.12 (m, 2H), 3.96 – 3.76 (m, 4H), 3.71 – 3.41 (m, H), 3.40 – 3.22 (m, 8H), 3.17 – 2.73 (m, 14H), 2.44 – 2.31 (m, 3H), 2.31 (s, 1H),

2.30 - 2.12 (m, 3H), 1.98 - 1.91 (m, 1H), 1.91 - 1.62 (m, 6H), 1.61 - 1.47 (m, 1H), 1.36 (t, J = 7.7 Hz, 2H), 1.32 - 1.20 (m, 2H).

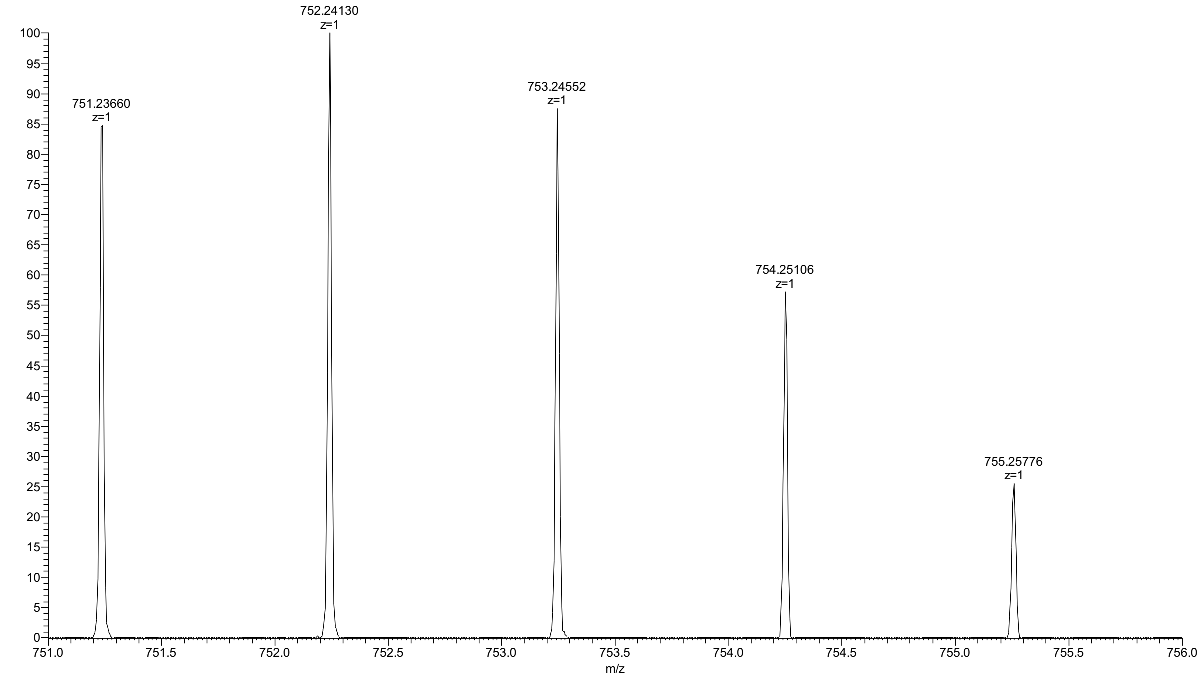

HRMS (ESI, m/z) of compound **s13a**.

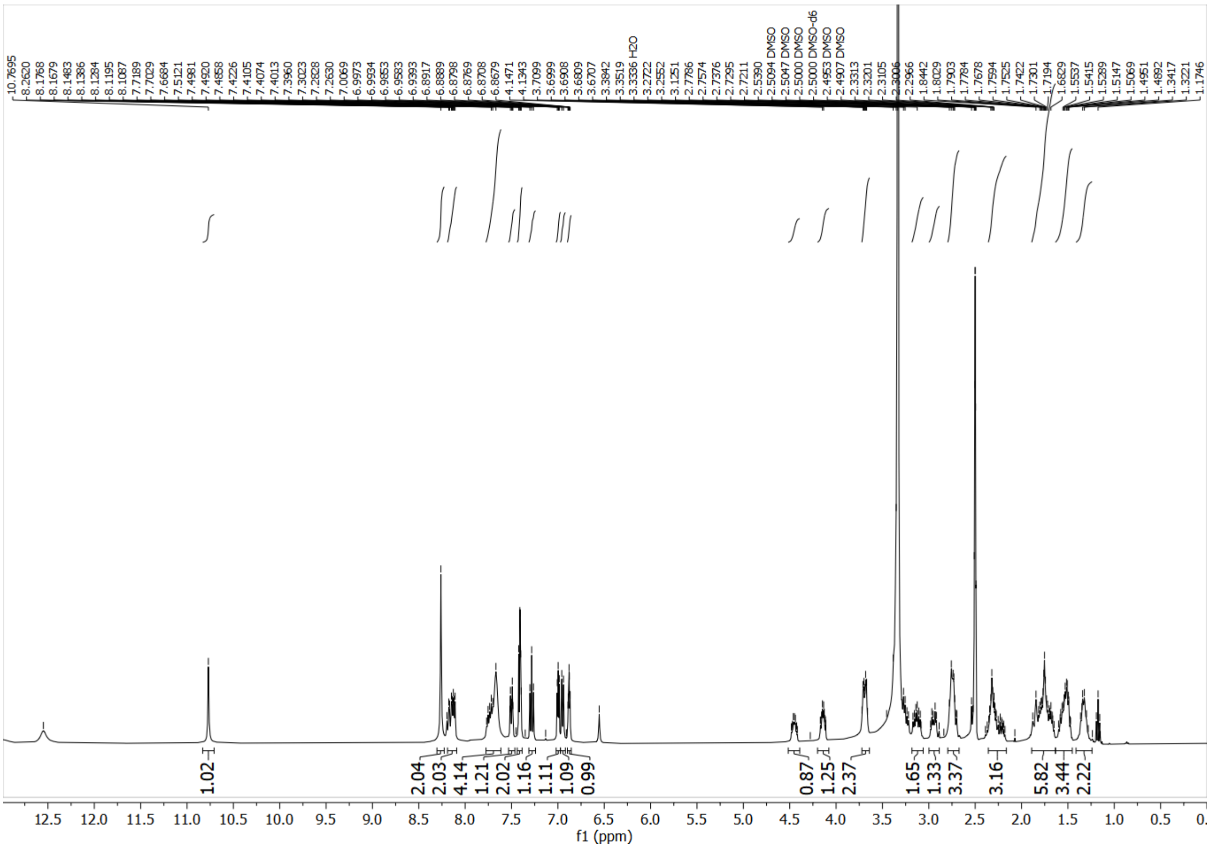

^{1}H NMR (400 MHz, DMSO-d_6) spectrum of compound **s13a**.

^{1}H NMR (400 MHz, DMSO-d_6) δ 10.77 (s, 1H), 8.26 (s, 2H), 8.22 - 8.09 (m, 2H), 7.78 - 7.61 (m, 4H), 7.51 (dd, J = 8.1, 2.5 Hz, 1H), 7.45 - 7.37 (m, 2H), 7.28 (t, J = 7.9 Hz, 1H), 7.00 (dd, J = 5.1, 3.5 Hz, 1H), 6.95 (d, J = 7.6 Hz, 1H), 6.88 (td, J = 4.2, 3.6, 1.1 Hz, 1H), 4.45 (tt, J = 8.4, 2.8 Hz, 1H), 4.19 - 4.09

(m, 1H), 3.74 - 3.63 (m, 3H), 3.13 (td, J = 13.2, 12.7, 6.1 Hz, 2H), 2.99 - 2.90 (m, 1H), 2.80 - 2.67 (m, 4H), 2.41 - 2.16 (m, 4H), 1.86 - 1.63 (m, 7H), 1.62 - 1.45 (m, 4H), 1.39 - 1.25 (m, 2H).

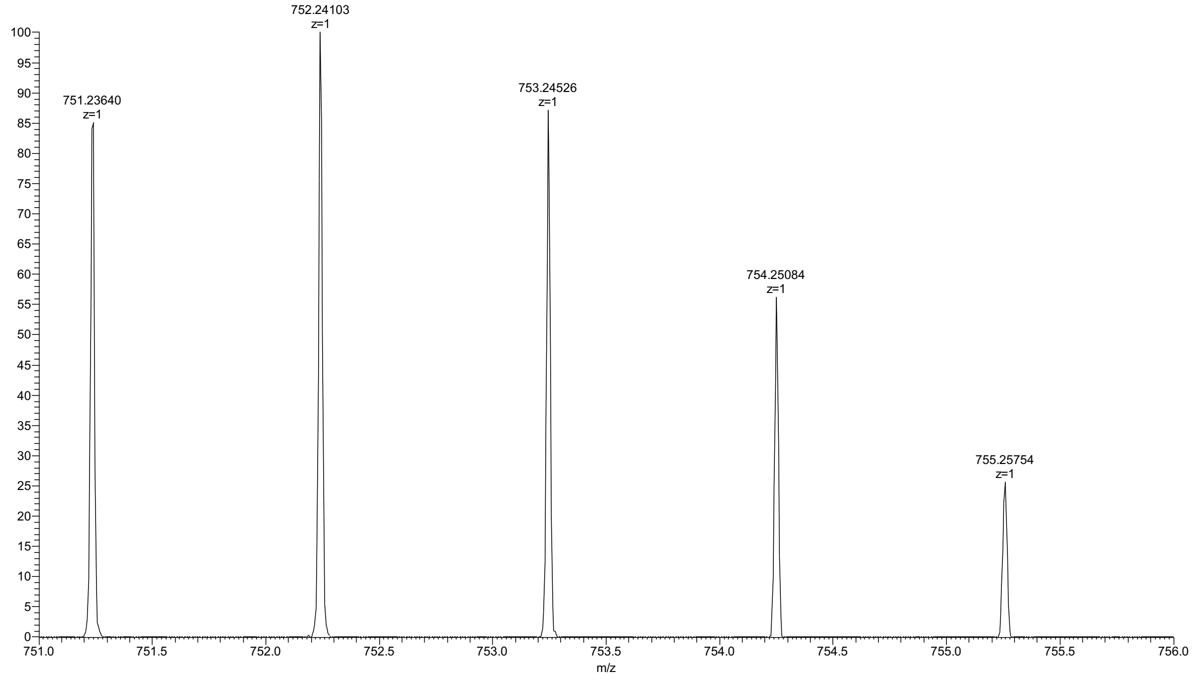

HRMS (ESI, m/z) of compound **s13b**.

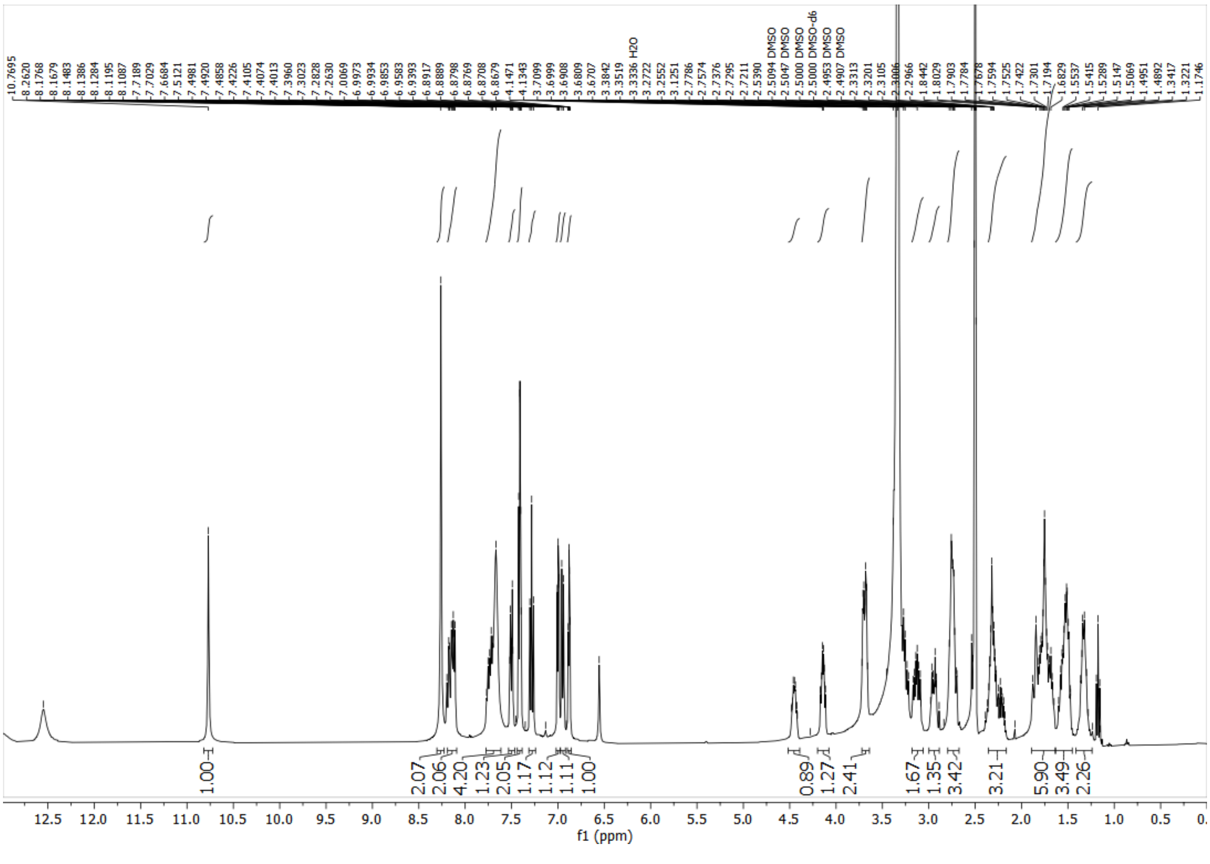

^{1}H NMR (400 MHz, DMSO-d_6) spectrum of compound **s13b**.

^{1}H NMR (400 MHz, DMSO-d_6) δ 10.76 (s, 1H), 8.25 (s, 2H), 8.23 - 8.08 (m, 2H), 7.76 - 7.58 (m, 4H), 7.51 (dd, *J* = 8.1, 2.5 Hz, 1H), 7.47 - 7.39 (m, 2H), 7.29 (t, *J* = 7.9 Hz, 1H), 7.00 (dd, *J* = 5.1, 3.5 Hz, 1H), 6.96 (d, *J* = 7.6 Hz, 1H), 6.84 (td, *J* = 4.2, 3.6, 1.1 Hz, 1H), 4.45 (tt, *J* = 8.8, 2.6 Hz, 1H), 4.19 - 4.09

(m, 1H), 3.74 - 3.63 (m, 3H), 3.13 (td, *J* = 13.5, 12.2, 6.3 Hz, 2H), 2.99 - 2.90 (m, 1H), 2.80 - 2.67 (m, 4H), 2.41 - 2.16 (m, 4H), 1.86 - 1.63 (m, 7H), 1.62 - 1.45 (m, 4H), 1.39 - 1.25 (m, 2H).

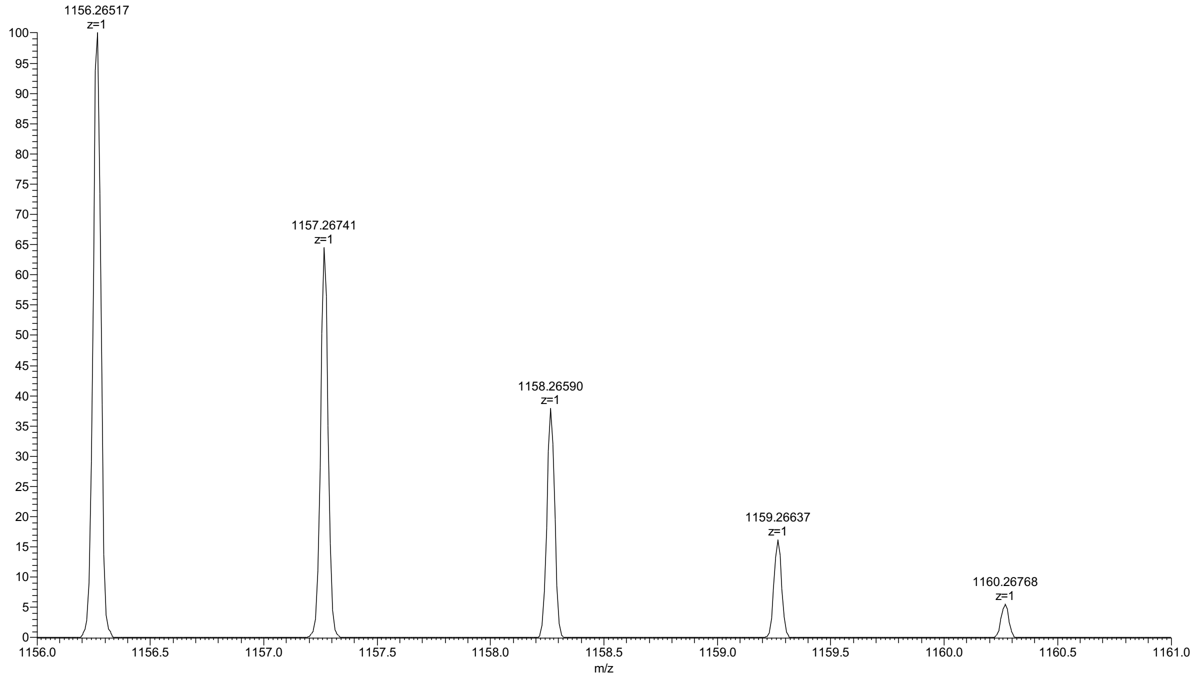

HRMS (ESI, m/z) of compound **23**.

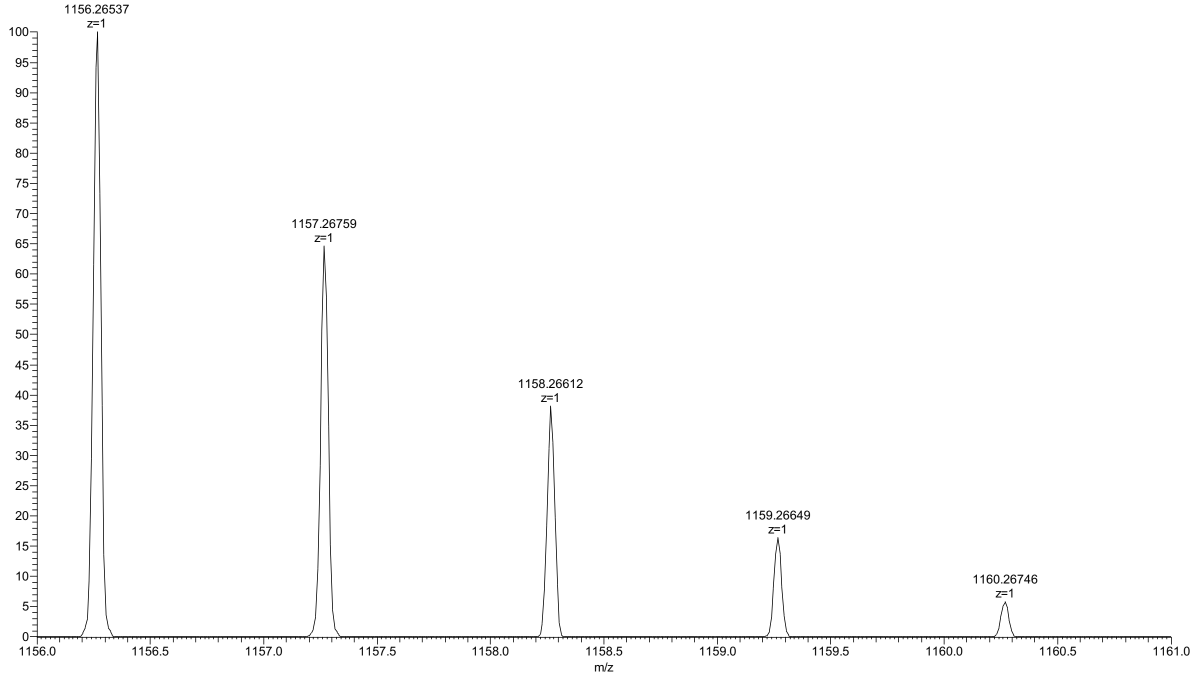

HRMS (ESI, m/z) of compound **24**.

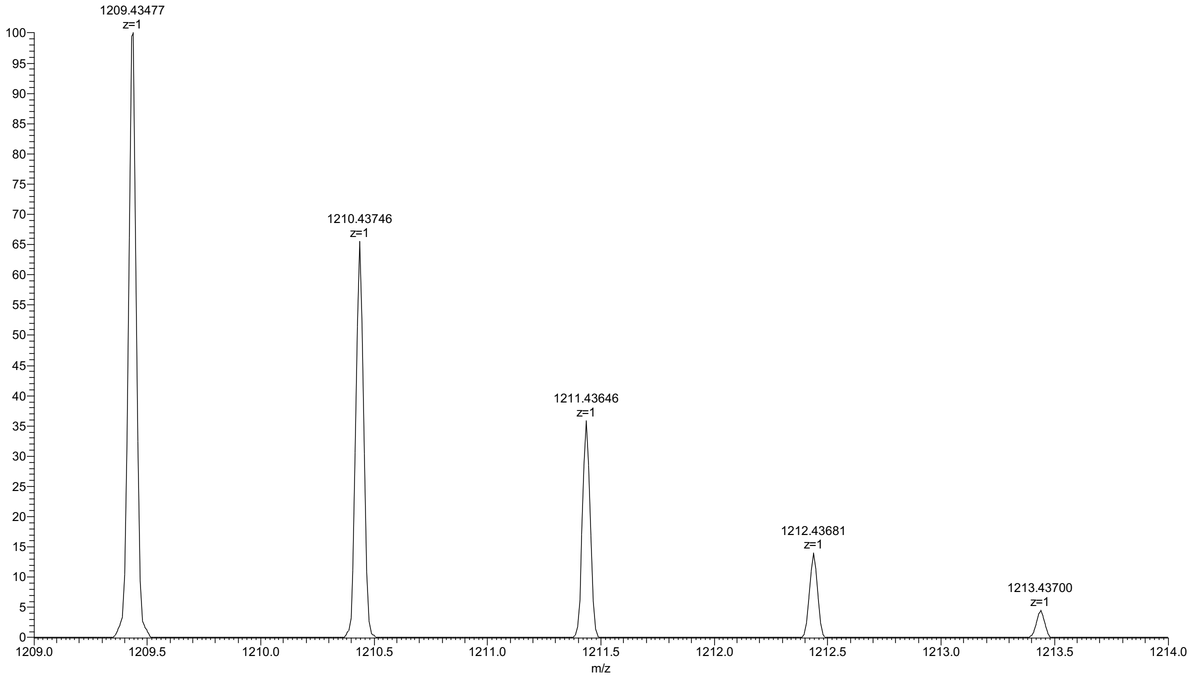

HRMS (ESI, m/z) of compound **25**.

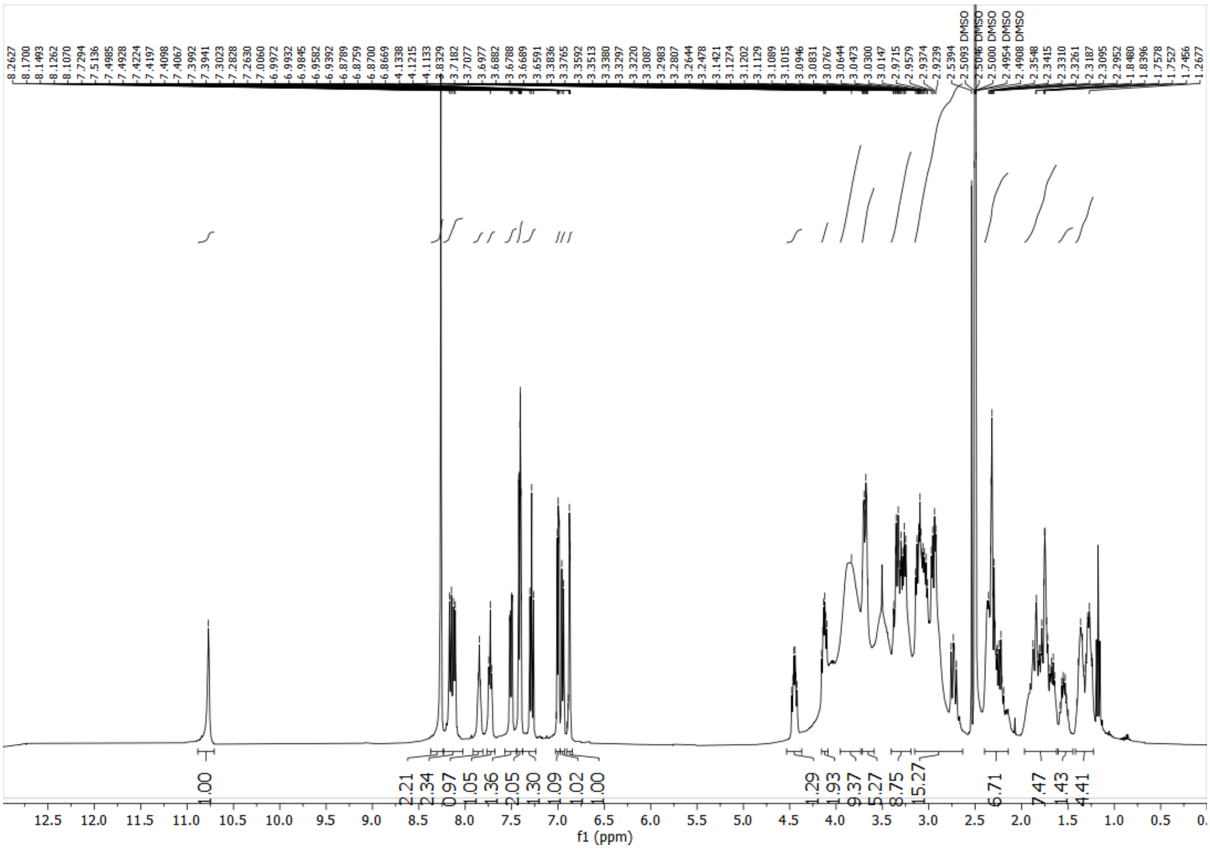

^{1}H NMR (400 MHz, DMSO-d_6) spectrum of compound **25**.

^{1}H NMR (400 MHz, DMSO-d_6) δ 10.77 (s, 1H), 8.26 (s, 2H), 8.14 (dd, J = 17.2, 8.0 Hz, 2H), 7.85 (t, J = 5.6 Hz, 1H), 7.73 (t, J = 6.4 Hz, 1H), 7.51 (dd, J = 8.3, 2.2 Hz, 1H), 7.44 - 7.37 (m, 2H), 7.28 (t, J = 7.8 Hz, 1H), 7.00 (dd, J = 5.1, 3.5 Hz, 1H), 6.97 - 6.93 (m, 1H), 6.87 (dd, J = 3.6, 1.2 Hz, 1H), 4.45 (td, J = 8.8, 5.2 Hz, 1H), 4.13 (td, J = 8.4, 5.4 Hz, 2H), 3.95 - 3.74 (m, 9H) 3.72 - 3.64 (m, 3H), 3.41 - 3.20

(m, 9H), 3.15 - 2.64 (m, 15H), 2.40 - 2.15 (m, 7H), 2.34 - 2.30 (m, 2H), 2.30 - 2.13 (m, 2H), 1.97 - 1.58 (m, 7H), 1.61 - 1.44 (h, J = 8.0 Hz, 1H), 1.41 - 1.22 (m, 4H).

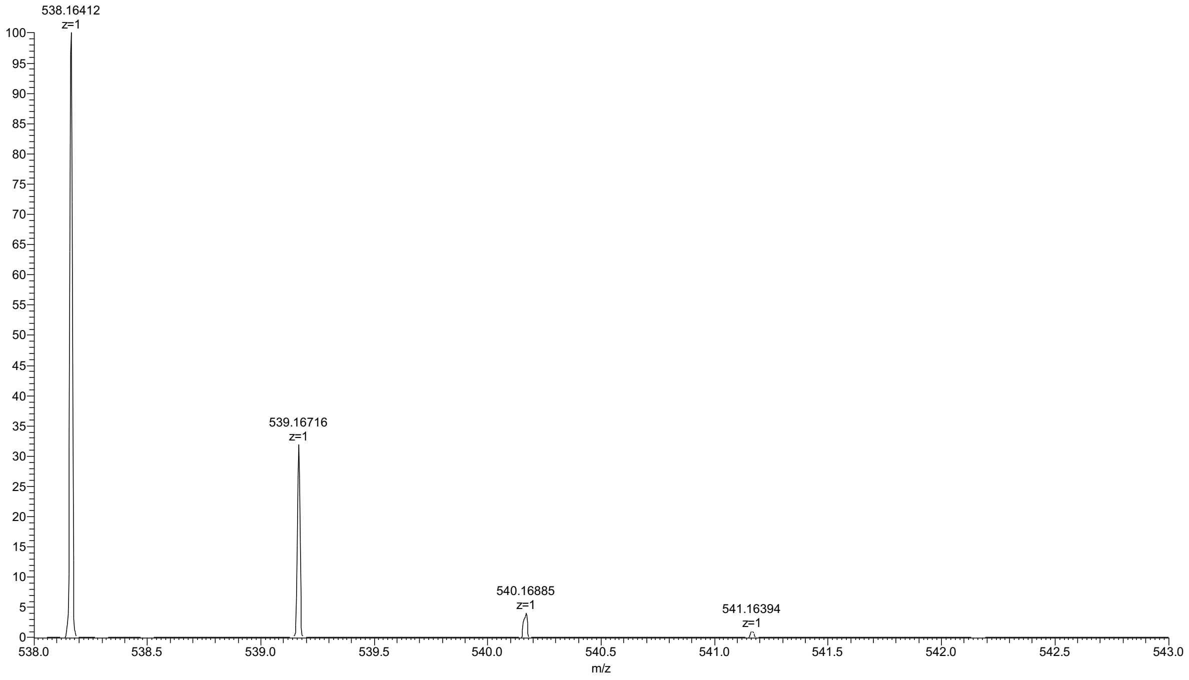

HRMS (ESI, m/z) of compound **L1**.

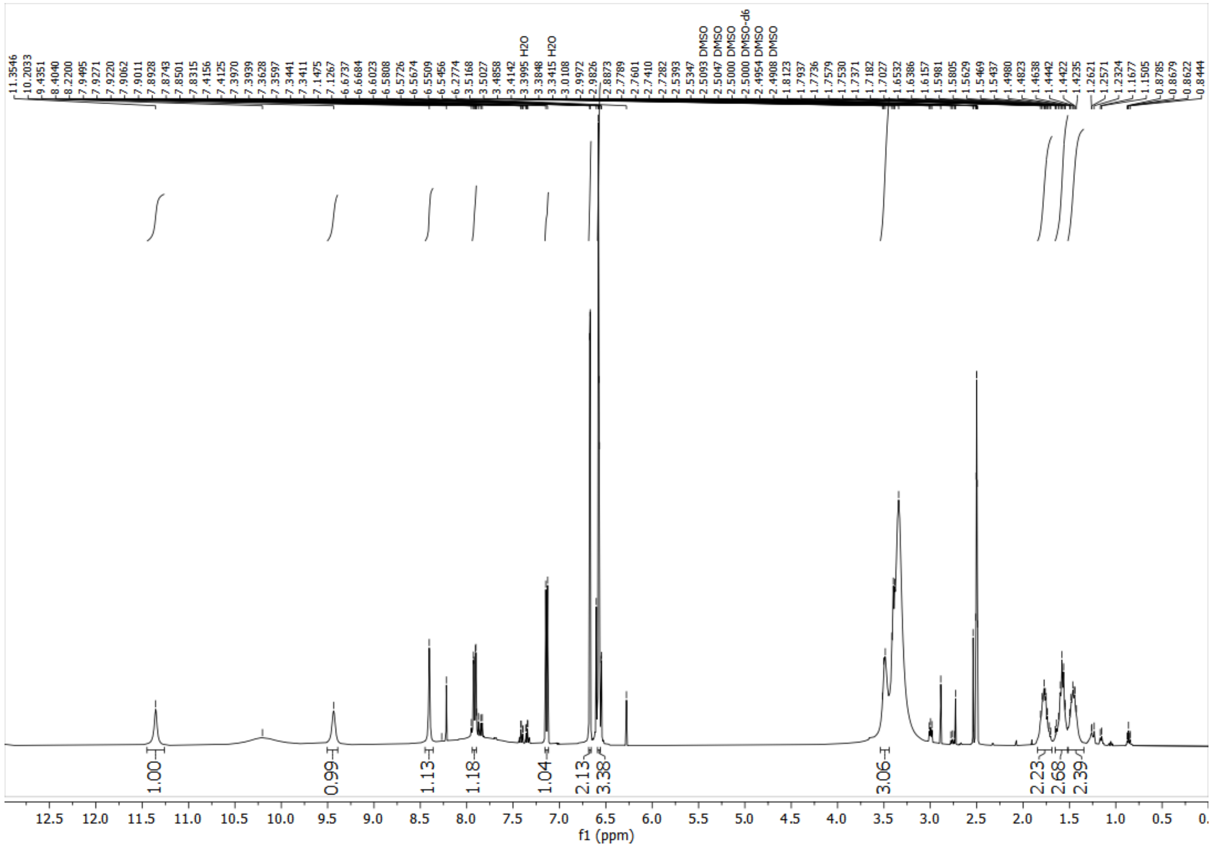

^{1}H NMR (400 MHz, DMSO-d_6) spectrum of compound **L1**.

^{1}H NMR (400 MHz, DMSO-d_6) δ 11.35 (s, 1H), 9.44 (s, 1H), 8.40 (s, 1H), 7.91 (dd, J = 8.4, 2.0 Hz, 1H), 7.14 (d, J = 8.3 Hz, 1H), 6.67 (d, J = 2.1 Hz, 2H), 6.60 - 6.55 (m, 3H), 3.49 (bs, 3H), 1.83 - 1.67 (m, 2H), 1.65 - 1.51 (m, 3H), 1.50 - 1.34 (q, J = 8.3, 7.8 Hz, 2H).

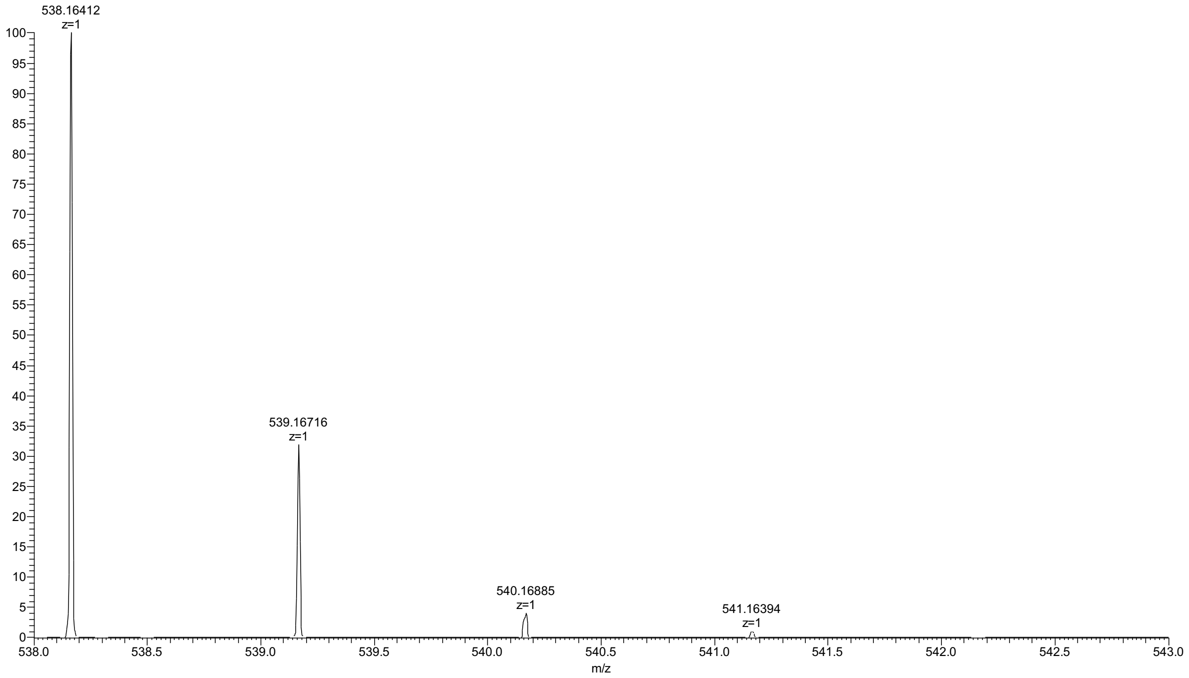

HRMS (ESI, m/z) of compound **L2**.

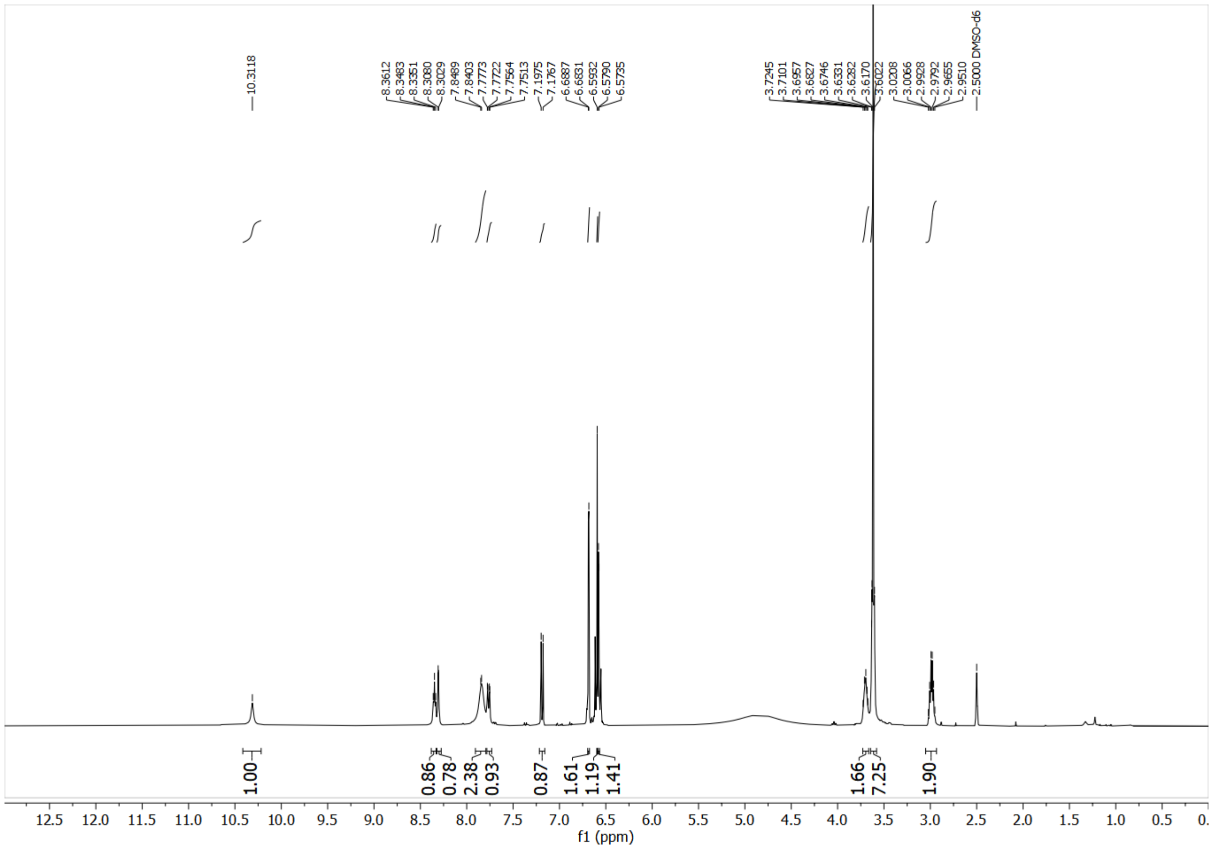

^{1}H NMR (400 MHz, DMSO-d_6) spectrum of compound **L2**.

^{1}H NMR (400 MHz, DMSO-d_6) δ 10.31 (s, 1H), 8.35 (t, J = 5.2 Hz, 1H), 8.31 (s, 1H), 7.84 (bs, 2H), 7.76 (d, J = 8.4, 1H), 7.19 (d, J = 8.3 Hz, 1H), 6.69 (d, J = 2.2 Hz, 2H), 6.59 (s, 1H), 6.58 (d, J = 2.2 Hz, 1H), 3.72 – 3.65 (m, 2H), 3.64 – 3.57 (m, 7H), 2.99 (p, J = 5.6 Hz, 2H).

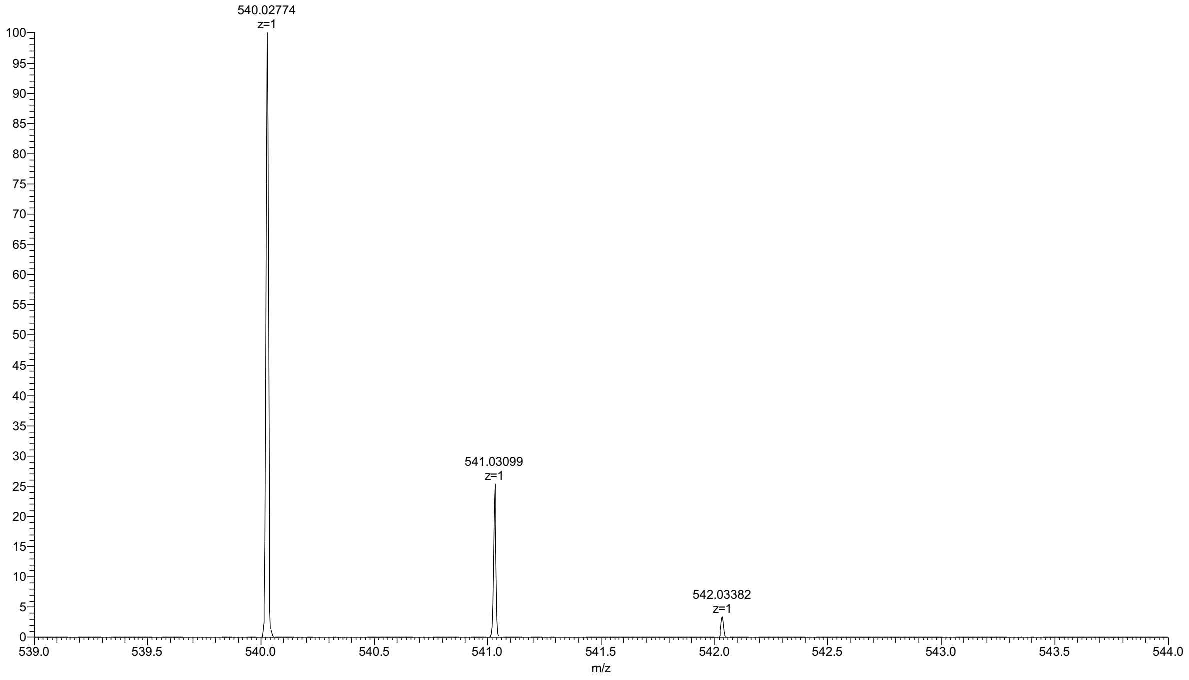

HRMS (ESI, m/z) of compound **s15a**.

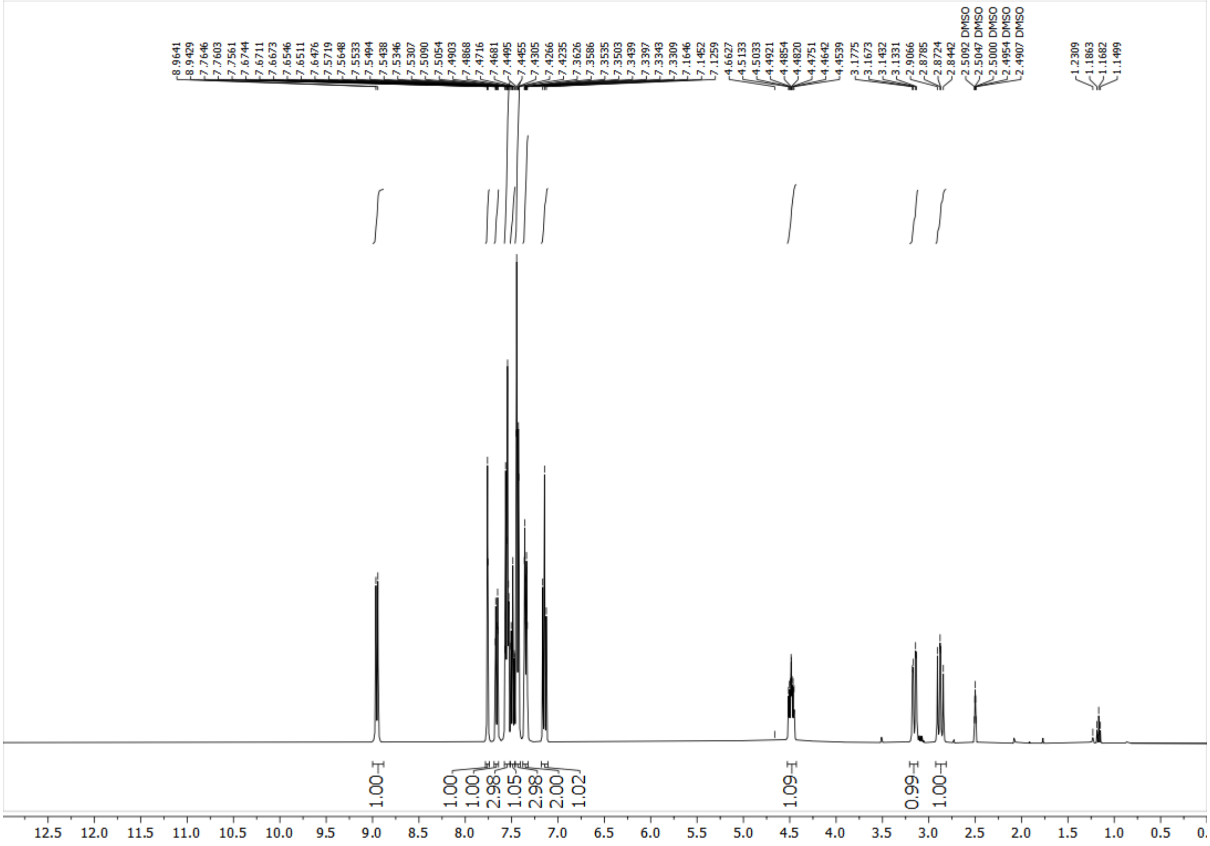

^{1}H NMR (400 MHz, DMSO-d_6) spectrum of compound **s15a**.

^{1}H NMR (400 MHz, DMSO-d_6) δ 8.95 (d, J = 8.5 Hz, 1H), 7.76 (t, J = 1.7 Hz, 1H), 7.66 (dt, J = 7.9, 1.4 Hz, 1H), 7.58 - 7.51 (m, 3H), 7.49 (td, J = 7.5, 1.4 Hz, 1H), 7.46 - 7.40 (m, 3H), 7.37 - 7.32 (m, 2H),

7.15 (t, J = 7.8 Hz, 1H), 4.48 (ddd, J = 11.2, 8.5, 4.0 Hz, 1H), 3.16 (dd, J = 13.7, 4.1 Hz, 1H), 2.88 (dd, J = 13.7, 11.3 Hz, 1H).

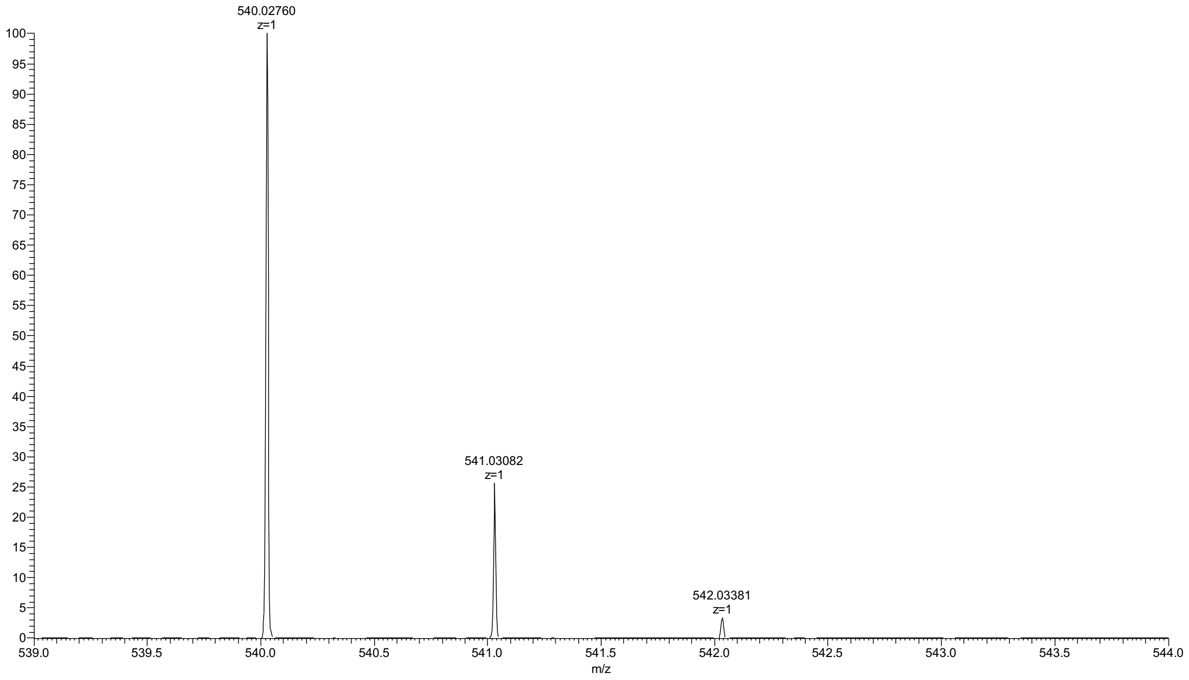

HRMS (ESI, m/z) of compound **s15b**.

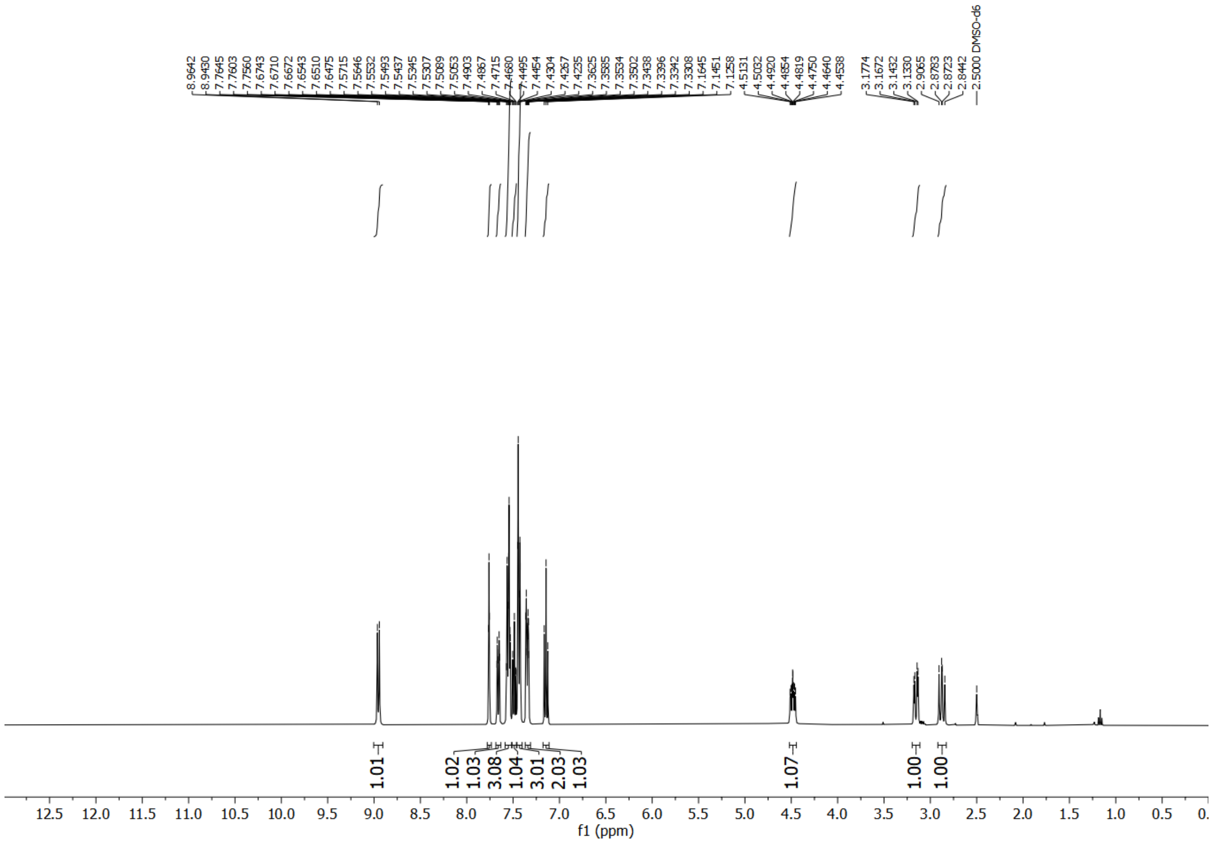

^{1}H NMR (400 MHz, DMSO-d_6) spectrum of compound **s15b**.

^{1}H NMR (400 MHz, DMSO-d_6) δ 8.95 (d, *J* = 8.5 Hz, 1H), 7.76 (t, *J* = 1.7 Hz, 1H), 7.66 (dt, *J* = 7.9, 1.4 Hz, 1H), 7.58 - 7.51 (m, 3H), 7.49 (td, *J* = 7.5, 1.4 Hz, 1H), 7.46 - 7.40 (m, 3H), 7.37 - 7.32 (m, 2H), 7.15 (t, *J* = 7.8 Hz, 1H), 4.48 (ddd, *J* = 11.2, 8.5, 4.0 Hz, 1H), 3.16 (dd, *J* = 13.7, 4.1 Hz, 1H), 2.88 (dd, *J* = 13.7, 11.3 Hz, 1H).

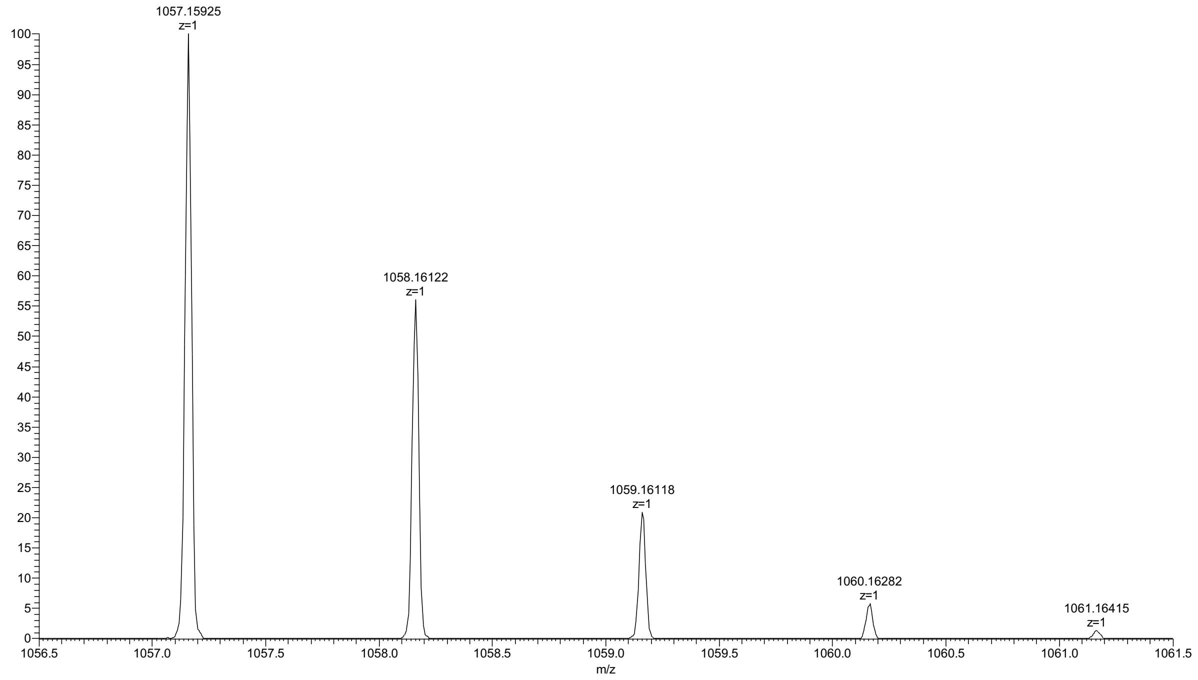

HRMS (ESI, m/z) of compound **26**.

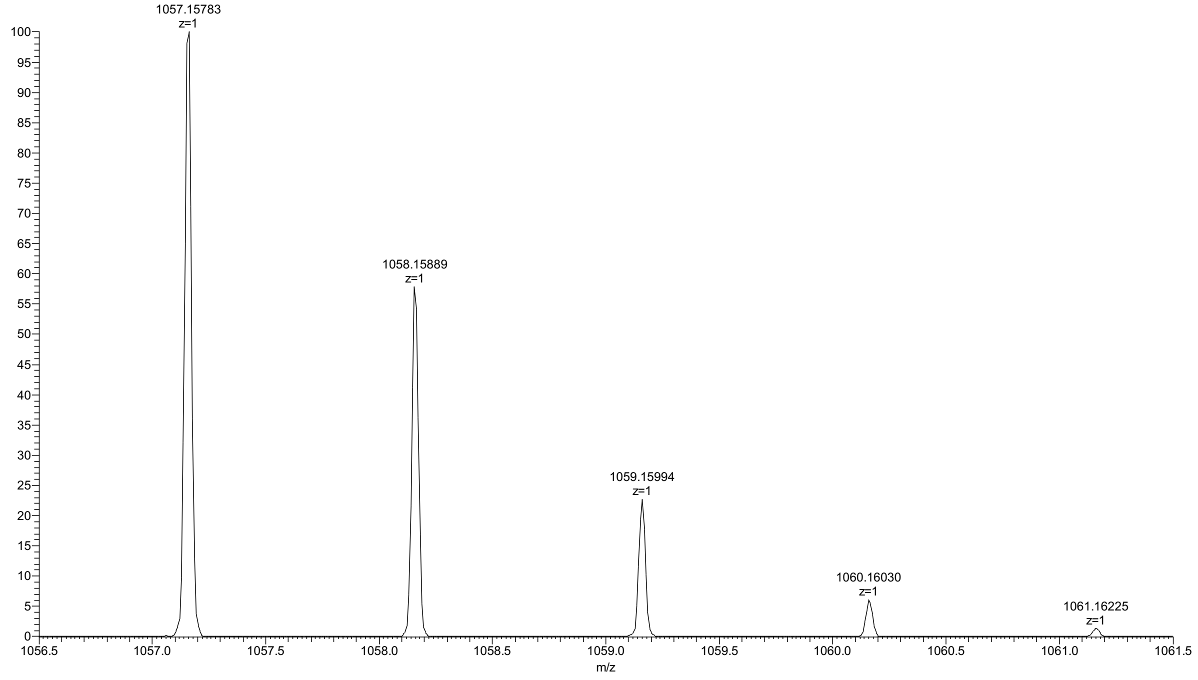

HRMS (ESI, m/z) of compound **27**.

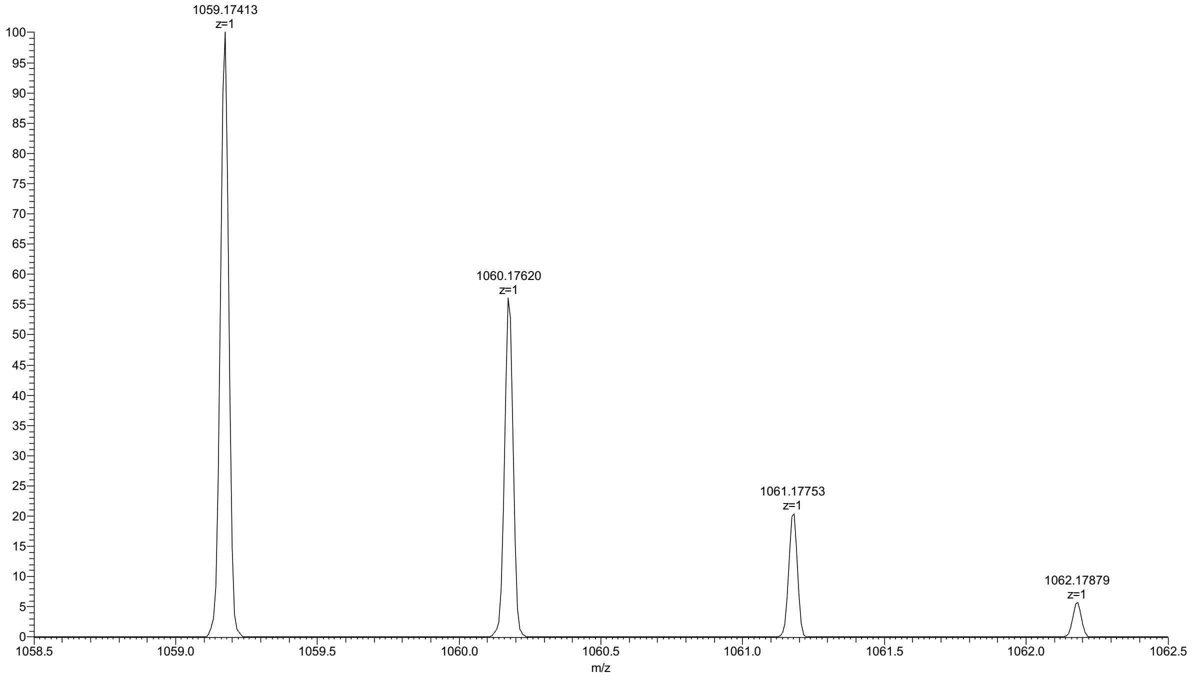

HRMS (ESI, m/z) of compound **28**.

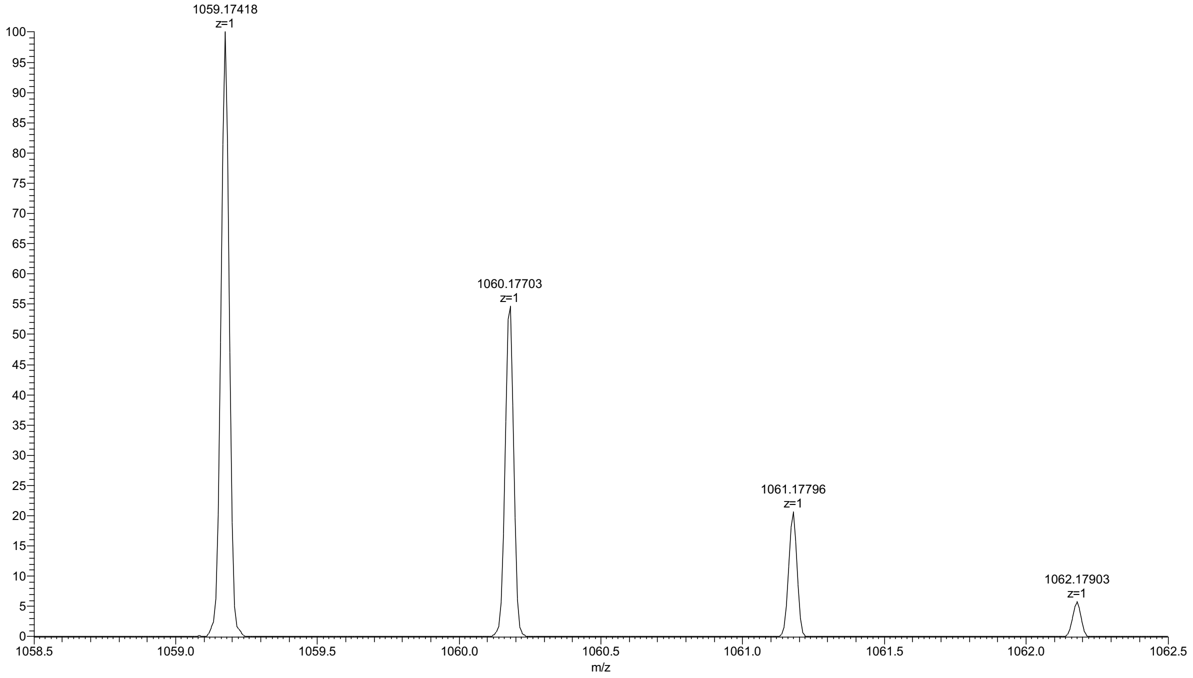

HRMS (ESI, m/z) of compound **29**.

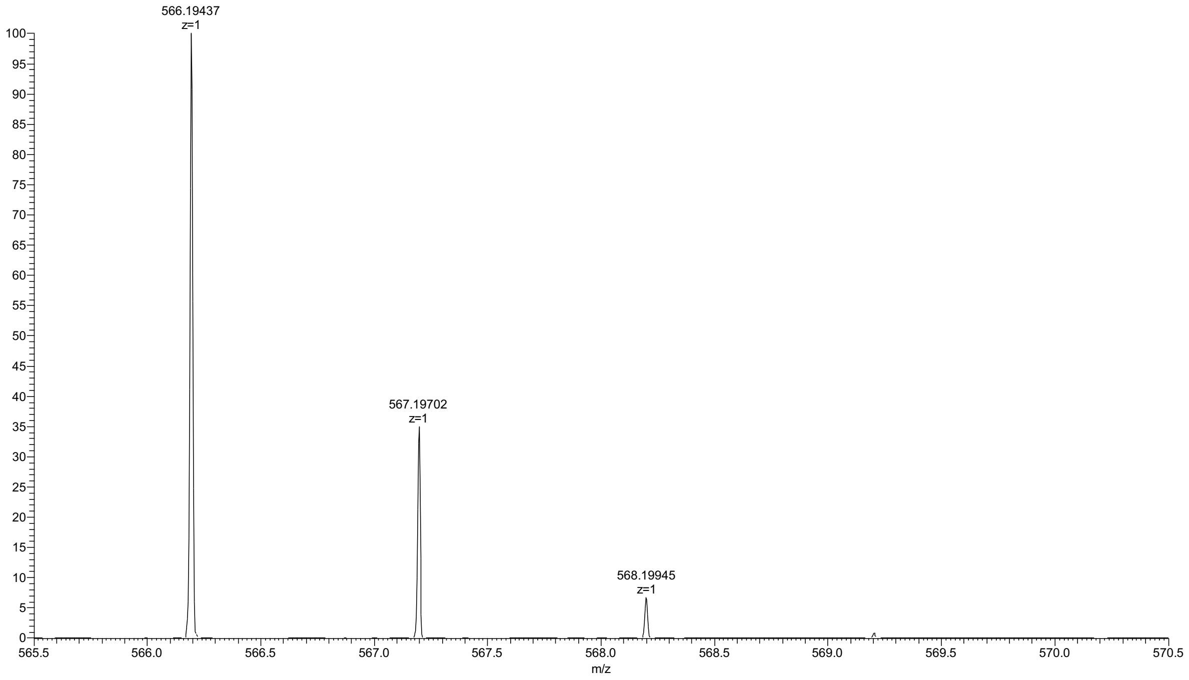

HRMS (ESI, m/z) of compound **s16a**.

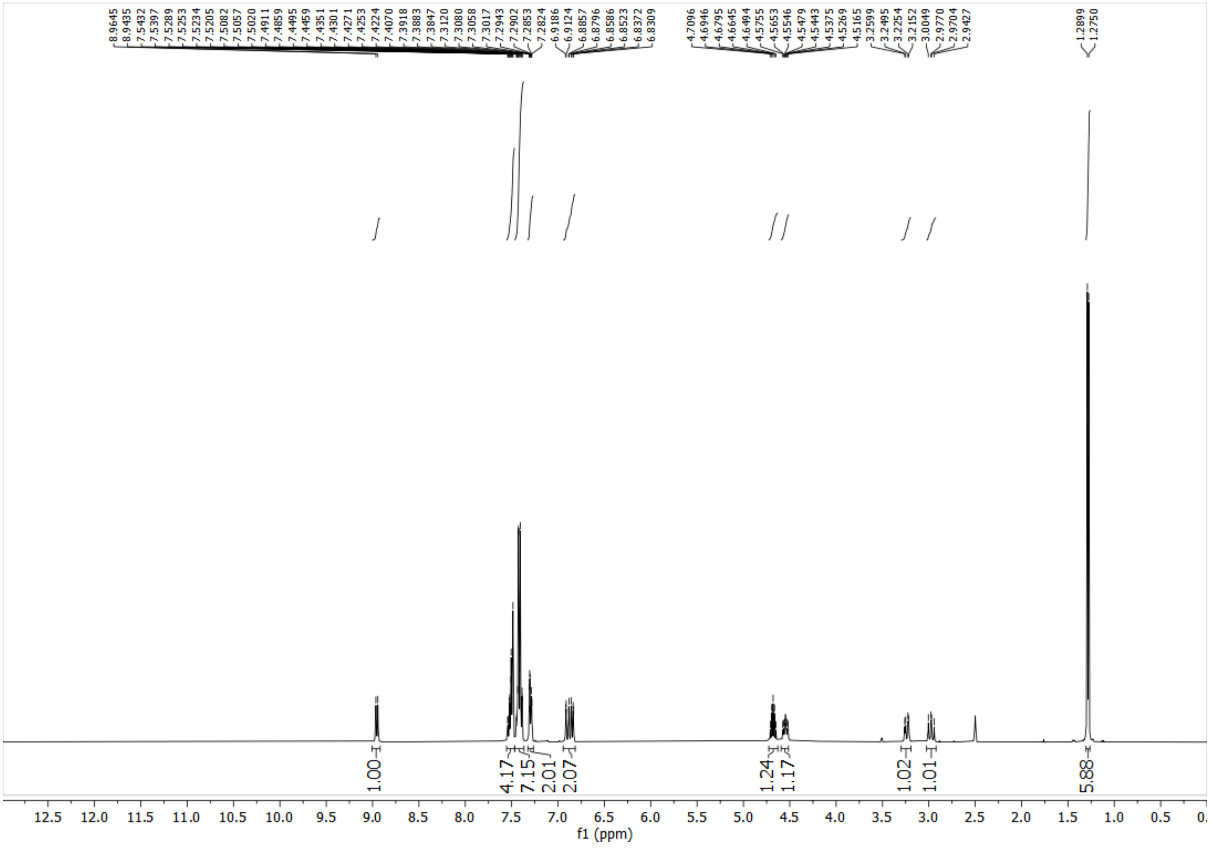

^{1}H NMR (400 MHz, DMSO-d_6) spectrum of compound **s16a**.

^{1}H NMR (400 MHz, DMSO-d_6) δ 8.95 (d, J = 8.4 Hz, 1H), 7.56 - 7.47 (m, 4H), 7.47 - 7.37 (m, 7H), 7.33 - 7.27 (m, J = 7.7, 2.8, 1.4 Hz, 2H), 6.94 - 6.81 (m, 2H), 4.68 (hept, J = 6.0 Hz, 1H), 4.55 (ddd, J = 11.1, 8.4, 4.1 Hz, 1H), 3.24 (dd, J = 13.7, 4.1 Hz, 1H), 2.97 (dd, J = 13.8, 11.1 Hz, 1H), 1.28 (d, J = 6.0 Hz, 6H).

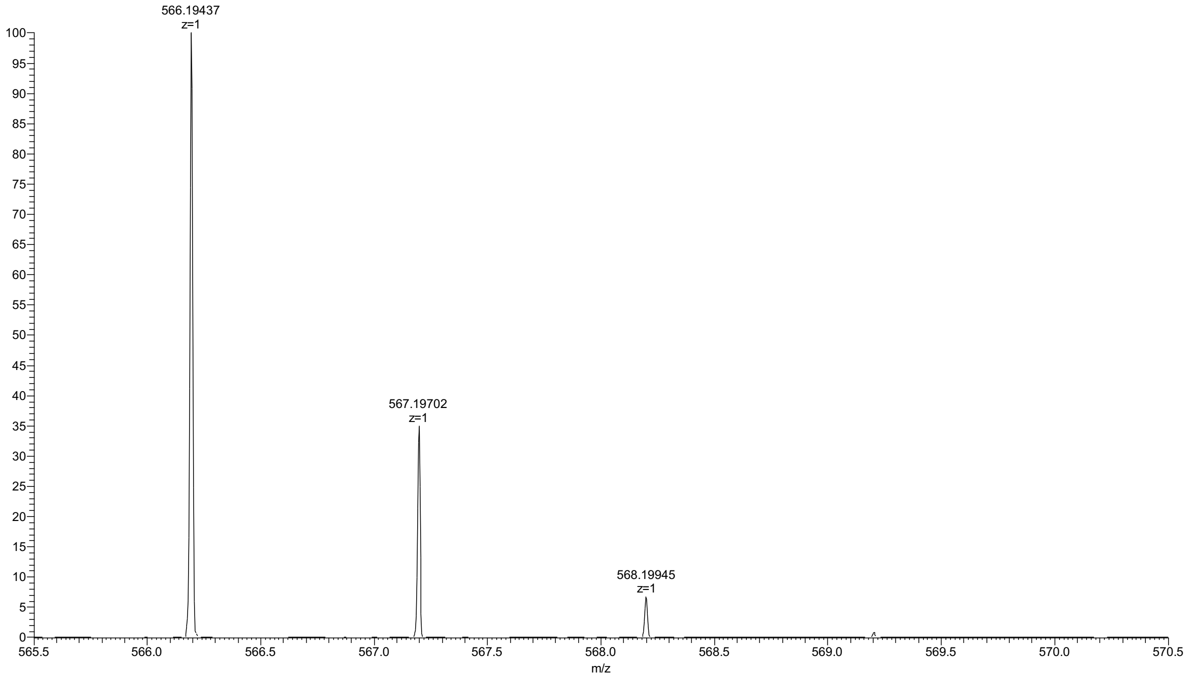

HRMS (ESI, m/z) of compound **s16b**.

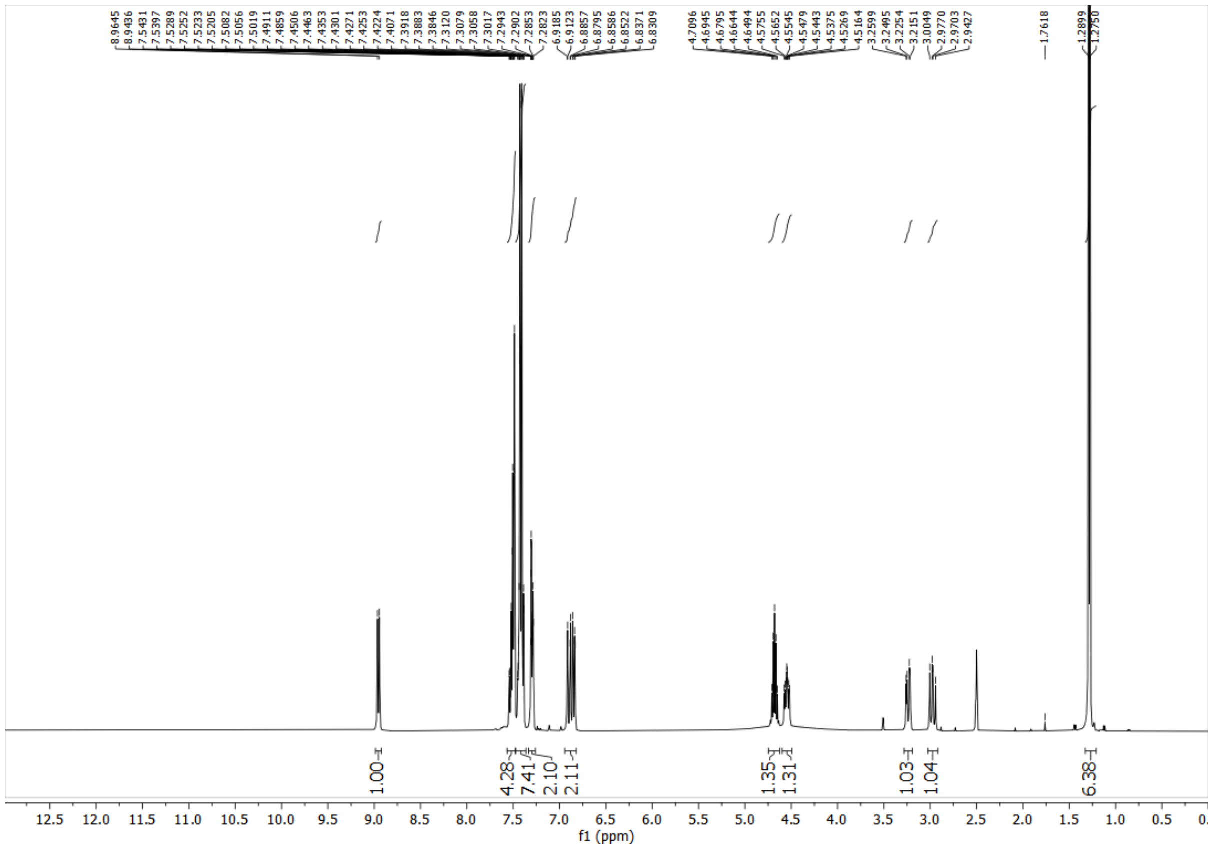

^{1}H NMR (400 MHz, DMSO-d_6) spectrum of compound **s16b**.

^{1}H NMR (400 MHz, DMSO-d_6) δ 8.95 (d, J = 8.4 Hz, 1H), 7.56 - 7.47 (m, 4H), 7.47 - 7.37 (m, 7H), 7.33 - 7.27 (m, J = 7.7, 2.8, 1.4 Hz, 2H), 6.94 - 6.81 (m, 2H), 4.68 (hept, J = 6.0 Hz, 1H), 4.55 (ddd, J = 11.1, 8.4, 4.1 Hz, 1H), 3.24 (dd, J = 13.7, 4.1 Hz, 1H), 2.97 (dd, J = 13.8, 11.1 Hz, 1H), 1.28 (d, J = 6.0 Hz, 6H).

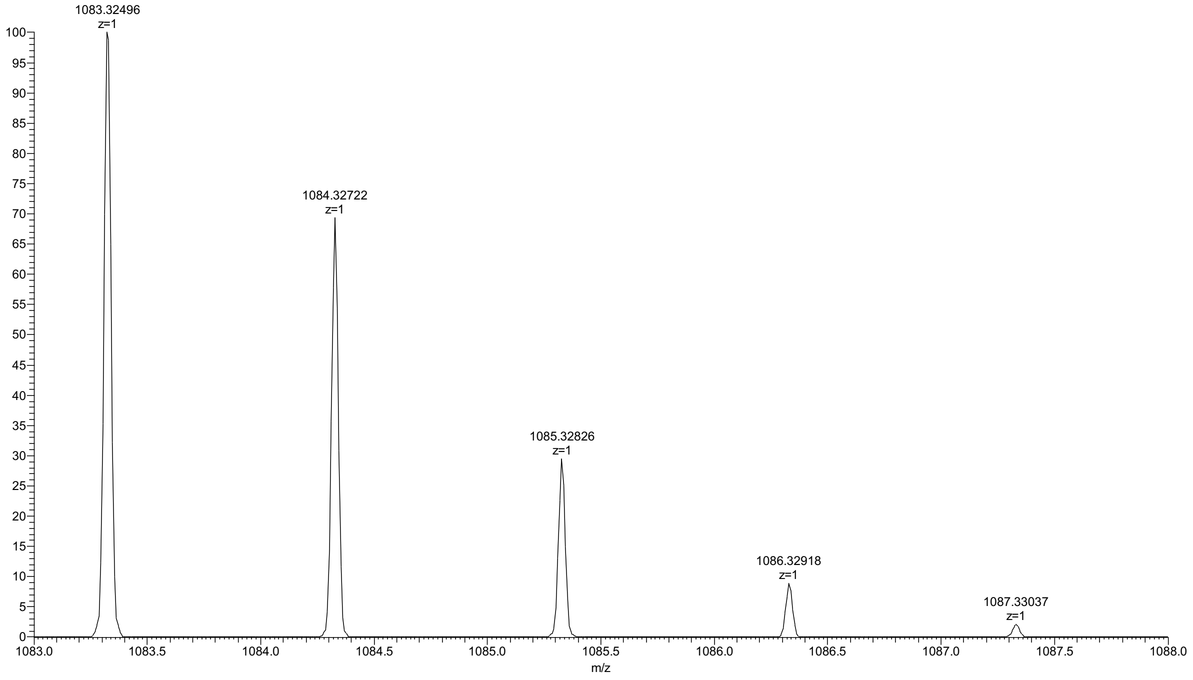

HRMS (ESI, m/z) of compound **30**.

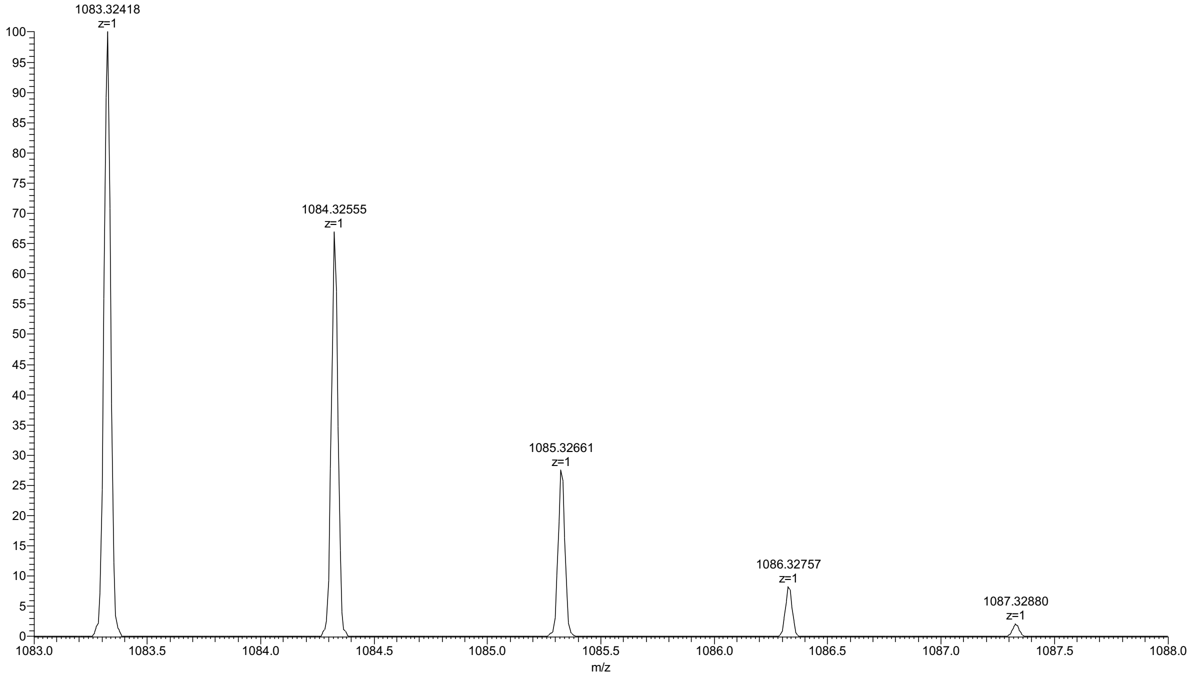

HRMS (ESI, m/z) of compound **31**.

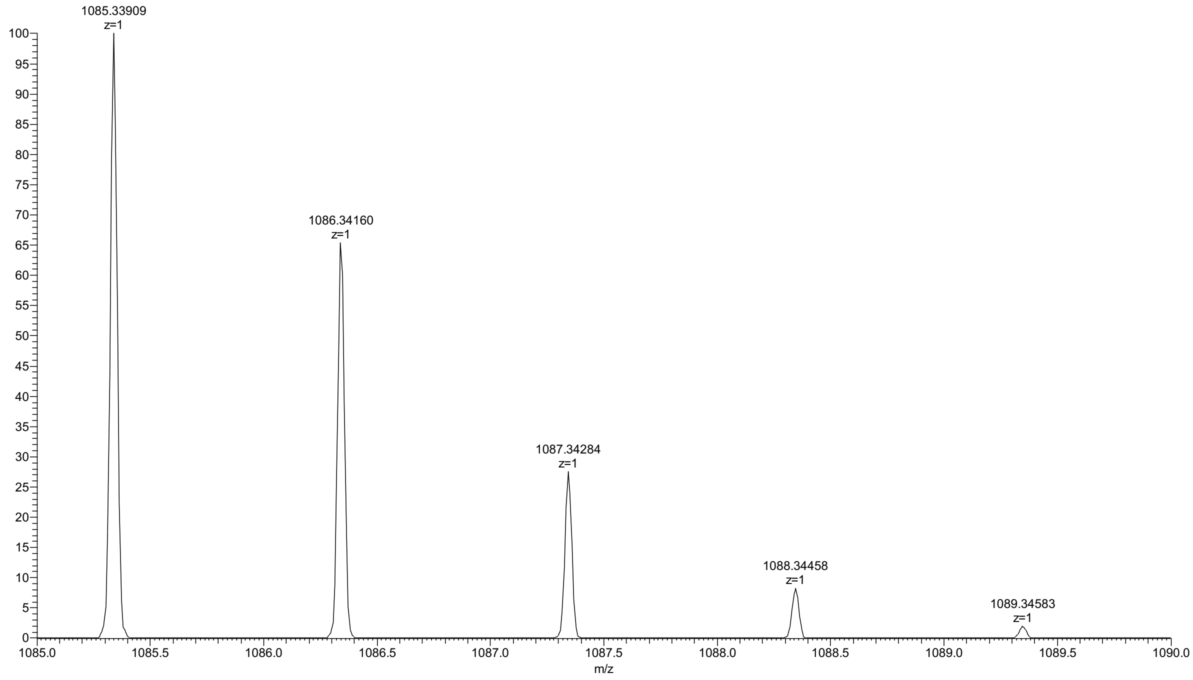

HRMS (ESI, m/z) of compound **32**.

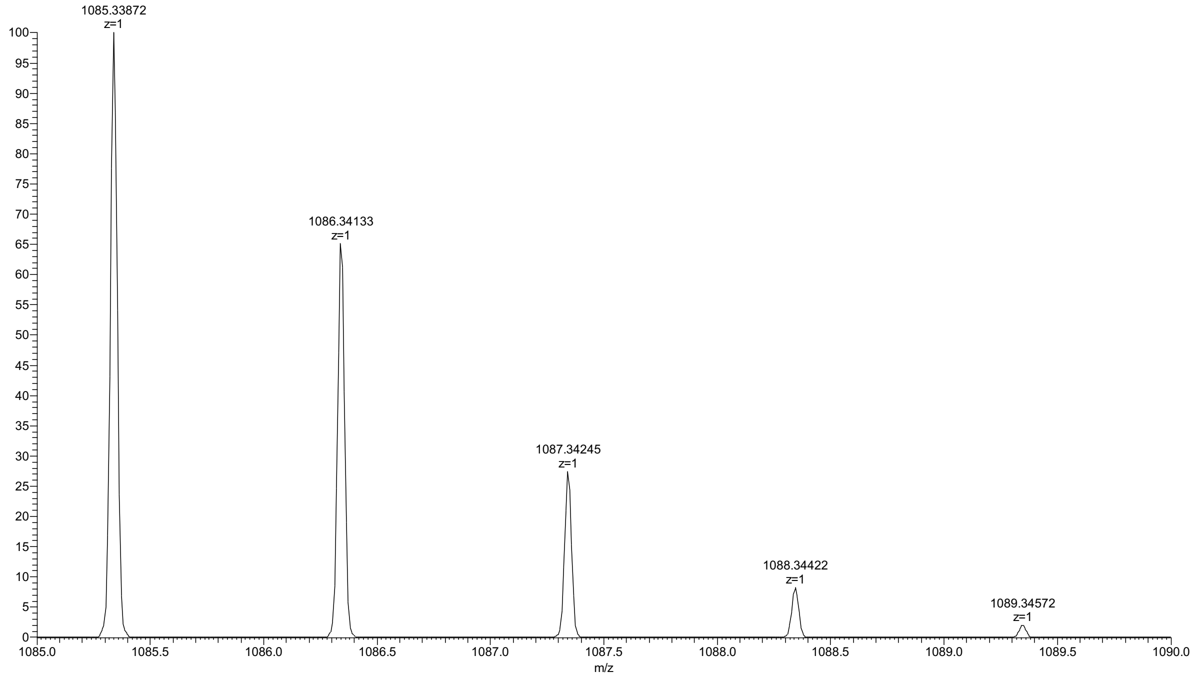

HRMS (ESI, m/z) of compound **33**.

8 References

1. Pedretti, A., Mazzolari, A., Gervasoni, S., Fumagalli, L. & Vistoli, G. The VEGA suite of programs: an versatile platform for cheminformatics and drug design projects. *Bioinformatics* **37**, 1174–1175 (2021).
2. Stewart, J. J. P. Optimization of parameters for semiempirical methods VI: more modifications to the NDDO approximations and re-optimization of parameters. *J Mol Model* **19**, 1–32 (2013).
3. Alterio, V. *et al.* Crystal structure of the catalytic domain of the tumor-associated human carbonic anhydrase IX. *Proceedings of the National Academy of Sciences* **106**, 16233–16238 (2009).
4. Schäfer, M. *et al.* Structure–Activity Relationships and Biological Insights into PSMA-617 and Its Derivatives with Modified Lipophilic Linker Regions. *ACS Omega* **10**, 7077–7090 (2025).
5. Phillips, J. C. *et al.* Scalable molecular dynamics with NAMD. *J Comput Chem* **26**, 1781–1802 (2005).
6. Korb, O., Stützle, T. & Exner, T. E. Empirical Scoring Functions for Advanced Protein–Ligand Docking with PLANTS. *J Chem Inf Model* **49**, 84–96 (2009).
7. Papeo, G. *et al.* Insights into PARP Inhibitors' Selectivity Using Fluorescence Polarization and Surface Plasmon Resonance Binding Assays. *SLAS Discovery* **19**, 1212–1219 (2014).
8. Wichert, M. *et al.* Dual-display of small molecules enables the discovery of ligand pairs and facilitates affinity maturation. *Nat Chem* **7**, 241–249 (2015).
9. Georgiev, T. *et al.* Discovery of high-affinity ligands for prostatic acid phosphatase via DNA-encoded library screening enables targeted cancer therapy. *Nature Biomedical Engineering,* (2025) doi: 10.1038/s41551-025-01432-6.
10. Migliorini, F., Ciamarone, A., Neri, D., Cazzamalli, S. & Favalli, N. Optimization of Reaction Conditions for On-DNA Synthesis of Ureas, Thioureas, and Sulfonamides. *Asian J Org Chem* (2024) doi:10.1002/ajoc.202400556.
11. Oehler, S. *et al.* A DNA-encoded chemical library based on chiral 4-amino-proline enables stereospecific isozyme-selective protein recognition. *Nat Chem* **15**, 1431–1443 (2023).
12. Decurtins, W. *et al.* Automated screening for small organic ligands using DNA-encoded chemical libraries. *Nat Protoc* **11**, 764–780 (2016).
13. Lucaroni, L. *et al.* DNA-encoded chemical libraries enable the discovery of potent PSMA-ligands with substantially reduced affinity towards the GCPIII anti-target. *Chem Sci* **15**, 6789–6799 (2024).
14. Torng, W. *et al.* Deep Learning Approach for the Discovery of Tumor-Targeting Small Organic Ligands from DNA-Encoded Chemical Libraries. *ACS Omega* **8**, 25090–25100 (2023).
15. Puca, E. *et al.* The antibody-based delivery of interleukin-12 to solid tumors boosts NK and CD8 $^{+}$ T cell activity and synergizes with immune checkpoint inhibitors. *Int J Cancer* **146**, 2518–2530 (2020).
16. Rotta, G. *et al.* A novel strategy to generate immunocytokines with activity-on-demand using small molecule inhibitors. *EMBO Mol Med* **16**, 904–926 (2024).
